# Oxetan-3-ols as 1,2-bis-Electrophiles in a Brønsted-Acid-Catalyzed Synthesis of 1,4-Dioxanes

**DOI:** 10.1021/acs.orglett.2c00568

**Published:** 2022-03-21

**Authors:** Juan J. Rojas, Elena Torrisi, Maryne A. J. Dubois, Riashat Hossain, Andrew J. P. White, Giovanni Zappia, James J. Mousseau, Chulho Choi, James A. Bull

**Affiliations:** † Department of Chemistry, Imperial College London, Molecular Sciences Research Hub, White City Campus, Wood Lane, London W12 0BZ, U.K.; ‡ Department of Biomolecular Sciences, School of Pharmacy, University of Urbino “Carlo Bo”, P.za Rinascimento, 6, 61029 Urbino (PU), Italy; § Pfizer Worldwide Research, Development and Medical, Eastern Point Road, Groton, Connecticut 06340, United States

## Abstract

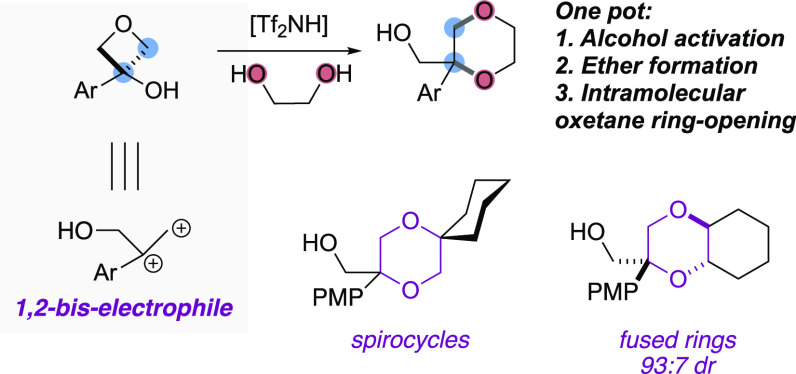

Annulations that combine diacceptors with bis-nucleophiles are uncommon. Here, we report the synthesis of 1,4-dioxanes from 3-aryloxetan-3-ols, as 1,2-bis-electrophiles and 1,2-diols. Brønsted acid Tf_2_NH catalyzes both the selective activation of the oxetanol, to form an oxetane carbocation that reacts with the diol, and intramolecular ring opening of the oxetane. High regio- and diastereoselectivity are achieved with unsymmetrical diols. The substituted dioxanes and fused bicyclic products present interesting motifs for drug discovery and can be further functionalized.

Annulation reactions combine two functionalized components to construct valuable ring systems, often in one pot.^1^ These take various forms, but typically, reactants will each contain nucleophilic and electrophilic sites, such as the Robinson annulation, or proceed in a concerted manner such as the Diels–Alder reaction. More unusual is the involvement of bis-electrophiles and bis-nucleophiles. Examples that successfully form substituted saturated rings through the combination of diacceptor fragments with bis-nucleophiles are rare.^2−4^ This is due to the low occurrence of reactive bis-electrophiles, whereas conversely, 1,2-bis-nucleophiles are readily available. Hence, methods to exploit new bis-electrophiles offer the potential to rapidly access new chemical space.

The 1,4-dioxane ring is an important class of saturated heterocycle and is present in a wide range of bioactive compounds (Figure 1A).^5^ Cyclic sp^3^-rich fragments have received increased recent interest in medicinal chemistry given the potential positive effect on pharmacokinetic properties and three-dimensional scaffolding.^6^ Despite this, synthetic methods to access 1,4-dioxanes are limited, and multistep processes are often required.^7^ Typically, complex hydroxy-ether precursors bearing a leaving group or pseudoleaving group (e.g., an epoxide) are prepared through lengthy synthetic sequences to assemble the 1,4-dioxane ring through an intramolecular cyclization (Figure 1B).^8^ Such strategies do not readily allow the rapid generation of further analogues that may be necessary in library synthesis in medicinal chemistry, as each example requires a separate synthetic sequence.

**Figure 1:**
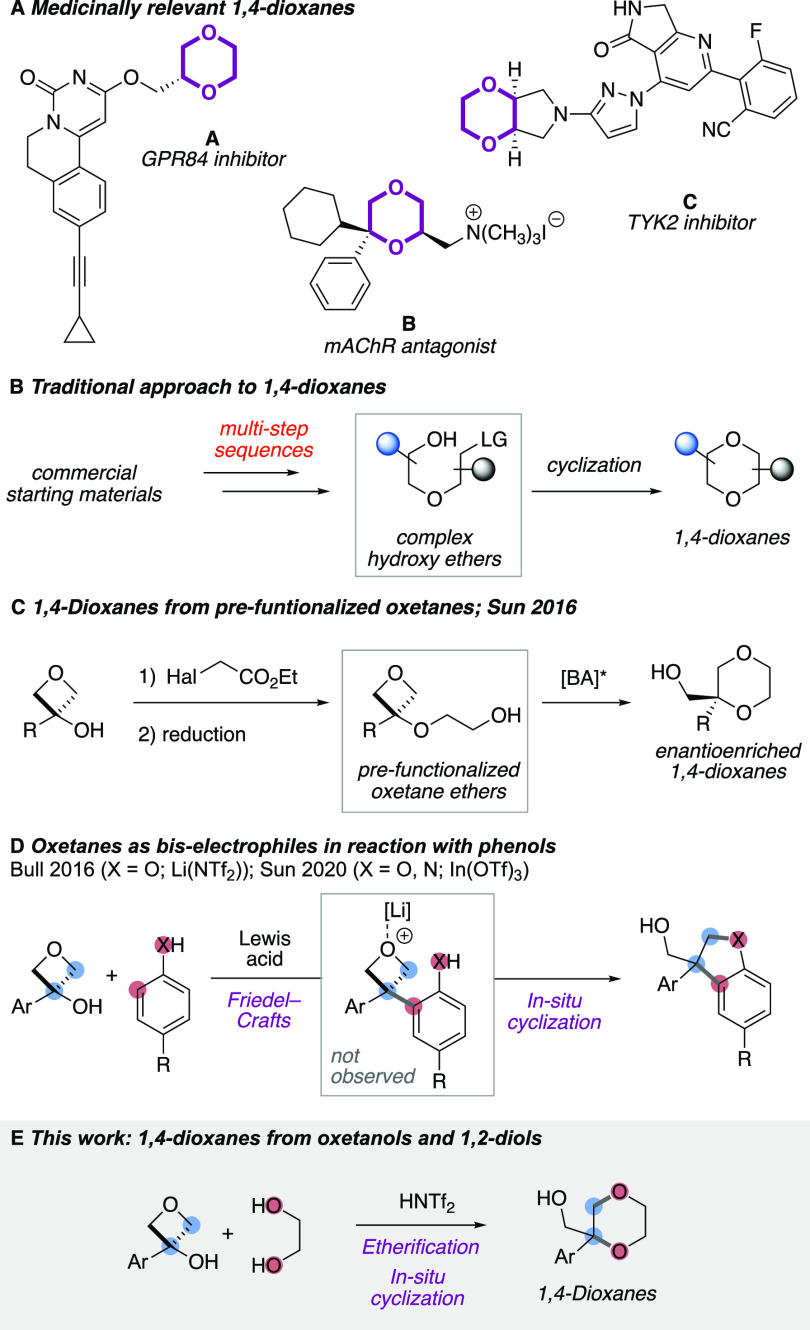
(A) Medicinally relevant 1,4-dioxane rings. (B) Traditional synthetic approaches. (C) Synthesis of 1,4-dioxanes from prefunctionalized oxetane ethers. [BA]* = chiral Brønsted acid catalyst. (D) Lewis-acid-catalyzed synthesis of dihydrobenzofurans and indolines from oxetanols and phenols. (E) This work: synthesis of 1,4-dioxanes directly from oxetanols and 1,2-diols using Brønsted acid catalysis.

Oxetanes offer intriguing potential as synthetic intermediates due to their moderate ring strain (106 kJ mol^–1^; cf. 112 kJ mol^–1^ for epoxides and 25 kJ mol^–1^ for THFs),^9^ which can be modulated by substituents. 3,3-Disubstituted oxetanes display high stability toward external nucleophiles, which has led to this substitution pattern in particular being adopted in medicinal chemistry.^10,11^ However, they can remain susceptible to ring opening by internal nucleophiles (i.e., intramolecular processes), especially under acidic conditions.^11,12^ This intramolecular cyclization strategy has been successfully employed for the synthesis of heterocycles from prefunctionalized oxetane intermediates.^13,14^ In particular, Sun has exploited this in the enantioselective syntheses of heterocycle derivatives employing a chiral phosphoric acid catalyst. This has included the enantioselective synthesis of 1,4-dioxanes from preformed hydroxy-ether-containing oxetanes (Figure 1C).^15^ Kuduk recently reported tandem amination and oxetane opening for the preparation of benzomorpholines.^16^

Recently, oxetanols have displayed potential to operate as bis-electrophiles. We have developed methods for the formation of oxetane carbocations using Lewis acid catalysts to dehydrate 3-aryloxetan-3-ols.^17,18^ Specifically, reaction with 4-substituted phenols gave a Friedel–Crafts reaction at the 2-position of the phenol and was followed by opening of the oxetane ring by the phenolic oxygen under the Lewis acidic conditions to yield dihydrobenzofurans (Figure 1D).^17^ Similarly, Sun reported the synthesis of indolines using In(OTf)_3_ as a Lewis acid catalyst.^19^

Here, we report the activation of oxetanols with HNTf_2_ as a Brønsted acid catalyst with 1,2-diols as bis-nucleophiles to yield functionalized 1,4-dioxanes (Figure 1E). This provides an unusual annulation reaction exploiting readily available diol substrates suitable for divergent synthesis, including cyclic diols to form saturated bicyclic heterocycles. The reaction occurs diastereoselectively, is metal-free, and generates water as the only byproduct.

Initial attempts to use diols with our previously reported conditions using Li catalysis, as successful for phenol nucleophiles, showed no reaction between 4-methoxyphenyl oxetanol **1a** and ethylene glycol (Table 1, entry 1). Only starting material **1a** was recovered which was attributed to chelation of the diols to the metal catalyst that led to deactivation.^20^

**Table 1:**
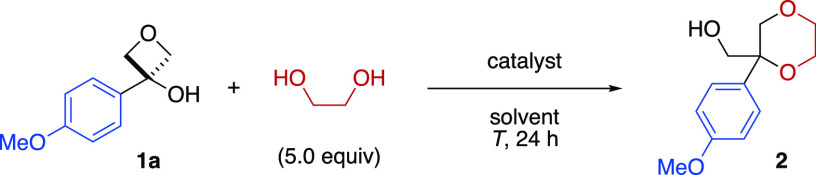
Selected Optimization for the Formation of 1,4-Dioxane **2** from Oxetanol **1a** and Ethylene Glycol

entry^a^	catalyst (mol %)	*T* (°C)	solvent (concN; M)	yield (%)^b^
1	Li(NTf_2_) (11)^c^	40	CHCl_3_ (0.5)	0 [RSM]
2^d^	TfOH (5)	40	CHCl_3_ (0.5)	42
3^d^	TfOH (5)	40	CH_2_Cl_2_ (0.5)	55
4^d^	TfOH (5)	40	toluene (0.5)	68
5^d^	TfOH (10)	40	toluene (0.5)	73
6^d^	TfOH (10)	40	MeCN (0.5)	84
7	Tf_2_NH (10)	40	MeCN (0.5)	86
8	Tf_2_NH (10)	40	MeCN (0.3)	91
**9**	**Tf**_**2**_**NH (10)**	**50**	**MeCN (0.3)**	**95 (91)**^e^
10^f^	Tf_2_NH (10)	50	MeCN (0.3)	90
11^g^	Tf_2_NH (10)	50	MeCN (0.3)	80

a Reactions run on a 0.25 mmol scale.

b Yield calculated by analysis of the ^1^H NMR spectrum of the crude mixture of the reaction using 1,3,5-trimethoxybenzene as an internal standard. Isolated yields in parentheses.

c With 5.5 mol % of Bu_4_NPF_6_ as an additive.

d Reaction run for 16 h. It was shown that there is no difference in yield between 16 and 24 h (Supporting Information Table S1).

e Isolated on a 0.91 mmol scale in a separate reaction.

f Using 3.0 equiv of ethylene glycol.

g Using 1.0 equiv of ethylene glycol. RSM = Returned starting material. See Supporting Information Table S1 for full optimization details.

Other Lewis acids were similarly unsuccessful. Instead, we investigated strong Brønsted acids.^21^ Using catalytic TfOH, we were delighted to obtain dioxane **2** in 42% yield (entry 2). A switch to toluene as solvent and an increase in catalyst loading to 10 mol % further improved the yield (entries 3–5). Acetonitrile was then investigated as a more polar solvent that could stabilize the oxetane carbocation and solubilize polar substrates (entry 6). The acid catalyst was changed from TfOH (a fuming liquid) to the more practical Tf_2_NH (a solid; entry 7).^22^ Further small modifications in temperature and concentration led to the optimal conditions with a yield of 95% of **2** (entries 8–9). Interestingly, no products from the Ritter reaction, i.e., attack of acetonitrile at the carbocation, were observed when conducting the reaction in the absence of nucleophile (Supporting Information Table S1). Importantly, though 5 equiv of nucleophile led to the highest yields of **2**, lowering the equivalents of diol to 3 or 1 maintained a high yield (entries 10 and 11). Using the diol as a limiting reagent with a slight excess of oxetanol (1.3 equiv) led to 96% of 1,4-dioxane **2** (Supporting Information Table S1).

With optimized conditions in hand, the scope of the reaction was explored with a series of oxetanols and 1,2-diols (Scheme 1).

**Scheme 1:**
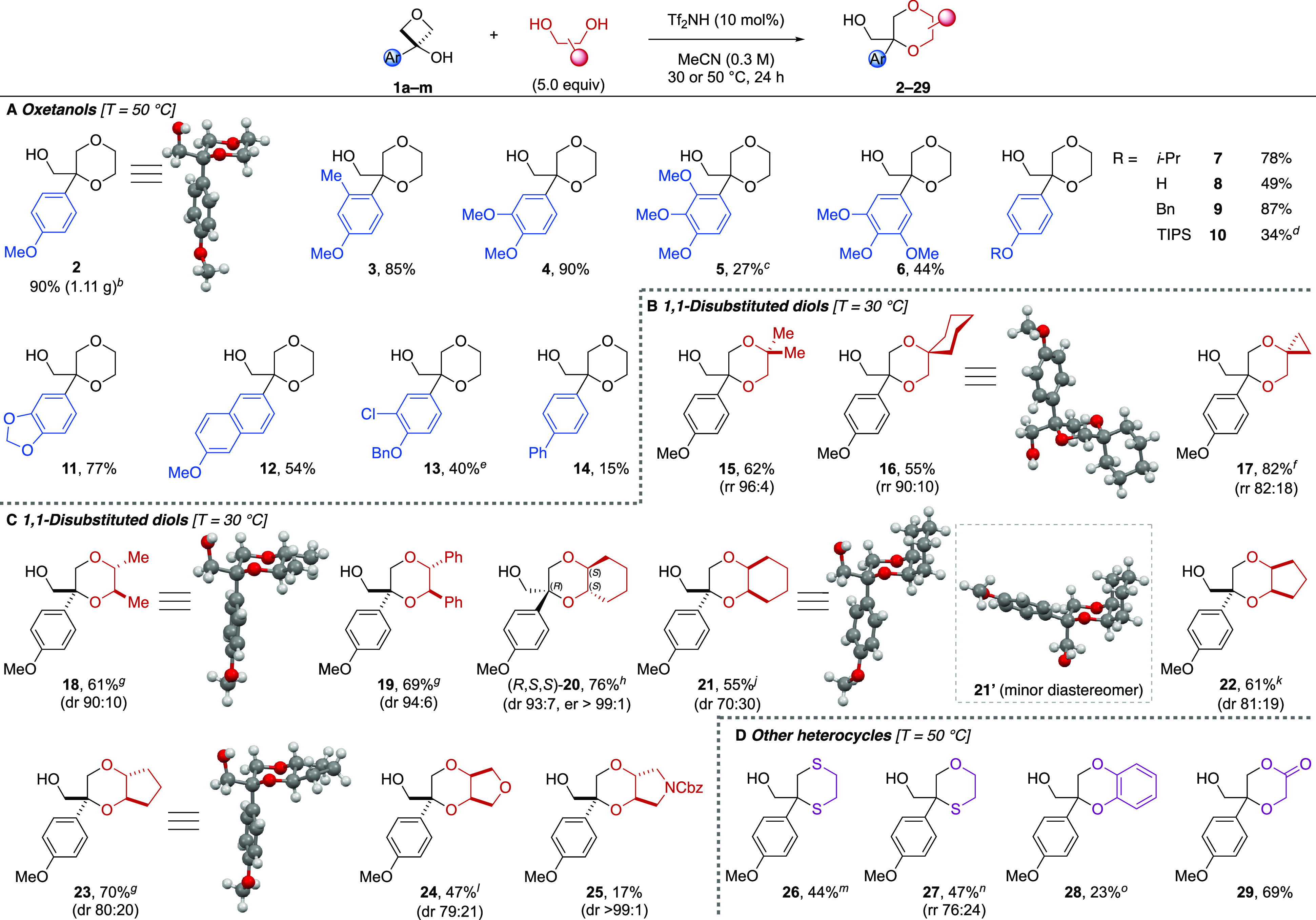
Annulation of Oxetanols and 1,2-Diols for the One-Pot Formation of 1,4-Dioxanes Reactions on a 0.25 mmol scale unless otherwise stated. Isolated yields are reported. Diastereomeric (dr) and regioisomeric (rr) ratios determined from the ^1^H NMR spectrum of the crude reaction mixture. Reaction run on a 5.5 mmol scale. Reaction run on a 0.22 mmol scale. 18% of phenol **8** was also isolated. Reaction run for 32 h. Reaction run on a 0.136 mmol scale and the product isolated as a mixture of regioisomers with the indicated rr. Product isolated as a mixture of diastereomers with the indicated dr. An additional 11% of a diastereomeric mixture was isolated (dr 67:33). Reaction run at 50 °C. Additional 10% of a diastereomeric mixture was isolated (dr 26:74). Additional 20% of a diastereomeric mixture was isolated (dr 39:61). Reaction run at 0–30 °C and using 1.2 equiv of bis-nucleophile (see Supporting Information Table S3). Reaction run on a 0.38 mmol scale (oxetanol) at 0–23 °C and using 0.75 equiv of bis-nucleophile (see Supporting Information Table S4). Yield based on bis-nucleophile. Using TfOH (5 mol %) in CHCl_3_ (0.5 M) at 25 °C (see Supporting Information Table S5).

PMP-dioxane **2** was obtained in 90% yield on a 5.5 mmol scale, generating 1.11 g of the desired product and highlighting the scalability of the protocol. Further substitution patterns were tolerated in moderate to high yields with electron-rich aromatic substituents (**3**–**10**). The successful reaction of *ortho*-substituted examples **3** and **5** is noteworthy because in the presumed planar carbocation structure *ortho*-substituents may clash with the oxetane methylene groups.^23^ Dioxane **6** bears the 3,4,5-trimethoxyphenyl pharmacophore, a motif present in prominent bioactive compounds such as colchicine, mescaline, and eudesmic acid derivatives but which has been challenging to activate through an oxetane carbocation.^18,23^ A different alkoxy substituent was tolerated (**7**), as well as free (**8**) and protected phenols (**9**–**10**). TIPS-protected dioxane **10** was partially deprotected by catalytic amounts of the acid catalyst. Interestingly, other aromatic rings like 1,3-benzodioxole and methoxynaphthalene were incorporated in good yields (**11** and **12**), as well as less electron-rich substrates, albeit in reduced yields (**13** and **14**).

Next, the scope of 1,2-diols was explored (Scheme 1B,C). The reaction temperature was lowered to 30 °C to improve diastereo- and regioselectivities without suffering from a reduced yield. Further improved dr was obtained at 0 °C but in lower yields (Supporting Information Table S2). 1,1-Disubstituted 1,2-diols were successful coupling partners, and 1,4-dioxanes were obtained in good yields and excellent regioisomeric ratios (**15**–**17**; Scheme 1B). Interesting spirocyclic dioxanes were synthesized by employing cyclic 1,1-disubstituted diols as nucleophiles. Monosubstituted diols led to a mixture of regio- and diastereoisomers (Supporting Information Scheme S1).

A series of acyclic and cyclic *cis*- and *trans*-1,2-disubstitued diols were probed to obtain monocyclic (**18** and **19**) and bicyclic dioxanes (**20**–**25**) in useful yields and high diastereoselectivities (Scheme 1C; see Supporting Information Scheme S2 for a discussion on the origins of diastereoselectivity). Notably, there was no erosion of enantiomeric excess when using an enantiopure diol (**20**), and further heterocycles such as a tetrahydrofuran (**24**) and pyrrolidine ring (**25**) could be incorporated. The fused dioxane-pyrrolidine motif is present in a number of bioactive compounds (e.g., **C**, Figure 1A).^5,24^

The protocol was extended to the synthesis of other ring systems (Scheme 1D). 1,2-Ethanedithiol and 2-mercaptoethanol could be used as bis-nucleophiles after slight adaptations of the reaction conditions to minimize overreactivity (**26** and **27**, Supporting Information Tables S3 and S4). Catechol led to a mixture of 1,4-dioxane **28**, dihydrobenzofuran, and diaryloxetane (Supporting Information Table S5). Glycolic acid was a successful coupling partner and yielded dioxanone **29** in 69% yield under the standard conditions.

Several 1,4-dioxanes were further characterized by X-ray crystallography (**2**, **16**, **18**, **21**, **21′**, and **23**; Scheme 1). The crystal structures revealed a preference of the CH_2_OH group for the equatorial position, leaving the aromatic substituent axial. The crystal structures also confirmed the relative stereochemistry of the major diastereomeric products in Scheme 1C, which was also independently assigned by NOE spectroscopy. Interestingly, the relative configuration of minor diastereomer **21′**, which was isolated and separated from **21** by column chromatography, was also confirmed by X-ray crystallography.

Further derivatization of the 1,4-dioxane products demonstrated their stability and potential as functionalizable building blocks (Scheme 2). Alcohol **2** was oxidized with potassium permanganate to carboxylic acid **30**. Alkylation of the alcohol installed an alkyne click handle (**31**), and a nucleophilic aromatic substitution reaction introduced a medicinally important pyridine ring (**32**). Selective triflation of phenol **8** in the presence of the aliphatic alcohol allowed a Suzuki cross-coupling reaction to expand the range of functionality on the aromatic ring (Scheme 2B).

**Scheme 2:**
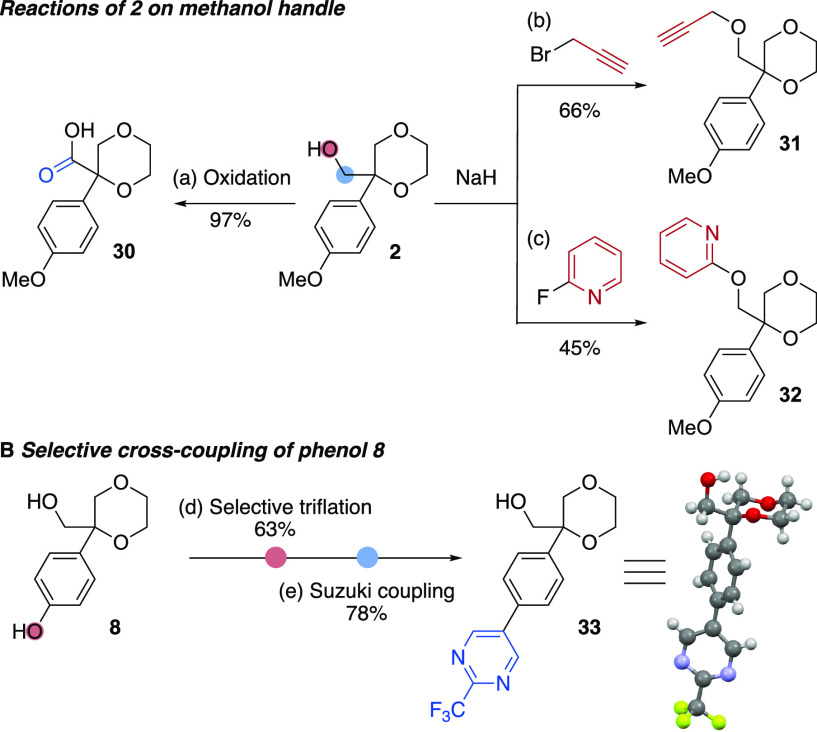
Derivatizations of 1,4-Dioxane Products Reactions on a 0.2 mmol scale. Isolated yields are reported. Conditions: (a) aq. KMnO_4_, 1 M aq. NaOH, 0–25 °C, 4 days. (b) Propargyl bromide (2.0 equiv), NaH (5.0 equiv), DMF, 0–25 °C, 19 h. (c) 2-Fluoropyridine (1.33 equiv), NaH (1.2 equiv), DMF, 0–90 °C, 24 h. (d) NTf_2_Ph (1.5 equiv), NEt_3_ (3.0 equiv), DMAP (10 mol %), CH_2_Cl_2_, 0–25 °C, 4 h. (e) Ar–Bpin (1.5 equiv), Pd(OAc)_2_ (5 mol %), SPhos (10 mol %), K_3_PO_4_ (2.0 equiv), dioxane/H_2_O (4:1), 65 °C, 44 h.

Mechanistically, two possibilities may be considered, where the order of key steps of hydroxyl substitution and oxetane ring opening are reversed (see the Supporting Information, page S19 for further discussion). Based on our observations and prior studies,^15,17^ we propose a catalytic cycle whereby the oxetanol first selectively reacts at the hydroxyl group, promoted by the Brønsted acid catalyst, to generate an oxetane carbocation (**I** and **II**; Scheme 3). Trapping of the carbocation by ethylene glycol leads to an oxetane ether intermediate (**III**), which is typically not observed^25^ and rapidly opens the protonated oxetane ring to form a 1,4-dioxane and regenerate the catalyst upon a final deprotonation (**IV**).

**Scheme 3:**
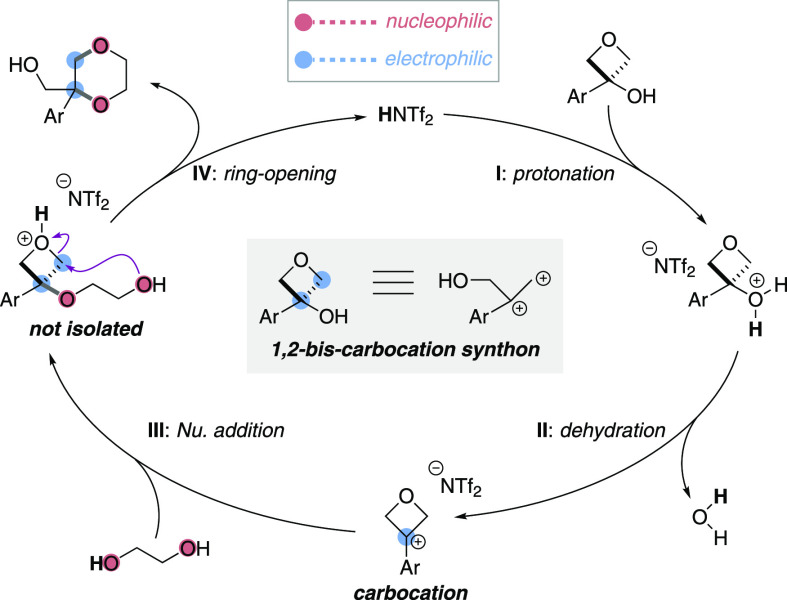
Mechanistic Hypothesis

Overall, oxetanols can act as 1,2-bis-carbocation synthons in the reaction with diols in an unusual annulation reaction to form dioxanes. 1,4-Dioxanes are formed in high yield from readily available oxetan-3-ols and 1,2-diols using Brønsted acid catalysis. A wide range of mono- and bicyclic dioxanes were generated in good yields and high regio- and diastereoselectivities, including fused ring and spirocyclic examples. The methodology was extended toward the synthesis of other heterocycles such as dioxanones and 1,4-dithianes. The products were diversified at the methanol handle through oxidation and alkylation reactions. This work further demonstrates the value of oxetanes as unusual synthons that allow for nonclassical retrosynthetic disconnections, providing a useful tool for the construction of complex molecules.

## Supporting Information Available

Experimental procedures, characterization data, and copies of ^1^H and ^13^C NMR spectra; detailed optimization tables; NMR studies on product stereochemistry; rationale for observed diastereoselectivity; and further mechanistic discussion (PDF)

## Accession Codes

CCDC 2144680–2144687 contain the supplementary crystallographic data for this paper. These data can be obtained free of charge via www.ccdc.cam.ac.uk/data_request/cif, or by emailing data_request@ccdc.cam.ac.uk, or by contacting The Cambridge Crystallographic Data Centre, 12 Union Road, Cambridge CB2 1EZ, UK; fax: +44 1223 336033.

## Notes

All characterization data for synthesized compounds can be found at 10.14469/hpc/10095.
