# Supplementary material for: Oxetan-3-ols as 1,2-bis-Electrophiles in a Brønsted-Acid-Catalyzed Synthesis of 1,4-Dioxanes
Source: Org Lett. 2022 Mar 21;24(12):2365–70. doi: 10.1021/acs.orglett.2c00568 (PMC9007565; doi:10.1021/acs.orglett.2c00568)
Supplement: Supplementary file 1 — ol2c00568_si_001.pdf [file ol2c00568_si_001.pdf]

SUPPORTING INFORMATION

Oxetan-3-ols as 1,2-bis-Electrophiles in a Brønsted Acid Catalyzed Synthesis of 1,4-Dioxanes

Juan J. Rojas,[a] Elena Torrisi,[a,b] Maryne, A. J. Dubois,[a] Riashat Hossain[a] Andrew, J. P. White,[a] Giovanni Zappia,[b] James, J. Mousseau,[c] Chulho Choi,[c] and James, A. Bull*,[a]

[a] Department of Chemistry, Imperial College London, Molecular Sciences Research Hub, White City Campus, Wood Lane, London W12 0BZ, UK.
[b] Department of Biomolecular Sciences, School of Pharmacy, University of Urbino "Carlo Bo", P.za Rinascimento, 6, 61029 URBINO (PU), Italy.
[c] Pfizer Global Research and Development, 445 Eastern Point Rd., Groton, CT 06340, USA.

E-mail: j.bull@imperial.ac.uk

Full List of References in Reference 5 ..... S3
General Experimental Conditions S4
Structures of Oxetan-3-ols S6
Structures of Additional Compounds in SI S7
Unsuccessful Oxetan-3-ols and Bis-nucleophiles S8
Synthesis of 1,4-Dioxanes S9–S18
Optimization Reactions S9
Optimization and Origin of Diastereoselectivity S11
General Procedure A & B S13
Optimization with 1,2-Ethanedithiol and 2-Mercaptoethanol S15
Catechol as Bis-nucleophile S18
Mechanistic Considerations S19–S21
Isolation and Investigation of an Oxetane Thioether Intermediate S20
Experimental Details and Characterization Data S22–S56
3-Aryl-Oxetan-3-ols (**1a**–**m**) S22
1,4-Dioxanes (**2**–**25**) S25
Other Heterocycles (**26**–**29**, **S1**–**S10**) S49
Derivatization of 1,4-Dioxane products (**30**–**33**) S54
HPLC Traces of Enantioenriched 1,4-Dioxane **20** S57
X-Ray Crystallography Details S58–S68
1,4-Dioxanes **2**, **16**, **18**, **21**, **21'**, **23**, **33** S58
Oxetane Thioether **S8** S67
^{1}H, ^{13}C and ^{19}F NMR Spectra of Selected Compounds S69–S132
References S133

Full List of References in Reference 5

(a) Labéguère, F.; Dupont, S.; Alvey, L.; Soulas, F.; Newsome, G.; Tirera, A.; Quenehen, V.; Mai, T. T. T.; Deprez, P.; Blanqué, R.; Oste, L.; Le Tallec, S.; De Vos, S.; Hagers, A.; Vandevelde, A.; Nelles, L.; Vandervoort, N.; Conrath, K.; Christophe, T.; van der Aar, E.; Wakselman, E.; Merciris, D.; Cottereaux, C.; da Costa, C.; Saniere, L.; Clement-Lacroix, P.; Jenkins, L.; Milligan, G.; Fletcher, S.; Brys, R.; Gosmini, R. Discovery of 9-Cyclopropylethynyl-2-((*S*)-1-[1,4]Dioxan-2-Ylmethoxy)-6,7-Dihydropyrimido[6,1-a]Isoquinolin-4-One (GLPG1205), a Unique GPR84 Negative Allosteric Modulator Undergoing Evaluation in a Phase II Clinical Trial. *J. Med. Chem.* **2020**, *63*, 13526–13545. (b) Del Bello, F.; Bonifazi, A.; Giorgioni, G.; Piergentili, A.; Sabbieti, M. G.; Agas, D.; Dell'Aera, M.; Matucci, R.; Górecki, M.; Pescitelli, G.; Vistoli, G.; Quaglia, W. Novel Potent Muscarinic Receptor Antagonists: Investigation on the Nature of Lipophilic Substituents in the 5- and/or 6-Positions of the 1,4-Dioxane Nucleus. *J. Med. Chem.* **2020**, *63*, 5763–5782. (c) Masse, C. E.; Greenwood, J. R.; Romero, D. L.; Harriman, G. C.; Wester, R. T.; Shelley, M.; Kennedy-Smith, J. J.; Dahlgren, M.; Mondal, S. *TYK2 Inhibitors and Uses Thereof*, WO2017040757A1, 2017.

General Experimental Considerations

All non-aqueous reactions were carried out under an inert atmosphere (argon) with flame-dried glassware, using standard techniques, unless specified. Anhydrous solvents were obtained by filtration through drying columns (toluene, $CHCl_3$, CH_2Cl_2, DMF) or used as supplied (MeCN, DCE).

Reactions that required thermal activation were heated using a water bath (for temperatures up to 25 °C) or a silicone oil bath (for temperatures >25 °C).

Flash column chromatography was performed using 230–400 mesh silica with the indicated solvent system according to standard techniques. Analytical thin-layer chromatography (TLC) was performed on precoated glass-backed silica gel plates. Visualization of the developed chromatogram was performed by UV absorbance (254 nm) and stained with aqueous potassium permanganate solution, phosphomolybdic acid solution, *para*-anisaldehyde solution or ninhydrin solution in ethanol.

Infrared spectra (ν_{max}, FTIR ATR) were obtained using an Agilent Technologies Cary 630 FTIR or a Perkin Elmer Spectrum 100 FTIR Spectrometer and recorded in reciprocal centimeters (cm^{-1}) (br = broad, w = weak, st = stretch, as = asymmetric, sy = symmetric). Only significantly strong and clearly assignable signals diagnostic for major functional groups are reported.

Nuclear magnetic resonance spectra were recorded on 400 or 500 MHz spectrometers. The frequency used to record the NMR spectra is given in each assignment and spectrum (^1H NMR at 400 or 500 MHz; ^{13}C NMR at 101 MHz or 126 MHz; ^{19}F at 377 MHz). Chemical shifts for ^1H NMR spectra are recorded in parts per million (ppm) from tetramethylsilane with the residual protic solvent resonance as the internal standard ($CHCl_3$: δ = 7.27 ppm, DMSO: δ = 2.50 ppm, CH_3OD: δ = 3.31 ppm). Data are reported as follows: chemical shift (multiplicity [s = singlet, d = doublet, t = triplet, q = quartet, hept = heptet, m = multiplet and br = broad], coupling constant (in Hz), integration of equivalent nuclei and assignment). ^{13}C NMR spectra were recorded with complete proton decoupling. Chemical shifts are reported in parts per million (ppm) from tetramethylsilane with the solvent resonance as the internal standard ($^{13}CDCl_3$: δ = 77.0 ppm, $(^{13}CD_3)_2SO$: δ = 39.5 ppm, $^{13}CD_3OD$: δ = 49.0 ppm). *J* values are reported in Hz. ^{19}F NMR spectra are indirectly referenced to $CFCl_3$ automatically by direct measurement of the absolute frequency of the deuterium lock signal by the spectrometer hardware.

Assignments of ^1H and ^{13}C spectra were based upon the analysis of δ and *J* values, by analogy of previous examples, as well as NOESY, COSY, HSQC and HMBC experiments where appropriate. Selected 2D spectra are included in this document (Supporting Figures S3–S7, S9–S16, S18–S19) and in the section with NMR spectra (page S69–S132). All raw and processed 2D spectra can be found at https://doi.org/10.14469/hpc/10095.

For clarity NMR spectra are displayed as follows unless this would obscure signals: ^1H NMR spectra are displayed between 10.0 ppm and 0.0 ppm; ^{13}C NMR spectra are displayed between 210 ppm and 0 ppm. ^{19}F spectra are displayed for the full sweep width as acquired.

Melting points were recorded using an Optimelt MPA100 apparatus and are uncorrected.

High resolution mass spectrometry (HRMS) analyses were performed through the Imperial College or EPSRC mass spectrometry service. HRMS analyses at Imperial College were performed using an electrospray ion source (ESI), nanospray ionization (NSI), electron impact ionization (EI) or atmospheric pressure chemical ionization (APCI) using an atmospheric solids analysis probe (ASAP). ESI was performed using a Waters LCT Premier (ES-ToF) equipped with an ESI source operated in positive or negative ion mode. APCI was performed using a Thermo Scientific Q-Extractive/Dionex Ultimate 3000 using an ASAP to insert samples into the APCI source operated in positive or negative mode. The sample was introduced at ambient temperature and the temperature increased until the sample vaporized. HRMS analyses at the EPSRC UK National Mass Spectrometry Facility (NMSF) were performed using a nano-electrospray ion source (NSI), chemical ionization (CI) or atmospheric pressure chemical ionization (APCI) using an atmospheric solids

analysis probe (ASAP). NSI was performed using a Thermo Scientific LTQ Orbitrap XL operated in positive or negative ion mode. CI was performed using a Finnigan MAT 95 XP operated in positive mode. APCI was performed using a Waters Xevo G2-S using an ASAP to insert samples into the APCI source operated in positive mode. The sample was introduced at ambient temperature and the temperature increased until the sample vaporized. The software used was either MassLynx 4.1 or Bruker Daltonics DataAnalysis 4.0. Please note: MassLynx 4.1 software, used at the Imperial College mass spectrometry service, does not account for the electron and all the calibrations/references are calculated accordingly, i.e. $[M+H]^+$ is detected and the mass is calibrated to output [M+H]. In the cases where this software is used, we report the HRMS as [M+H].

Observed optical rotations (α') were measured at the indicated temperature (T °C) and converted to the corresponding specific rotations $[\alpha]_D^T$ in deg cm^2 g^{-1}, concentration (*c*) in g per 100 mL. HPLC analyses were carried out on an Agilent 1260 Infinity Series system, employing Daicel Chiracel columns.

Reagents: Where the synthesis of a reagent is not stated, the reagent was commercially available. Commercial reagents were used as supplied or purified by standard techniques where necessary.

- Trifluoromethanesulfonimide (Tf_2NH) was purchased from Fluorochem (CAS: 82113-65-3, product code: 093934), stored under argon in the fridge (+4 °C) and used without further purification.
- The exact concentration of *n*-BuLi (1.6 M in hexanes, purchased from Sigma-Aldrich, CAS: 109-72-8) was determined by titration with salicylaldehyde phenylhydrazone as indicator before each reaction using a literature procedure.[1] The average of three titrations was taken.
- (*S*,*S*)-cyclohexane-1,2-diol (purchased from Sigma-Aldrich, CAS: 57794-08-8) was purified before use by filtration through a plug of silica.

Structures of Oxetan-3-ols

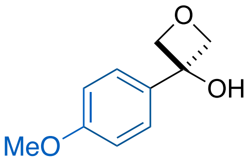

1a

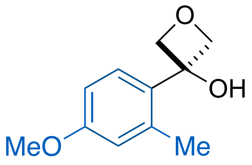

1b

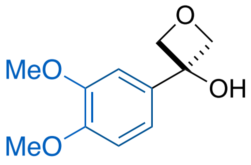

1c

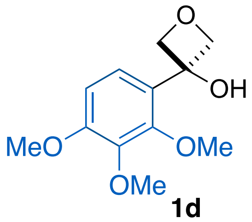

1d

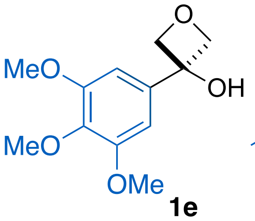

1e

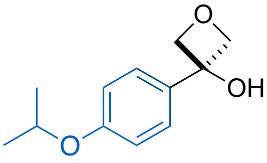

1f

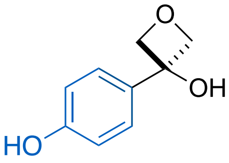

1g

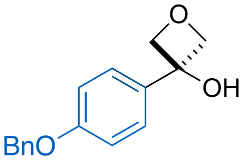

1h

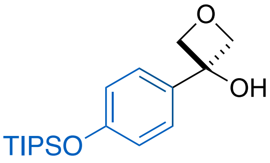

1i

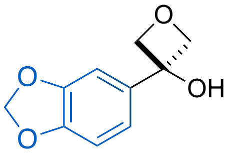

1j

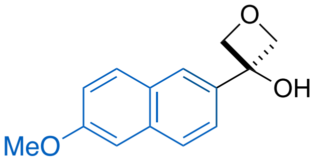

1k

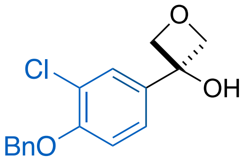

1l

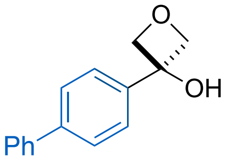

1m

Structures of Additional Compounds in SI

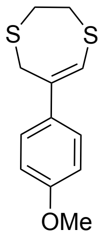

S1

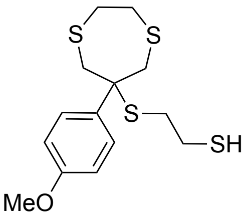

S2

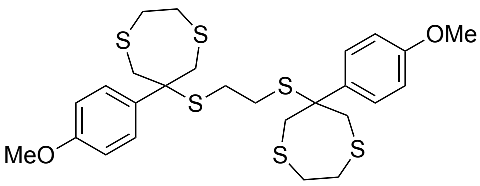

S3

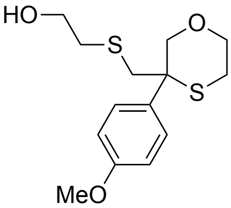

S4

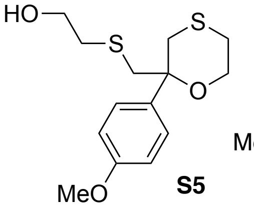

S5

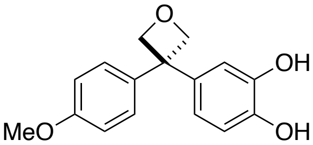

S6

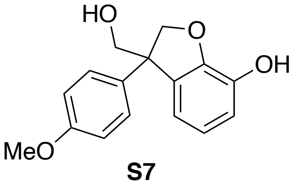

S7

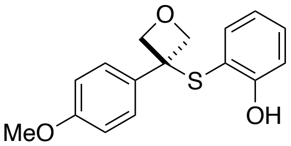

S8

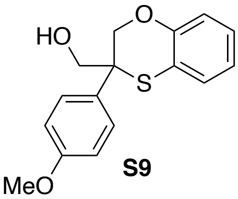

S9

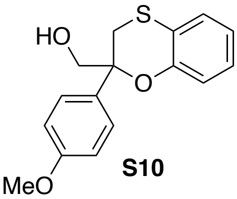

S10

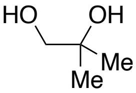

S11

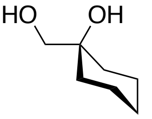

S12

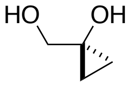

S13

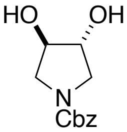

S14

Unsuccessful Oxetan-3-ols and Bis-nucleophiles

Scheme S1 shows additional 3-aryl-oxetan-3-ols and bis-nucleophiles where the reaction did not yield the dioxane product. Non electron-rich oxetanols, which do not provide sufficient stabilization for the oxetane carbocation (e.g. *p*-tolyl), mono-substituted 1,2-bis-nucleophiles, which lead to a mixture of regio and diastereomers, and insoluble diols (in MeCN) are limitations of this methodology.

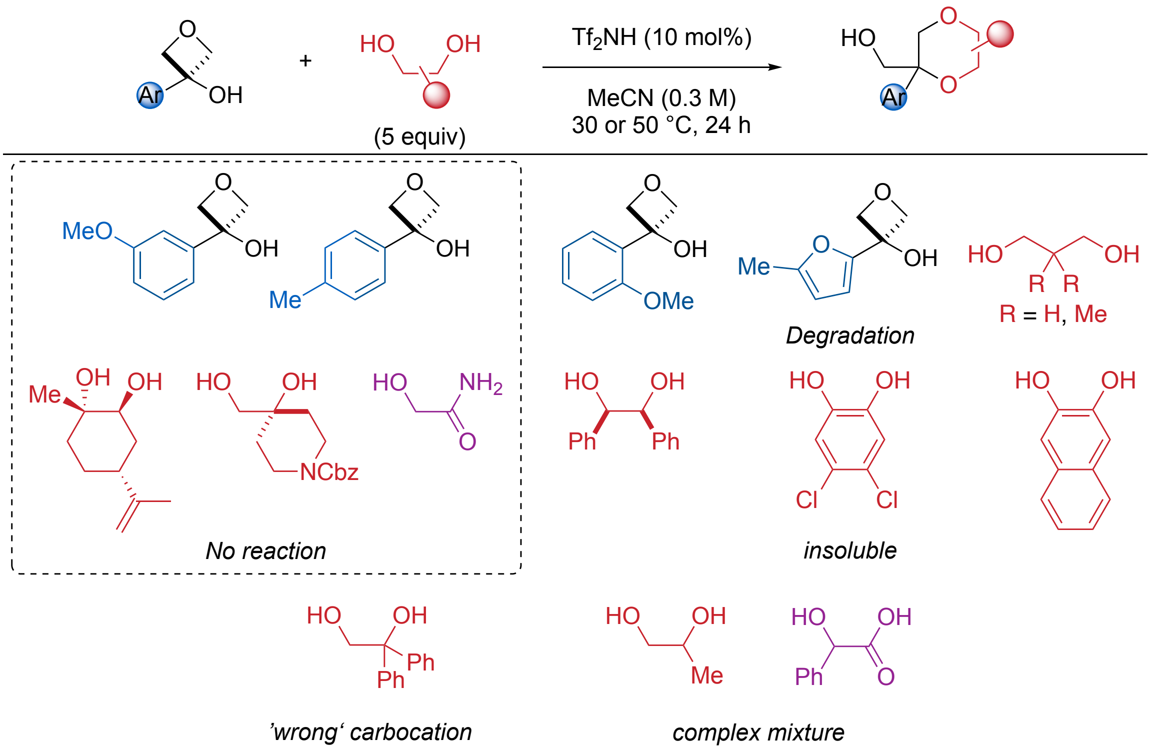

Scheme S1 Unsuccessful oxetan-3-ols and bis-nucleophiles.

Synthesis of 1,4-Dioxanes

Optimization Reactions

Table S1 shows all optimization reactions performed (including those depicted in Table 1). Lewis acids previously shown to activate oxetanol **1a** were unsuccessful (entries 1–2).[2,3,4,5] A change to a Brønsted acid catalyst (TfOH) showed moderate amount of 1,4-dioxane **2** and MeCN was chosen as the optimal solvent (entries 3–8). Reaction time was increased to 24 h and the catalyst changed from TfOH (a fuming liquid) to Tf_2NH (a solid) to improve practicality (entries 9–10). A screening of different combinations of temperature and concentration revealed 50 °C and 0.3 M to be optimal (entries 10–18). The equivalents of ethylene glycol could be lowered to 3 or even 1 equivalents without significant reductions in yield (entries 19–20) and ethylene glycol could be used as limiting reagent with a slight excess of oxetanol to obtain **2** in a very high yield (entry 21). Reaction in the absence of nucleophile did not show any Ritter reactivity with acetonitrile and 54% of oxetanol **1a** were recovered (entry 22).

Table S1 Optimization reactions for the synthesis of 1,4-dioxane **2** from oxetanol **1a** and ethylene glycol. Reactions were carried out on a 0.25 mmol scale using **General Conditions A** with any change noted.

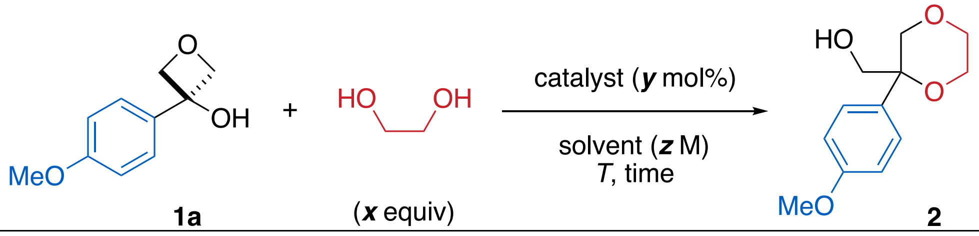

Entry	cat. (*y*)	*x*	Solvent (*z*)	*T* / °C	Time / h	Yield / %[a] 1a (RSM)	Yield / %[a] 2
1	Li(NTf$_2$) (11)[b]	5	CHCl$_3$ (0.5)	40	24	ND	0
2	Ca(NTf$_2$)$_2$ (5)[c]	5	CHCl$_3$ (0.5)	40	24	ND	0
3	TfOH (5)	5	CHCl$_3$ (0.5)	40	16	39	42
4	TfOH (5)	5	CH$_2$Cl$_2$ (0.5)	40	16	31	55
5[d]	TfOH (5)	5	DCE (0.5)	40	16	54	27
6	TfOH (5)	5	toluene (0.5)	40	16	12	68
7	TfOH (10)	5	toluene (0.5)	40	16	10	73
8	TfOH (10)	5	MeCN (0.5)	40	16	0	84
9	TfOH (10)	5	MeCN (0.5)	40	24	7	88
10	Tf$_2$NH (10)	5	MeCN (0.5)	40	24	0	86
11	Tf$_2$NH (10)	5	MeCN (0.3)	40	24	0	91
12	Tf$_2$NH (10)	5	MeCN (0.7)	40	24	0	86
13	Tf$_2$NH (10)	5	MeCN (0.5)	50	24	0	84
14	Tf$_2$NH (10)	5	MeCN (0.3)	50	24	0	95 (91)[e]
15	Tf$_2$NH (10)	5	MeCN (0.7)	50	24	0	87
16	Tf$_2$NH (10)	5	MeCN (0.5)	60	24	0	88
17	Tf$_2$NH (10)	5	MeCN (0.3)	60	24	0	93
18	Tf$_2$NH (10)	5	MeCN (0.7)	60	24	0	93
19	Tf$_2$NH (10)	3	MeCN (0.3)	50	24	0	90
20	Tf$_2$NH (10)	1	MeCN (0.3)	50	24	0	80
21[f]	Tf$_2$NH (10)	0.75	MeCN (0.3)	50	24	1	96
22	Tf$_2$NH (10)	0	MeCN (0.3)	50	24	54	NA

[a] Yields calculated by analysis of the ^{1}H NMR spectrum of the crude mixture of the reaction using 1,3,5-trimethoxybenzene as internal standard. Isolated yields in parentheses. [b] With 5.5 mol% of Bu$_4$NPF$_6$ as additive. [c] With 5.0 mol% of Bu$_4$NPF$_6$ as additive. [d] 0.5 mmol scale. [e] Isolated on a 0.91 mmol scale in a separate reaction. [f] Yield based on ethylene glycol, RSM based on oxetanol **1a**. RSM = Returned starting material. ND = Not determined. DCE = 1,2-dichloroethane. NA = Not applicable.

Optimization and Origin of Diastereoselectivity

The reaction was optimized for diastereoselectivity using *trans*-1,2-cyclohexanediol (Table S2). Decreasing the reaction temperature increased diastereoselectivity, but too low temperatures were detrimental for reactivity. 30 °C was found to be optimal to maintain a high reactivity whilst achieving good diastereoselectivities and all unsymmetrical 1,2-diols were run at this temperature.

Table S2 Optimization of diastereoselectivity of 1,4-dioxane **20** from oxetanol **1a** and *trans*-1,2-cyclohexanediol. Reactions were carried out on a 0.25 mmol scale using **General Conditions A** with any change noted.

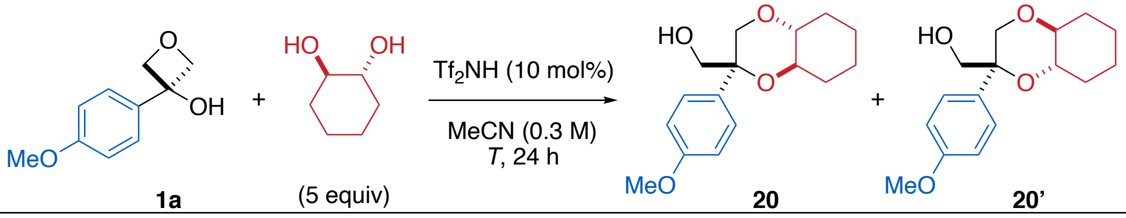

Entry	*T* / °C	dr (20:20')[a]	Yield / %[b]		
			**1a (RSM)**	**20**	**20 + 20'**
1	50	77:23	0	75	98
2	40	88:12	0	86	92
3	30	93:7	0	89 (78[c])	96
4	22	93:7	7	85	93
5	0	98:2	32	58	61

[a] Diastereomeric ratios (dr) determined from the ^{1}H NMR spectrum of the crude reaction mixture. [b] Yields calculated by analysis of the ^{1}H NMR spectrum of the crude mixture of the reaction using 1,3,5-trimethoxybenzene as internal standard. Isolated yields in parentheses. [c] Additional 7% were isolated as a mixture of diastereomers (dr 52:48). RSM = Returned starting material.

Diastereoselectivity is proposed to arise from the preference of an unstrained chair-like transition state (**TS1**) that leads to the major 1,4-dioxane diastereomer (Scheme S2). The transition state to obtain the minor diastereomer would either involve a severely strained oxetane ring (**TS2**) or a twist-boat conformation (**TS3**). 1,3-Diaxial interactions in **TS1** between the aromatic and an axial proton reduce the difference in transition state barrier heights ($\Delta\Delta G^{\ddagger}$) between both pathways. The approach of the nucleophile to breaking the C–O bond of oxetane, the S_N2-like transition state geometry of the carbon being attacked (trigonal bipyramidal) and the proposed chair-like transition state are all in accordance with the computational models described by Houk and Wheeler for the enantioselective phosphoric acid-catalyzed synthesis of 1,4-dioxanes from oxetane hydroxy ether precursors ("Notably, the chairlike conformation of the forming six-membered ring is always the most favorable").[6] An analogous analysis with 1,2-*cis*-diols leads to the same outcome.

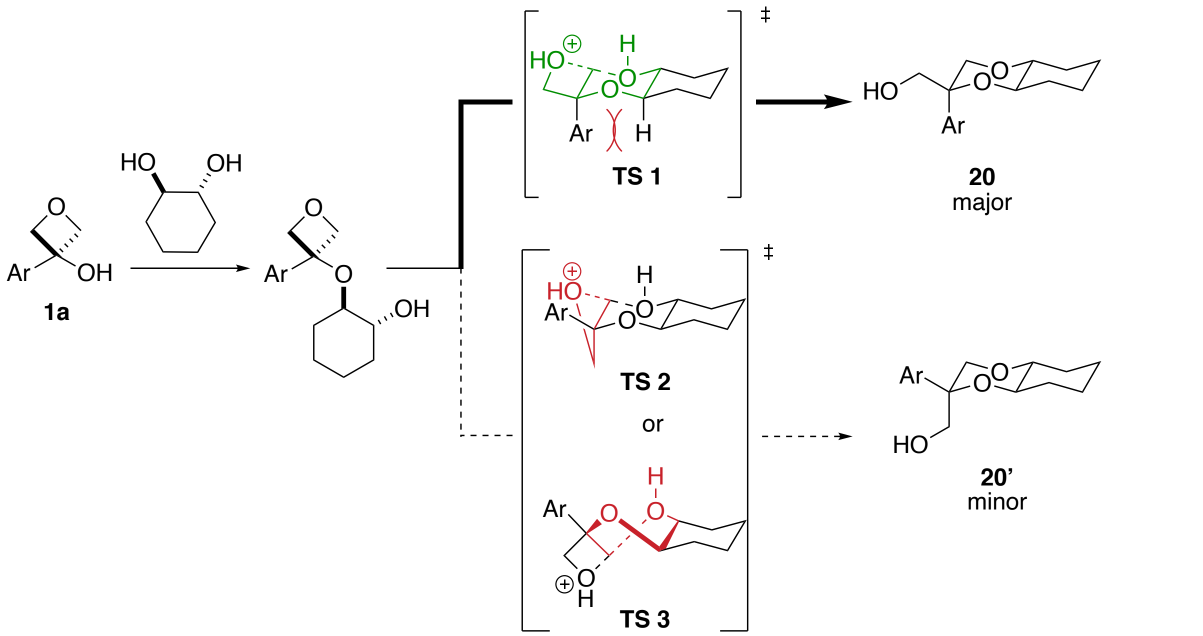

Scheme S2 Proposed origin of diastereoselectivity favoring an unstrained oxetane chair-like transition state (**TS1**).

General Procedure A

Oxetanol **1a–m** (0.25 mmol, 1.0 equiv) was added to a solution of trifluoromethane sulfonimide (7.0 mg, 0.025 mmol, 0.1 equiv) and bis-nucleophile (1.25 mmol, 5.0 equiv) in anhydrous MeCN (0.83 mL, 0.3 M) in a flame-dried reaction tube under Ar. The reaction mixture was stirred at 50 °C for 24 h and then quenched with sat. aq. $NaHCO_3$ (10 mL). The aqueous layer was extracted with CH_2Cl_2 (3 × 10 mL). The organic layers were combined, dried over Na_2SO_4, filtered and concentrated *in vacuo* using a rotatory evaporator. Purification by flash column chromatography afforded dioxane **2–14**.

General Procedure B

Reactions with unsymmetrical diols

Oxetanol **1a** (45.1 mg, 0.25 mmol, 1.0 equiv) was added to a solution of trifluoromethane sulfonimide (7.0 mg, 0.025 mmol, 0.1 equiv) and bis-nucleophile (1.25 mmol, 5.0 equiv) in anhydrous MeCN (0.83 mL, 0.3 M) in a flame-dried reaction tube under Ar. The reaction mixture was stirred at 30 °C for 24 h and then quenched with sat. aq. $NaHCO_3$ (10 mL). The aqueous layer was extracted with CH_2Cl_2 (3 × 10 mL). The organic layers were combined, dried over Na_2SO_4, filtered and concentrated *in vacuo* using a rotatory evaporator. Purification by flash column chromatography afforded dioxane **15–25**.

Further remarks:

The Brønsted acid catalyst ($HNTf_2$, trifluoromethane sulfonimide) is a hygroscopic and deliquescent solid and was thus, weighed out directly into the reaction vial under Ar, followed by MeCN and bis-nucleophile. $HNTf_2$ will quickly deliquesce with minimal amounts of moisture in the atmosphere and may 'liquify' on the walls of the reaction vessel, which was not found to be detrimental for the reaction.

In general, the tertiary solvent system Et_2O/CH_2Cl_2/pentane was found beneficial to separate diastereomeric products by flash column chromatography.

Racemic mixtures of diastereomeric 1,4-dioxane products were distinguished with the empiric denominators *cis* and *trans*, always referring to the out-of-plane substituent with the highest rank according to IUPAC and starting with the relative configuration of the 1,2-diol (see Fig. S1 for an example).

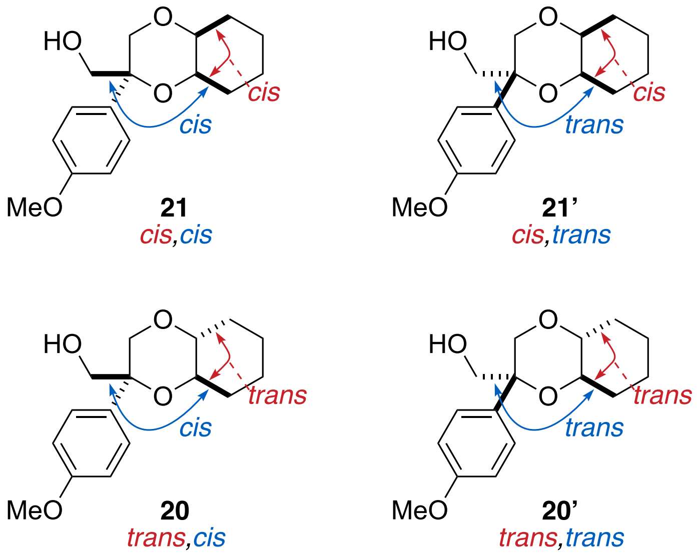

Fig. S1 Naming of racemic 1,4-dioxane diastereomeric products.

$HNTf_2$ fumes lightly under a humid atmosphere and should be handled in a fume hood at all times. Excess acid on spatula was quenched with a sat. aq. $NaHCO_3$ solution.

When using thiol-containing bis-nucleophiles (toxic, smelly and volatile) use a rotatory evaporator in a ventilated space during workup. Quench excess thiol in the syringe with a dilute bleach bath (bleach:H_2O = 1:1) and leave the syringe to stand in the bleach bath for 24 h before disposal.

Optimization with 1,2-Ethanedithiol and 2-Mercaptoethanol

Thiol-containing bis-nucleophiles were found to be too reactive under the standard reaction conditions and led to complex mixtures of ring-opened products (Scheme S3 and Table S3, entry 1), as had been previously observed with simple thiol (mono) nucleophiles[3]. These side products are believed to be formed through ring opening of the oxetane *before* the desired S_N1 condensation reaction could occur. Ring-opened intermediate **A** can then reclose the ring by substitution of the less hindered primary alcohol by the remaining free thiol to form 1,4-dithiepane **B** and form an alternative carbocation **C** which can undergo further reactions to generate some of the isolated side products **S1**–**S3** (Scheme S3).

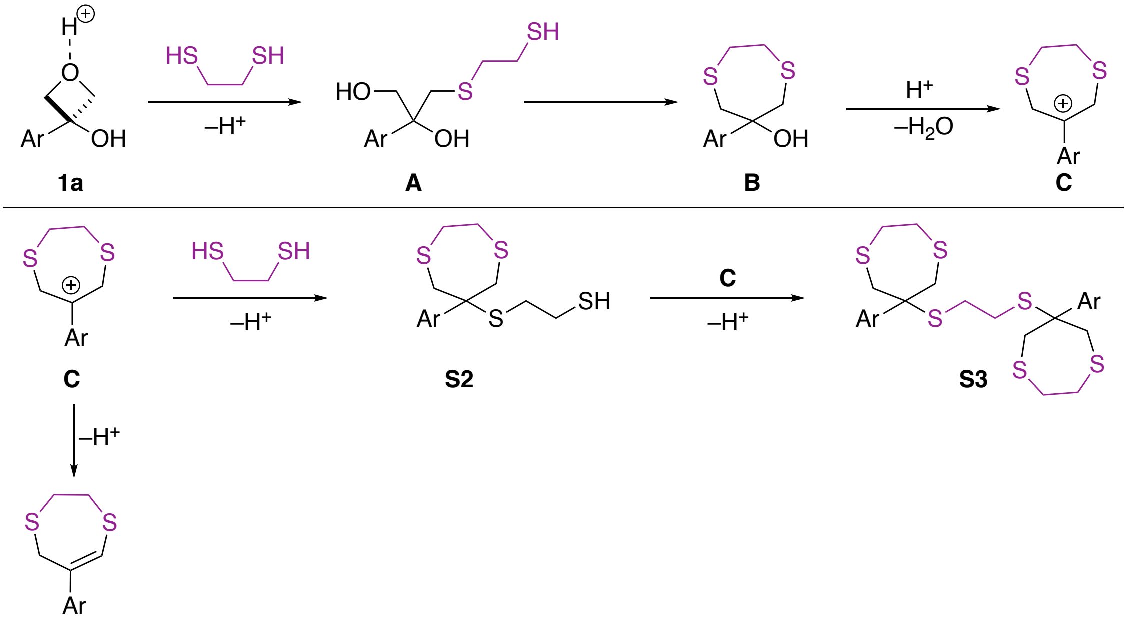

Scheme S3 Proposed mechanism for the formation of side products **S1**–**S3**.

The typical Lithium catalyzed conditions[2] still showed no reactivity, presumably due to deactivation of the metal catalyst caused by chelation of the bis-nucleophile (Table S3, entry 2). Reducing the equivalents of bis-nucleophile (entry 3) and the temperature (entry 4) began to show productive reactivity, albeit still alongside significant amounts of side products. A screening of different combinations of temperature and equivalents of 1,2-ethanedithiol showed 30 °C and 1.2 equivalents to be optimal (entries 5–8). The protocol was also modified to add oxetanol **1a** to the cold (0 °C) reaction mixture followed by slow warming to the indicated temperature to prevent side reactions caused by temporary super-excesses of dithiol.

An analogous approach was performed to optimize the formation of 1,4-oxathiane **27** which now has the additional challenge of chemoselectivity between S and O attack (Table S4). The optimal conditions used 2-mercaptoethanol as limiting reagent with a slight excess of oxetanol **1a** (1.3 equiv) at 23 °C to obtain 1,4-oxathiane **27** in 47% isolated yield and a good chemoselectivity.

Table S3 Optimization reactions for the synthesis of 1,4-dithiane **26** from oxetanol **1a** and 1,2-ethanedithiol. Reactions were carried out on a 0.25 mmol scale using **General Conditions A** with any change noted.

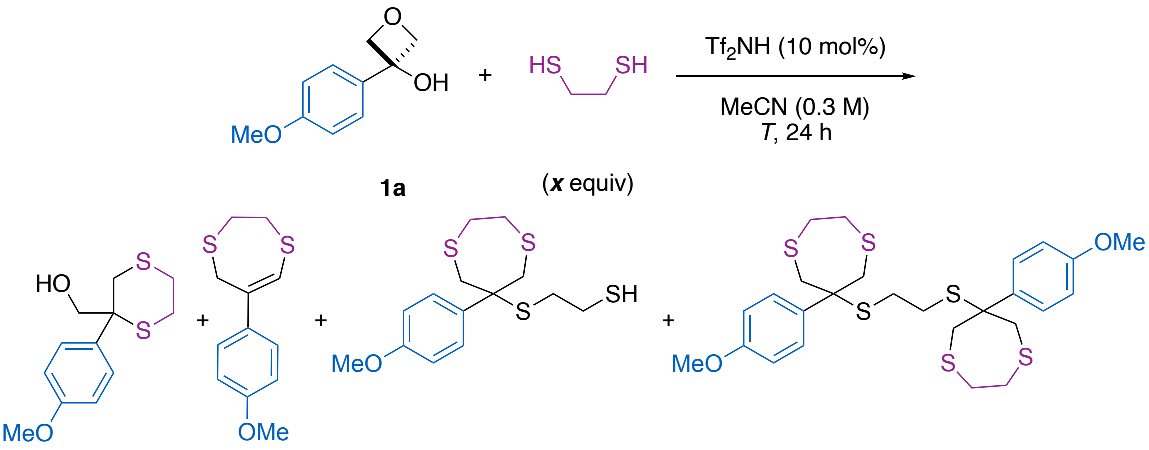

Entry	*x*	*T* / °C	Yield / %[a]				
			1a (RSM)	26	S1	S2	S3
1	5	50	0	*complex mixture obtained*			
2[b]	5	40	ND	0	0	0	0
3	2	50	0	(4)	(9)	(35)	(22)
4	2	30	0	(27)	(0)	(34)	(20)
5	1.2	0–30	0	52 (44)	0	22	0
6[c]	0.75	0–30	27	50	0	0	0
7	1.2	0–23	12	47	0	0	0
8[c]	0.75	0–23	26	45	0	0	0

[a] Yields calculated by analysis of the ^{1}H NMR spectrum of the crude mixture of the reaction using 1,3,5-trimethoxybenzene as internal standard. Isolated yields in parentheses. [b] Using $Li(NTf_2)$ (11 mol%) as catalyst and Bu_4NPF_6 (5.5 mol%) as activator, in $CHCl_3$ (0.5 M). [c] Yields based on 1,2-ethanedithiol, RSM based on oxetanol **1a**. ND = Not determined.

Table S4 Optimization reactions for the synthesis of 1,4-oxathiane **27** from oxetanol **1a** and 2-mercaptoethanol. Reactions were carried out on a 0.25 mmol scale using **General Conditions A** with any change noted.

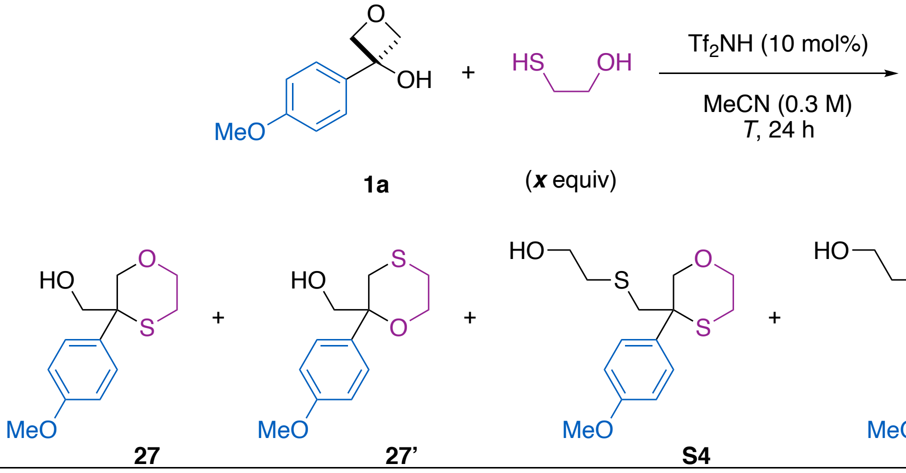

Entry	*x*	*T* / °C	Yield / %[a]				
			**1a (RSM)**	**27**	**27'**	**S4**[b]	**S5**[b]
1	5	50	0	*complex mixture obtained*			
2[c]	5	40	ND	0	0	0	0
3	2	50	0	*complex mixture obtained*			
4	2	30	0	(25)	(9)	(36)	(7)
5	1.2	0–30	0	37	20	15	13
6[d]	0.75	0–30	7	59	23	11	0
7	1.2	0–23	12	30	27	20	23
8[d]	0.75	0–23	13	60 (47)[e]	20 (15)	9	0

[a] Yields calculated by analysis of the ^{1}H NMR spectrum of the crude mixture of the reaction using 1,3,5-trimethoxybenzene as internal standard. Isolated yields in parentheses. [b] Product not characterized. Structure tentatively assigned based on the (impure) ^{1}H NMR spectrum. [c] Using $Li(NTf_2)$ (11 mol%) as catalyst and Bu_4NPF_6 (5.5 mol%) as activator, in $CHCl_3$ (0.5 M). [d] Yields based on 2-mercaptoethanol, RSM based on oxetanol **1a**. [e] Discrepancy between NMR and isolated yield was caused by a challenging separation of **27** from **S4** and **27'**. ND = Not determined.

Catechol as Bis-nucleophile

Preliminary reactions with catechol as nucleophile showed low yields and a mixture of benzodioxane **28**, diaryloxetane **S6** and dihydrobenzofuran **S7**, akin to our previously reported lithium catalyzed Friedel–Crafts reaction (Table S5).[2] Additionally, catechol was largely insoluble in the investigated solvents which led to degradation of the oxetanol starting material.

Table S5 Preliminary investigation of catechol as nucleophile. Reactions were carried out on a 0.25 mmol scale using **General Conditions A** with any change noted.

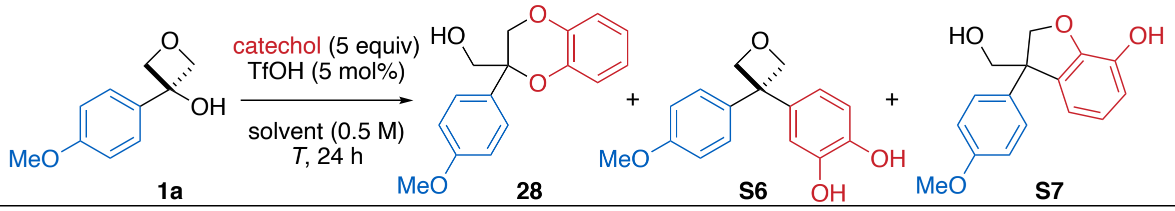

Entry	Solvent	T / °C	Yield / %[a]			
			**1a (RSM)**	**28**	**S6**[b]	**S7**[b]
1[c]	toluene	50	0	3	2	<1
2[c]	toluene	25	0	5	5	<1
3	toluene	25	0	4	4	<1
4	CH_2Cl_2	25	0	10	17	3
5[d]	$CHCl_3$	25	0	16 (23)	26[b]	4[b]

[a] Yields calculated by analysis of the ^{1}H NMR spectrum of the crude mixture of the reaction using 1,3,5-trimethoxybenzene as internal standard. Isolated yields in parentheses. [b] Products not characterized. Structures tentatively assigned based on the (impure) ^{1}H NMR spectrum of a 1:1 mixture of **S6**/**S7**. [c] Concentration 0.75 M. [d] On a 0.45 mmol scale. RSM = Recovered starting material.

Mechanistic Considerations

Two viable mechanistic pathways were considered for this reaction involving either:

A) substitution at the hydroxyl group, followed by intramolecular oxetane ring opening (as the preferred pathway) or:
B) the alternative whereby the oxetane is ring opened first and undergoes subsequent intramolecular displacement of the hydroxyl group.

There are several aspects that point to the first mechanism scenario, as is presented in Scheme 3 in the manuscript.
In short, an alternative order of events with oxetane ring-opening followed by OH-displacement (**IV** to **II** to **III**, see scheme 3) is very unlikely due to i) the requirement for electron-rich aromatics on oxetane, ii) isolation and reactivity of oxetane thioether **S8** (see below) c) literature precedent on related transformations (references 15, 17a and 23 in the manuscript) and d) the stability of 3,3-disubstituted oxetane towards intermolecular nucleophilic ring-opening (references 10 and 11 in the manuscript).

- The transformation only works with electron-rich 3-aryl-oxetan-3-ols. For example *p*-tolyl-oxetanol or *m*-methoxyphenyl-oxetanol were unreactive (see Scheme S1). Had oxetane ring-opening happened first, the ring-opened intermediate would have been observed regardless of the electronics of the aromatic ring. On the other hand, the electronics of the aromatic directly affect the stability of the proposed benzylic oxetane carbocation intermediate, which is only formed when the carbocation is sufficiently stabilized through resonance with the aromatic ring. This is consistent with the experimental observation that *p*-methoxyphenyl-oxetanol reacts smoothly under the conditions, but *p*-tolyl-oxetanol is unreactive.

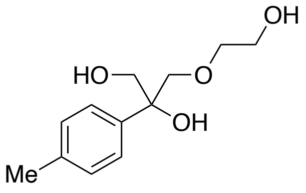

not observed.

- In the reaction with 2-mercaptophenol as nucleophile, the oxetane thioether intermediate (**S8**) was isolated and characterized by X-ray crystallography. Submitting this intermediate to the reaction conditions yielded 1,4-benzoxathiane **S9** and thereby providing evidence for the proposed (thio)-ether intermediate (see discussion below Table S6 and Scheme S4).
- Ether formation through attack of an alcohol on an oxetane carbocation is feasible, as we have observed in previous work (references 17c and 23 in the manuscript).
- In our previous related work which coupled 3-aryl-oxetan-3-ols to phenols using Lewis acids as catalysts (reference 17a in the manuscript), the Friedel–Crafts product was obtained when *ortho*-substituted phenols were used, proposedly through an oxetane carbocation intermediate. No oxetane ring-opening was observed with these substrates. On the other hand, *para*-substituted phenols yielded dihydrobenzofurans, through an analogous Friedel–Crafts pathway on the now more reactive *ortho* position, followed by intramolecular oxetane ring-opening by the phenol which is now positioned in close proximity to the oxetane. We hence proposed the related formation of dioxanes to proceed in an analogous fashion with ether formation followed by intramolecular oxetane ring-opening.
- Sun's work (reference 15 in the manuscript) demonstrates the viability for the proposed intermediate to readily cyclize to the 1,4-dioxane. These hydroxyl-ether oxetanes undergo smooth cyclization to dioxanes under Brønsted acidic conditions, even using less acidic catalysts such as phosphoric acids.

(10) (a) Wuitschik, G.; Carreira, E. M.; Wagner, B.; Fischer, H.; Parrilla, I.; Schuler, F.; Rogers-Evans, M.; Müller, K. Oxetanes in Drug Discovery: Structural and Synthetic Insights. *J. Med. Chem.* **2010**, *53*, 3227. (b) Dubois, M. A. J.; Croft, R. A.; Ding, Y.; Choi, C.; Owen, D. R.; Bull, J. A.; Mousseau, J. J. Investigating 3,3-diaryloxetanes as potential bioisosteres through matched molecular pair analysis. *RSC Med. Chem.* **2021**, *12*, 2045.
(11) (a) Bull, J. A.; Croft, R. A.; Davis, O. A.; Doran, R.; Morgan, K. F. Oxetanes: Recent Advances in Synthesis, Reactivity, and Medicinal Chemistry. *Chem. Rev.* **2016**, *116*, 12150. (b) Burkhard, J. A.; Wuitschik, G.; Rogers-Evans, M.; Müller, K.; Carreira, E. M. Oxetanes as Versatile Elements in Drug Discovery and Synthesis. *Angew. Chem. Int. Ed.* **2010**, *49*, 9052. (c) Rojas, J. J.; Bull, J. A. Oxetanes and Oxetenes-Monocyclic. In *Comprehensive Heterocyclic Chemistry IV*, Vol. 1; Black, D. StC; Cossy, J.; Stevens, C. V. Ed.; Elsevier, 2022; pp. 212.
(15) Yang, W.; Sun, J. Organocatalytic Enantioselective Synthesis of 1,4-Dioxanes and Other Oxa-Heterocycles by Oxetane Desymmetrization. *Angew. Chem. Int. Ed.* **2016**, *55*, 1868.
(17) (a) Croft, R. A.; Mousseau, J. J.; Choi, C.; Bull, J. A. Structurally Divergent Lithium Catalyzed Friedel-Crafts Reactions on Oxetan-3-Ols: Synthesis of 3,3-Diaryloxetanes and 2,3-Dihydrobenzofurans. *Chem. Eur. J.* **2016**, *22*, 16271. (b) Croft, R. A.; Dubois, M. A. J.; Boddy, A. J.; Denis, C.; Lazaridou, A.; Voisin-Chiret, A. S.; Bureau, R.; Choi, C.; Mousseau, J. J.; Bull, J. A. Catalytic Friedel-Crafts Reactions on Saturated Heterocycles and Small Rings for sp^3-sp^2 Coupling of Medicinally Relevant Fragments. *Eur. J. Org. Chem.* **2019**, *2019*, 5385. (c) Croft, R. A.; Mousseau, J. J.; Choi, C.; Bull, J. A. Oxetane Ethers Are Formed Reversibly in the Lithium-Catalyzed Friedel–Crafts Alkylation of Phenols with Oxetanols: Synthesis of Dihydrobenzofurans, Diaryloxetanes, and Oxetane Ethers. *Tetrahedron* **2018**, *74*, 5427.
(23) Rojas, J. J.; Croft, R. A.; Sterling, A. J.; Briggs, E. L.; Antermite, D.; Schmitt, D. C.; Blagojevic, L.; Haycock, P.; White, A. J. P.; Duarte, F.; Choi, C.; Mousseau, J. J.; Bull, J. A. Amino-oxetanes as amide isosteres by an alternative defluorosulfonylative coupling of sulfonyl fluorides. *Nat. Chem.* **2022**, *14,* 160.

Isolation and investigation of an Oxetane Thioether Intermediate

Preliminary reactions with 2-mercaptophenol using our previously reported lithium catalyzed conditions for the synthesis of oxetane sulfides[3] showed a strong dependence on reaction time (Table S6). Interestingly, stopping the reaction after 25 min yielded mainly oxetane thioether **S8** (entry 1), whilst leaving the reaction for 6 or 24 h gave exclusively 1,4-benzoxathiane **S9** (entries 2–3).

Table S6 Preliminary investigation of 2-mercaptophenol as nucleophile. Reactions were carried out on a 0.25 mmol scale.

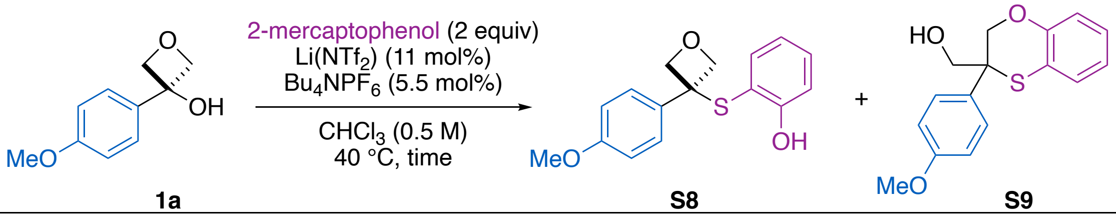

Entry	Time	Yield / %[a]		
		**1a (RSM)**	**S8**	**S9**
1	25 min	0	46	4
2	6 h	0	0	38[b]
3	24 h	0	0	35[b]

[a] Isolated yields are reported. [b] Isolated as a mixture of regioisomers. RSM = Recovered starting material.

Resubmission of thioether **S8** to the reaction conditions showed transformation into benzoxathiane **S9** alongside significant amounts of degradation products (Scheme S4 A). Resubmission of benzoxathiane **S9** to the reaction conditions showed its complete consumption and the same degradation products. (Scheme S4 B). The transformation of thioether **S8** into **S9** provides evidence for the oxetane (thio)ether intermediate proposed in Scheme 3 and speaks against a mechanism where the oxetane ring is first ring-opened and then dehydrated.

A

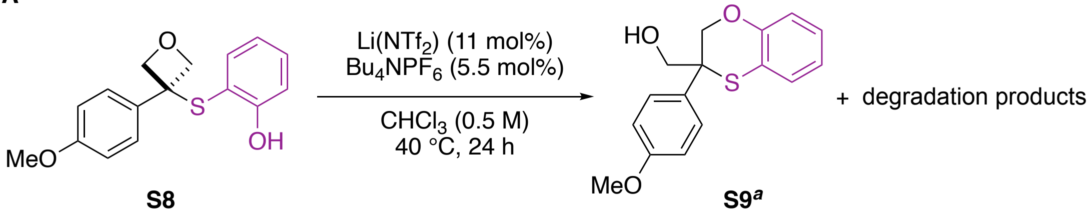

B

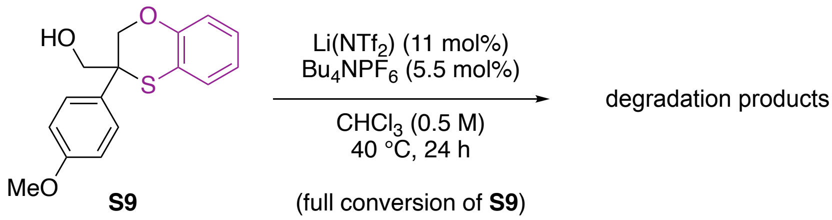

Scheme S4 Reactivity of thioether **S8** (**A**) and benzoxathiane **S9** (**B**) under the lithium catalyzed conditions. [a] As a 71:29 mixture of **S8**:**S9**.

Thioether **S8** was further characterized X-ray crystallography (Fig. S2, also see Fig. S28). The crystal structure revealed the following points worthy of note:

1. A relatively short distance between phenol and one of the methylene groups on oxetane (3.25 Å; Fig. S2 A).
2. A good alignment of phenol with one of the C–O bond coordinates on oxetane (Fig. S2 B).
3. A small angle of 54 ° to rotate phenol behind one of the C–O bonds on oxetane (Fig. S2 C). This angle is the optimal position to minimize unfavorable steric interactions. A reduction of the angle would result in a steric clash between the aromatic ring on phenol and an ortho proton on the para-methoxyphenyl ring (distance: 3.30 Å). Overcoming this unfavorable steric interaction presumably contributes significantly to the energy barrier to opening the oxetane ring.

This crystal structure suggests that thioether **S8** is geometrically set up to interact with one of the C–O σ* antibonding orbitals on oxetane and open the ring. The steric clashes caused by the aromatic ring of 2-mercaptophenol (*vide supra*) presumably led to a higher-than-usual energy barrier to oxetane ring-opening and hence allowed for the isolation of thioether intermediate **S8**. This steric clash would be present to a much lesser extent in aliphatic bis-nucleophiles, leading to a lower energy barrier to oxetane ring-opening and in line with the non-detection of any aliphatic oxetane ether intermediates.

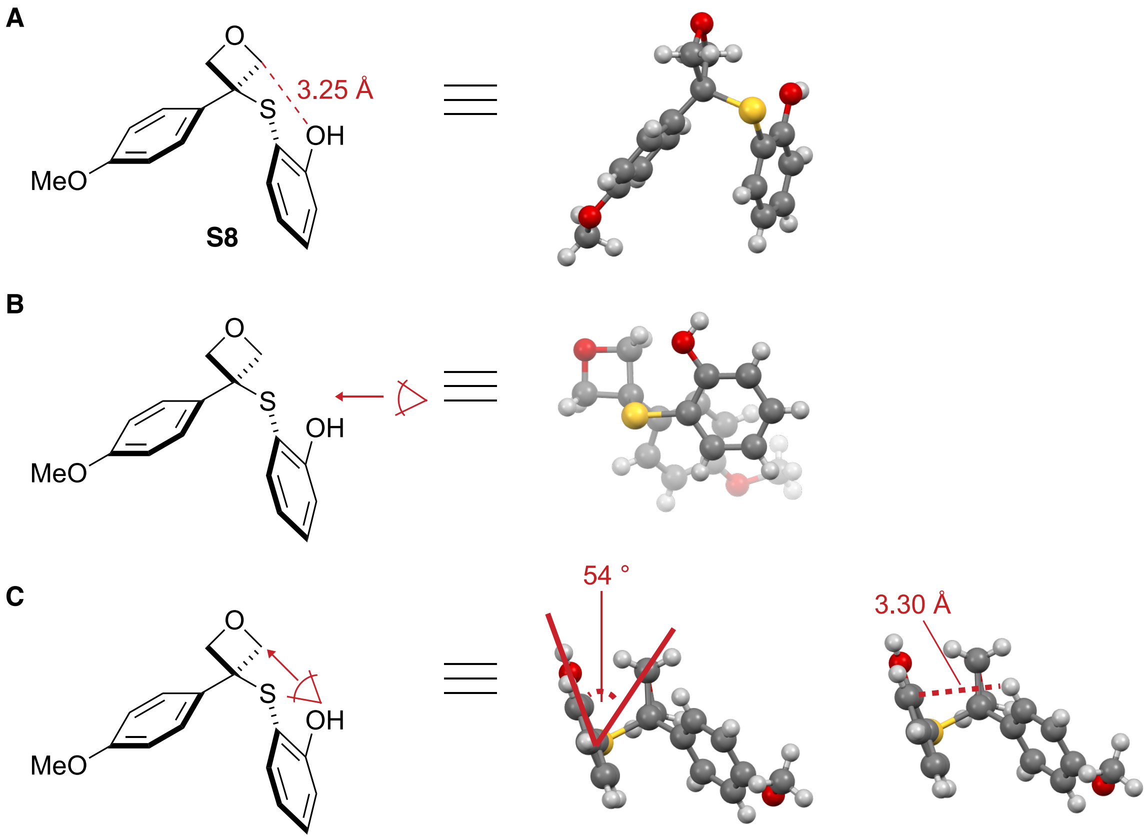

Fig. S2 Crystal structure of thioether **S8** from different perspectives reveals an optimal position of the phenol to attack the C–O σ* antibonding orbital on oxetane. The torsion angle in panel **C** was measured between O20–C14–S13–C4.

Experimental Details and Characterization Data

<u>3-Aryl-Oxetan-3-ols (**1a**–**m**)</u>

The same batches of the following, previously prepared oxetan-3-ols were used: 3-(4-methoxyphenyl)oxetan-3-ol **1a**, [7] 3-(3,4,5-trimethoxyphenyl)oxetan-3-ol **1e**[7], 3-(4-isopropoxyphenyl)oxetan-3-ol **1f**[7], 3-(4-(benzyloxy)phenyl)oxetan-3-ol **1h**,[7] 3-(4-((triisopropylsilyl)oxy)phenyl)oxetan-3-ol**1i**,[7] 3-(benzo[*d*][1,3]dioxol-5-yl)oxetan-3-ol **1j**,[2] 3-(6-methoxynaphthalen-2-yl)oxetan-3-ol **1k**[8] and 3-(4-(benzyloxy)-3-chlorophenyl)oxetan-3-ol **1l**.[2]

3-(4-Methoxy-2-methylphenyl)oxetan-3-ol (1b)

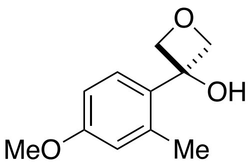

n-BuLi (1.53 M in hexanes, 3.0 mL, 4.59 mmol, 1.1 equiv) was added dropwise over 5 min to a solution of 1-bromo-4-methoxy-2-methylbenzene (1.0 g, 4.97 mmol, 1.2 equiv) in anhydrous THF (20 mL, 0.21 M) at –78 °C in a 100 mL round bottom flask. After stirring for 20 min, oxetan-3-one (0.27 mL, 4.14 mmol, 1.0 equiv) was added dropwise to the reaction mixture. After further 25 min of stirring at –78 °C, the reaction mixture was warmed to 25 °C, stirred for 1 h and quenched with distilled water (15 mL). The aqueous layer was extracted with diethylether (3 × 20 mL). The organic layers were combined, washed with brine (25 mL), dried over Na_2SO_4, filtered and concentrated *in vacuo* using a rotatory evaporator. Purification by flash column chromatography (50% EtOAc/pentane) afforded oxetanol **1b** as a white solid (627 mg, 78%). R_f = 0.37 (50% EtOAc/pentane); mp = 73–76 °C; IR (film)/cm^{-1} 3369 (br OH), 2946, 1609, 1503, 1247, 1129, 977, 867; ^1H NMR (400 MHz, $CDCl_3$) δ 7.07 (d, *J* = 8.4 Hz, 1 H, Ar-CH), 6.76–6.70 (m, 2 H, 2 × Ar-CH), 5.18 (d, *J* = 6.9 Hz, 2 H, C*H*HOC*H*H), 4.86 (d, *J* = 6.9 Hz, 2 H, CH*H*OCH*H*), 3.80 (s, 3 H, OCH_3), 2.54 (s, 1 H, OH), 2.23 (s, 3 H, CH_3); ^{13}C NMR (101 MHz, $CDCl_3$) δ 159.5 (Ar-C_qOMe), 138.1 (Ar-C_q), 131.8 (Ar-C_q), 126.9 (Ar-CH), 117.3 (Ar-CH), 110.6 (Ar-CH), 83.6 (CH_2OCH_2), 77.4 (C_q), 55.2 (OCH_3), 19.4 (CH_3); HRMS (ESI) *m/z* calcd for $C_{11}H_{13}O_2$ [M–OH]: 177.0916, found: 177.0912.

3-(3,4-Dimethoxyphenyl)oxetan-3-ol (1c)

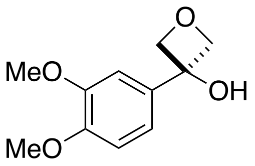

n-BuLi (1.426 M in hexanes, 8.45 mL, 12.0 mmol, 1.2 equiv) was added dropwise over 5 min to a solution of 1-bromo-3,4-dimethoxybenzene (1.7 mL, 13.0 mmol, 1.3 equiv) in anhydrous THF (45 mL, 0.2 M) at –78 °C in a 250 mL round bottom flask. After stirring for 20 min, oxetan-3-one (0.64 mL, 10.0 mmol, 1.0 equiv) was added dropwise to the reaction mixture. After further 25 min of stirring at –78 °C, the reaction mixture was warmed to 25 °C, stirred for 1 h and quenched with distilled water (60 mL). The aqueous layer was extracted with diethylether (3 × 45 mL). The organic layers were combined, washed with brine (50 mL), dried over Na_2SO_4, filtered and concentrated *in vacuo* using a rotatory evaporator. Purification by flash column chromatography (50% EtOAc/pentane) afforded oxetanol **1c** as a white solid (890 mg, 42%). R_f = 0.15 (50% EtOAc/pentane); mp = 51 °C [lit. 49–51 °C][7]; IR (film)/cm^{-1} 3325 (br OH), 2952, 2877, 1513, 1453, 1133, 1021, 969; ^1H NMR (400 MHz, $CDCl_3$) δ 7.14–7.10 (m, 2 H, 2 × Ar-CH), 6.90 (dd, ^3J = 8.2 Hz, ^4J = 1.2 Hz, 1 H, Ar-CH), 4.93–4.89 (m, 4 H, CH_2OCH_2), 3.92 (s, 3 H, OCH_3), 3.90 (s, 3 H, OCH_3), 1.72–1.65 (br m, 1 H, OH); ^{13}C NMR (101 MHz, $CDCl_3$) δ 149.1 (Ar-C_qOMe), 148.7 (Ar-C_qOMe), 134.9 (Ar-C_qC_q), 116.7 (Ar-CH), 110.9 (Ar-CH), 107.9 (Ar-CH), 85.6 (CH_2OCH_2), 75.7 (C_q), 55.9 (2 × × OCH_3). The observed characterization data (R_f, mp, IR, ^1H, ^{13}C) were consistent with that previously reported in the group.[7]

3-(2,3,4-Trimethoxyphenyl)oxetan-3-ol (1d)

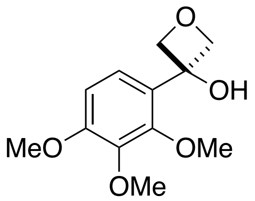

n-BuLi (1.426 M in hexanes, 2.5 mL, 3.6 mmol, 1.2 equiv) was added dropwise over 5 min to a solution of 1-bromo-2,3,4-trimethoxybenzene (954 mg, 3.9 mmol, 1.3 equiv) in anhydrous THF (14 mL, 0.2 M) at –78 °C in a 100 mL round bottom flask. After stirring for 20 min, oxetan-3-one (0.19 mL, 3.0 mmol, 1.0 equiv) was added dropwise to the reaction mixture. After further 25 min of stirring at –78 °C, the reaction mixture was warmed to 25 °C, stirred for 1 h and quenched with distilled water (20 mL). The aqueous layer was extracted with diethylether (3 × 20 mL). The organic layers were combined, washed with brine (30 mL), dried over Na_2SO_4, filtered and concentrated *in vacuo* using a rotatory evaporator. Purification by flash column chromatography (40% EtOAc/pentane) afforded oxetanol **1d** as a white solid (220 mg, 31%). R_f = 0.37 (50% EtOAc/pentane); mp = 69 °C; IR (film)/cm^{-1} 3340 (br OH), 2937, 1606, 1502, 1461, 1413, 1081, 1006, 947; ^{1}H NMR (400 MHz, $CDCl_3$) δ 6.94 (d, *J* = 8.6 Hz, 1 H, Ar-CH), 6.65 (d, *J* = 8.6 Hz, 1 H, Ar-CH), 5.02 (d, *J* = 6.9 Hz, 2 H, C*H*HOC*H*H), 4.85 (d, *J* = 6.9 Hz, 2 H, CH*H*OCH*H*), 3.96 (s, 3 H, OCH_3), 3.87 (s, 3 H, OCH_3), 3.86 (s, 3 H, OCH_3), 3.45 (br s, 1 H, OH); ^{13}C NMR (101 MHz, $CDCl_3$) δ 154.0 (Ar-C_qOMe), 151.3 (Ar-C_qOMe), 142.1 (Ar-C_qOMe), 127.4 (Ar-*C*$_q$C$_q$), 120.4 (Ar-CH), 106.6 (Ar-CH), 83.5 (CH_2OCH_2), 75.7 (C_q), 61.1 (OCH_3), 60.7 (OCH_3), 56.0 (OCH_3); HRMS (APCI) *m/z* calcd for $C_{12}H_{17}O_5^+$ $[M+H]^+$: 241.1071, found: 241.1073.

3-(4-Hydroxyphenyl)oxetan-3-ol (1g)

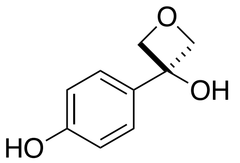

Tetrabutylammonium fluoride (1.0 M in THF, 3.72 mL, 3.72 mmol, 1.2 equiv) was added dropwise over 5 min to a solution of oxetanol **1i** (1.00 g, 3.1 mmol, 1.0 equiv) in anhydrous THF (10.3 mL, 0.3 M) at 0 °C in a 100 mL round bottom flask. After further 3 h of stirring at 0 °C, the reaction mixture was warmed to 25 °C and quenched with distilled water (75 mL). The aqueous layer was extracted with EtOAc (3 × 60 mL). The organic layers were combined, dried over Na_2SO_4, filtered and concentrated *in vacuo* using a rotatory evaporator. Purification by flash chromatography (60% EtOAc/hexane) afforded oxetanol **1g** as a white solid (460 mg, 89%). R_f = 0.21 (60% EtOAc/hexane); mp = 117 °C [lit. 84–85 °C][9]; IR (film)/cm^{-1} 3272 (br OH), 2960, 2886, 1614, 1594, 1519, 1431, 1349, 1256, 1225, 1182, 1166, 1140, 1126, 1057, 1014, 961, 938, 870, 832, 736; ^{1}H NMR (400 MHz, $(CD_3)_2SO$) δ 9.37 (s, 1 H, OH), 7.38–7.34 (m, 2 H, 2 × Ar-CH), 6.78–6.74 (m, 2 H, 2 × Ar-CH), 6.13 (s, 1 H, OH), 4.71 (d, *J* = 6.3 Hz, 2 H, CH*H*OCH*H*), 4.64 (d, *J* = 6.3 Hz, 2 H, C*H*HOC*H*H); ^{13}C NMR (101 MHz, $(CD_3)_2SO$) δ 156.4 (Ar-C_qOH), 134.5 (Ar-*C*$_q$C$_q$), 125.8 (2 × Ar-CH), 114.8 (2 × Ar-CH), 85.3 (CH_2OCH_2), 73.9 (C_q). The observed characterization data (^{1}H, ^{13}C) were consistent with that previously reported.[9]

3-([1,1'-Biphenyl]-4-yl)oxetan-3-ol (1m)

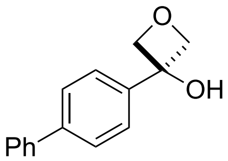

n-BuLi (1.47 M in hexanes, 1.5 mL, 2.2 mmol, 1.1 equiv) was added dropwise over 5 min to a solution of 4-bromobiphenyl (559 mg, 2.40 mmol, 1.2 equiv) in anhydrous THF (4.0 mL, 0.5 M) at –78 °C in a 25 mL round bottom flask. After stirring for 25 min, oxetan-3-one (130 μL, 2.0 mmol, 1.0 equiv) was added dropwise to the reaction mixture. After further 30 min of stirring at –78 °C, the reaction mixture was warmed to 25 °C, stirred for 1 h and quenched with distilled water (30 mL). The aqueous layer was extracted with diethylether (3 × 30 mL). The organic layers were combined, dried over Na_2SO_4, filtered and concentrated *in vacuo* using a rotatory evaporator. Purification by flash column chromatography (40% Et_2O/pentane) afforded oxetanol **1m** as a white solid (347 mg, 77%). R_f = 0.46 (70% Et_2O/pentane); mp = 144–146 °C [lit. 139–142 °C][10]; IR (film)/cm^{-1} 3358 (br, OH), 1420, 1374, 961; ^{1}H NMR (400 MHz, $CDCl_3$) δ 7.70–7.65 (m, 4 H, 4 × Ar-CH), 7.63–7.60 (m, 2 H, 2 × Ar-CH), 7.49–7.45 (m, 2 H, 2 × Ar-CH), 7.40–7.36 (m, 1 H, 2 × Ar-CH), 4.98 (d, *J* = 7.1 Hz, 2 H, C*H*HOC*H*H), 4.95 (d, *J* = 7.1 Hz, 2 H, CH*H*OCH*H*), 2.85 (s, 1 H, OH); ^{13}C NMR (101 MHz, $CDCl_3$) δ 141.2 (Ar-C_qC$_q$), 140.9 (Ar-C_qC$_q$), 140.4 (Ar-C_qC$_q$), 128.8 (2 × Ar-CH), 127.5 (Ar-CH), 127.4 (2 × Ar-CH), 127.1 (2 × Ar-CH), 124.9 (2 × Ar-CH), 85.7 (CH_2OCH_2), 75.7 (C_q). The observed characterization data (^{1}H and ^{13}C NMR) were consistent with that previously reported.[10]

1,4-Dioxanes (**2**–**25**)

(2-(4-Methoxyphenyl)-1,4-dioxan-2-yl)methanol (2)

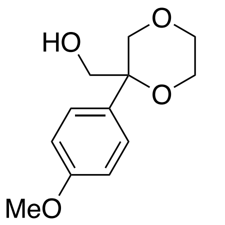

Oxetanol **1a** (991 mg, 5.5 mmol, 1.0 equiv) was added to a solution of trifluoromethane sulfonimide (155 mg, 0.55 mmol, 0.1 equiv) and ethylene glycol (1.53 mL, 27.5 mmol, 5.0 equiv) in anhydrous MeCN (18.3 mL, 0.3 M) in a flame-dried reaction tube (10–20 mL Biotage microwave reaction vial) under Ar. The reaction mixture was stirred at 50 °C for 24 h and then quenched with sat. aq. $NaHCO_3$ (50 mL). The aqueous layer was extracted with CH_2Cl_2 (3 × 50 mL). The organic layers were combined, dried over Na_2SO_4, filtered and concentrated *in vacuo* using a rotatory evaporator. Purification by flash column chromatography (20% Et_2O/CH_2Cl_2) afforded dioxane **2** as a light-yellow gum that crystallized upon slow evaporation from $CDCl_3$ to colorless crystals (1.11 g, 90%). R_f = 0.16 (20% Et_2O/CH_2Cl_2); mp = 55 °C; IR (film)/cm^{-1} 3451 (br, OH), 2961, 1512, 1249, 1110, 907; ^{1}H NMR (400 MHz, $CDCl_3$) δ 7.42–7.38 (m, 2 H, 2 × Ar-CH), 6.97–6.93 (m, 2 H, 2 × Ar-CH), 4.27 (d, *J* = 12.3 Hz, 1 H, C_qC*H*HOCH$_2$), 3.99 (d, *J* = 12.3 Hz, 1 H, C_qCH*H*OCH$_2$), 3.82 (s, 3 H, OCH_3), 3.76–3.59 (m, 6 H, 3 × OCH_2), 1.88 (br s, 1 H, OH); ^{13}C NMR (101 MHz $CDCl_3$) δ 159.1 (Ar-C_qOMe), 131.0 (Ar-*C*$_q$C$_q$), 128.2 (2 × Ar-CH), 114.0 (2 × Ar-CH), 76.8 (C_q), 69.2 (C_q*C*H$_2$OCH$_2$), 67.6 (OCH_2), 66.8 (OCH_2), 61.3 (C_q*O*CH$_2$), 55.2 (OCH_3); HRMS (ESI) *m/z* calcd for $C_{14}H_{20}NO_4$ [M+MeCN+H]: 266.1392, found: 266.1393.

Notes:

***2** was further characterized by X-ray crystallography (see Fig. S20–S21).*

(2-(4-Methoxy-2-methylphenyl)-1,4-dioxan-2-yl)methanol (3)

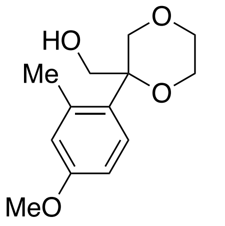

Prepared according to **General Procedure A** using oxetanol **1b** (48.6 mg) and ethylene glycol (0.07 mL). Purification by flash column chromatography (50% Et_2O/pentane) afforded dioxane **3** as a transparent oil (50.5 mg, 85%). R_f = 0.30 (50% EtOAc/pentane); IR (film)/cm^{-1} 3446 (br, OH), 2952, 1607, 1248, 1109, 1068; ^{1}H NMR (400 MHz, $CDCl_3$) δ 7.36–7.34 (m, 1 H, Ar-CH), 6.78–6.75 (m, 2 H, 2 × Ar-CH), 4.50 (d, *J* = 12.5 Hz, 1 H, C_qC*H*HOCH$_2$), 4.00 (d, *J* = 12.5 Hz, 1 H, C_qCH*H*OCH$_2$), 3.84 (dd, ^2J = 12.0 Hz, ^3J = 4.7 Hz, 1 H, C*H*HOH), 3.81 (s, 3 H, OCH_3), 3.78–3.74 (m, 1 H, OCH), 3.67–3.60 (m, 3 H, 3 × OCH), 3.53 (dd, ^2J = 12.0 Hz, ^3J = 8.4 Hz, 1 H, CH*H*OH), 2.48 (s, 3 H, CH_3), 1.99 (dd, *J* = 8.4, 4.7 Hz, 1 H, OH); ^{13}C NMR (101 MHz, $CDCl_3$) δ 158.9 (Ar-C_qOMe), 139.4 (Ar-C_q), 131.0 (Ar-CH), 127.6 (Ar-C_q), 118.8 (Ar-CH), 110.6 (Ar-CH), 78.9 (C_q), 69.7 (OCH_2), 66.9 (OCH_2), 65.7 (OCH_2), 61.2 (OCH_2), 55.1 (OCH_3), 21.9 (CH_3); HRMS (ESI) *m/z* calcd for $C_{15}H_{22}NO_4$ [M+H+MeCN]: 280.1549, found: 280.1547.

(2-(3,4-Dimethoxyphenyl)-1,4-dioxan-2-yl)methanol (4)

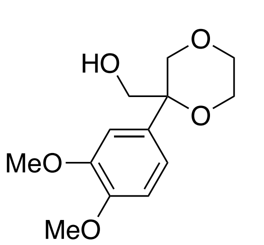

Prepared according to **General Procedure A** using oxetanol **1c** (53.0 mg) and ethylene glycol (0.07 mL). Purification by flash column chromatography (50% EtOAc/pentane) afforded dioxane **4** as a transparent oil (57.2 mg, 90%). R_f = 0.45 (50% EtOAc/pentane); IR (film)/cm^{-1} 3463 (br, OH), 2952, 2863, 1513, 1461, 1260, 1111, 1025; ^{1}H NMR (400 MHz, $CDCl_3$) δ 7.03 (d, *J* = 2.1 Hz,1 H, Ar-C_qAr-CHAr-C_q), 7.00 (dd, ^3J = 8.3 Hz, ^4J = 2.1 Hz, 1 H, Ar-C_qOMeAr-CHAr-C*H*), 6.90 (d, *J* = 8.3 Hz, 1 H, Ar-C_qOMeAr-C*H*Ar-CH), 4.27 (d, *J* = 12.3 Hz, 1 H, C_qC*H*HOCH$_2$), 3.99 (d, *J* = 12.3 Hz, 1 H, C_qCH*H*OCH$_2$), 3.91 (s, 3 H, OCH_3), 3.89 (s, 3 H, OCH_3), 3.79–3.61 (m, 6 H, 3 × OCH_2), 1.90 (dd, *J* = 8.0, 5.4 Hz, 1 H, OH); ^{13}C NMR (101 MHz, $CDCl_3$) δ 149.2 (Ar-C_qOMe), 148.6 (Ar-C_qOMe), 131.5 (Ar-*C*$_q$C$_q$), 119.3 (Ar-CH), 111.0 (Ar-CH), 109.9 (Ar-CH), 76.9 (C_q), 69.2 (C_q*C*H$_2$OCH$_2$), 67.6 (OCH_2), 66.7 (OCH_2), 61.3 (C_q*O*CH$_2$), 55.94 (OCH_3), 55.87 (OCH_3); HRMS (APCI) *m/z* calcd for $C_{13}H_{17}O_4^+$ [M–OH]$^+$: 237.1121, found: 237.1122.

(2-(2,3,4-Trimethoxyphenyl)-1,4-dioxan-2-yl)methanol (5)

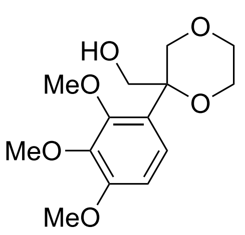

Oxetanol **1d** (54.0 mg, 0.22 mmol, 1.0 equiv) was added to a solution of trifluoromethane sulfonimide (6.3 mg, 0.022 mmol, 0.1 equiv) and ethylene glycol (0.063 mL, 1.10 mmol, 5.0 equiv) in anhydrous MeCN (0.75 mL, 0.3 M) in a flame-dried reaction tube under Ar. The reaction mixture was stirred at 50 °C for 24 h and then quenched with sat. aq. $NaHCO_3$ (10 mL). The aqueous layer was extracted with CH_2Cl_2 (3 × 10 mL). The organic layers were combined, dried over Na_2SO_4, filtered and concentrated *in vacuo* using a rotatory evaporator. Purification by flash column chromatography (40% EtOAc/pentane) afforded impure dioxane **5** as a light-yellow gum (32.6 mg). Repurification by flash column chromatography (10–30% acetone/pentane) afforded dioxane **5** as a colorless gum (16.7 mg, 27%). R_f = 0.29 (30% acetone/pentane); IR (film)/cm^{-1} 3465 (br, OH), 2939, 1595, 1492, 1457, 1409, 1085, 1012; ^{1}H NMR (400 MHz, $CDCl_3$) δ 7.25 (d, *J* = 8.9 Hz, 1 H, Ar-C_qC_qAr-CH), 6.70 (d, *J* = 8.9 Hz, 1 H, Ar-C_qOMeAr-CH), 4.25 (d, *J* = 12.0 Hz, 1 H, C_qC*H*HOCH$_2$), 4.13 (d, *J* = 12.0 Hz, 1 H, C_qCH*H*OCH$_2$), 3.99 (dd, *J* = 11.6, 7.1 Hz, 1 H, C_qC*H*HOH), 3.91 (s, 3 H, OCH_3), 3.87 (s, 3 H, OCH_3), 3.85 (s, 3 H, OCH_3), 3.85–3.74 (m, 4 H, C_qCH*H*OH + C_qOC*H$_2$*C*H*H), 3.66 (ddd, *J* = 11.4, 4.3, 4.3 Hz, 1 H, C_qOCH_2CH*H*), 1.96 (dd, *J* = 7.1, 5.6 Hz, 1 H, OH); ^{13}C NMR (101 MHz, $CDCl_3$) δ 153.5 (Ar-C_qOMe), 151.9 (Ar-C_qOMe), 142.5 (Ar-C_qOMe), 124.2 (Ar-*C*$_q$C$_q$), 123.4 (Ar-CH), 106.8 (Ar-CH), 77.0 (C_q), 70.2 (C_qCH_2O*C*H$_2$), 66.7 (C_qCH_2OCH$_2$), 64.6 (C_q*C*H$_2$OH), 61.2 (C_qO*C*H$_2$), 60.8 (OCH_3), 60.5 (OCH_3), 55.9 (OCH_3); HRMS (ESI) *m/z* calcd for $C_{14}H_{19}O_5$ [M–OH]: 267.1232, found: 267.1232.

Notes:

The C_q signal at 77.0 ppm in the ^{13}C NMR spectrum was observed as a small shoulder of the $CDCl_3$ solvent peak. Its identity was confirmed by a HMBC correlation spectrum.

The singlet at 3.85 ppm (3 H) and multiplet at 3.85–3.74 ppm (4 H) in the ^{1}H NMR spectrum were integrated together to 7 H.

2-(3,4,5-Trimethoxyphenyl)-1,4-dioxan-2-yl)methanol (6)

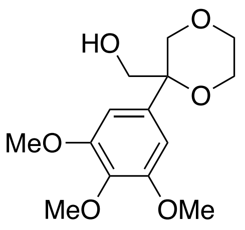

Prepared according to **General Procedure A** using oxetanol **1e** (60.0 mg) and ethylene glycol (0.07 mL). Purification by flash column chromatography (70% EtOAc/pentane) afforded dioxane **6** as a transparent oil (31.0 mg, 44%). R_f = 0.11 (50% EtOAc/pentane); IR (film)/cm^{-1} 3466 (br, OH), 2963, 1588, 1461, 1416, 1334, 1241, 1126, 910, 731; ^{1}H NMR (400 MHz, $CDCl_3$) δ 6.68 (s, 2 H, 2 × Ar-CH), 4.23 (d, *J* = 12.3 Hz, 1 H, C_qC*H*HOCH$_2$), 3.99 (d, *J* = 12.3 Hz, 1 H, C_qCH*H*OCH$_2$), 3.89 (s, 6 H, 2 × OCH_3), 3.86 (s, 3 H, OCH_3), 3.83–3.66 (m, 6 H, 3 × OCH_2); ^{13}C NMR (101 MHz, $CDCl_3$) δ 153.4 (2 × Ar-C_qOMe), 137.5 (Ar-*C*$_q$C$_q$), 134.9 (Ar-C_qOMe), 103.8 (2 × Ar-CH), 77.2 (C_q), 69.4 (C_q*C*H$_2$OCH$_2$), 67.4 (OCH_2), 66.7 (OCH_2), 61.4 (C_qO*C*H$_2$), 60.8 (OCH_3), 56.2 (2 × OCH_3); HRMS (APCI) *m/z* calcd for $C_{14}H_{21}O_6^+$ $[M+H]^+$: 285.1333, found: 285.1329.

(2-(4-Isopropoxyphenyl)-1,4-dioxan-2-yl)methanol (7)

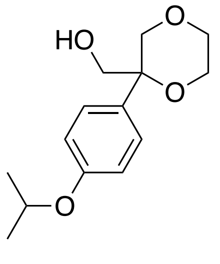

Prepared according to **General Procedure A** using oxetanol **1f** (52.0 mg) and ethylene glycol (0.07 mL). Purification by flash column chromatography (50% EtOAc/pentane) afforded dioxane **7** as a pale pink oil (49.4 mg, 78%). R_f = 0.50 (30% EtOAc/pentane); IR (film)/cm^{-1} 3437 (br, OH), 2974, 1610, 1510, 1241, 1111; ^{1}H NMR (400 MHz, $CDCl_3$) δ 7.39–7.35 (m, 2 H, 2 × Ar-CH), 6.93–6.90 (m, 2 H, 2 × Ar-CH), 4.56 (hept, 1 H, *J* = 6.1 Hz, CH), 4.26 (d, *J* = 12.3 Hz, 1 H, C_qC*H*HOCH$_2$), 3.98 (d, *J* = 12.3 Hz, 1 H, C_qCH*H*OCH$_2$), 3.76–3.61 (m, 6 H, 3 × OCH_2), 1.99 (br s, 1 H, OH), 1.35 (d, *J* = 6.1 Hz, 6 H, 2 × CH_3); ^{13}C NMR (101 MHz, $CDCl_3$) δ 157.5 (Ar-C_qO*i*-Pr), 130.7 (Ar-*C*$_q$C$_q$), 128.1 (2 × Ar-CH), 115.8 (2 × Ar-CH), 76.8 (C_q), 69.8 (CH or C_q*C*H$_2$OCH$_2$), 69.2 (CH or C_q*C*H$_2$OCH$_2$), 67.6 (OCH_2), 66.8 (OCH_2), 61.3 (C_qO*C*H$_2$), 22.0 (2 × CH_3); HRMS (APCI) *m/z* calcd for $C_{14}H_{19}O_3^+$ $[M–OH]^+$: 235.1329, found: 235.1333.

4-(2-(Hydroxymethyl)-1,4-dioxan-2-yl)phenol (8)

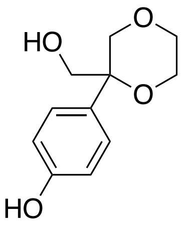

Oxetanol **1g** (41.4 mg, 0.25 mmol, 1.0 equiv) was added to a solution of trifluoromethane sulfonimide (7.0 mg, 0.025 mmol, 0.1 equiv) and ethylene glycol (0.7 mL, 1.25 mmol, 5.0 equiv) in anhydrous MeCN (0.83 mL, 0.3 M) in a flame-dried reaction tube under Ar. The reaction mixture was stirred at 50 °C for 24 h and then quenched with sat. aq. $NaHCO_3$ (10 mL). The aqueous layer was acidified to pH 1 using 1 M aq. HCl and extracted with CH_2Cl_2 (6 × 10 mL) and EtOAc (6 × 10 mL). The organic layers were combined, dried over Na_2SO_4, filtered and concentrated *in vacuo* using a rotatory evaporator. Purification by flash column chromatography (65% EtOAc/pentane) afforded impure dioxane **8** as a white solid. Repurification by flash column chromatography (0–5% MeOH/CH_2Cl_2) afforded dioxane **8** as a white solid (25.9 mg, 49%). R_f = 0.18 (5% MeOH/CH_2Cl_2); mp = 119–126 °C; IR (film)/cm^{-1} 3367 (br, OH), 2508, 1613, 1515, 1248, 1104; ^{1}H NMR (400 MHz, CD_3OD) δ 7.31–7.28 (m, 2 H, 2 × Ar-CH), 6.82–6.78 (m, 2 H, 2 × Ar-CH), 4.25 (d, *J* = 12.1 Hz, 1 H, $C_qC\mathit{H}HOCH_2$), 3.91 (d, *J* = 12.1 Hz, 1 H, $C_qCH\mathit{H}OCH_2$), 3.70–3.55 (m, 6 H, 3 × OCH_2); ^{13}C NMR (101 MHz, CD_3OD) δ 157.8 (Ar-C_qOH), 131.8 (Ar-C_qC_q), 129.5 (2 × Ar-CH), 116.1 (2 × Ar-CH), 78.1 (C_q), 70.5 ($C_q\mathit{C}H_2OCH_2$), 68.2 (OCH_2), 67.9 (OCH_2), 62.2 ($C_qO\mathit{C}H_2$); HRMS (APCI) *m/z* calcd for $C_{11}H_{13}O_4$ [M–H]: 209.0808, found: 209.0806.

(2-(4-(Benzyloxy)phenyl)-1,4-dioxan-2-yl)methanol (9)

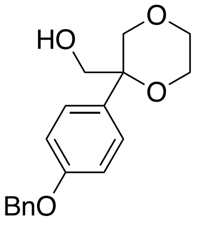

Prepared according to **General Procedure A** using oxetanol **1h** (64.1 mg) and ethylene glycol (0.07 mL). Purification by flash column chromatography (50–100% Et_2O/pentane) afforded dioxane **9** as a white solid (65.7 mg, 87%). R_f = 0.46 (50% EtOAc/pentane); mp = 112 °C; IR (film)/cm^{-1} 3444 (br, OH), 2960, 1510, 1245, 1111, 910; ^{1}H NMR (400 MHz, $CDCl_3$) δ 7.46–7.38 (m, 6 H, 2 × Ar-CH + 4 × Ph-CH), 6.36–7.32 (m, 1 H, Ph-CH), 7.04–7.01 (m, 2 H, 2 × Ar-CH), 5.08 (s, 2 H, CH_2Ph), 4.28 (d, *J* = 12.3 Hz, 1 H, $C_qC\mathit{H}HOCH_2$), 4.00 (d, *J* = 12.3 Hz, 1 H, $C_qCH\mathit{H}OCH_2$), 3.77–3.59 (m, 6 H, 3 × OCH_2), 1.90 (dd, *J* = 8.1, 5.2 Hz, 1 H, OH); ^{13}C NMR (101 MHz, $CDCl_3$) δ 158.4 (Ar-C_qOBn), 136.9 (Ar-C_qC_q), 131.2 (Ar-C_qC_q), 128.6 (2 × Ar-CH), 128.2 (2 × Ar-CH), 128.0 (Ph-CH), 127.5 (2 × Ar-CH), 115.0 (2 × Ar-CH), 76.8 (C_q), 70.0 (CH_2Ph), 69.2 ($C_q\mathit{C}H_2OCH_2$), 67.6 (OCH_2), 66.8 (OCH_2), 61.3 ($C_qO\mathit{C}H_2$); HRMS (APCI) *m/z* calcd for $C_{18}H_{19}O_3^+$ $[M–OH]^+$: 283.1329, found: 283.1320.

(2-(4-((Triisopropylsilyl)oxy)phenyl)-1,4-dioxan-2-yl)methanol (10)

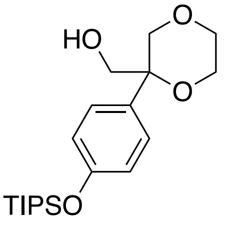

Prepared according to **General Procedure A** using oxetanol **1i** (80.6 mg) and ethylene glycol (0.07 mL). Purification by flash column chromatography (10–50% EtOAc/pentane) afforded dioxane **10** as a colorless oil (31.1 mg, 34%) followed by phenol dioxane **8** as a white solid (9.5 mg, 18%). R_f = 0.54 (50% EtOAc/pentane); IR (film)/cm^{-1} 3460 (br, OH), 2945, 2867, 1607, 1509, 1266, 1114, 916; ^{1}H NMR (400 MHz, $CDCl_3$) δ 7.34–7.30 (m, 1 H, 2 × Ar-CH), 6.93–6.89 (m, 2 H, 2 × Ar-CH), 4.26 (d, *J* = 12.3 Hz, 1 H, $C_qC\mathit{H}HOCH_2$), 3.99 (d, *J* = 12.3 Hz, 1 H, $C_qCH\mathit{H}OCH_2$), 3.75–3.59 (m, 6 H, 3 × OCH_2), 1.85 (dd, *J* = 8.1, 5.2 Hz, 1 H, OH), 1.31–1.22 (m, 3 H, 3 × CH), 1.11 (d, *J* = 7.3 Hz, 18 H, 6 × CH_3); ^{13}C NMR (101 MHz, $CDCl_3$) δ 155.6 (Ar-C_qOTIPS), 131.2 (Ar-C_qC_q), 128.1 (2 × Ar-CH), 119.9 (2 × Ar-CH), 76.8 (C_q), 69.1 ($C_q\mathit{C}H_2OCH_2$), 67.5 (OCH_2), 66.8 (OCH_2), 61.3 ($C_qO\mathit{C}H_2$), 17.9 (6 × CH_3), 12.6 (3 × CH); HRMS (APCI) $C_{20}H_{38}NO_4Si^+$ $[M+NH_4]^+$: 384.2565, found: 384.2568.

(2-(Benzo[d][1,3]dioxol-5-yl)-1,4-dioxan-2-yl)methanol (11)

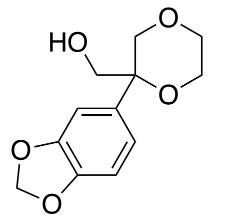

Prepared according to **General Procedure A** using oxetanol **1j** (49.0 mg) and ethylene glycol (0.07 mL). Purification by flash column chromatography (40% EtOAc/pentane) afforded dioxane **11** as a pale yellow solid (45.8 mg, 77%). R_f = 0.19 (20% Et_2O/CH_2Cl_2); mp = 115 °C; IR (film)/cm^{-1} 3449 (br, OH), 2871, 1487, 1236, 1110, 1037; ^{1}H NMR (400 MHz, $CDCl_3$) δ 6.99 (d, J = 1.8 Hz, 1 H, Ar-C_qAr-CHAr-C_q), 6.94 (dd, ^3J = 8.1 Hz, ^4J = 1.8 Hz, 1 H, Ar-C*H*Ar-CHAr-C_qO), 6.85 (d, J = 8.1 Hz, 1 H, Ar-CHAr-C*H*Ar-C_qO), 5.99 (d, J = 1.4 Hz, 1 H, OC*H*HO), 5.98 (d, J = 1.4 Hz, 1 H, OCH*H*O), 4.23 (d, J = 12.3 Hz, 1 H, C_qC*H*HOCH$_2$), 3.98 (d, J = 12.3 Hz, 1 H, C_qCH*H*OCH$_2$), 3.79–3.58 (m, 6 H, 3 × OCH$_2$), 1.84 (dd, J = 7.8, 5.4 Hz, 1 H, OH); ^{13}C NMR (101 MHz, $CDCl_3$) δ 148.1 (Ar-C_qO), 147.1 (Ar-C_qO), 133.0 (Ar-*C*$_q$C$_q$), 120.4 (Ar-CH), 108.3 (Ar-CH), 107.4 (Ar-CH), 101.1 (OCH$_2$O), 77.0 (C_q), 69.3 (C_q*C*H$_2$OCH$_2$), 67.6 (OCH$_2$), 66.7 (OCH$_2$), 61.3 (C_qO*C*H$_2$); HRMS (APCI) $C_{12}H_{13}O_4^+$ [M–OH]$^+$: 221.0808, found: 221.0807.

(2-(6-Methoxynaphthalen-2-yl)-1,4-dioxan-2-yl)methanol (12)

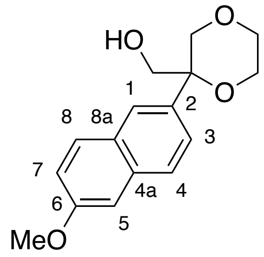

Prepared according to **General Procedure A** using oxetanol **1k** (57.0 mg) and ethylene glycol (0.07 mL). Purification by flash column chromatography (50% EtOAc/pentane) afforded dioxane **12** as a transparent oil that solidified in the freezer to a white solid (37.0 mg, 54%). R_f = 0.45 (50% EtOAc/pentane); mp = 97 °C; IR (film)/cm^{-1} 3437 (br, OH), 2859, 1607, 1484, 1103, 1029; ^{1}H NMR (400 MHz, $CDCl_3$) δ 7.88 (d, J = 1.9 Hz, 1 H, H1), 7.79 (d, J = 8.8 Hz, 1 H, H4), 7.76 (d, J = 8.9 Hz, 1 H, H8), 7.55 (dd, ^3J = 8.8 Hz, ^4J = 1.9 Hz, 1 H, H3), 7.18 (dd, ^3J = 8.9 Hz, ^4J = 2.5 Hz, 1 H, H7), 7.15 (d, J = 2.5 Hz, 1 H, H5), 4.43 (d, J = 12.3 Hz, 1 H, C_qC*H*HOCH$_2$), 4.08 (d, J = 12.3 Hz, 1 H, C_qCH*H*OCH$_2$), 3.93 (s, 3 H, OCH$_3$), 3.79–3.64 (m, 6 H, 3 × OCH$_2$); ^{13}C NMR (101 MHz, $CDCl_3$) δ 157.9 (C6), 134.1 (C2 or C4a), 134.0 (C2 or C4), 129.7 (C8), 128.7 (C8a), 127.3 (C4), 126.4 (C1), 124.8 (C3), 119.0 (C7), 105.4 (C5), 77.2 (C_q), 69.2 (C_q*C*H$_2$OCH$_2$), 67.5 (OCH$_2$), 66.8 (OCH$_2$), 61.5 (C_qO*C*H$_2$), 55.3 (OCH$_3$); HRMS (APCI) $C_{16}H_{19}O_4^+$ [M+H]$^+$: 275.1278, found: 275.1273.

(2-(4-(Benzyloxy)-3-chlorophenyl)-1,4-dioxan-2-yl)methanol (13)

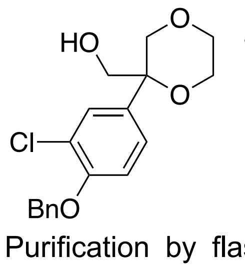

Oxetanol **1l** (72.6 mg, 0.25 mmol, 1.0 equiv) was added to a solution of trifluoromethane sulfonimide (7.0 mg, 0.025 mmol, 0.1 equiv) and ethylene glycol (0.07 mL, 1.25 mmol, 5.0 equiv) in anhydrous MeCN (0.83 mL, 0.3 M) in a flame-dried reaction tube under Ar. The reaction mixture was stirred at 50 °C for **32 h** and then quenched with sat. aq. $NaHCO_3$ (10 mL). The aqueous layer was extracted with CH_2Cl_2 (3 × 10 mL). The organic layers were combined, dried over Na_2SO_4, filtered and concentrated *in vacuo* using a rotatory evaporator. Purification by flash column chromatography (50% EtOAc/pentane) afforded dioxane **13** as a colorless gum (33.3 mg, 40%). R_f = 0.26 (50% EtOAc/pentane); IR (film)/cm^{-1} 3440 (br, OH), 2865, 1499, 1261, 1111, 1060; ^{1}H NMR (400 MHz, $CDCl_3$) δ 7.53 (d, J = 2.3 Hz, 1 H, Ar-C_qClAr-CH), 7.49–7.47 (m, 2 H, 2 × Ar-CH), 7.42–7.32 (m, 3 H, 3 × Ar-CH), 7.28 (dd, ^3J = 8.6 Hz, ^4J = 2.3 Hz, 1 H, Ar-C*H*Ar-CHAr-C_qOBn), 6.99 (d, J = 8.6 Hz, 1 H, Ar-CHAr-C*H*Ar-C_qOBn), 5.18 (s, 2 H, CH$_2$Ph), 4.21 (d, J = 12.3 Hz, 1 H, C_qC*H*HOCH$_2$), 3.98 (d, J = 12.3 Hz, 1 H, C_qCH*H*OCH$_2$), 3.77–3.59 (m, 6 H, 3 × OCH$_2$); ^{13}C NMR (101 MHz, $CDCl_3$) δ 153.7 (Ar-C_qOBn), 136.4 (Ar-*C*$_q$C$_q$), 132.7 (Ph-*C*$_q$CH$_2$), 129.0 (Ph-CH), 128.6 (2 × Ph-CH), 128.0 (Ar-C_qClAr-*C*H), 127.0 (2 × Ph-CH), 126.3 (Ar-*C*HAr-CHAr-C_qOBn), 123.6 (Ar-C_qCl), 113.8 (Ar-CHAr-*C*HAr-C_qOBn), 76.5 (C_q), 70.8 (CH$_2$Ph), 69.0 (C_q*C*H$_2$OCH$_2$), 67.4 (OCH$_2$), 66.7 (OCH$_2$), 61.3 (C_qO*C*H$_2$); HRMS (APCI) $C_{18}H_{18}O_3{}^{35}Cl^+$ [M–OH]$^+$: 317.0939, found: 317.0938.

(2-([1,1'-Biphenyl]-4-yl)-1,4-dioxan-2-yl)methanol (14)

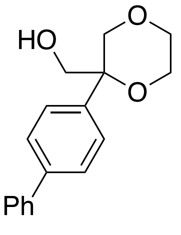

Prepared according to **General Procedure A** using oxetanol **1m** (57.1 mg) and ethylene glycol (0.07 mL). Purification by flash column chromatography (50% EtOAc/pentane) afforded dioxane **14** as a white solid (10.0 mg, 15%). R_f = 0.39 (50% EtOAc/pentane); mp = 112 °C; IR (film)/cm^{-1} 3403 (br, OH), 2919, 1107, 1070, 1029; ^{1}H NMR (400 MHz, $CDCl_3$) δ 7.66–7.64 (m, 2 H, 2 × Ar-CH), 7.62–7.60 (m, 2 H, 2 × Ar-CH), 7.57–7.55 (m, 2 H, 2 × Ar-CH), 7.48–7.44 (m, 2 H, 2 × Ar-CH), 7.39–7.35 (m, 1 H, Ph-CH), 4.35 (d, *J* = 12.3 Hz, 1 H, C_qCH*H*OCH_2), 4.06 (d, *J* = 12.3 Hz, 1 H, C_qCH*H*OCH_2), 3.87–3.67 (m, 6 H, 3 × OCH_2), 1.93 (dd, *J* = 7.9, 5.4 Hz, 1 H, OH); ^{13}C NMR (101 MHz, $CDCl_3$) δ 140.7 (Ar-C_q), 140.6 (Ar-C_q), 138.2 (Ar-C_q), 128.8 (2 × Ar-CH), 127.5 (2 × Ar-CH), 127.4 (Ph-CH), 127.3 (2 × Ar-CH), 127.1 (2 × Ar-CH), 69.3 ($C_qCH_2OCH_2$), 67.6 (OCH_2), 66.8 (OCH_2), 61.5 (C_qOCH_2); HRMS (APCI) *m/z* calcd for $C_{17}H_{17}O_2^+$ $[M–OH]^+$: 253.1223, found: 253.1225.

Notes:

The C_q signal was not observed in the ^{13}C NMR spectrum. Presumably it is hidden under the $CDCl_3$ peak.

2-Methylpropane-1,2-diol (S11)[11]

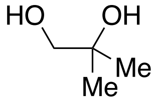

A solution of 2,2-dimethyloxirane (0.44 mL, 5.0 mmol, 1.0 equiv) in distilled water (29 mL, 0.17 M) in a 100 mL round bottom flask attached to a findenser was stirred at 60 °C for 3.5 h. The reaction mixture was extracted with EtOAc (6 × 100 mL) and the organic extracts washed with brine (500 mL). The aqueous phase was re-extracted with a 3:1 $CHCl_3$:isopropanol mixture (3 × 100 mL) and dichloromethane (3 × 100 mL). The combined organic extracts were dried over Na_2SO_4, filtered and concentrated *in vacuo* using a rotatory evaporator. Purification by flash column chromatography (70% Et_2O/pentane) afforded diol **S11** as a pale yellow liquid (255 mg, 57%). R_f = 0.18 (70% EtOAc/pentane); IR (film)/cm^{-1} 3325 (br, OH), 2972, 1159, 1048, 907; ^{1}H NMR (400 MHz, $CDCl_3$) δ 3.44 (s, 2 H, CH_2), 1.96 (br s, 2 H, 2 × OH), 1.23 (s, 6 H, 2 × CH_3); ^{13}C NMR (101 MHz, $CDCl_3$) δ 71.1 (C_q), 70.7 (CH_2), 25.5 (2 × CH_3). The observed characterization data (^{1}H, ^{13}C) were consistent with that previously reported.[12]

(2-(4-Methoxyphenyl)-5,5-dimethyl-1,4-dioxan-2-yl)methanol (15)

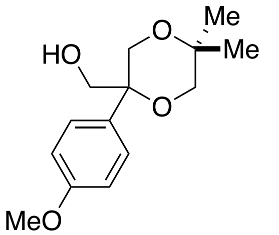

Prepared according to **General Procedure B** using 2-methylpropane-1,2-diol **S11** (0.11 mL). Purification by flash column chromatography (5–20% Et_2O/CH_2Cl_2) afforded dioxane **15** as a pale pink gum that solidified in the freezer to a pale pink solid (39.1 mg, 62%). rr in crude reaction mixture: 96:4. R_f = 0.20 (15% Et_2O/CH_2Cl_2); mp = 70–80 °C; IR (film)/cm^{-1} 3442 (br, OH), 2972, 1512, 1248, 1078; ^{1}H NMR (400 MHz, $CDCl_3$) δ 7.43–7.39 (m, 2 H, 2 × Ar-CH), 6.95–6.92 (m, 2 H, 2 × Ar-CH), 4.18 (d, *J* = 12.7 Hz, 1 H, C_qC*H*$HOC_q(Me)_2$), 4.11 (d, *J* = 12.7 Hz, 1 H, C_qCH*H*$OC_q(Me)_2$), 3.82 (s, 3 H, OCH_3), 3.70 (dd, ^2J = 11.9 Hz, ^3J = 3.4 Hz, 1 H, C*H*HOH), 3.63 (dd, ^2J = 11.9 Hz, ^3J = 6.9 Hz, 1 H, CH*H*OH), 3.39 (s, 2 H, $C_q(Me)_2CH_2$), 2.03–2.00 (br m, 1 H, OH), 1.36 (s, 3 H, CH_3), 1.06 (s, 3 H, CH_3); ^{13}C NMR (101 MHz $CDCl_3$) δ 159.1 (Ar-C_qOMe), 131.0 (Ar-C_qC_q), 128.1 (2 × Ar-CH), 113.9 (2 × Ar-CH), 76.0 (Ar-C_qC_q), 69.8 ($C_q(Me)_2$), 69.0 ($C_q(Me)_2CH_2$), 67.4 ($C_qCH_2OC_q(Me)_2$), 62.5 (CH_2OH), 55.2 (OCH_3), 24.5 (CH_3), 22.1 (CH_3); HRMS (ESI) *m/z* calcd for $C_{14}H_{19}O_3$ [M–OH]: 235.1334, found: 235.1326.

Notes:

*Using **General Conditions A**, **15** was obtained in 66% yield (41.4 mg) and a regioisomeric ratio of 95:5.*

*The regiochemistry of **15** was determined with an HMBC correlation spectrum (see Fig. S3).*

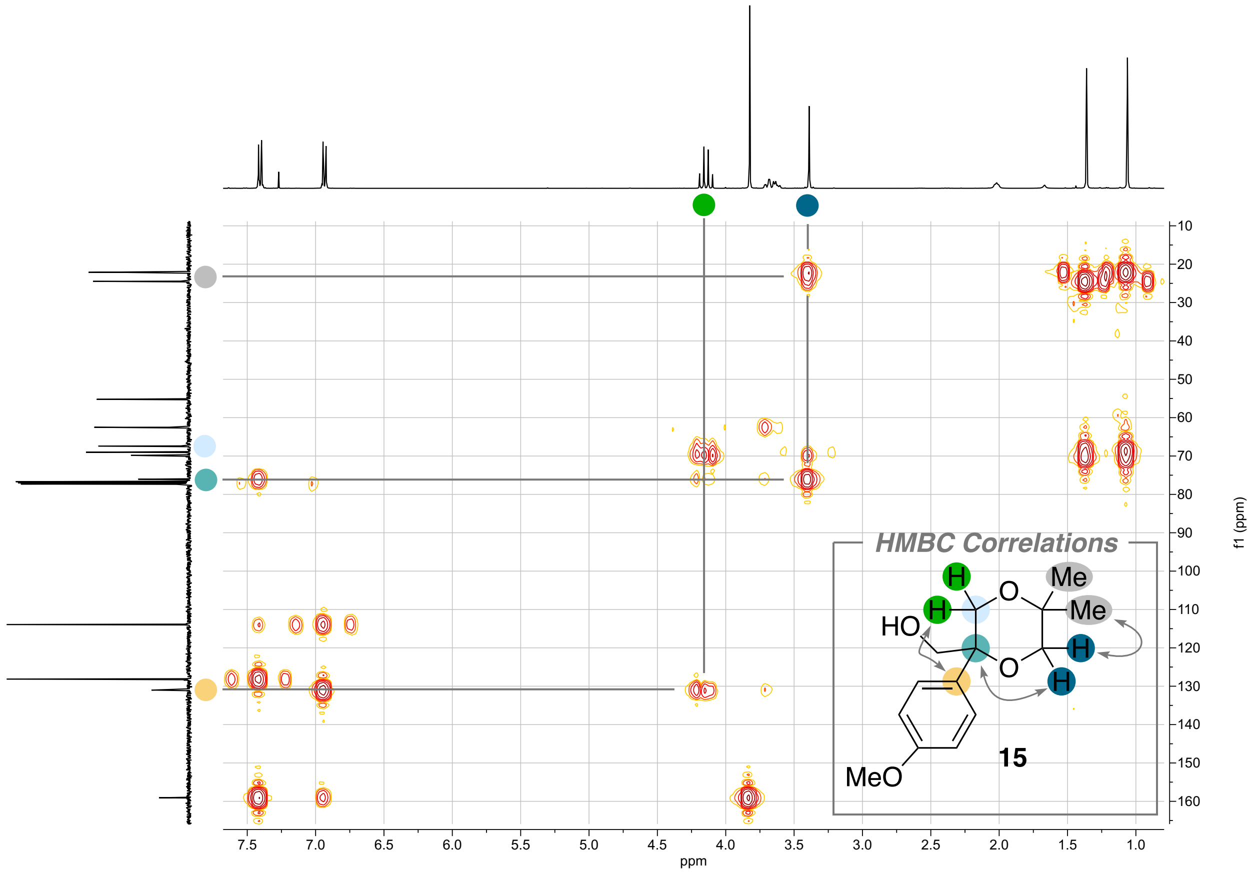

Fig. S3 HMBC spectrum (^{1}H: $CDCl_3$, 400 MHz; ^{13}C: $CDCl_3$, 101 MHz) and most relevant HMBC correlations of **15** (*inlet*).

1-(Hydroxymethyl)cyclohexan-1-ol (S12)

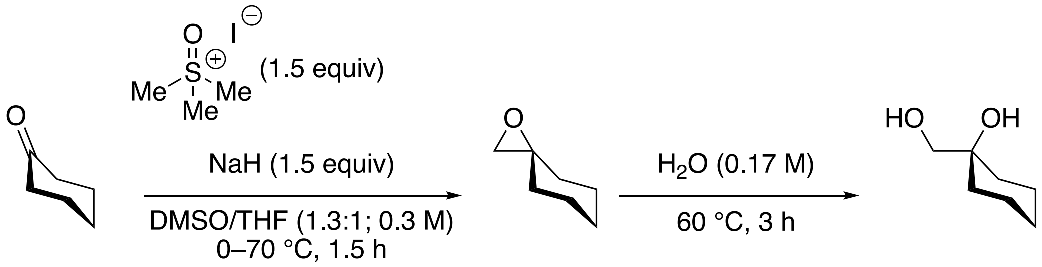

S12

NaH (600 mg, 15.0 mmol, 1.5 equiv, 60% dispersion in mineral oil) in anhydrous DMSO (10 mL) was heated to 70 °C in a 100 mL round bottom flask. After 20 min, the reaction mixture was diluted with anhydrous THF (10 mL) and then cooled to 0°C. A solution of trimethyl-sulfonium iodide (3.06 g, 15.0 mmol, 1.5 equiv) in anhydrous DMSO (10 mL) was added over a period of 5 min, followed by a solution of cyclohexanone (1.03 mL, 10.0 mmol, 1.0 equiv) in anhydrous THF (5 mL). The reaction was stirred at 25 °C for 1 h, then quenched with distilled water (50 mL). The aqueous layer was extracted with Et_2O (4 × 50 mL). The organic layer was washed with water (50 mL), brine (50 mL), dried over Na_2SO_4 and concentrated *in vacuo* using a rotatory evaporator to afford the epoxide intermediate as a pale yellow oil. The crude epoxide was taken up in distilled water (60 mL), stirred for 3 h at 60° C and then extracted with ethyl acetate (3 × 100 mL). The combined organic layers were concentrated *in vacuo* using a rotatory evaporator to afford diol **S12** as a white solid (780 mg, 60%). R_f = 0.33 (CH_2Cl_2/pentane/EtOAc 5:1:1); mp = 73–76 °C; IR (film)/cm^{-1} 3261 (br, OH), 2929, 2848, 1446, 1044; ^{1}H NMR (400 MHz, $CDCl_3$) δ 3.46 (s, 2 H, OCH_2), 2.06 (br s, 2 H, 2 × OH), 1.65–1.27 (m, 10 H, 5 × CH_2); ^{13}C NMR (101 MHz, $CDCl_3$) δ 71.7 (C_q), 70.2 (CH_2OH), 34.1 (2 × CH_2C_q), 25.9 (CH_2), 21.9 (2 × $CH_2CH_2C_q$). The observed characterization data (^{1}H, ^{13}C) were consistent with that previously reported.[13]

(3-(4-Methoxyphenyl)-1,4-dioxaspiro[5.5]undecan-3-yl)methanol (16)

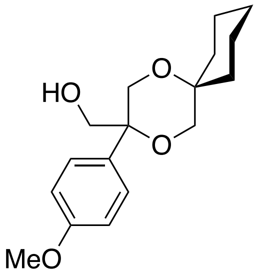

Prepared according to **General Procedure B** using cyclohexane diol **S12** (162.6 mg). Purification by flash column chromatography (50% EtOAc/pentane) afforded dioxane **16** as a white solid (40.0 mg, 55%). rr in crude reaction mixture: 90:10. R_f = 0.45 (50% EtOAc/pentane); mp = 110 °C; IR (film)/cm^{-1} 3448 (br, OH), 2930, 2859, 1513, 1245, 1074, 1029; ^{1}H NMR (400 MHz, $CDCl_3$) δ 7.43–7.39 (m, 2 H, 2 × Ar-CH), 6.95–6.91 (m, 2 H, 2 × Ar-CH), 4.15 (d, *J* = 12.7 Hz, 1 H, $C_qCHHOC_q(CH_2)_2$), 4.07 (1 H, d, *J* = 12.7 Hz, $C_qCHHOC_q(CH_2)_2$), 3.83 (s, 3 H, OCH_3), 3.68–3.65 (m, 2 H, CH_2OH), 3.49 (d, *J* = 11.5 Hz, 1 H, $C_q(CH_2)_2CHH$), 3.38 (d, *J* = 11.5 Hz, 1 H, $C_q(CH_2)_2CHH$), 2.09 (br s, 1 H, *C*HH), 1.93 (dd, *J* = 7.5, 5.6 Hz, 1 H, OH), 1.62–1.46 (m, 5 H, CH*H* + 2 × CH_2), 1.35–1.18 (m, 4 H, 2 × CH_2); ^{13}C NMR (101 MHz $CDCl_3$) δ 159.0 (Ar-C_qOMe), 131.3 (Ar-C_qC_q), 128.0 (2 × Ar-CH), 113.9 (2 × Ar-CH), 76.3 (Ar-C_qC_q), 70.4 ($C_q(CH_2)_2$), 68.5 ($C_q(CH_2)_2CH_2$), 67.4 (CH_2OH), 61.6 ($C_qCH_2OC_q(CH_2)_2$), 55.2 (OCH_3), 33.0 (CH_2), 30.4 (CH_2), 26.1 (CH_2), 21.4 (CH_2), 21.3 (CH_2); HRMS (ESI) *m/z* calcd for $C_{17}H_{25}O_4$ [M+H]: 293.1753, found: 293.1759.

Notes:

The regiochemistry of ***16*** *was determined with an HMBC correlation spectrum (see Fig. S4).*

Crystals suitable for X-ray crystallography were grown by slow evaporation from acetone and ***16*** *was further characterized by X-ray crystallography, confirming the regiochemistry (see Fig. S22).*

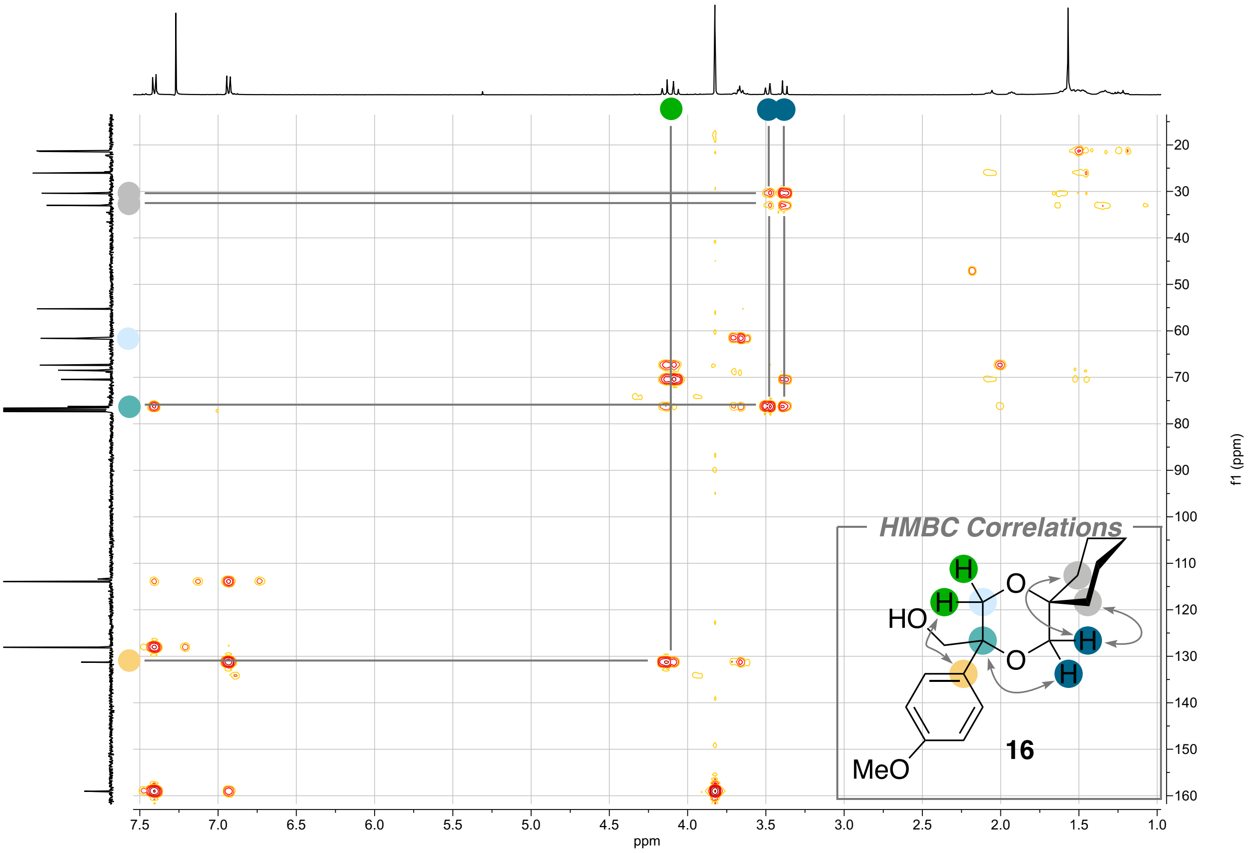

Fig. S4 HMBC spectrum (^{1}H: $CDCl_3$, 400 MHz; ^{13}C: $CDCl_3$, 101 MHz) and most relevant HMBC correlations of **16** (*inlet*).

1-(Hydroxymethyl)cyclopropan-1-ol (S13)[14]

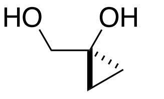

Lithium aluminium hydride (227.7 mg, 6.0 mmol, 1.5 equiv) was added portionwise to a solution of 1-hydroxycyclopropane-1-carboxylic acid (408.4 mg, 4.0 mmol, 1.0 equiv) in anhydrous THF (6.7 mL, 0.6 M) at 0 °C in a 25 mL round bottom flask. The reaction mixture was warmed to 25 °C and stirred for 7 h. Then, the reaction mixture was cooled to 0 °C, diluted with diethyl ether (10 mL) and distilled water (0.3 mL) was added slowly followed by 15% aq. NaOH (0.3 mL) and further distilled water (1.0 mL). The reaction mixture was warmed to 25 °C, stirred for 15 min, $MgSO_4$ was added, stirred for further 15 min, filtered and concentrated *in vacuo* using a rotatory evaporator. Purification by flash column chromatography (70–100% EtOAc/pentane) afforded oxetanol **S13** as a yellow oil (64 mg, 18%). R_f = 0.23 (100% EtOAc/pentane); IR (film)/cm^{-1} 3304 (br, OH), 2924, 1033; ^{1}H NMR (400 MHz, $CDCl_3$) δ 3.64 (s, 2 H, CH_2), 3.44 (br s, 1 H, OH), 2.76 (br s, 1 H, OH), 0.84 (br t, *J* = 6.2 Hz, 2 H, C*H*HC$_q$C*H*H), 0.58 (dd, *J* = 6.7, 5.3 Hz, 2 H, CH*H*C$_q$CH*H*); ^{13}C NMR (101 MHz, $CDCl_3$) δ 68.9 (CH_2OH), 56.6 (C$_q$), 11.6 (*C*H$_2$C$_q$*C*H$_2$). The observed characterization data (^{1}H) were consistent with that previously reported.[14]

Notes:

*Attempts to purify **S13** by distillation resulted in its degradation.*

(6-(4-Methoxyphenyl)-4,7-dioxaspiro[2.5]octan-6-yl)methanol (17) and **(5-(4-methoxyphenyl)-4,7-dioxaspiro[2.5]octan-5-yl)methanol (17')**

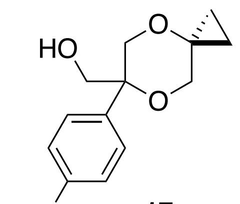
17

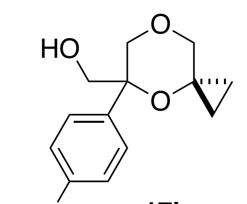
17'

Oxetanol **1a** (24.5 mg, 0.136 mmol, 1.0 equiv) was added to a solution of trifluoromethane sulfonimide (3.8 mg, 0.0136 mmol, 0.1 equiv) and cyclopropane diol **S13** (60.0 mg, 0.68 mmol, 5.0 equiv) in anhydrous MeCN (0.45 mL, 0.3 M) in a flame-dried reaction tube under Ar. The reaction mixture was stirred at 30 °C for 24 h and then quenched with sat. aq. $NaHCO_3$ (10 mL). The aqueous layer was extracted with CH_2Cl_2 (3 × 10 mL). The organic layers were combined, dried over Na_2SO_4, filtered and concentrated *in vacuo* using a rotatory evaporator. Purification by flash column chromatography (30% EtOAc/pentane) afforded an 82:18 mixture of dioxane **17** and dioxane **17'** as a colorless gum (27.8 mg, 82%). rr in crude reaction mixture: 82:18. R_f = 0.14 (30% EtOAc/pentane); IR (film)/cm^{-1} 3454 (br, OH), 3001, 2956, 1512, 1249, 1068; ^{1}H NMR (400 MHz, $CDCl_3$) δ 7.45–7.41 (m, 2 H, 2 × Ar-CH, **17**), 7.40–7.36 (m, 0.40 H, 2 × Ar-CH, **17'**), 6.98–6.94 (m, 2 H, 2 × Ar-CH, **17**), 6.92–6.89 (m, 0.39 H, 2 × Ar-CH, **17'**), 4.30 (d, *J* = 12.0 Hz, 1 H, C$_q$C*H*HOC$_{q\text{-(cyclopropane)}}$, **17**), 4.15 (d, *J* = 12.0 Hz, 1.18 H, C$_q$CH*H*OC$_{q\text{-(cyclopropane)}}$ (**17**) + C*H*H (**17'**)), 4.05 (d, *J* = 12.1 Hz, 0.21 H, C$_q$C*H*HOCH$_2$C$_{q\text{-(cyclopropane)}}$, **17'**), 3.98 (dd, *J* = 11.7, 1.6 Hz, 1.22 H, C$_{q\text{-(cyclopropane)}}$C*H*H (**17**) + CH*H* (**17'**)), 3.88 (d, *J* = 12.1 Hz, 0.21 H, C$_q$CH*H*OCH$_2$C$_{q\text{-(cyclopropane)}}$, **17'**), 3.83 (s, 3 H, OCH_3, **17**), 3.81 (s, 0.65 H, OCH_3, **17'**), 3.72 (dd, ^2J = 11.8 Hz, ^3J = 5.0 Hz, 1.23 H, C*H*HOH (**17**) + C*H*H (**17'**)), 3.64 (dd, ^2J = 11.8 Hz, ^3J = 8.1 Hz, 1 H, CH*H*OH, **17**), 3.54 (d, *J* = 11.5 Hz, 0.22 H, CH*H*, **17'**), 3.27 (d, *J* = 11.7 Hz, 1 H, C$_q$(CH$_2$)$_2$CH*H*, **17**), 2.03 (dd, *J* = 8.1, 5.0 Hz, 1 H, OH, **17**), 1.57 (dd, *J* = 6.4, 6.4 Hz, 0.21 H, OH, **17'**), 0.97–0.92 (m, 0.24 H, C*H*HC$_q$CH$_2$, **17'**), 0.88–0.78 (m, 2 H, C*H*HC$_q$C*H*H, **17**), 0.75–0.69 (m, 0.21 H, CH$_2$C$_q$C*H*H, **17'**), 0.67–0.62 (m, 1.19 H, CH*H*C$_q$CH$_2$ (**17**) + CH*H*C$_q$CH$_2$ (**17'**)), 0.52–0.46 (m, 1 H, CH$_2$C$_q$CH*H*, **17**), 0.44–0.40 (m, 0.18 H, CH$_2$C$_q$CH*H*, **17'**); ^{13}C NMR (101 MHz $CDCl_3$) δ 159.1 (Ar-C$_q$OMe, **17**), 130.8 (Ar-*C$_q$*C$_q$, **17**), 128.2 (2 × Ar-CH, **17**), 127.4 (2 × Ar-CH, **17'**), 114.1 (2 × Ar-CH, **17**), 113.7 (2 × Ar-CH, **17'**), 76.4 (Ar-C$_q$*C$_q$*, **17**), 71.9 (CH_2O, **17'**), 70.1 (CH_2O, **17'**), 68.6 (C$_q$*C*H$_2$OC$_q$(CH$_2$)$_2$, **17**), 67.6 (CH_2OH, **17**), 66.0 (C$_q$(CH$_2$)$_2$*C*H$_2$, **17**), 64.7 (CH_2O, **17'**), 58.4 (CH$_2$*C$_q$*CH$_2$, **17**), 55.3 (OCH_3, **17**), 54.3 (OCH_3, **17'**), 11.9 (*C*H$_2$C$_q$CH$_2$, **17'**), 11.3 (*C*H$_2$C$_q$CH$_2$, **17**), 9.7 (CH$_2$C$_q$*C*H$_2$, **17**), 9.6 (CH$_2$C$_q$*C*H$_2$, **17'**); HRMS (APCI) *m/z* calcd for $C_{14}H_{17}O_3^+$ $[M–OH]^+$: 233.1172, found: 233.1173.

Notes:

*The regiochemistry of **17** was determined with an HMBC correlation spectrum (see Fig. S5).*

*Signals in the ^{1}H and ^{13}C NMR spectra of the minor diastereomer **17’** were reported only when these were unambiguous. If unclear, these signals were not reported.*

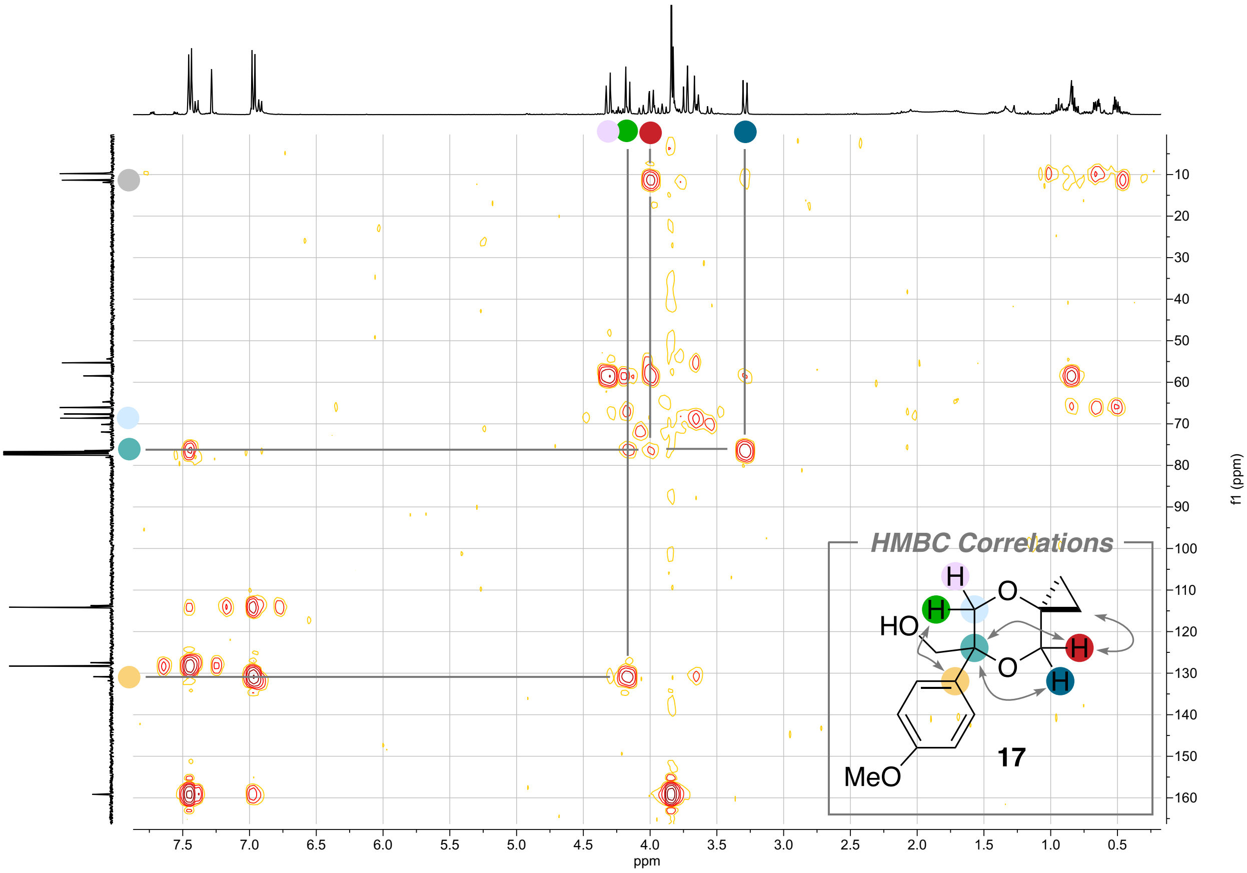

Fig. S5 HMBC spectrum (^{1}H: $CDCl_3$, 400 MHz; ^{13}C: $CDCl_3$, 101 MHz) and most relevant HMBC correlations of **17** (*inlet*).

((2*S*,5*R*,6*R*)-2-(4-Methoxyphenyl)-5,6-dimethyl-1,4-dioxan-2-yl)methanol (18) and **((2*R*,5*R*,6*R*)-2-(4-methoxyphenyl)-5,6-dimethyl-1,4-dioxan-2-yl)methanol (18’)**

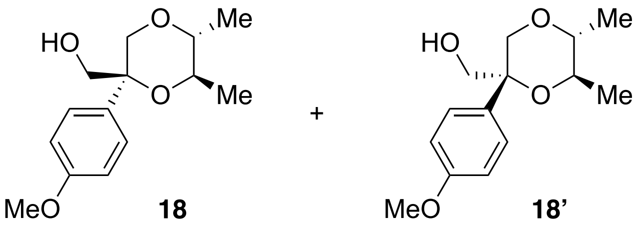

Prepared according to **General Procedure B** using (*R*,*R*)-butane-2,3-diol (0.11 mL). Purification by flash column chromatography (10% Et_2O/CH_2Cl_2) afforded a 90:10 diastereomeric mixture of dioxanes **18** and **18’** as white crystals (38.5 mg, 61%). dr in crude reaction mixture: 90:10. R_f = 0.14 (10% Et_2O/CH_2Cl_2); mp = 106–109 °C; IR (film)/cm^{-1} 3461 (br, OH), 2973, 1511, 1247, 1103; ^{1}H NMR (400 MHz, $CDCl_3$) δ 7.40–7.37 (m, 2.10 H, 2 × Ar-CH, **18** + **18’**), 6.95–6.90 (m, 2.10 H, 2 × Ar-CH, **18** + **18’**), 4.48 (d, *J* = 12.3 Hz, 1 H, $C_qCH_{eq}HOCMe$, **18**), 4.42 (d, *J* = 11.8 Hz, 0.09 H, $C_qCHHOCMe$, **18’**), 4.03 (d, *J* = 12.3 Hz, 1 H, $C_qCHH_{ax}OCMe$, **18**), 3.93 (d, *J* = 11.8 Hz, 0.13 H, $C_qCHHOCMe$, **18’**), 3.82 (s, 3 H, OCH_3, **18**), 3.80 (s, 0.31 H, OCH_3, **18’**), 3.59 (d, *J* = 11.7 Hz, 1 H, C_qCHHOH, **18**), 3.50 (d, *J* = 12.0 Hz, 0.10 H, C_qCHHOH, **18’**), 3.38–3.25 (m, 3 H, C_qCHHOH (*left*) + C_qOCH (*middle*) + CH_2OCH (*right*), **18**), 2.12 (br s, 1 H, OH, **18**), 1.23 (d, *J* = 6.2 Hz, 0.30 H, CH_3, **18’**), 1.19 (d, *J* = 6.2 Hz, 0.29 H, CH_3, **18’**), 1.10 (d, *J* = 5.9 Hz, 3 H, C_qOCHCH_3, **18**), 1.00 (d, *J* = 5.9 Hz, 3 H, CH_2OCHCH_3, **18**); ^{13}C NMR (101 MHz, $CDCl_3$) δ 158.9 (Ar-C_qOMe, **18**), 131.5 (Ar-C_qC_q, **18**), 128.3 (2 × Ar-CH, **18**), 126.5 (2 × Ar-CH, **18’**), 114.0 (2 × Ar-CH, **18**), 113.8 (2 × Ar-CH, **18’**), 77.3 (C_q + CH_2OCH, **18**), 71.2 (C_qOCH, **18**), 69.1 (CH_2OH, **18**), 68.2 (C_qCH_2OMe, **18**), 55.2 (OCH_3, **18**), 17.4 (C_qOCHCH_3, **18**), 17.1 (CH_2OCHCH_3, **18**); HRMS (APCI) *m/z* calcd for $C_{14}H_{19}O_3^+$ [M–OH]$^+$: 235.1329, found: 235.1329.

Notes:

The complex multiplet between 3.38–3.25 ppm in the ^{1}*H NMR spectrum, which is composed of a doublet* (C_qCHHOH) *and two doublets of quartets* (C_qOCH + CH_2OCH)*, could be further dissected into a left* (C_qCHHOH)*, middle* (C_qOCH) *and right part* (CH_2OCH) *with help of coupling constants and 2D correlation spectra (see Fig. S6). This was necessary to elucidate the relative stereochemistry of major diastereomer* ***18*** *using a NOESY spectrum (see Fig. S7). The determined stereochemistry was in accordance with other examples.*

18 *was further characterized by X-ray crystallography, confirming the stereochemistry (see Fig. S23). Assignment of the solved X-ray structure to the major diastereomer was performed by HPLC (see Fig. S8; NMR analysis was unsuitable due to the small size of the single crystal used for X-ray diffraction (0.41 µg)).*

The C_q *and* CH_2OCH *signals at 77.3 ppm in the* ^{13}*C NMR spectrum were identified with an HMBC correlation spectrum.*

Signals in the ^{1}*H and* ^{13}*C NMR spectra of the minor diastereomer* ***18'*** *were reported only when these were unambiguous. If unclear, these signals were not reported.*

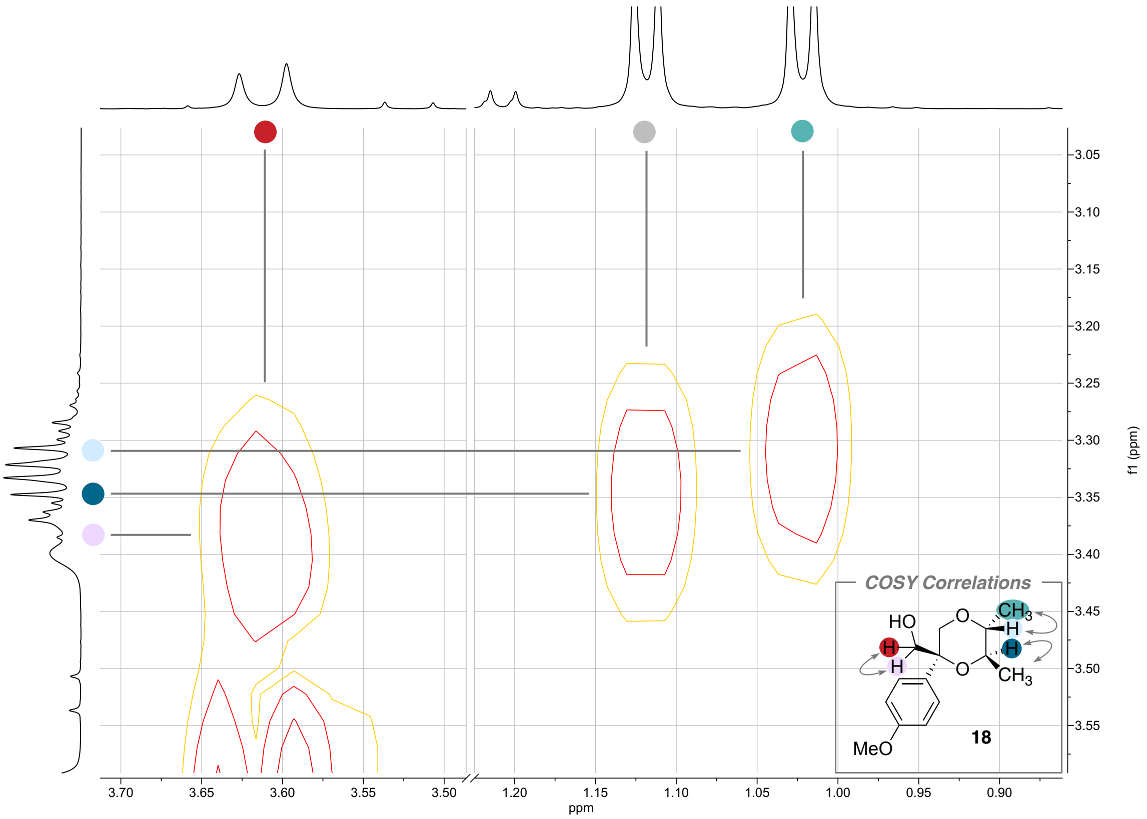

Fig. S6 Zoomed and cut COSY spectrum ($CDCl_3$, 400 MHz) and most relevant COSY correlations (*inlet*) as an example of the dissection of the multiplet between 3.38–3.25 ppm in the ^{1}H NMR spectrum. This was and can also be performed with HSQC and HMBC correlation spectra.

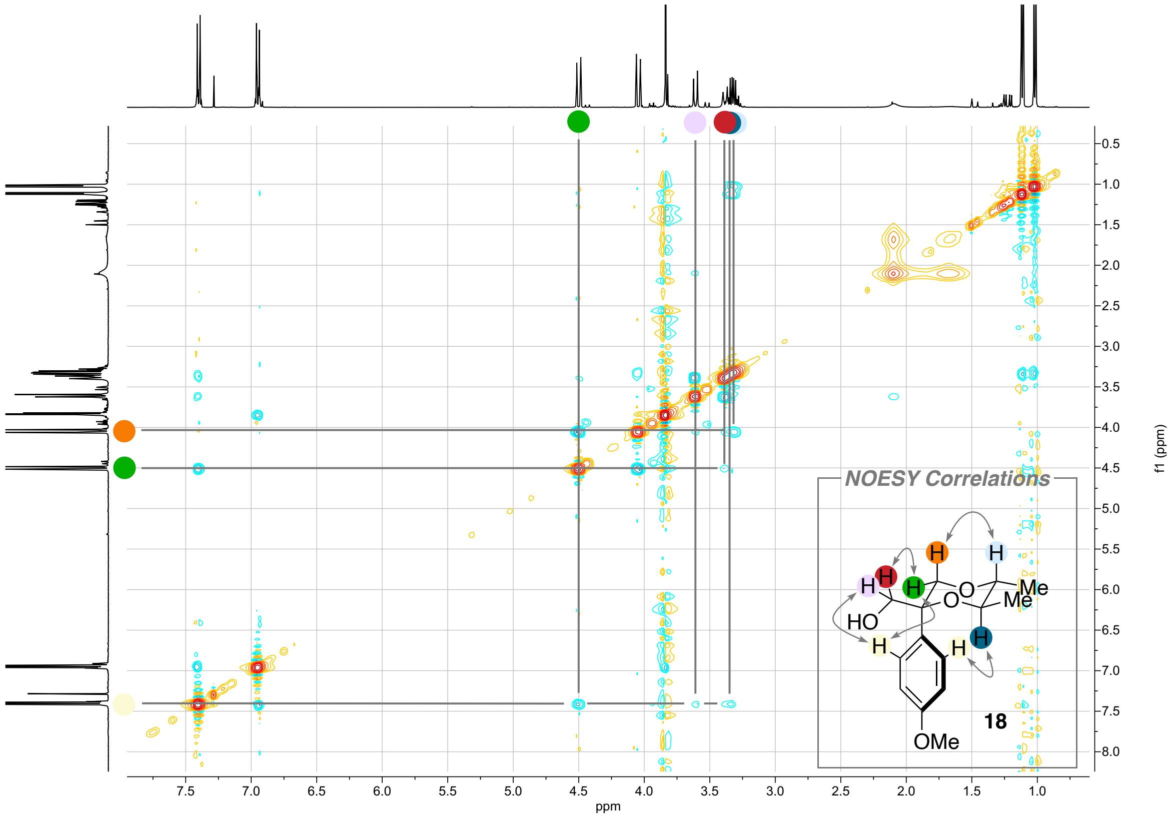

Fig. S7 NOESY spectrum ($CDCl_3$, 400 MHz) and most relevant NOESY correlations of **18** (*inlet*).

Diastereomeric mixture of 18 and 18’ (90:10 by ^{1}H NMR)

Conditions: Chiralpak IA 3-column, 95:5 *n*-hexane:*i*-PrOH, flow rate: 1 mL min^{-1}, 35 °C. UV detection wavelength: 270 nm.

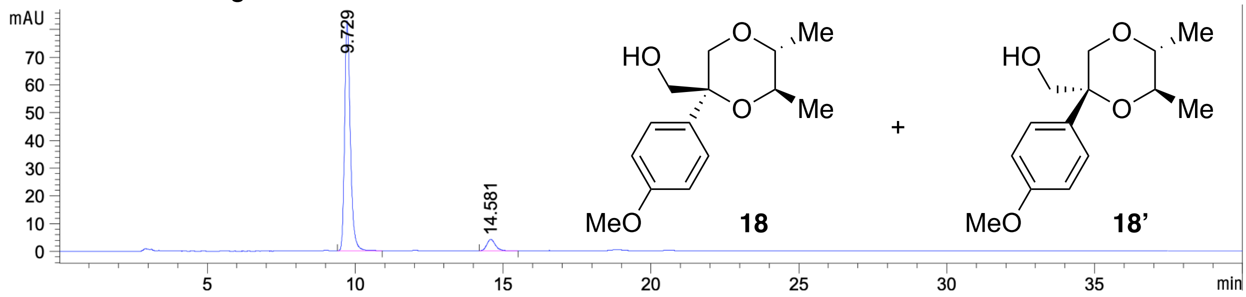

Peak #	RetTime [min]	Type	Width [min]	Area [mAU*s]	Height [mAU]	Area %
1	9.729	BB	0.2049	1116.80396	83.02229	93.0571
2	14.581	BB	0.3030	83.32367	4.13577	6.9429

((2*S*,5*R*,6*R*)-2-(4-Methoxyphenyl)-5,6-dimethyl-1,4-dioxan-2-yl)methanol (18) from single crystal used for X-ray analysis

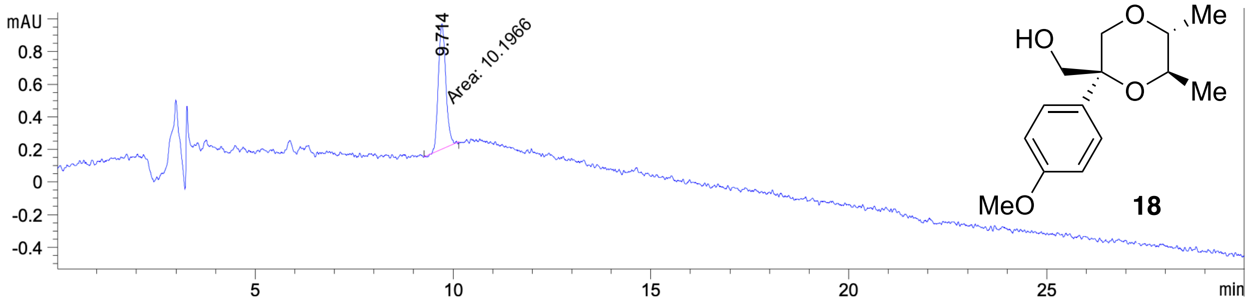

Peak #	RetTime [min]	Type	Width [min]	Area [mAU*s]	Height [mAU]	Area %
1	9.714	MM	0.2207	10.19659	7.69863e-1	100.0000

Fig. S8 HPLC trace of diastereomeric mixture (**18** + **18’**; *Top*) and single crystal (**18**, *Bottom*).

((2*S*,5*R*,6*R*)-2-(4-Methoxyphenyl)-5,6-diphenyl-1,4-dioxan-2-yl)methanol (19) and **((2*R*,5*R*,6*R*)-2-(4-methoxyphenyl)-5,6-diphenyl-1,4-dioxan-2-yl)methanol (19’)**

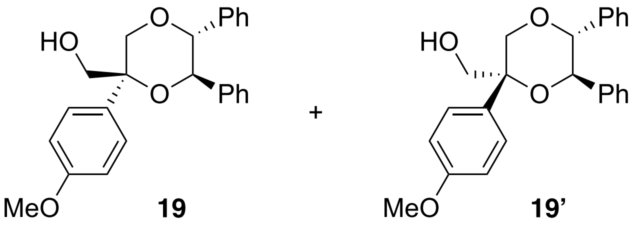

19 **19’**

Prepared according to **General Procedure B** using (*R*,*R*)-hydrobenzoin (267.8 mg). Purification by flash column chromatography (pentane/CH_2Cl_2/Et_2O 4:5:1) afforded a 94:6 diastereomeric mixture of dioxanes **19** and **19’** as a colorless gum (64.5 mg, 69%). dr in crude reaction mixture: 94:6. R_f = 0.19 (pentane/CH_2Cl_2/Et_2O 4:5:1); IR (film)/cm^{-1} 3399 (br, OH), 2937, 1607, 1513, 1245, 1148, 1066, 1025; ^{1}H NMR (400 MHz, $CDCl_3$) δ 7.58–7.52 (m, 2.12 H, 2 × $Ar_{(PMP)}$-CH, **19** + **19’**), 7.26–7.18 (m, 3 H, 3 × Ph-CH, **19**), 7.16–7.07 (m, 3 H, 3 × Ph-CH, **19**), 7.04–6.99 (m, 4 H, 2 × Ph-CH + 2 × $Ar_{(PMP)}$-CH, **19**), 6.84–6.82 (m, 2 H, 2 × Ph-CH, **19**), 4.96 (d, *J* = 9.3 Hz, 0.06 H, CH, **19’**), 4.80 (d, *J* = 12.4 Hz, 1.06 H, C_qCH_{eq}HOCPh (**19**) + CH (**19’**)), 4.51 (d, *J* = 9.3 Hz, 1 H, CH_2OCH, **19**), 4.46 (d, *J* = 9.3 Hz, 1 H, C_qOCH, **19**), 4.41 (d, *J* = 12.4 Hz, 1.05 H, C_qCHH_{ax}OCPh (**19**) + CH (**19’**)), 4.25 (d, *J* = 12.1 Hz, 0.07 H, C_qCH*H*OCPh, **19’**), 4.16 (dd, *J* = 11.8, 7.7 Hz, 0.07 H, C_qC*H*HOH, **19’**), 3.89 (s, 3 H, OCH_3,

19), 3.84 (s, 0.20 H, OCH_3, **19'**), 3.82 (dd, *J* = 11.9, 4.8 Hz, 1 H, C_qCHHOH, **19**), 3.56 (dd, *J* = 11.9, 9.0 Hz, 1 H, C_qCHHOH, **19**), 2.15 (dd, *J* = 9.0, 4.8 Hz, 1 H, OH, **19**), 1.69 (dd, *J* = 7.7, 4.5 Hz, 0.06 H, OH, **19'**); ^{13}C NMR (101 MHz, $CDCl_3$) δ 159.2 ($Ar_{(PMP)}$-C_qOMe), 137.7 (Ph-C_q), 137.5 (Ph-C_q), 130.8 ($Ar_{(PMP)}$-$C_q$$C_q$), 128.9 (2 × $Ar_{(PMP)}$-CH), 128.2 (Ph-CH), 128.0 (2 × Ph-CH), 127.9 (Ph-CH), 127.8 (2 × Ph-CH), 127.7 (2 × Ph-CH), 127.4 (2 × Ph-CH), 114.1 (2 × $Ar_{(PMP)}$-CH), 84.0 (CH_2OCH), 78.2 (C_qOCH), 77.7 (C_q), 69.2 (C_qCH_2OH), 68.3 (C_qCH_2OCPh), 55.3 (OCH_3); HRMS (APCI) *m/z* calcd for $C_{24}H_{23}O_3^+$ $[M–OH]^+$: 359.1642, found: 359.1634.

Notes:

*Signals in the ^{1}H NMR spectrum of the minor diastereomer **19'** were reported only when these were unambiguous. If unclear if, and how many protons of **19'** present, these signals were not reported. For example, in the aromatic region (7.30–6.90 ppm), it is evident from the observed integrals, that some of the peaks are a mixture of **19** and **19'**, but it is unclear how many protons of **19'** are present, also due to the residual $CHCl_3$ signal. In this case, only the peaks for **19** are reported.*

*Signals in the ^{1}H NMR spectrum of the minor diastereomer **19'** that could not be unambiguously assigned are reported as 'CH'.*

*No signals of **19'** were observed in the ^{13}C NMR spectrum.*

*The relative stereochemistry of major diastereomer **19** was determined by a NOESY correlation spectrum (see Fig. S9).*

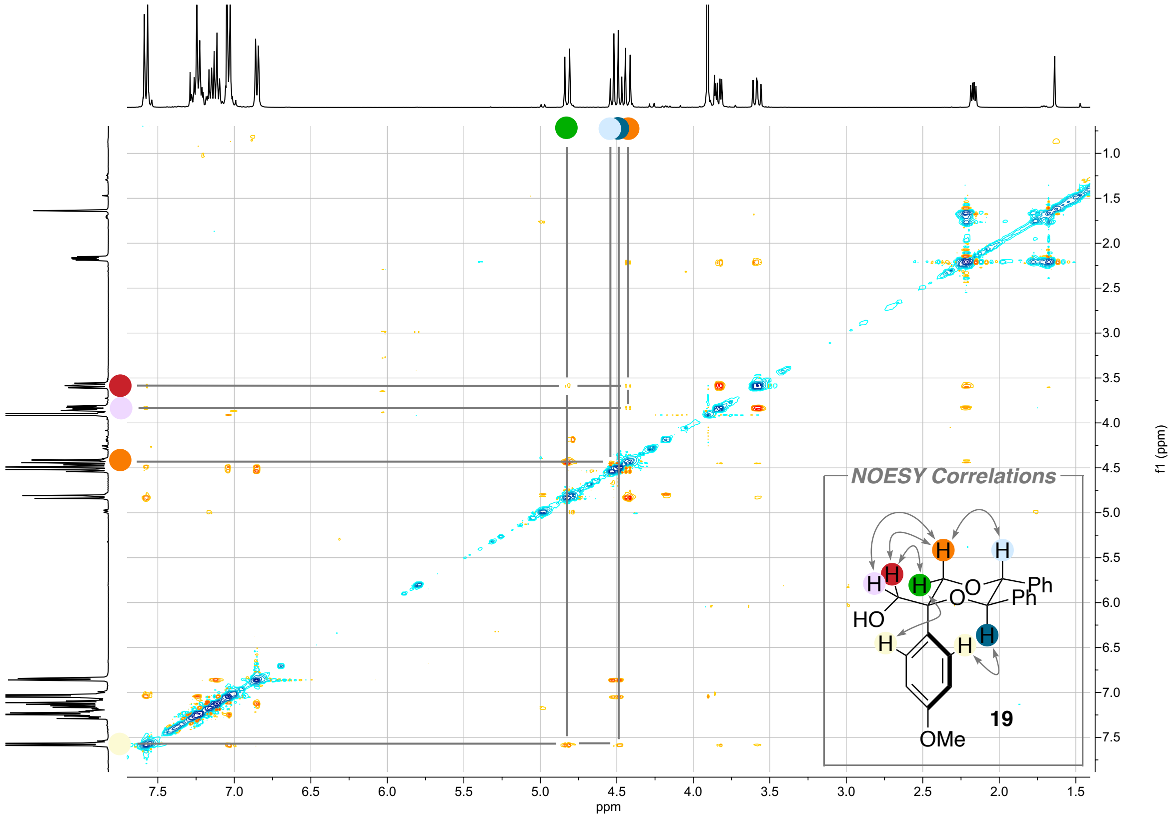

Fig. S9 NOESY spectrum ($CDCl_3$, 500 MHz) and most relevant NOESY correlations of **19** (inlet).

(2*R*,4a*S*,8a*S*)-(2-(4-Methoxyphenyl)octahydrobenzo[b][1,4]dioxin-2-yl)methanol ((–)-20)

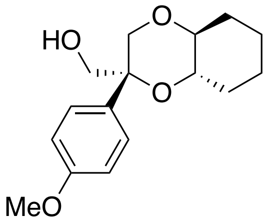

Prepared according to **General Procedure B** using (*S*,*S*)-cyclohexane-1,2-diol (145.2 mg). Purification by flash column chromatography (pentane/CH_2Cl_2/Et_2O 2:5:1) afforded a mixture of dioxanes **20** and **20'** as a colorless gum (18.6 mg, 27%, dr 79:21), followed by dioxane **20** as a colorless gum (42.5 mg). The diastereomeric mixture was repurified by flash column chromatography using the same solvent mixture to afford a mixture of dioxanes **20** and **20'** as a colorless gum (8.0 mg, 11%, dr 67:33), followed by dioxane **20** as a colorless gum (10.5 mg; total: 53.0 mg, 76%, er >99:1) dr in crude reaction mixture: 93:7. $[\alpha]_D^{21}$ – 36.0 (*c* 1.00, $CDCl_3$). R_f = 0.41 (pentane/CH_2Cl_2/Et_2O 2:6:1); IR (film)/cm^{-1} 3450 (br, OH), 2935, 1513, 1249, 1096; ^{1}H NMR (400 MHz, $CDCl_3$) δ 7.43–7.40 (m, 2 H, 2 × Ar-CH), 6.96–6.92 (m, 2 H, 2 × Ar-CH), 4.54 (d, *J* = 12.3 Hz, 1 H, CH_{eq}HOCH), 4.11 (d, *J* = 12.3 Hz, 1 H, CHH_{ax}OCH), 3.83 (s, 3 H, OCH_3), 3.61 (dd, ^2J = 11.7 Hz, ^3J = 4.2 Hz, 1 H, C*H*HOH), 3.37 (dd, ^2J = 11.7 Hz, ^3J = 9.2 Hz, 1 H, CH*H*OH), 3.24–3.16 (m, 2 H, 2 × CH), 2.00 (dd, *J* = 9.2, 4.2 Hz, 1 H, OH), 1.84–1.81 (m, 2 H, 2 × CHCH_{eq}H), 1.71–1.66 (m, 2 H, 2 × CH_2CH_{eq}H), 1.37–1.12 (4 H, m, 4 × CHH_{ax}); ^{13}C NMR (101 MHz, $CDCl_3$) δ 159.0 (Ar-C_qOMe), 131.6 (Ar-$C_q$$C_q$), 128.4 (2 × Ar-CH), 114.0 (2 × Ar-CH), 80.3 (CH_2O*C*H), 77.8 (C_q), 73.9 (C_qO*C*H), 69.1 (*C*H_2OCH), 68.6 (CH_2OH), 55.2 (OCH_3), 30.2 (C_qOCH*C*H_2), 29.8 (CH_2OCH*C*H_2), 24.1 (2 × $CHCH_2$*C*H_2); HRMS (APCI) *m/z* calcd for $C_{16}H_{21}O_3^+$ $[M–OH]^+$: 261.1485, found: 261.1484.

HPLC Conditions: Chiralpak IA 3-column, 90:10 *n*-hexane:*i*-PrOH, flow rate: 1 mL·min^{-1}, 35 ºC. UV detection wavelength: 270 nm. Retention times: 7.8 min (2*S*,4a*S*,8a*S* enantiomer), 9.1 min (2*R*,4a*S*,8a*S* enantiomer).

Notes:

*The relative stereochemistry of major diastereomer **20** was determined by a NOESY correlation spectrum (see Fig. S10).*

Commercial (S,S)-cyclohexane-1,2-diol was purified by filtration through a plug of silica before use. There was no reactivity with the unpurified commercial diol. Presumably, an NMR-silent basic impurity in the commercial material poisoned the Brønsted acid catalyst (2% of an impurity is enough to quench all catalyst).

*Under **General Conditions A** and (±)-trans-1,2-cyclohexanediol, (±)-**20** was isolated in 41% yield (28.2 mg) alongside minor diastereomer **20'** in 6% yield (colorless gum, 4.5 mg). dr in crude reaction mixture: 80:20.*

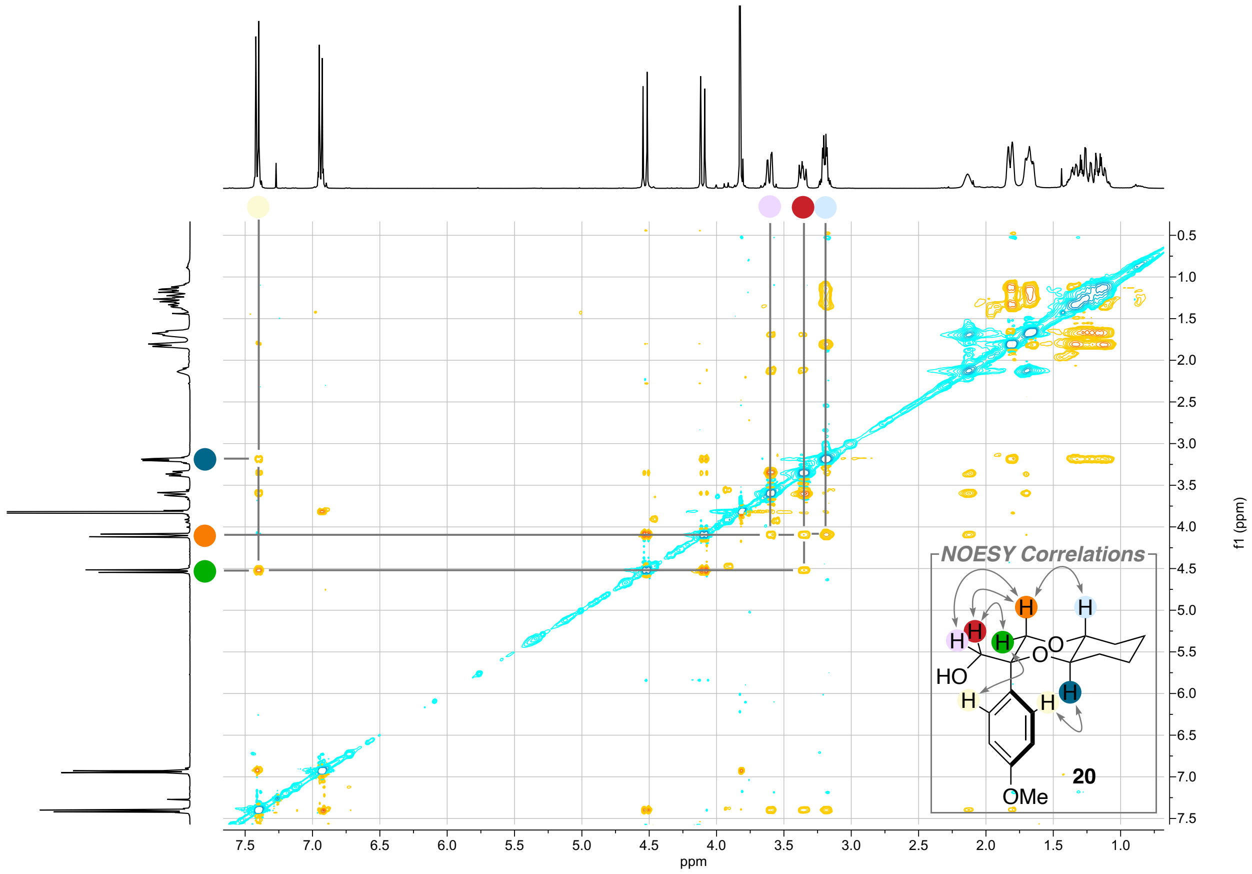

Fig. S10 NOESY spectrum ($CDCl_3$, 400 MHz) and most relevant NOESY correlations of **20** (inlet).

***all-trans*-(2-(4-Methoxyphenyl)octahydrobenzo[b][1,4]dioxin-2-yl)methanol (20')**

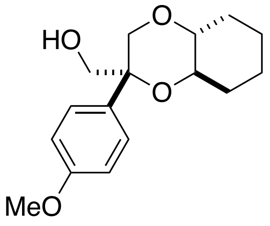

Prepared according to **General Procedure A** using oxetanol **1a** (45.1 mg) and (±)-*trans*-1,2-cyclohexanediol (145.2 mg). Purification by flash column chromatography (pentane/CH_2Cl_2/Et_2O 2:5:1) afforded dioxane **20'** as a colorless gum (2.4 mg), followed by a mixture of **20'** and **20** (13.6 mg) and by dioxane **20** as a pale pink gum (24.3 mg). Repurification of the mixture by flash column chromatography (pentane/CH_2Cl_2/Et_2O 2:5:1) afforded **20'** (2.1 mg) followed by **20** (3.9 mg) to give a total of 4.5 mg **20'** (6% yield) and 28.2 mg **20** (41% yield). dr in crude reaction mixture: 80:20. R_f = 0.45 (pentane/CH_2Cl_2/Et_2O 1:6:2); IR (film)/cm^{-1} 3434 (br, OH), 2937, 1514, 1249, 1108, 1081; ^{1}H NMR (400 MHz, $CDCl_3$) δ 7.41–7.37 (m, 2 H, 2 × Ar-CH), 6.93–6.89 (m, 2 H, 2 × Ar-CH), 4.50 (dd, ^2J = 11.7 Hz, ^3J = 4.1 Hz, 1 H, C*H*HOH), 3.93 (d, *J* = 12.0 Hz, 1 H, CH_{eq}HOCH), 3.91 (d, *J* = 11.7 Hz, 1 H, CH*H*OH), 3.81 (s, 3 H, OCH_3), 3.65 (ddd, $J_{ax,ax}$ = 10.8, 9.0 Hz, $J_{ax,eq}$ = 4.2 Hz, 1 H, C_qOCH), 3.58 (d, *J* = 12.0 Hz, 1 H, CHH_{ax}OCH), 3.15 (ddd, $J_{ax,ax}$ = 10.8, 9.0 Hz, $J_{ax,eq}$ = 4.2 Hz, 1 H, CH_2OC*H*), 1.99–1.92 (m, 2 H, 2 × CHCH_{eq}H), 1.80–1.78 (m, 2 H, 2 × CH_2CH_{eq}H), 1.47–1.26 (m, 4 H, 4 × H_{ax}); ^{13}C NMR (101 MHz $CDCl_3$) δ 159.0 (Ar-C_qOMe), 132.6 (Ar-$C_q$$C_q$), 126.5 (2 × Ar-CH), 113.8 (2 × Ar-CH), 80.3 (CH_2OCH), 76.4 (C_q), 73.6 (C_qOCH), 72.3 (CH_2OCH), 62.0 (CH_2OH), 55.3 (OCH_3), 30.7 (C_qOCHCH_2), 30.1 (CH_2OCHCH_2), 24.3 (CH$CH_2$$CH_2$), 24.2 ($CHCH_2CH_2$); HRMS (APCI) *m/z* calcd for $C_{16}H_{21}O_3^+$ $[M–OH]^+$: 261.1485, found: 261.1485.

Notes:

The doublets at 3.93 and 3.91 ppm in the ^{1}H NMR spectrum were integrated together to 2 H.

*The O–H signal was confirmed to be under the water peak and the relative stereochemistry of minor diastereomer **20'** was determined by a NOESY correlation spectrum (see Fig. S11).*

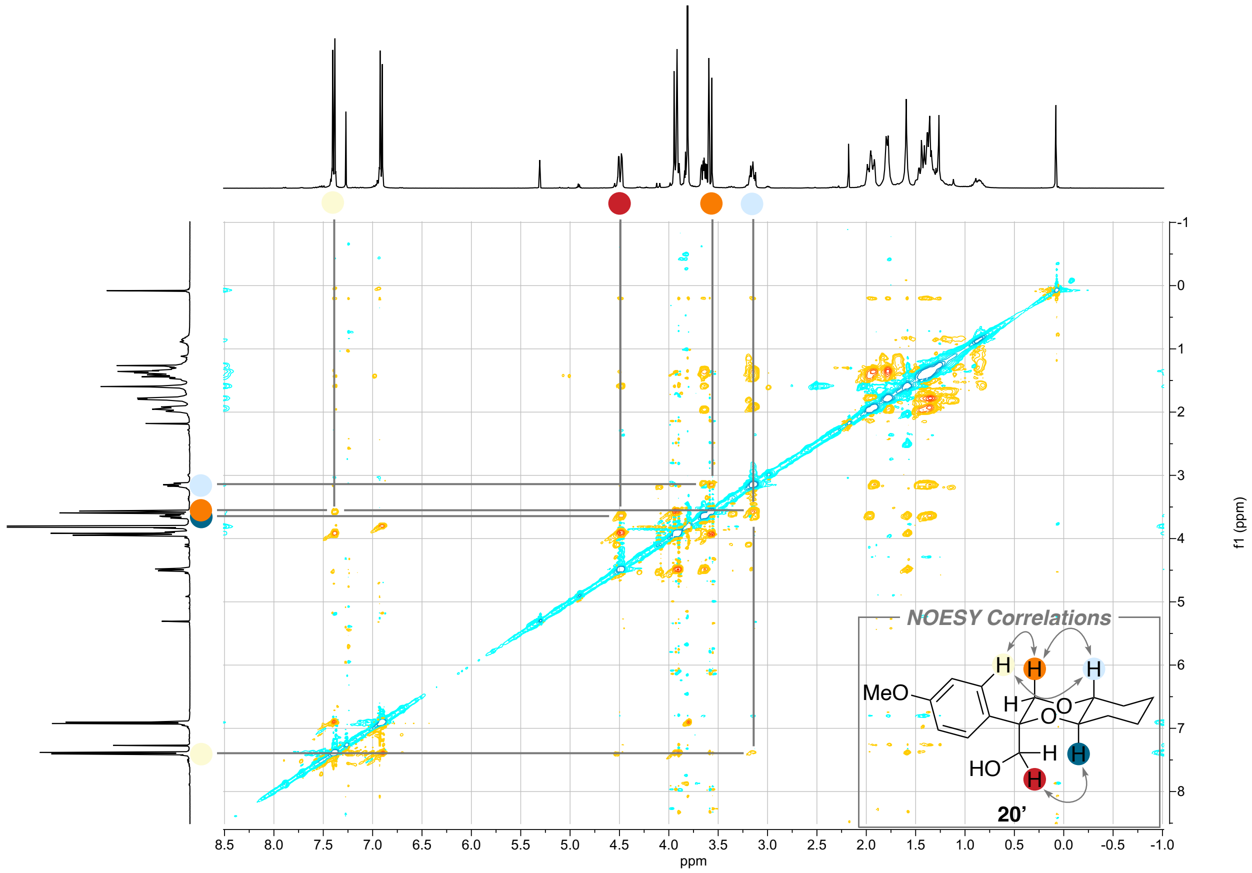

Fig. S11 NOESY spectrum ($CDCl_3$, 400 MHz) and most relevant NOESY correlations of **20'** (inlet).

***all-cis*-(2-(4-Methoxyphenyl)octahydrobenzo[b][1,4]dioxin-2-yl)methanol (21)** and ***cis,trans*-(2-(4-Methoxyphenyl)octahydrobenzo[b][1,4]dioxin-2-yl)methanol (21')**

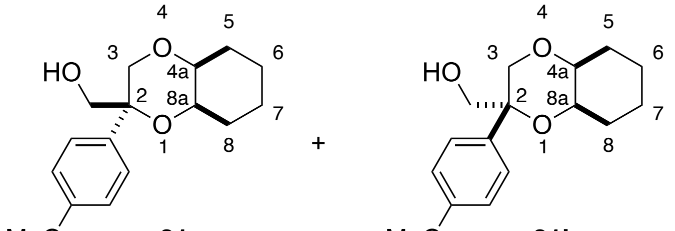

21 + **21'**

Prepared according to **General Procedure A** using oxetanol **1a** (45.1 mg) and *cis*-1,2-cyclohexanediol (145.2 mg). Purification by flash column chromatography (10–50% EtOAc/pentane) afforded dioxane **21** as white crystals (38.6 mg, 55%), followed by dioxane **21'** as a white solid (13.3 mg, 19%). dr in crude reaction mixture: 70:30.

Dioxane **21**: R_f = 0.47 (50% EtOAc/pentane); mp = 105 °C; IR (film)/cm^{-1} 3442 (br, OH), 2934, 1511, 1249, 1074, 1010, 830; ^{1}H NMR (400 MHz, $CDCl_3$) δ 7.42–7.38 (m, 2 H, 2 × Ar-CH), 6.96–6.92 (m, 2 H, 2 × Ar-CH), 4.32 (d, J = 12.6 Hz, 1 H, $H3_{ax}$), 4.13 (d, J = 12.6 Hz, 1 H, $H3_{eq}$), 3.83–3.82 (m, 1 H, H8a), 3.82 (s, 3 H, OCH_3), 3.65 (dd, ^2J = 11.8 Hz, ^3J = 2.8 Hz, 1 H, C*H*HOH), 3.46 (ddd, $J_{ax,ax}$ = 12.5 Hz, $J_{ax,eq}$ = 5.0, 3.2 Hz, 1 H, H4a), 3.37 (dd, ^2J = 11.8 Hz, ^3J = 9.1 Hz, 1 H, CH*H*OH), 2.27 (dddd, ^2J = 12.5 Hz, $J_{ax,ax}$ = 12.5, 12.5 Hz, $J_{ax,eq}$ = 3.8 Hz, 1 H, $H5_{ax}$), 2.17 (dd, J = 9.2, 2.8 Hz, 1 H, OH), 1.87–1.82 (m, 2 H, $H8_{eq}$ + $H6_{eq}$), 1.61–1.57 (m, 1 H, $H5_{eq}$), 1.51–1.36 (m, 3 H, $H8_{ax}$ + 2 × H7), 1.27–1.16 (m, 1 H, $H6_{ax}$); ^{13}C NMR (101 MHz $CDCl_3$) δ 159.1 (Ar-C_qOMe), 131.5 (Ar-$C_q$$C_q$), 128.3 (2 × Ar-CH), 114.0 (2 × Ar-CH), 77.6 (C_q), 72.3 (C4a), 69.4 (C8a), 67.1 (C3), 59.8 (CH_2OH), 55.2 (OCH_3), 30.6 (CH_2), 24.3 (CH_2), 23.7 (CH_2), 20.2 (CH_2); HRMS (ESI) *m/z* calcd for $C_{16}H_{21}O_3$ [M–OH]: 261.1491, found: 261.1502.

Dioxane **21'**: R_f = 0.31 (50% EtOAc/pentane); mp = 80–85 °C; IR (film)/cm^{-1} 3435 (br, OH), 2937, 1513, 1247, 1105, 1006; ^{1}H NMR (400 MHz, $CDCl_3$) δ 7.45–7.41 (m, 2 H, 2 × Ar-CH), 6.94–6.90 (m, 2 H, 2 × Ar-CH), 4.28 (d, J = 11.8 Hz, 1 H, C*H*HOH), 4.19–4.17 (m, 1 H, H8a), 3.94 (d, J = 12.4 Hz, 1 H, $H3_{ax}$), 3.82–3.79 (m, 1 H, CH*H*OH), 3.82 (s, 3 H, OCH_3), 3.70 (ddd, $J_{ax,ax}$ = 10.5 Hz, $J_{ax,eq}$ = 4.7,

3.2 Hz, 1 H, H4a), 3.63 (d, J = 12.4 Hz, 1 H, $H3_{eq}$), 2.15 (dddd, ^2J = 14.4 Hz, $J_{ax,ax}$ = 10.8, 10.5 Hz, $J_{ax,eq}$ = 3.9 Hz, 1 H, $H5_{ax}$), 1.86–1.82 (m, 1 H, $H8_{eq}$), 1.72–1.65 (m, 2 H, OH + $H6_{eq}$), 1.60–1.47 (m, 3 H, $H8_{ax}$ + $H5_{eq}$ + $H7_{eq}$), 1.40–1.36 (m, 1 H, $H7_{ax}$), 1.32–1.23 (m, 1 H, $H6_{ax}$); ^{13}C NMR (101 MHz $CDCl_3$) δ 159.0 (Ar-C_qOMe), 133.7 (Ar-C_qC_q), 126.7 (2 × Ar-CH), 113.8 (2 × Ar-CH), 75.5 (C_q), 72.7 (C4a), 68.0 (C8a), 64.6 (C3), 63.1 (CH_2OH), 55.2 (OCH_3), 30.6 (CH_2), 24.5 (CH_2), 23.6 (CH_2), 20.9 (CH_2); FTMS (APCI) *m/z* calcd for $C_{16}H_{23}O_4^+$ $[M+H]^+$: 279.1591, found: 279.1596.

Notes:

*The multiplet between 3.83–3.82 (1 H) and singlet at 3.82 ppm (3 H) in the ^{1}H NMR spectrum of **21** were integrated together to 4 H. The multiplet between 3.82–3.79 (1 H) and singlet at 3.82 ppm (3 H) in the ^{1}H NMR spectrum of **21'** were integrated together to 4 H.*

*X-ray quality crystals of **21'** were grown from slow evaporation from acetone. **21** and **21'** were further characterized by X-ray crystallography, confirming the stereochemistry of both the major and minor diastereomers (see Fig. S24 and S25).*

*The relative stereochemistry of both diastereomers **21** and **21'** were determined by NOESY correlation spectra (see Fig. S12 and S13).*

*Under **General Conditions B**, **21** was isolated in 44% yield (30.6 mg) alongside minor diastereomer **21'** in 4% yield (2.8 mg). dr in crude reaction mixture: 75:25.*

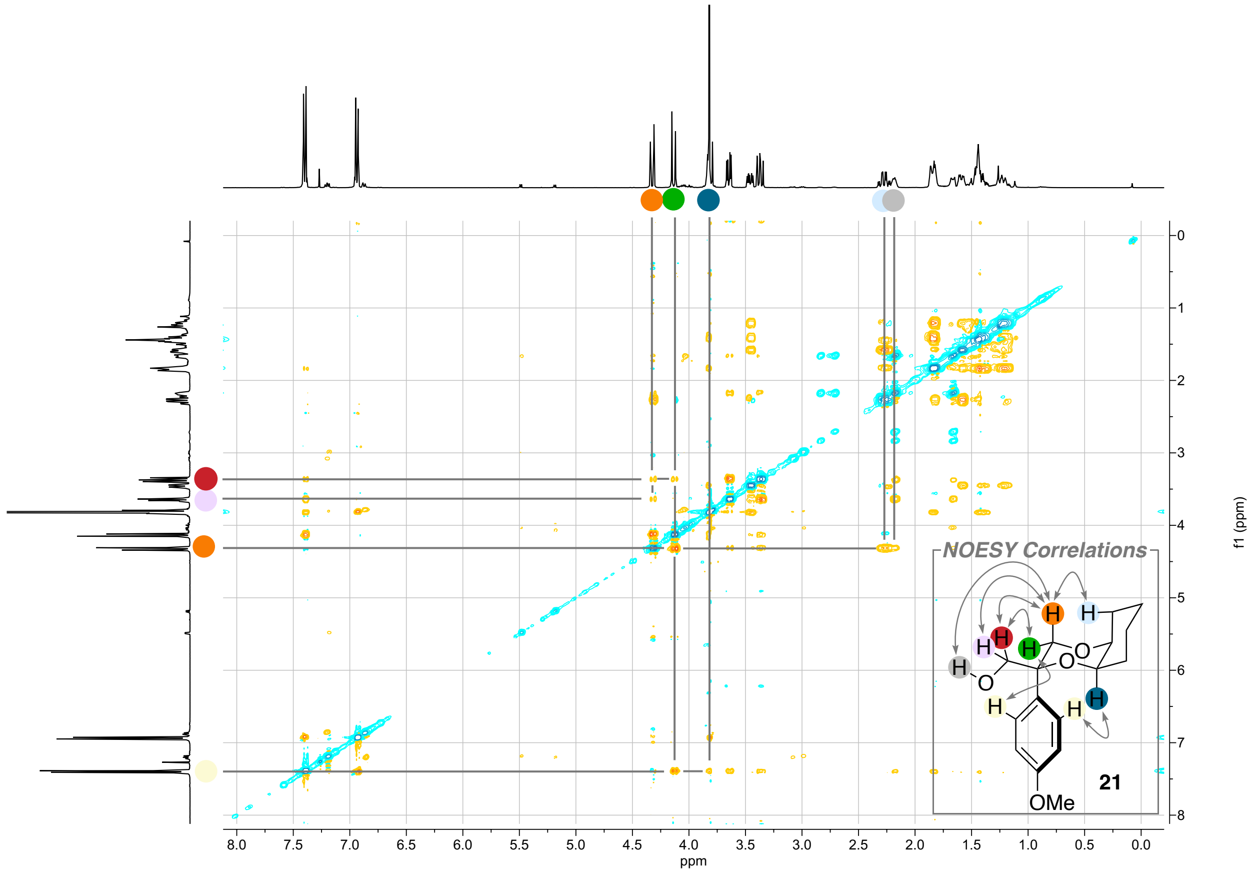

Fig. S12 NOESY spectrum ($CDCl_3$, 400 MHz) and most relevant NOESY correlations of **21** (inlet).

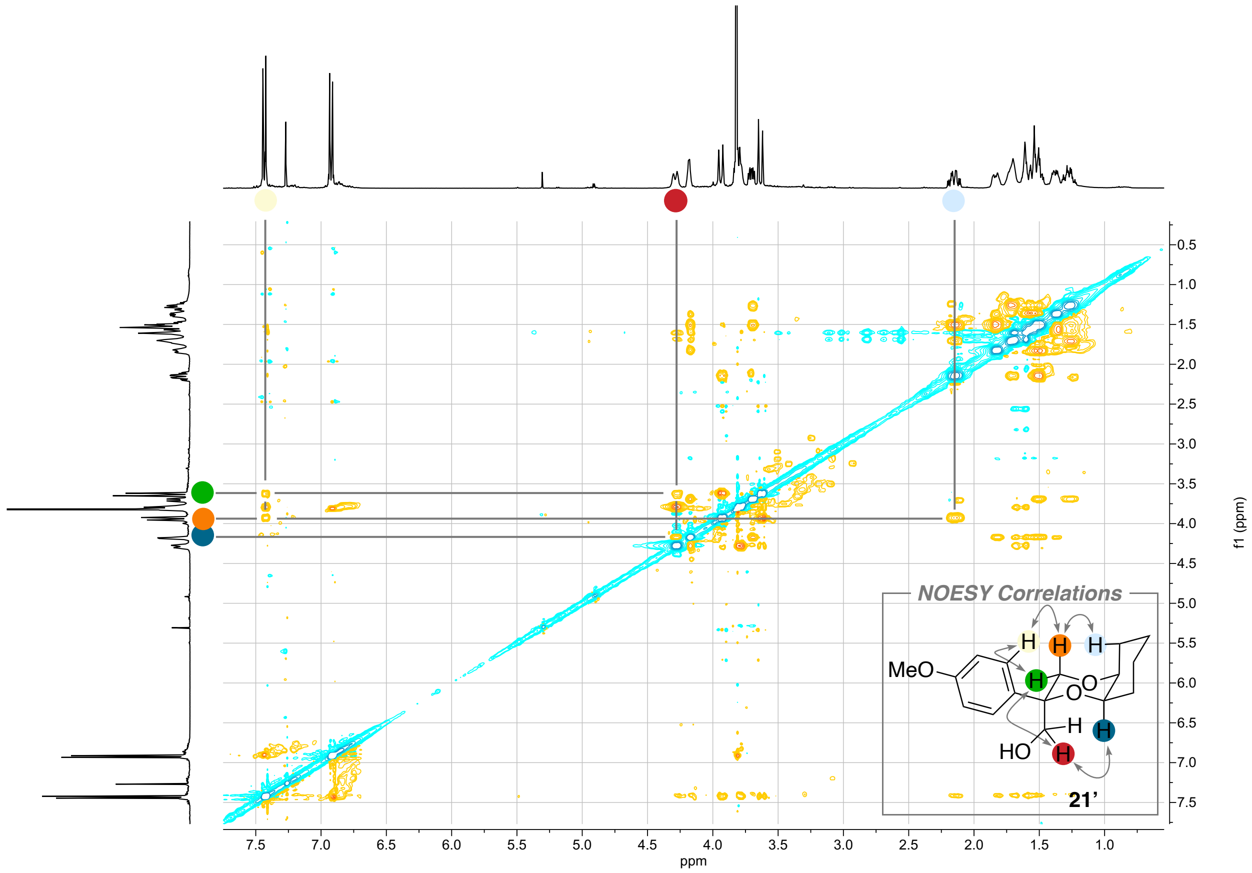

Fig. S13 NOESY spectrum ($CDCl_3$, 400 MHz) and most relevant NOESY correlations of **21'** (inlet).

***all-cis*-(2-(4-Methoxyphenyl)hexahydro-5*H*-cyclopenta[*b*][1,4]dioxin-2-yl)methanol (22)**

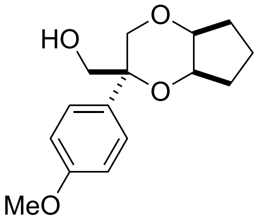

Prepared according to **General Procedure B** using *cis*-cyclopentane-1,2-diol (127.7 mg). Purification by flash column chromatography (pentane/CH_2Cl_2/Et_2O 3:5:2) afforded dioxane **22** as a colorless gum (40.2 mg, 61%), followed by a 26:74 mixture of dioxane **22** and its minor diastereomer as a colorless gum (6.3 mg, 10%; not characterized). dr in crude reaction mixture: 81:19. R_f = 0.33 (pentane/CH_2Cl_2/Et_2O 3:5:2); IR (film)/cm^{-1} 3451 (br, OH), 2955, 1510, 1247, 1129; ^{1}H NMR (400 MHz, $CDCl_3$) δ 7.34–7.30 (m, 2 H, 2 × Ar-CH), 6.95–6.91 (m, 2 H, 2 × Ar-CH), 4.26 (d, *J* = 12.1 Hz, 1 H, $C_qCH_{ax}HOCH$), 3.89–3.84 (m, 2 H, C_qOCH (*left*) + CH_2OCH (*middle*)), 3.86 (d, *J* = 12.1 Hz, 1 H, $C_qCHH_{eq}OCH$), 3.82 (s, 3 H, OCH_3), 3.64 (dd, *J* = 11.4, 4.9 Hz, 1 H, C*H*HOH), 3.50 (dd, *J* = 11.4, 8.1 Hz, 1 H, CH*H*OH), 2.17 (dd, *J* = 8.1, 4.9 Hz, 1 H, OH), 2.07–1.98 (m, 1 H, CH_2OCHC*H*H), 1.97–1.88 (m, 1 H, CH_2C*H*HCH_2), 1.86–1.73 (m, 3 H, CH_2OCHCH*H* + C_qOCHC*H_2*), 1.58–1.48 (m, 1 H, CH_2CH*H*CH_2); ^{13}C NMR (101 MHz, $CDCl_3$) δ 159.0 (Ar-C_qOMe), 131.8 (Ar-*C_q*C_q), 127.6 (2 × Ar-CH), 113.9 (2 × Ar-CH), 77.7 (CH_2O*C*H), 76.0 (C_q), 70.6 (C_qO*C*H), 70.3 (CH_2OH), 64.1 (C_qCH_2OCH), 55.2 (OCH_3), 31.4 (C_qOCH*CH_2*), 27.4 (CH_2OCH*CH_2*), 21.1 (CH_2*CH_2*CH_2); HRMS (ESI) *m/z* calcd for $C_{15}H_{21}O_4$ [M+H]: 265.1440, found: 265.1432.

Notes:

The doublet at 3.86 ppm (1 H) and multiplet at 3.89–3.84 ppm (2 H) in the ^{1}H NMR spectrum were integrated together to 3 H. This multiplet could be further dissected into a left (C_qOCH), middle (CH_2OCH) and right part ($C_qCHH_{eq}OCH$) with help of coupling constants and 2D correlation spectra (see Fig. S14 for further details). This was necessary to elucidate the relative stereochemistry of major diastereomer ***22*** *using a NOESY spectrum (see Fig. S15). The determined stereochemistry was in accordance with other examples.*

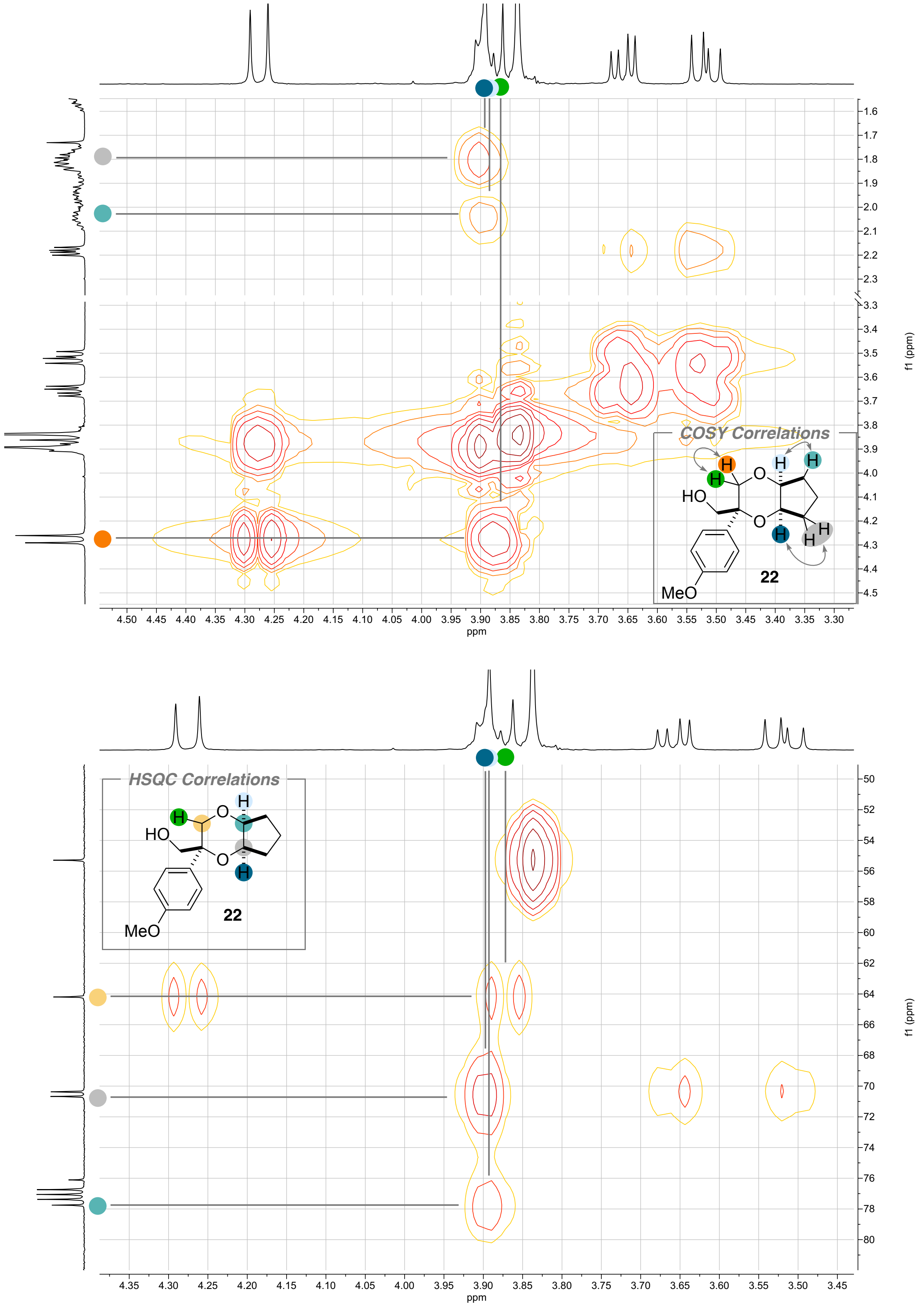

Fig. S14 *Top*: Zoomed and cut COSY spectrum ($CDCl_3$, 400 MHz) and most relevant COSY correlations (*inlet*) for the dissection of the multiplet between 3.89–3.84 ppm. *Bottom*: Zoomed HSQC spectrum ($CDCl_3$, 400 MHz) and most relevant HSQC correlations (*inlet*) for the dissection of the multiplet between 3.89–3.84 ppm.

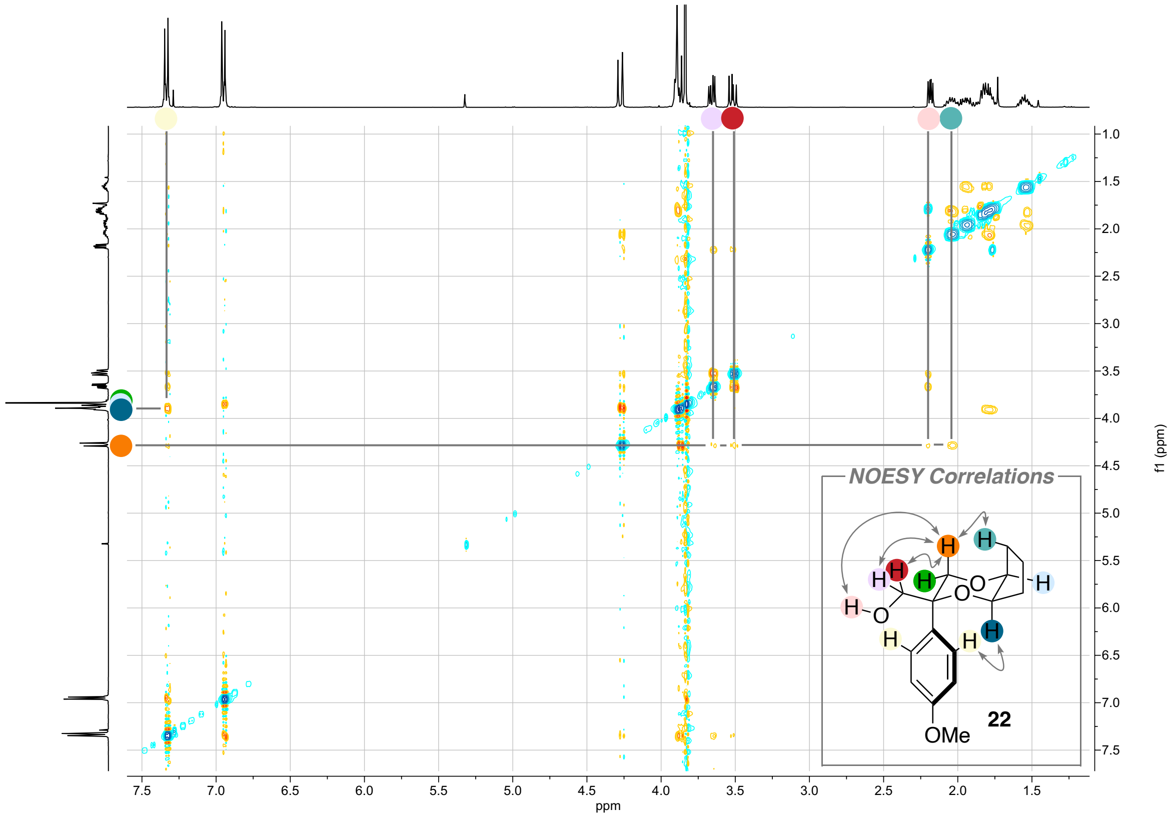

Fig. S15 NOESY spectrum ($CDCl_3$, 500 MHz) and most relevant NOESY correlations of **22** (*inlet*).

***trans-cis*-(2-(4-Methoxyphenyl)hexahydro-5*H*-cyclopenta[*b*][1,4]dioxin-2-yl)methanol (23)** and ***all-trans*-(2-(4-methoxyphenyl)hexahydro-5*H*-cyclopenta[*b*][1,4]dioxin-2-yl)methanol (23')**

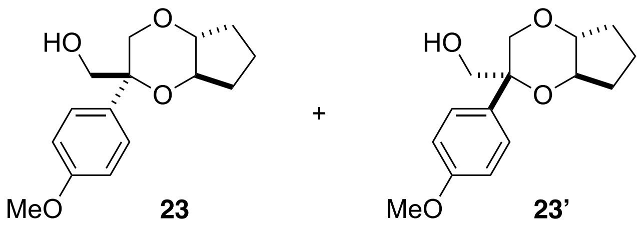

Prepared according to **General Procedure B** using (±)-*trans*-cyclopentane-1,2-diol (127.7 mg). Purification by flash column chromatography (pentane/CH_2Cl_2/Et_2O 3:5:2) afforded a 80:20 diastereomeric mixture of dioxanes **23** and **23'** as a colorless gum (46.1 mg, 70%). dr in crude reaction mixture: 80:20. R_f = 0.24 (pentane/CH_2Cl_2/Et_2O 3:5:2); IR (film)/cm^{-1} 3444 (br, OH), 2953, 1511, 1248, 1125, 1023; ^{1}H NMR (400 MHz, $CDCl_3$) δ 7.46–7.39 (m, 2.39 H, 2 × Ar-CH, **23** + **23'**), 6.96–6.90 (m, 2.36 H, 2 × Ar-CH, **23** + **23'**), 4.59 (d, *J* = 12.6 Hz, 1 H, C_qCH_{eq}HOCH, **23**), 4.49 (dd, *J* = 11.9, 4.3 Hz, 0.23 H, C*H*HOH, **23'**), 4.12 (d, *J* = 12.6 Hz, 1 H, C_qCHH_{ax}OCH, **23**), 3.96 (d, *J* = 12.3 Hz, 0.24 H, C_qC*H*HOCH, **23'**), 3.92–3.86 (m, 0.49 H, CH*H*OH + OCH, **23'**), 3.83 (s, 3 H, OCH_3, **23**), 3.81 (s, 0.67 H, OCH_3, **23'**), 3.66 (dd, *J* = 11.9, 4.4 Hz, 1 H, C*H*HOH, **23**), 3.59 (d, *J* = 12.3 Hz, 0.25 H, C_qCH*H*OCH, **23'**), 3.44–3.39 (m, 2.21 H, 2 × OCH (**23**) + OCH (**23'**)), 3.41 (dd, *J* = 11.9, 9.2 Hz, 1 H, CH*H*OH, **23**), 2.14 (dd, *J* = 9.2, 4.4 Hz, 1 H, OH, **23**), 1.96–1.87 (m, 0.51 H, CH_2, **23'**), 1.85–1.71 (m, 2.98 H, CH_2OCHC*H*H (**23**) + CH_2C*H*HCH_2 (**23**) + CH_2 (**23'**)), 1.67–1.59 (m, 2.20 H, C_qOCHC*H*H (**23**) + CH_2CH*H*CH_2 (**23**) + C*H*H (**23'**)), 1.56–1.48 (m, 1.23 H, C_qOCHCH*H* (**23**) + CH*H* (**23'**)), 1.38–1.31 (m, 1 H, CH_2OCHCH*H*, **23**); ^{13}C NMR (101 MHz, $CDCl_3$) δ 159.1 (Ar-C_qOMe, **23**), 159.0 (Ar-C_qOMe, **23'**), 132.3 (Ar-*C_q*C_q, **23'**), 131.6 (Ar-*C_q*C_q, **23**), 128.4 (2 × Ar-CH, **23**), 126.7 (2 × Ar-CH, **23'**), 114.0 (2 × Ar-CH, **23**), 113.8 (2 × Ar-CH, **23'**), 81.5 (CH_2O*C*H, **23**), 81.4 (CH_2O*C*H, **23'**), 78.3 (C_q, **23**), 77.1 (C_q, **23'**), 75.2 (C_qO*C*H, **23**), 74.5 (OCH, **23'**), 72.5 (C_q*C*H_2OCH, **23'**), 68.94 ($C_q$$CH_2$OCH, **23**), 68.87 ($CH_2$OH, **23**), 61.9 ($CH_2$OH, **23'**), 55.2 ($OCH_3$, **23** + **23'**), 24.7 (CH_2, **23'**), 24.2 (C_qOCH*C*H_2, **23**), 24.0 (CH_2, **23'**), 23.8 (CH_2OCH*C*H_2, **23**), 16.24 (CH_2*C*$H_2$$CH_2$, **23'**), 16.17 ($CH_2$*C*$H_2$$CH_2$, **23**); HRMS (ESI) *m/z* calcd for $C_{15}H_{21}O_4$ [M+H]: 265.1440, found: 265.1427.

Notes:

The doublet of doublet at 3.41 ppm (1 H) and multiplet at 3.44–3.39 ppm (2.21 H) in the ^{1}H NMR spectrum were integrated together to 3.21 H.

The assignment of $C_qCH_{eq}HOCH$ *(4.59 ppm) and* $C_qCHH_{ax}OCH$ *(4.12 ppm) in the ^{1}H NMR spectrum was performed by analogy to other examples.*

The chemical shifts of the two OCH *and* CHHOH *signals are too similar, and these appear as an indissectable multiplet at 3.44–3.39 ppm in the ^{1}H NMR spectrum. These signals could hence not be used for an unambiguous NOESY analysis for the determination of the relative stereochemistry of the major product. Nonetheless, the most relevant unambiguous NOESY signals are depicted in Fig. S16.*

***23** was further characterized by X-ray crystallography, confirming the stereochemistry (see Fig. S26). Assignment of the solved X-ray structure to the major diastereomer was performed by ^{1}H NMR (see Fig. S17).*

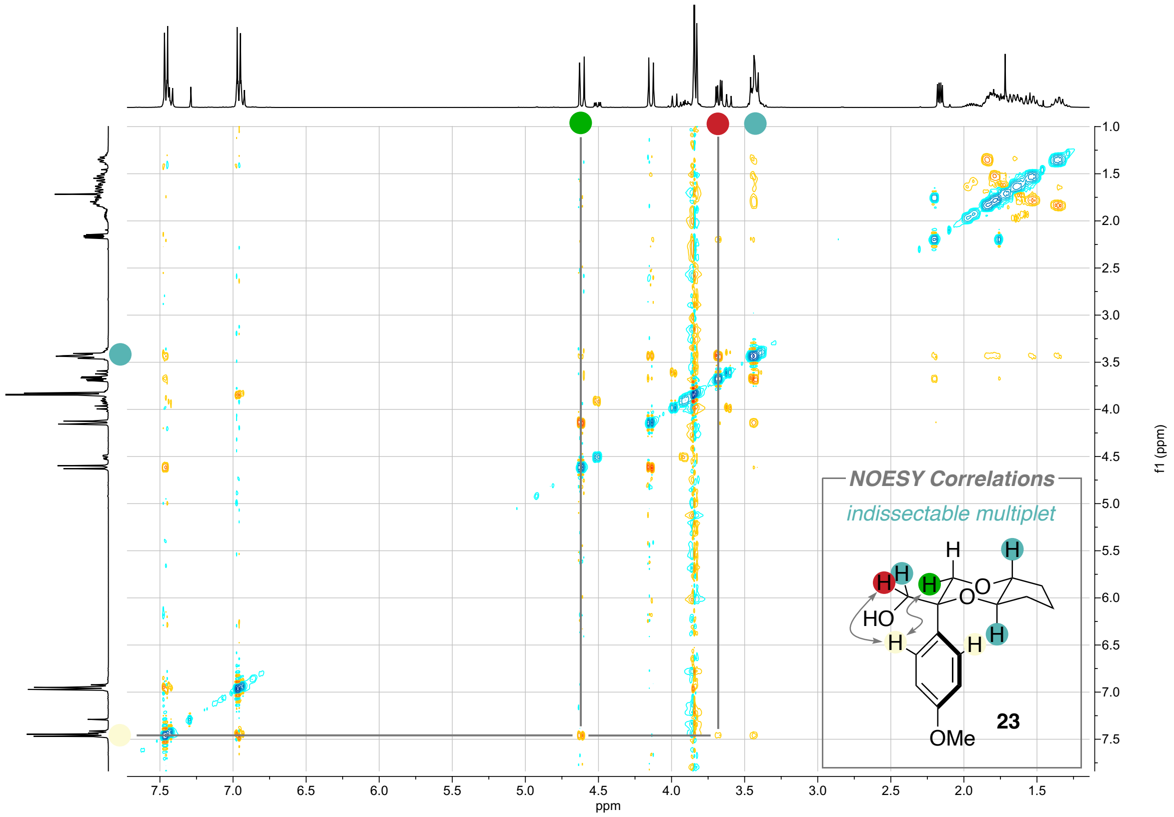

Fig. S16 NOESY spectrum ($CDCl_3$, 500 MHz) and most relevant NOESY correlations of **23** (*inlet*). Teal-colored spheres represent the protons present in the indissectable multiplet which impeded any further unambiguous analysis.

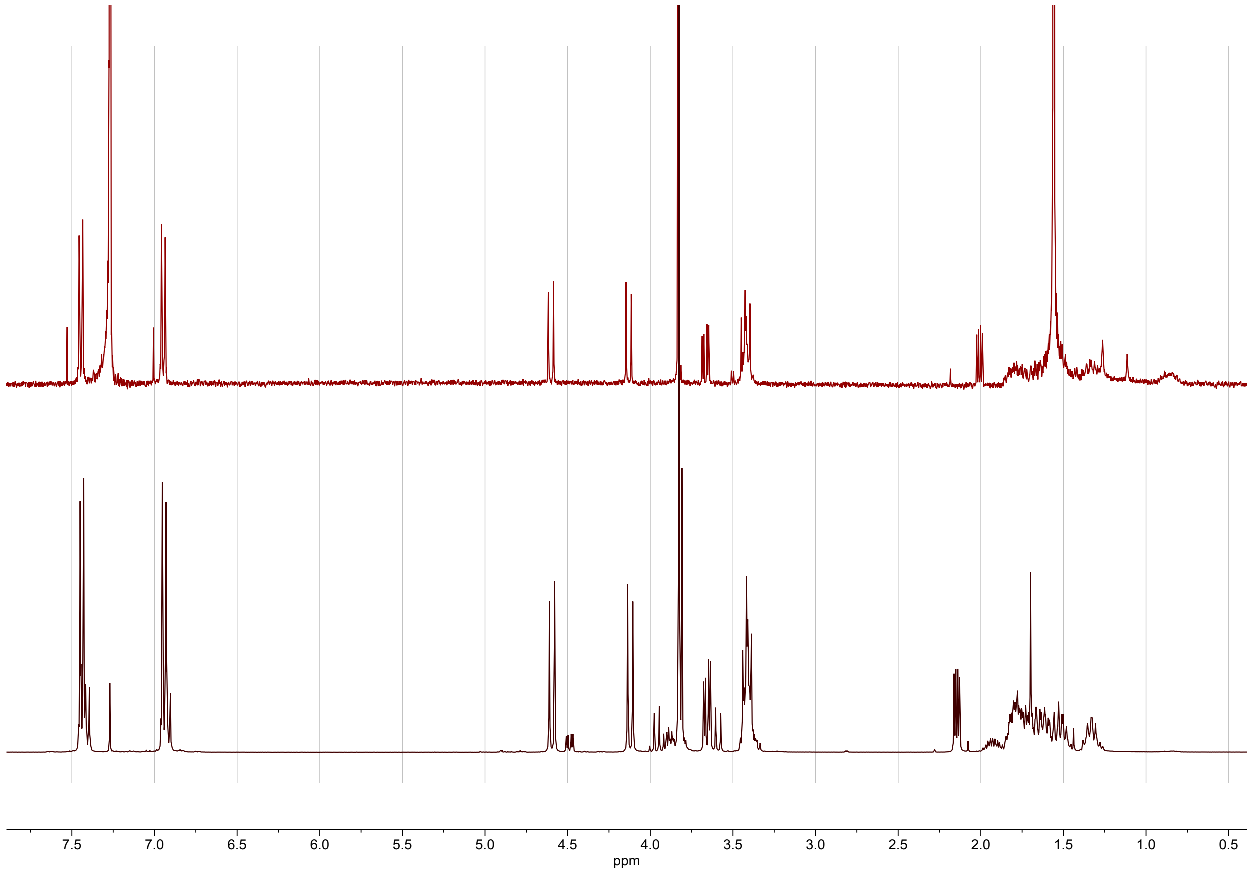

Fig. S17 ^{1}H NMR spectrum of the single crystal used for X-ray crystallography (**23**; *Top spectrum*) and of the diastereomeric mixture of **23** + **23'** (80:20; *Bottom spectrum*). This shows that the solved X-ray structure corresponds to the major diastereomer.

***all-cis*-(2-(4-Methoxyphenyl)hexahydrofuro[3,4-*b*][1,4]dioxin-2-yl)methanol (24)**

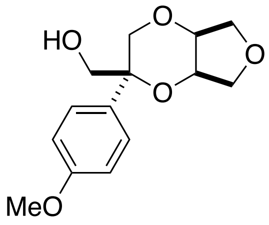

Prepared according to **General Procedure B** using 1,4-anhydroerythritol (102 μL). Purification by flash column chromatography (50–70% EtOAc/pentane) afforded dioxane **24** as a colorless gum (31.0 mg, 47%), followed by a 39:61 mixture of dioxane **24** and its minor diastereomer as a colorless gum (13.1 mg, 20%; not characterized). dr in crude reaction mixture: 79:21. R_f = 0.26 (50% EtOAc/pentane); IR (film)/cm^{-1} 3435 (br, OH), 2933, 1511, 1247, 1072; ^{1}H NMR (400 MHz, $CDCl_3$) δ 7.31–7.28 (m, 2 H, 2 × Ar-CH), 6.96–6.92 (m, 2 H, 2 × Ar-CH), 4.33 (d, *J* = 12.0 Hz, 1 H, $C_qCH_{eq}HOCH$), 4.13–4.09 (m, 1 H, CH_2OCH), 4.07–4.02 (m, 3 H, C_qOCH + CHH + CHH), 3.86–3.80 (m, 3 H, $C_qCHH_{ax}OCH$ + CHH + CHH), 3.82 (s, 3 H, OCH_3), 3.70 (d, *J* = 11.6 Hz, CHHOH), 3.52 (d, *J* = 11.6 Hz, CHHOH), 2.25 (br s, 1 H, OH); ^{13}C NMR (101 MHz, $CDCl_3$) δ 159.2 (Ar-C_qOMe), 131.2 (Ar-C_qC_q), 127.5 (2 × Ar-CH), 114.1 (2 × Ar-CH), 75.9 (C_q), 75.5 (CH_2OCH), 73.4 (CH_2), 70.3 (CH_2OH), 69.5 (CH_2), 69.0 (C_qOCH), 65.1 (C_qCH_2O), 55.3 (OCH_3); HRMS (ESI) *m/z* calcd for $C_{14}H_{18}O_5Na$ [M+Na]: 289.1052, found: 289.1065.

Notes:

The multiplet at 3.86–3.80 ppm (3 H) and singlet at 3.82 ppm (3 H) in the ^{1}H NMR spectrum were integrated together to 6 H.

*The relative stereochemistry of **24** was determined by a NOESY correlation spectrum (see Fig. S18). The key correlation between* Ar–CH *(6.31–7.28 ppm) and the multiplet between 4.13–4.09 ppm must be with* C_qOCH *since an NOE correlation with any of the other protons forming the multiplet would*

not be possible in neither diastereomer. Similarly, the correlation between CH_2OH *(3.70 and 3.52 ppm) and the multiplet between 3.86–3.80 ppm is only possible with* $CHH_{ax}OCH$.

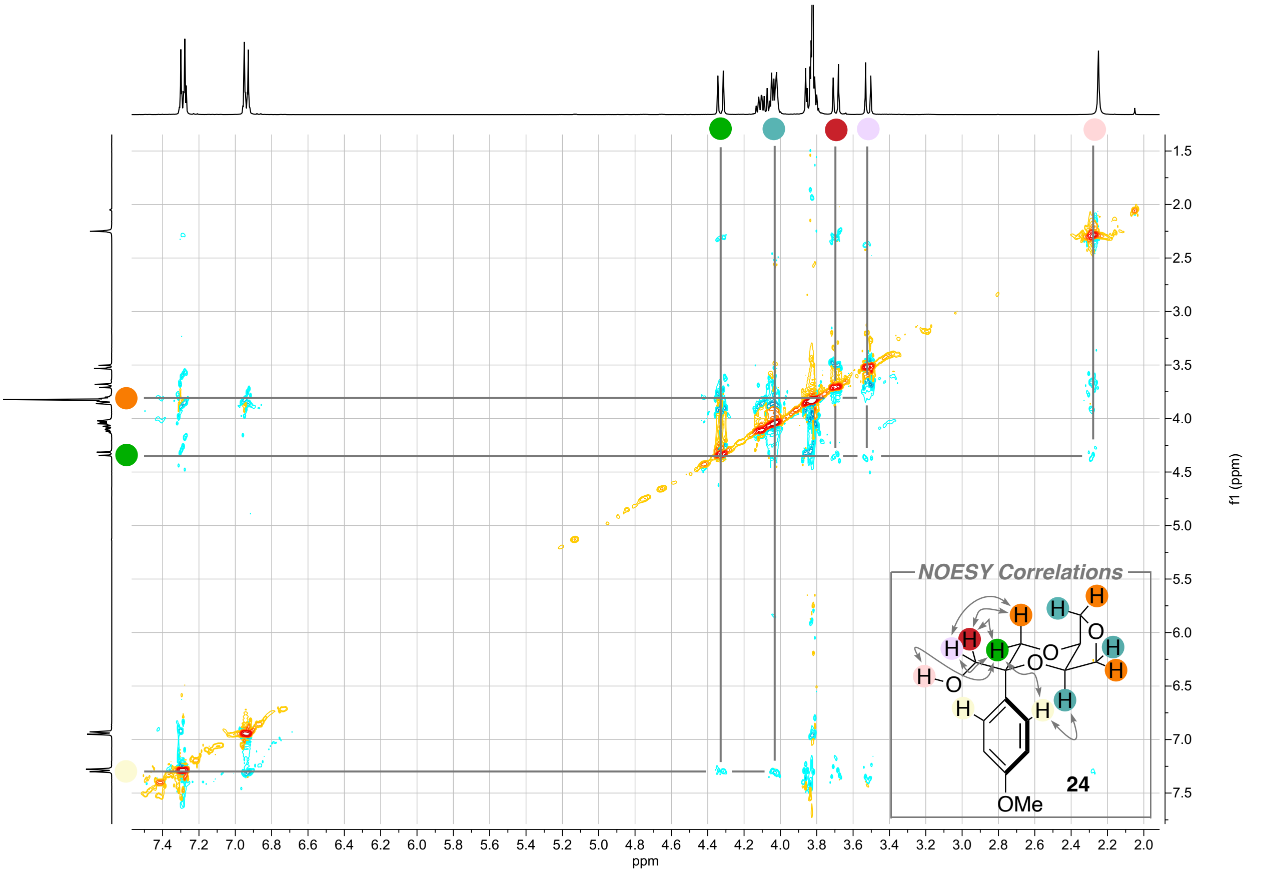

Fig. S18 NOESY spectrum ($CDCl_3$, 400 MHz) and most relevant NOESY correlations of **24** (*inlet*).

***trans*-Benzyl 3,4-dihydroxypyrrolidine-1-carboxylate (S14)**[15]

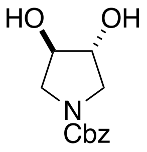

A solution of benzyl 6-oxa-3-azabicyclo[3.1.0]hexane-3-carboxylate (658 mg, 3.0 mmol, 1.0 equiv) in 2 M aq. H_2SO_4/Et_2O (1:1, 0.03 M) in a 250 mL round bottom flask was stirred at 25 °C for 20 h, then at 30 °C for 10 h and then at 40 °C for further 16 h. The reaction mixture was cooled down to 25 °C, Et_2O was added (50 mL) and the phases were separated. The aqueous layer was extracted with EtOAc (3 × 70 mL) and the organic extracts were combined, dried over Na_2SO_4, filtered and concentrated *in vacuo* using a rotatory evaporator to afford diol **S14** as a light-brown oil (521 mg, 73%). R_f = 0.18 (80% EtOAc/pentane); IR (film)/cm^{-1} 3410 (br, OH), 2923, 1675 (C=O st), 1456, 1436; ^{1}H NMR (400 MHz, $CDCl_3$) δ 7.58–7.29 (m, 5 H, 5 × Ph-CH), 5.10 (s, 2 H, CH_2Ph), 4.16–4.12 (m, 2 H, 2 × C*H*OH), 3.68 (dd, ^2J = 12.0 Hz, ^3J = 4.1 Hz, 2 H, C*H*HNC*H*H), 3.41–3.36 (br m, 4 H, 2 × OH + CH*H*NCH*H*); ^{13}C NMR (101 MHz, $CDCl_3$) δ 155.5 (C_q=O), 136.5 (Ph-C_q), 128.5 (2 × Ph-CH), 128.0 (Ph-CH), 127.8 (2 × Ph-CH), 75.3 and 74.8 (weak and broad, 2 × CHOH), 67.1 (CH_2Ph), 51.9 and 51.6 (weak and broad, CH_2NCH_2). The observed characterization data (R_f, IR, ^{1}H, ^{13}C) were consistent with that previously reported.[15]

Notes:

Hindered rotation around the C–N carbamate bond led to weak and broad signals of the pyrrolidine ring carbons in the ^{13}C NMR spectrum.

***trans-cis*-Benzyl 2-(hydroxymethyl)-2-(4-methoxyphenyl)hexahydro-6*H*-[1,4]dioxino[2,3-*c*]pyrrole-6-carboxylate (25)**

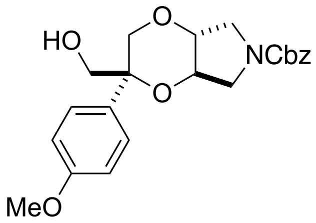

Prepared according to **General Procedure B** using pyrrolidine diol **S14** (296.6 mg). Purification by flash column chromatography (20–100% EtOAc/pentane) afforded oxetanol **1a** as a light-yellow gum (18.4 mg, 41% RSM), followed by dioxane **25** as a colorless gum (17.3 mg, 17%). dr in crude reaction mixture: >99:1. R_f = 0.11 (50% EtOAc/pentane); IR (film)/cm^{-1} 3415 (br, OH), 2947, 1700 (C=O st), 1513, 1421, 1249, 1180, 1114; ^{1}H NMR (400 MHz, $CDCl_3$) δ 7.38–7.30 (m, 5 H, 5 × Ph-CH), 7.27–7.24 (m, 2 H, 2 × Ar-CH), 6.96–6.92 (m, 2 H, 2 × Ar-CH), 5.11 and 5.10 (s, 2 H, CH_2Ph), 4.98–4.85 (2 × CH_2O), 4.12–4.10 and 4.05–4.04 (m, 1 H, CH), 3.834 and 3.825 (s, 3 H, OCH_3), 3.73–3.65 (m, 2 H, CH + C*H*HN), 3.36–3.24 (m, 2 H, CH*H*N + C*H*HN), 3.18–3.13 (CH*H*N), 2.17 (br s, 1 H, OH); ^{13}C NMR (101 MHz, $CDCl_3$) δ 159.7 (Ar-C_qOMe), 154.9 (C_q=O), 136.7 (Ph-C_q), 131.8 and 131.5 (Ar-$C_q$$C_q$), 128.5 (2 × Ph-CH), 128.0 (Ph-CH), 127.9 and 127.8 (2 × Ph-CH), 127.7 (2 × Ar-CH), 114.33 and 114.27 (2 × Ar-CH), 81.8 and 81.6 (CH_2O), 81.4 (CH_2O), 80.8 and 80.7 (C_q), 78.1 (CH), 74.5 and 73.7 (CH), 66.93 and 66.89 (CH_2Ph), 55.3 (OCH_3), 51.4 and 50.9 (CH_2N), 49.8 (CH_2N); HRMS (ESI) *m/z* calcd for $C_{22}H_{26}NO_6$ [M+H]: 400.1760, found: 400.1767.

Notes:

Only one diastereomer was observed in the ^{1}H NMR spectrum of the crude reaction mixture.

Hindered rotation around the C–N carbamate bond led to an equilibrium of rotamers which led to the splitting of some signals in the ^{1}H and ^{13}C NMR spectra.

*The relative stereochemistry of **25** could not be determined with a NOESY correlation spectrum due to the overlapping of key protons in the ^{1}H NMR spectrum. The stereochemistry was assumed to be the same as the major diastereomer in all other examples.*

*Under **General Conditions A**, **25** was isolated in 14% yield (14.2 mg). dr in crude reaction mixture: >99:1.*

Other Heterocycles (**26–29**, **S1–S10**)

(2-(4-Methoxyphenyl)-1,4-dithian-2-yl)methanol (26)

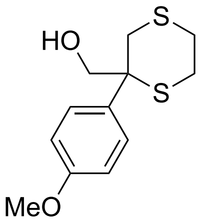

Oxetanol **1a** (45.1 mg, 0.25 mmol, 1.0 equiv) was added to an ice-cold (0 °C) solution of trifluoromethane sulfonimide (7.0 mg, 0.025 mmol, 0.1 equiv) and 1,2-ethanedithiol (0.025 mL, 0.3 mmol, 1.2 equiv) in anhydrous MeCN (0.83 mL, 0.3 M) in a flame-dried reaction tube under Ar. The reaction mixture was warmed up slowly to 30 °C, stirred at this temperature for 24 h and then quenched with sat. aq. $NaHCO_3$ (10 mL). The aqueous layer was extracted with CH_2Cl_2 (3 × 10 mL). The organic layers were combined, washed with 1 M aq. NaOH (20 mL), dried over Na_2SO_4, filtered and concentrated *in vacuo* using a rotatory evaporator. Purification by flash column chromatography (10–50% Et_2O/pentane) afforded dithiane **26** as a colorless gum (28.5 mg, 44%). R_f = 0.50 (50% Et_2O/pentane); IR (film)/cm^{-1} 3429 (br, OH), 2952, 1513, 1416, 1185, 1036; ^{1}H NMR (400 MHz, $CDCl_3$) δ 7.55–7.51 (m, 2 H, 2 × Ar-CH), 6.95–6.91 (m, 2 H, 2 × Ar-CH), 4.21 (dd, ^2J = 11.5 Hz, ^3J = 8.5 Hz, C*H*HOH), 4.05 (dd, ^2J = 11.5 Hz, ^3J = 5.1 Hz, CH*H*OH), 3.82 (s, 3 H, OCH_3), 3.48 (d, *J* = 14.3 Hz, C_qC*H*HS), 3.25 (d, *J* = 14.3 Hz, C_qCH*H*S), 3.10 (ddd, ^2J = 13.7 Hz, ^3J =7.8, 3.7 Hz, 1 H, C*H*HS), 2.87–2.84 (m, 2 H, CH_2S), 2.78 (ddd, ^2J = 13.7 Hz, ^3J =6.3, 3.2 Hz, 1 H, CH*H*S), 1.69 (dd, *J* = 8.5, 5.1 Hz, 1 H, OH); ^{13}C NMR (101 MHz, $CDCl_3$) δ 158.8 (Ar-C_qOMe), 132.8 (Ar-$C_q$$C_q$), 128.7 (2 × Ar-CH), 114.0 (2 × Ar-CH), 67.2 (CH_2OH), 55.3 (OCH_3), 48.8 (C_q), 35.7 ($C_q$$CH_2$S), 28.3 ($CH_2S$), 27.8 ($CH_2S$); HRMS (EI) *m/z* calcd for $C_{12}H_{16}O_2S_2^{\cdot+}$ [M]$^{\cdot+}$: 256.0586, found: 256.0591.

Notes:

*Running the reaction at 50 °C and using 2.0 equiv of 1,2-ethanedithiol led to isolation of side products **S1**, **S2** and **S3** (see Table S3).*

(2-(4-Methoxyphenyl)-1,4-dithian-2-yl)methanol (26), 6-(4-methoxyphenyl)-2,3-dihydro-5*H*-1,4-dithiepine (S1), **2-((6-(4-methoxyphenyl)-1,4-dithiepan-6-yl)thio)ethane-1-thiol (S2)** and **1,2-bis((6-(4-methoxyphenyl)-1,4-dithiepan-6-yl)thio)ethane (S3)**

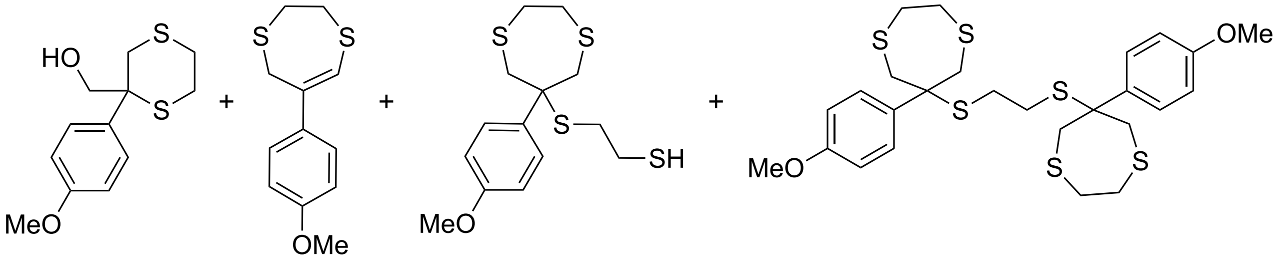

26 **S1** **S2** **S3**

Oxetanol **1a** (45.1 mg, 0.25 mmol, 1.0 equiv) was added to a solution of trifluoromethane sulfonimide (7.0 mg, 0.025 mmol, 0.1 equiv) and 1,2-ethanedithiol (0.035 mL, 0.5 mmol, 2.0 equiv) in anhydrous MeCN (0.83 mL, 0.3 M) in a flame-dried reaction tube under Ar. The reaction mixture was stirred at 50 °C for 24 h and then quenched with sat. aq. $NaHCO_3$ (10 mL). The aqueous layer was extracted with CH_2Cl_2 (3 × 10 mL). The organic layers were combined, washed with 1 M aq. NaOH (20 mL), dried over Na_2SO_4, filtered and concentrated *in vacuo* using a rotatory evaporator. Purification by flash column chromatography (10–40% Et_2O/pentane) afforded dithiepine **S1** as a white solid (5.5 mg, 9%), followed by dithiepane **S2** as a colorless gum (29.5 mg, 35%), dimer **S3** as a white solid (15.9 mg, 22%) and dithiane **26** as a colorless gum (2.5 mg, 4%).

Dithiepine **S1**: R_f = 0.59 (20% Et_2O/pentane); mp = 114 °C; IR (film)/cm^{-1} 2952, 1595, 1252, 1021, 790; ^{1}H NMR (400 MHz, $CDCl_3$) δ 7.36–7.32 (m, 2 H, 2 × Ar-CH), 6.90–6.87 (m, 2 H, 2 × Ar-CH), 6.56 (s, 1 H, C=CH), 3.95 (s, 2 H, C_qCH_2), 3.83 (s, 3 H, OCH_3), 3.22–3.20 (m, 2 H, CH_2SC*H_2*CH_2), 2.96–2.94 (m, 2 H, CH_2SCH_2C*H_2*); ^{13}C NMR (101 MHz, $CDCl_3$) δ 159.5 (Ar-C_qOMe), 152.1 (Ar-C_q*C_q*), 132.9 (Ar-*C_q*C_q), 126.9 (2 × Ar-CH), 118.9 (C=*C*H), 113.9 (2 × Ar-CH), 55.3 (OCH_3), 35.4 (CH_2SCH_2),

33.84 (CH_2S), 33.76 (CH_2S); HRMS (APCI) *m/z* calcd for $C_{12}H_{15}OS_2^+$ $[M+H]^+$: 239.0559, found: 239.0556.

Dithiepane **S2**: R_f = 0.37 (20% Et_2O/pentane); IR (film)/cm^{-1} 2907, 1506, 1219, 1192, 1029; ^{1}H NMR (400 MHz, $CDCl_3$) δ 7.38–7.34 (m, 2 H, 2 × Ar-CH), 6.90–6.86 (m, 2 H, 2 × Ar-CH), 3.82 (s, 3 H, OCH_3), 3.67 (d, *J* = 15.0 Hz, 2 H, C*H*HC_qC*H*H), 3.58 (d, *J* = 15.0 Hz, 2 H, CH*H*C_qCH*H*), 3.06–2.99 (m, 2 H, SC*H*HC*H*HS), 2.98–2.91 (m, 2 H, SCH*H*CH*H*S), 2.48–2.36 (m, 4 H, SCH_2CH_2SH), 1.52 (t, *J* = 7.8 Hz, 1 H, SH); ^{13}C NMR (101 MHz, $CDCl_3$) δ 158.5 (Ar-C_qOMe), 134.4 (Ar-C_qC_q), 128.4 (2 × Ar-CH), 113.8 (2 × Ar-CH), 58.8 (C_q), 55.2 (OCH_3), 44.0 ($CH_2C_qCH_2$), 38.2 (SCH_2CH_2S), 33.7 (CH_2CH_2SH), 24.5 (CH_2CH_2SH); HRMS (APCI) *m/z* calcd for $C_{12}H_{15}OS_2^+$ $[M–SCH_2CH_2SH]^+$: 239.0559, found: 239.0559.

Dimer **S3**: R_f = 0.15 (20% Et_2O/pentane); mp = 180 °C; IR (film)/cm^{-1} 2907, 1513, 1239, 1185, 1118, 1036; ^{1}H NMR (400 MHz, $CDCl_3$) δ 7.26–7.22 (m, 4 H, 4 × Ar-CH), 6.88–6.84 (m, 4 H, 4 × Ar-CH), 3.84 (s, 6 H, 2 × OCH_3), 3.52 (d, *J* = 15.0 Hz, 4 H, 2 × C*H*HC_qC*H*H), 3.43 (d, *J* = 15.0 Hz, 4 H, 2 × CH*H*C_qCH*H*), 3.02–2.95 (m, 4 H, 2 × SC*H*HC*H*HS), 2.94–2.87 (m, 4 H, 2 × SCH*H*CH*H*S), 2.10 (s, 4 H, 2 × CH_2); ^{13}C NMR (101 MHz, $CDCl_3$) δ 158.4 (2 × Ar-C_qOMe), 134.4 (2 × Ar-C_qC_q), 128.4 (4 × Ar-CH), 113.7 (4 × Ar-CH), 58.8 (2 × C_q), 55.3 (2 × OCH_3), 43.8 (2 × $CH_2C_qCH_2$), 38.2 (2 × SCH_2CH_2S), 29.4 (2 × CH_2); HRMS (APCI) *m/z* calcd for $C_{26}H_{34}O_2S_6{}^{35}Cl^-$ $[M+Cl]^-$: 605.0577, found: 605.0574.

(3-(4-Methoxyphenyl)-1,4-oxathian-3-yl)methanol (27) and **(2-(4-methoxyphenyl)-1,4-oxathian-2-yl)methanol (27')**

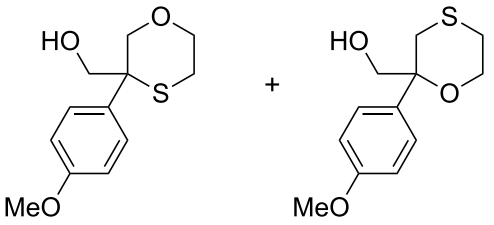

27 **27'**

Oxetanol **1a** (68.3 mg, 0.379 mmol, 1.0 equiv) was added to an ice-cold (0 °C) solution of trifluoromethane sulfonimide (10.7 mg, 0.038 mmol, 0.1 equiv) and 2-mercaptoethanol (0.020 mL, 0.285 mmol, 0.75 equiv) in anhydrous MeCN (1.26 mL, 0.3 M) in a flame-dried reaction tube under Ar. The reaction mixture was warmed up slowly to 23 °C, stirred at this temperature for 24 h and then quenched with sat. aq. $NaHCO_3$ (10 mL). The aqueous layer was extracted with CH_2Cl_2 (3 × 10 mL). The organic layers were combined, washed with 1 M aq. NaOH (20 mL), dried over Na_2SO_4, filtered and concentrated *in vacuo* using a rotatory evaporator. Purification by flash column chromatography (10–20% acetone/pentane) afforded oxathiane–O **27'** as a colorless gum (10.0 mg, 15% {yield based on 2-mercaptoethanol}), followed by a mixture of oxathiane–S **27** with oxetanol **1a** (79:21 **27**/**1a**, 46.0 mg). Repurification by flash column chromatography (30% EtOAc/pentane) afforded oxathiane–S **27** as a colorless gum (32.5 mg, 47% {yield based on 2-mercaptoethanol}).

Oxethiane–O **27'**: R_f = 0.28 (20% acetone/pentane); IR (film)/cm^{-1} 3653 (OH free), 3422 (br, OH bridged), 2982, 1513, 1252, 1081; ^{1}H NMR (400 MHz, $CDCl_3$) δ 7.34–7.30 (m, 2 H, 2 × Ar-CH), 6.98–6.94 (m, 2 H, 2 × Ar-CH), 3.96 (ddd, ^2J = 11.7 Hz, ^3J = 3.3, 3.3 Hz, 1 H, C_qOC*H*H), 3.87 (dd, ^2J = 11.7 Hz, ^3J = 2.4 Hz, 1 H, C_qOCH*H*), 3.83 (s, 3 H, OCH_3), 3.65 (dd, ^2J = 11.4 Hz, ^3J = 5.0 Hz,1 H, C*H*HOH), 3.52 (dd, ^2J = 11.4 Hz, ^3J = 8.5 Hz, 1 H, CH*H*OH), 3.38 (d, *J* = 14.3 Hz, 1 H, C_qC*H*HS), 3.02 (d, *J* = 14.3 Hz, 1 H, C_qCH*H*S), 2.90 (ddd, ^2J = 13.4 Hz, ^3J = 11.1, 3.3 Hz, 1 H, CH_2C*H*HS), 2.26 (ddd, ^2J = 13.4 Hz, ^3J = 2.5, 2.4 Hz, 1 H, CH_2CH*H*S), 1.93 (dd, *J* = 8.5, 5.0 Hz,1 H, OH); ^{13}C NMR (101 MHz, $CDCl_3$) δ 159.0 (Ar-C_qOMe), 130.9 (Ar-C_qC_q), 128.6 (2 × Ar-CH), 114.1 (2 × Ar-CH), 77.2 (C_q), 71.6 (CH_2OH), 62.5 (C_qOCH_2), 55.2 (OCH_3), 29.5 (C_qCH_2S), 26.9 (SCH_2CH_2); HRMS (EI) *m/z* calcd for $C_{11}H_{13}O_2S^+$ $[M–CH_2OH]^+$: 209.0631, found: 209.0629.

Oxethiane–S **27**: R_f = 0.24 (20% acetone/pentane); IR (film)/cm^{-1} 3429 (br, OH), 2937, 1513, 1252, 1185; ^{1}H NMR (400 MHz, $CDCl_3$) δ 7.52–7.48 (m, 2 H, 2 × Ar-CH), 6.93–6.89 (m, 2 H, 2 × Ar-CH), 4.19 (12.2 Hz, 1 H, C_qCHHOCH$_2$), 4.16 (12.2 Hz, 1 H, C_qCH*H*OCH$_2$), 4.14–4.08 (m, 1 H, CH$_2$C*H*HO), 4.07–3.99 (m, 2 H, CH$_2$OH), 3.84 (dd, *J* = 9.3, 2.6 Hz, 1 H, CH$_2$CH*H*O), 3.81 (s, 3 H, OCH_3), 2.95 (ddd, ^2J = 13.8 Hz, ^3J = 9.3, 3.2 Hz, 1 H, SC*H*H), 2.46 (ddd, ^2J = 13.8 Hz, ^3J = 4.7, 2.7 Hz, 1 H, SCH*H*), 1.88 (dd, *J* = 8.2, 5.2 Hz,1 H, OH); ^{13}C NMR (101 MHz, $CDCl_3$) δ 158.8 (Ar-C_qOMe), 131.4 (Ar-$C_q$$C_q$), 128.5 (2 × Ar-CH), 114.0 (2 × Ar-CH), 73.9 (C_qCH$_2$OCH$_2$), 68.3 (CH$_2$CH$_2$O), 65.1 (CH$_2$OH), 55.2 (OCH_3), 49.1 (C_q), 25.2 (SCH_2); HRMS (EI) *m/z* calcd for $C_{12}H_{16}O_3S^{\cdot+}$ $[M]^{\cdot+}$: 240.0815, found: 240.0808.

(2-(4-Methoxyphenyl)-2,3-dihydrobenzo[b][1,4]dioxin-2-yl)methanol (28)

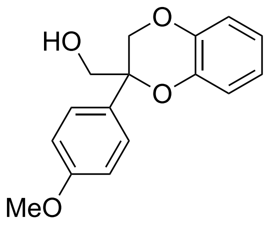

Oxetanol **1a** (81.8 mg, 0.45 mmol, 1.0 equiv) was added to a solution of trifluoromethane sulfonic acid (3.4 mg, 0.0226 mmol, 0.05 equiv) and catechol (248 mg, 2.25 mmol, 5.0 equiv) in anhydrous chloroform (0.91 mL, 0.5 M) in a flame-dried reaction tube under Ar. The reaction mixture was stirred at 25 °C for 24 h and then quenched with sat. aq. $NaHCO_3$ (10 mL). The aqueous layer was extracted with CH_2Cl_2 (3 × 10 mL). The organic layers were combined, dried over Na_2SO_4, filtered and concentrated *in vacuo* using a rotatory evaporator. Purification by flash column chromatography (3–20% Et_2O/pentane) afforded benzodioxane **28** as a white solid (28.1 mg, 23%). R_f = 0.60 (10% Et_2O/pentane); mp = 128–131 °C; IR (film)/cm^{-1} 3390 (br, OH), 2952, 1494, 1263; ^{1}H NMR (400 MHz, $CDCl_3$) δ 7.42–7.38 (m, 2 H, 2 × Ar-CH), 7.05–7.03 (m, 1 H, Ar-CH), 6.92–6.87 (m, 3 H, 3 × Ar-CH), 6.84–6.81 (m, 2 H, 2 × Ar-CH), 4.52 (d, *J* = 11.8 Hz, 1 H, C*H*HOAr-C_q), 4.42 (d, *J* = 11.8 Hz, 1 H, CH*H*OAr-C_q), 3.94 (dd, ^2J = 12.2 Hz, ^3J = 5.4 Hz, 1 H, C*H*HOH), 3.83 (dd, ^2J = 12.2 Hz, ^3J = 8.2 Hz, 1 H, CH*H*OH), 3.79 (s, 3 H, OCH_3), 1.95 (dd, *J* = 8.2, 5.4 Hz, 1 H, OH); ^{13}C NMR (101 MHz, $CDCl_3$) δ 159.4 (Ar-C_qOMe), 142.8 (Ar-C_qOCH$_2$), 142.4 (Ar-C_qOC_q), 129.7 (Ar-$C_q$$C_q$), 127.3 (2 × Ar-CH), 121.8 (Ar-CH), 121.5 (Ar-CH), 117.6 (Ar-CH), 117.2 (Ar-CH), 114.1 (2 × Ar-CH), 78.6 (C_q), 67.5 (CH$_2$OH), 66.6 (*C*H$_2$OAr-C_q), 55.2 (OCH_3); HRMS (ESI) *m/z* calcd for $C_{16}H_{15}O_4$ [M–H]: 271.0970, found: 271.0964.

5-(Hydroxymethyl)-5-(4-methoxyphenyl)-1,4-dioxan-2-one (29)

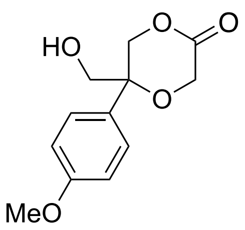

Oxetanol **1a** (45.1 mg, 0.25 mmol, 1.0 equiv) was added to a solution of trifluoromethane sulfonimide (7.0 mg, 0.025 mmol, 0.1 equiv) and glycolic acid (95.1 mg, 1.25 mmol, 5.0 equiv) in anhydrous MeCN (0.83 mL, 0.3 M) in a flame-dried reaction tube under Ar. The reaction mixture was stirred at 50 °C for 24 h and then quenched with distilled water (10 mL). The aqueous layer was extracted with CH_2Cl_2 (3 × 10 mL). The organic layers were combined, dried over Na_2SO_4, filtered and concentrated *in vacuo* using a rotatory evaporator. Purification by flash column chromatography (40–50% EtOAc/pentane) afforded dioxanone **29** as a colorless gum (41.0 mg, 69%). R_f = 0.27 (50% EtOAc/pentane); IR (film)/cm^{-1} 3474 (br, OH), 2937, 1744 (C=O st), 1610, 1513, 1252; ^{1}H NMR (400 MHz, $CDCl_3$) δ 7.39–7.35 (m, 2 H, 2 × Ar-CH), 6.98–6.94 (m, 2 H, 2 × Ar-CH), 4.95 (d, *J* = 12.2 Hz, 1 H, C*H*HOC=O), 4.91 (d, *J* = 12.2 Hz, 1 H, CH*H*OC=O), 4.42 (d, *J* = 17.8 Hz, 1 H, OC*H*HC=O), 4.16 (d, *J* = 17.8 Hz, 1 H, OCH*H*C=O), 3.82 (s, 3 H, OCH_3), 3.80 (dd, ^2J = 12.1 Hz, ^3J = 4.5 Hz, 1 H, C*H*HOH), 3.59 (dd, ^2J = 12.1 Hz, ^3J = 8.3 Hz, 1 H, CH*H*OH), 2.12 (dd, *J* = 8.3, 4.5 Hz, 1 H, OH); ^{13}C NMR (101 MHz, $CDCl_3$) δ 166.8 (C_q=O), 160.0 (Ar-C_qOMe), 128.1 (2 × Ar-CH), 126.9 (Ar-$C_q$$C_q$), 114.6 (2 × Ar-CH), 76.1 (C_q), 69.9 (*C*H$_2$OC=O), 67.8 (CH$_2$OH), 61.9 (O*C*H$_2$C=O), 55.3 (OCH_3); HRMS (APCI) *m/z* calcd for $C_{12}H_{15}O_6^-$ $[M+OH]^-$: 255.0874, found: 255.0867.

Notes:

A basic environment, in particular during workup, was found to ring-open the lactone, which reformed under acidic conditions.

*The regiochemistry of **29** was determined with an HMBC correlation spectrum (see Fig. S19).*

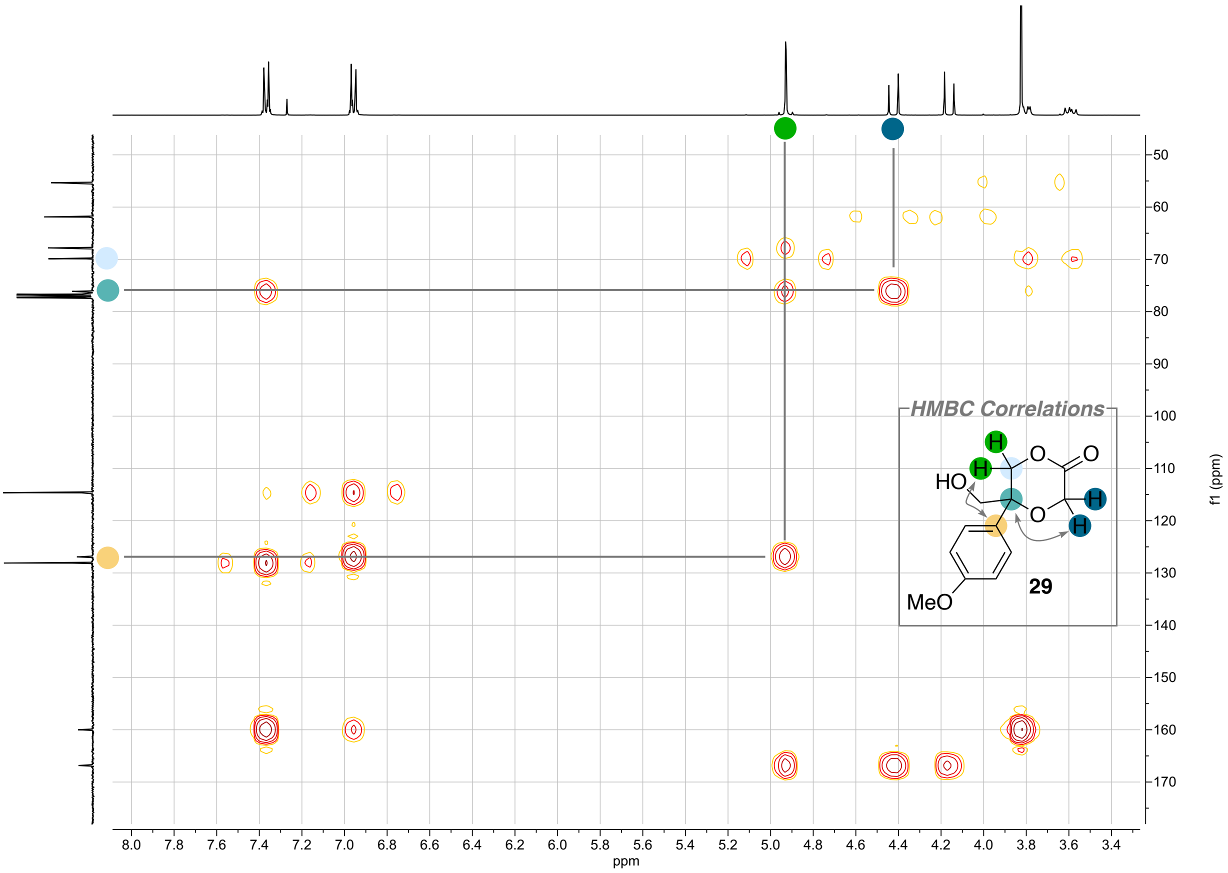

Fig. S19 HMBC spectrum (^{1}H: $CDCl_3$, 400 MHz; ^{13}C: $CDCl_3$, 101 MHz) and most relevant HMBC correlations of **29** (*inlet*).

(3-(4-Methoxyphenyl)-2,3-dihydrobenzo[b][1,4]oxathiin-3-yl)methanol (S9) and (2-(4-methoxyphenyl)-2,3-dihydrobenzo[b][1,4]oxathiin-2-yl)methanol (S10)

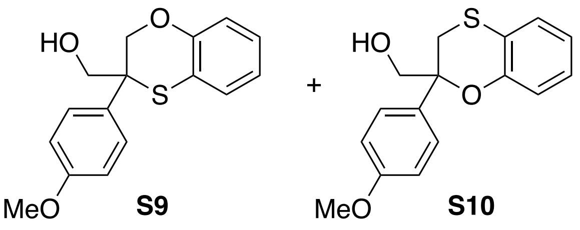

Oxetanol **1a** (84.0 mg, 0.47 mmol, 1.0 equiv) was added to a solution of trifluoromethane sulfonic acid (3.5 mg, 0.023 mmol, 0.1 equiv) and 2-mercaptophenol (0.09 mL, 0.93 mmol, 2.0 equiv) in toluene (0.63 mL, 0.75 M) in a flame-dried reaction tube under Ar. The reaction mixture was stirred at 40 °C for 24 h and then quenched with sat. aq. $NaHCO_3$ (10 mL). The aqueous layer was extracted with CH_2Cl_2 (3 × 10 mL). The organic layers were combined, dried over Na_2SO_4, filtered and concentrated *in vacuo* using a rotatory evaporator. Purification by flash column chromatography (2.5% Et_2O/CH_2Cl_2) afforded a 76:24 mixture of benzoxathianes **S9** and **S10** as a colorless oil (47.4 mg, 35%). R_f = 0.21 (100% CH_2Cl_2); IR (film)/cm^{-1} 3418 (br, OH), 2933, 1512, 1474, 1250, 1182, 1125, 1028, 750; ^{1}H NMR (400 MHz, $CDCl_3$) δ 7.53–7.49 (m, 2 H, 2 × Ar-CH, **S9**), 7.33–7.30 (m, 0.73 H, 2 × Ar-CH, **S10**), 7.11–7.08 (m, 1 H, Ar-CH, **S9**), 7.07–7.02 (m, 1.68 H, Ar-CH (**S9**) + 2 × Ar-CH (**S10**)), 6.99–6.97 (m, 0.37 H, Ar-CH, **S10**), 6.96–6.92 (m, 3.71 H, 3 × Ar-CH (**S9**) + 2 × Ar-CH (**S10**)), 6.90–6.85 (m, 1 H, Ar-CH, **S9**), 6.84–6.82 (m, 0.31 H, Ar-CH, **S10**), 4.68 (d, *J* = 11.6 Hz, 1 H, C*H*HOAr-C_q, **S9**), 4.39 (d, *J* = 11.6 Hz, 1 H, CH*H*OAr-C_q, **S9**), 4.11 (dd, ^2J = 11.9 Hz, ^3J = 7.4 Hz, 1 H, C*H*HOH, **S9**), 4.05 (dd, ^2J = 11.9 Hz, ^3J = 5.1 Hz, 1 H, CH*H*OH, **S9**), 3.94 (dd, ^2J = 11.8 Hz, ^3J = 4.7 Hz, 1 H, C*H*HOH, **S10**), 3.82 (s, 3 H, OCH_3, **S9**), 3.80–3.79 (m, 0.33 H, CH*H*OH, **S10**), 3.78 (s, 0.99 H, OCH_3, **S10**), 3.56 (d, *J* = 13.7 Hz, 0.30 H, C*H*HOAr-C_q, **S10**), 3.28 (d, *J* = 13.7 Hz, 0.28 H, CH*H*OAr-C_q, **S10**), 2.23–2.20 (m, 1.29 H, OH (**S9**) + OH (**S10**)); ^{13}C NMR (101 MHz, $CDCl_3$) δ 159.1 (Ar-C_qOMe, **S9**), 159.0 (Ar-C_qOMe, **S10**), 150.4 (Ar-C_qOCH$_2$, **S9**), 131.7 (Ar-C_qC$_q$, **S10**), 130.1 (Ar-C_qC$_q$, **S9**), 128.4 (2 × Ar-CH, **S9**), 127.4 (Ar-CH, **S10**), 127.1 (Ar-CH, **S9**), 127.0 (2 × Ar-CH, **S10**), 125.8 (Ar-CH, **S10**), 125.6 (Ar-CH, **S9**), 121.9 (Ar-CH, **S9**), 121.4 (Ar-CH, **S10**), 118.8 (Ar-C_qS, **S9**), 118.2 (Ar-CH, **S9**), 117.8 (Ar-CH, **S10**), 117.0 (Ar-

C_qS, **S10**), 114.2 (2 × Ar-CH, **S9**), 113.9 (2 × Ar-CH, **S10**), 78.7 (C_q, **S10**), 70.2 (CH_2OH, **S10**), 69.6 (CH_2O, **S9**), 65.5 (CH_2O, **S9**), 55.2 (OCH_3, **S9**), 55.1 (OCH_3, **S10**), 50.9 (C_q, **S9**), 29.5 (CH_2S, **S10**); HRMS (APCI) *m/z* calcd for $C_{16}H_{17}O_3S^+$ $[M+H]^+$: 289.0893, found: 289.0895.

Notes:

The Ar-$C_q$$OCH_2$ *signal from **S10** was not observed in the* ^{13}*C NMR spectrum. Presumably, it lies under the* Ar-$C_q$$OCH_2$ *signal from **S9** (150.4 ppm).*

2-((3-(4-Methoxyphenyl)oxetan-3-yl)thio)phenol (S8)

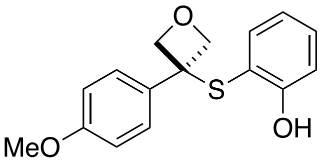

Lithium bis(trifluoromethanesulfonimide) (15.8 mg, 0.055 mmol, 0.11 equiv) and tetrabutylammonium hexafluorophosphate (10.7 mg, 0.0275 mmol, 0.055 equiv) were added sequentially to a solution of oxetanol **1a** (90.0 mg, 0.5 mmol, 1.0 equiv) and 4-mercaptophenol (0.10 mL, 1 mmol, 2.0 equiv) in chloroform (1.0 mL, 0.5 M). The reaction mixture was stirred at 40 °C for 2 h, then quenched with sat. aq. $NaHCO_3$ (10 mL). The aqueous layer was extracted with CH_2Cl_2 (3 × 10 mL). The organic layers were combined, dried over Na_2SO_4, filtered and concentrated *in vacuo* using a rotatory evaporator. Purification by flash chromatography (2.5% Et_2O/CH_2Cl_2) afforded thioether **S8** as a pale-yellow oil which crystallized in the freezer to a white solid (46.0 mg, 32%). R_f = 0.38 (2.5% Et_2O/CH_2Cl_2); mp = 83–86 °C; IR (film)/cm^{-1} 3398 (br, OH), 2955, 1513, 1468, 1254, 1179, 1027; ^{1}H NMR (400 MHz, $CDCl_3$) δ 7.33–7.32 (m, 1 H, Ar-CH), 7.24–7.22 (m, 1 H, Ar-CH), 6.95–6.93 (m, 1 H, Ar-CH), 6.86–6.85 (m, 5 H, 4 × $Ar_{(PMP)}$-CH + Ar-CH), 6.50 (s, 1 H, OH), 5.13 (d, *J* = 6.6 Hz, 2 H, C*H*HOC*H*H), 4.97 (d, *J* = 6.6 Hz, 2 H, CH*H*OCH*H*), 3.83 (s, 3 H, OCH_3); ^{13}C NMR (101 MHz, $CDCl_3$) δ 159.0 (Ar-C_qOMe), 158.1 (Ar-C_qOH), 137.4 (Ar-$C_q$$C_q$), 134.3 (Ar-CH), 132.4 (Ar-CH), 127.2 (2 × Ar-CH), 120.6 (Ar-CH), 115.9 (Ar-C_qS), 115.1 (Ar-CH), 114.0 (2 × Ar-CH), 82.1 (CH_2OCH_2), 56.1 (C_q), 55.3 (OCH_3); HRMS (APCI) *m/z* calcd for $C_{16}H_{15}O_3S$ [M–H]: 287.0736, found: 287.0739.

Notes:
***S8** was further characterized by X-ray crystallography (see Fig. S2 and Fig. S28).*

Derivatization of 1,4-Dioxane products (**30**–**33**)

2-(4-Methoxyphenyl)-1,4-dioxane-2-carboxylic acid (30)[16]

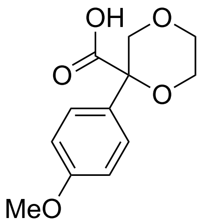

An aqueous solution of $KMnO_4$ (1.11 M, 0.48 mL, 0.532 mmol, 2.66 equiv) was added dropwise over 5 min to a solution of 1,4-dioxane **2** (44.9 mg, 0.2 mmol, 1.0 equiv) in 1 M aq. NaOH (0.27 mL, 0.74 M) at 0 °C in a reaction tube. The reaction mixture was warmed to 25 °C and stirred at this temperature for 91 h (~50% conversion by ^{1}H NMR). Further $KMnO_4$ was added (aq. solution, 1.11 M, 0.48 mL, 0.532 mmol, 2.66 equiv) and after stirring for 5 h, the reaction mixture was acidified to pH ~ 1 with 1 M aq. H_2SO_4. The aqueous mixture was extracted with EtOAc (4 × 10 mL), the combined organic layers filtered through celite, dried over Na_2SO_4, filtered and concentrated *in vacuo* using a rotatory evaporator to afford carboxylic acid **30** as a colorless gum which solidified to a light-yellow solid (46.2 mg, 97%). R_f = 0.14 (50% EtOAc/pentane); mp = 80 °C; IR (film)/cm^{-1} 3534 (br, COOH free), 2997 (br, COOH bridged), 2922, 1715 (C=O st), 1513, 1245, 1088; ^{1}H NMR (400 MHz, $CDCl_3$) δ 7.65 (br s, 1 H, COOH), 7.46 (d, *J* = 8.6 Hz, 2 H, 2 × Ar-CH), 6.91 (d, *J* = 8.6 Hz, 2 H, 2 × Ar-CH), 4.55 (d, *J* = 11.8 Hz, 1 H, C_q*H*HO), 4.06 (ddd, ^2J = 12.6 Hz, ^3J = 9.7, 3.1 Hz, 1 H, C*H*HO), 3.90 (ddd, ^2J = 13.1 Hz, ^3J = 3.3, 3.3 Hz, 1 H, C*H*HO), 3.81 (s, 3 H, OCH_3), 3.83–3.81 (m, 1 H, CH*H*O), 3.71 (d, *J* = 11.8 Hz,1 H, C_qH*H*O), 3.73–3.67 (m, 1 H, CH*H*O); ^{13}C NMR (101 MHz, $CDCl_3$) δ 176.0 (C_q=O), 159.8 (Ar-C_qOMe), 128.4 (Ar-*C*$_q$C$_q$), 126.6 (2 × Ar-CH), 114.0 (2 × Ar-CH), 78.5 (C_q), 71.6 (CH_2O), 66.3 (CH_2O), 63.5 (CH_2O), 55.3 (OCH_3); HRMS (APCI) *m/z* calcd for $C_{12}H_{13}O_5^-$ [M–H]$^-$: 237.0768, found: 237.0765.

Notes:

It is not recommended to follow the reaction by TLC since there are Mn-byproducts that overlap with carboxylic acid ***30*** *and give the false impression of high conversion. Following the reaction by ^{1}H NMR was found to be reliable.*

The broad singlet at 7.65 ppm (1 H) and doublet at 7.46 ppm (2 H) in the ^{1}H NMR spectrum were integrated together to 3 H.

The singlet at 3.81 ppm (3 H) and multiplet at 3.83–3.81 ppm (1 H) in the ^{1}H NMR spectrum were integrated together to 4 H.

The doublet at 3.71 ppm (1 H) and multiplet at 3.73–3.67 ppm (1 H) in the ^{1}H NMR spectrum were integrated together to 2 H.

2-(4-Methoxyphenyl)-2-((prop-2-yn-1-yloxy)methyl)-1,4-dioxane (31)

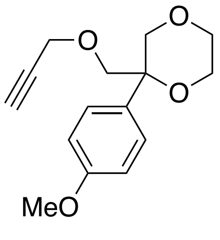

NaH (60% in mineral oil, 40.0 mg, 1.0 mmol, 5.0 equiv) was added portionwise to a solution of 1,4-dioxane **2** (44.9 mg, 0.2 mmol, 1.0 equiv) in anhydrous DMF (0.61 mL, 0.33 M) at 0 °C in a reaction tube. After stirring for 30 min at 0 °C, 3-bromopropyne (80% in toluene, 44.6 μL, 0.4 mmol, 2.0 equiv) was added, the reaction mixture warmed to 25 °C and stirred at this temperature for 19 h. The reaction was quenched with ice-cold (0 °C) distilled water (10 mL) and the aqueous mixture was extracted with EtOAc (4 × 10 mL). The organic layers were combined, dried over Na_2SO_4, filtered and concentrated *in vacuo* using a rotatory evaporator. Purification by flash chromatography (20% Et_2O/pentane) afforded alkyne **31** as a light-yellow gum (34.5 mg, 66%). R_f = 0.11 (20% Et_2O/pentane); IR (film)/cm^{-1} 3280 (C≡C–H st), 2857, 1512, 1248, 1110, 1095; ^{1}H NMR (400 MHz, $CDCl_3$) δ 7.45–7.41 (m, 2 H, 2 × Ar-CH), 6.96–6.92 (m, 2 H, 2 × Ar-CH), 4.27 (d, *J* = 12.2 Hz, 1 H, C_qC*H*HO), 4.16 (dd, ^2J = 16.0 Hz, ^4J = 2.4 Hz, 1 H, C*H*HC≡CH), 4.10 (dd, ^2J = 16.0 Hz, ^4J = 2.4 Hz, 1 H, CH*H*C≡CH), 3.99 (d, *J* = 12.2 Hz, 1 H, C_qCH*H*O), 3.82 (s, 3 H, OCH_3), 3.80–3.71 (m, 2 H, C*H*HC*H*HO), 3.68 (d, *J* = 10.3 Hz, 1 H, C_qC*H*HO), 3.61 (d, *J* = 10.3 Hz, 1 H, C_qCH*H*O), 3.69–3.60 (m, 2 H, CH*H*CH*H*O), 2.41 (dd, *J* = 2.4, 2.4 Hz, 1 H, C≡CH); ^{13}C NMR (101 MHz, $CDCl_3$) δ 159.0 (Ar-C_qOMe), 131.4 (Ar-*C*$_q$C$_q$), 128.2 (2 × Ar-CH), 113.8 (2 × Ar-CH), 79.4

(*C*≡CH), 76.1 (C_q), 74.7 (C≡*C*H), 74.2 (C_q*C*H_2O), 69.6 (C_q*C*H_2O), 66.8 (*C*H_2CH_2O), 61.3 (CH_2*C*H_2O), 58.8 (*C*H_2C≡CH), 55.2 (OCH_3); molecular ion not found by APCI in negative or positive mode.

Notes:

The doublet at 3.68 ppm (1 H), doublet at 3.61 ppm (1 H) and multiplet at 3.69–3.60 ppm (2 H) in the 1H NMR spectrum were integrated together to 4 H.

Presumably strong fragmentation, known for aliphatic alkynes,[17] *led to intractable ion fragments in the mass spectrum.*

2-((2-(4-Methoxyphenyl)-1,4-dioxan-2-yl)methoxy)pyridine (32)[18]

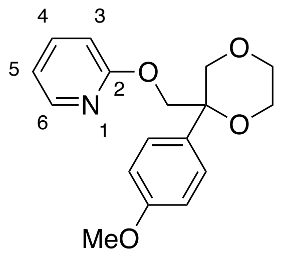

NaH (60% in mineral oil, 9.6 mg, 0.24 mmol, 1.2 equiv) was added portionwise to a solution of 1,4-dioxane **2** (44.9 mg, 0.2 mmol, 1.0 equiv) in anhydrous DMF (1.1 mL, 0.18 M) at 0 °C in a reaction tube. After stirring for 30 min at 0 °C, a solution of 2-fluoropyridine in DMF (2 M, 133 μL, 0.266 mmol, 1.3 equiv) was added, the reaction mixture warmed to 90 °C and stirred at this temperature for 24 h. The reaction was cooled to 25 °C, sat. aq. NH_4Cl (10 mL) was added and the aqueous mixture was extracted with EtOAc (3 × 10 mL). The organic layers were combined, dried over Na_2SO_4, filtered and concentrated *in vacuo* using a rotatory evaporator. Purification by flash chromatography (20–40% Et_2O/pentane) afforded pyridine **32** as a colorless gum (27.3 mg, 45%). R_f = 0.31 (40% Et_2O/pentane); IR (film)/cm^{-1} 2954, 1599, 1431, 1249, 1113; ^{1}H NMR (400 MHz, $CDCl_3$) δ 8.09 (dd, ^3J = 5.1 Hz, ^4J = 2.1 Hz, 1 H, H6), 7.54 (ddd, ^3J = 8.4, 7.2 Hz, ^4J = 2.1 Hz, 1 H, H4), 7.51–7.47 (m, 2 H, 2 × Ar-CH), 6.96–6.92 (m, 2 H, 2 × Ar-CH), 6.84 (dd, *J* = 7.2, 5.1 Hz, 1 H, H5), 6.76 (d, *J* = 8.4 Hz, 1 H, H3), 4.49 (d, *J* = 11.4 Hz, 1 H, C_qC*H*HO), 4.43 (d, *J* = 11.4 Hz, 1 H, C_qCH*H*O), 4.33 (d, *J* = 12.2 Hz, 1 H, C_qC*H*HO), 4.06 (d, *J* = 12.2 Hz, 1 H, C_qCH*H*O), 3.82 (s, 3 H, OCH_3), 3.79–3.75 (m, 3 H, C*H*HC*H₂*O), 3.71–3.64 (m, 1 H, CH*H*CH_2O); ^{13}C NMR (101 MHz, $CDCl_3$) δ 163.4 (C2), 159.0 (Ar-C_qOMe), 146.6 (C6), 138.5 (C4), 131.3 (Ar-*C*$_q$C$_q$), 128.3 (2 × Ar-CH), 116.9 (C5), 113.8 (2 × Ar-CH), 111.3 (C3), 75.8 (C_q), 69.7 (C_q*C*H_2O), 69.2 (C_q*C*H_2O), 66.9 (*C*H_2CH_2O), 61.4 (CH_2*C*H_2O), 55.2 (OCH_3); HRMS (ESI) *m/z* calcd for $C_{17}H_{20}NO_4$ [M+H]: 302.1392, found: 302.1387.

4-(2-(Hydroxymethyl)-1,4-dioxan-2-yl)phenyl trifluoromethanesulfonate (33')

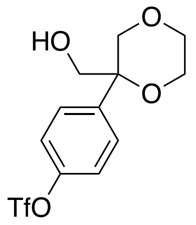

Triethylamine (42 μL, 0.30 mmol, 3.0 equiv), 4-dimethylaminopyridine (DMAP, 1.2 mg, 0.01 mmol, 0.1 equiv) and *N*,*N*-bis(Trifluoromethylsulfonyl)aniline (NTf_2Ph, 53.6 mg, 0.15 mmol, 1.5 equiv) were added sequentially to a solution of phenol dioxane **8** (21.0 mg, 0.10 mmol, 1.0 equiv) in anhydrous CH_2Cl_2 (0.5 mL, 0.2 M) at 0 °C. After stirring for 5 min at 0 °C, the ice bath was removed and the reaction mixture was stirred for further 4 h at 25 °C. Then, the reaction mixture was concentrated *in vacuo* using a rotatory evaporator. Purification by flash column chromatography (5–20% Et_2O/CH_2Cl_2) afforded triflate **33'** as a colorless gum (21.4 mg, 63%). R_f = 0.27 (20% Et_2O/CH_2Cl_2); IR (film)/cm^{-1} 3467 (br, OH), 2963, 1423, 1211, 1140, 889; ^{1}H NMR (400 MHz, $CDCl_3$) δ 7.60–7.56 (m, 2 H, 2 × Ar-CH), 7.34–7.30 (m, 2 H, 2 × Ar-CH), 4.25 (d, *J* = 12.5 Hz, 1 H, C_qC*H*HOCH_2), 4.03 (d, *J* = 12.5 Hz, 1 H, C_qCH*H*OCH_2), 3.80–3.63 (m, 6 H, 3 × OCH_2), 1.96 (br t, *J* = 6.5 Hz, 1 H, OH); ^{13}C NMR (101 MHz, $CDCl_3$) δ 149.0 (Ar-C_qOTf), 140.1 (Ar-*C*$_q$C$_q$), 129.0 (2 × Ar-CH), 121.5 (2 × Ar-CH), 118.7 (q, J_{CF} = 320.6 Hz, CF_3), 76.7 (C_q), 69.1 (C_q*C*H_2OCH_2), 67.2 (OCH_2), 66.7 (OCH_2), 61.5 (C_qO*C*H_2); ^{19}F NMR (377 MHz, CDCl3) δ –72.9; HRMS (EI) *m/z* calcd for $C_{11}H_{10}F_3O_5S^+$ [M–CH_2OH]$^+$: 311.0196, found: 311.0201.

Notes:

Only the two central peaks of the CF_3 signal (quartet) were observed in the ^{13}C NMR spectrum.

(2-(4-(2-(Trifluoromethyl)pyrimidin-5-yl)phenyl)-1,4-dioxan-2-yl)methanol (33)

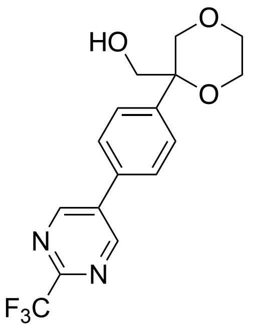

A reaction tube was charged sequentially with triflate **33'** (19.0 mg, 0.0555 mmol, 1.0 equiv), (2-(trifluoromethyl)pyrimidin-5-yl)boronic acid pinacol ester (22.8 mg, 0.0833 mmol, 1.5 equiv), $Pd(OAc)_2$ (0.6 mg, 0.0028 mmol, 5 mol%), SPhos (2.3 mg, 0.00555 mmol, 0.1 equiv) and K_3PO_4 (23.6 mg, 0.111 mmol, 2.0 equiv). The reaction vessel was sealed and evacuated and then refilled with argon (× 3). In a separate flask, anhydrous dioxane was sparged with argon for 15 min. The same procedure was performed with distilled water. Sparged dioxane (0.44 mL, dioxane:H_2O = 4:1, 0.1 M) and sparged water (0.11 mL) were to the reaction mixture added by syringe. After stirring the reaction mixture at 65 °C for 44 h, it was left to cool to 25 °C and diethylether (10 mL) was added. The diluted crude mixture was filtered through a plug of celite, eluting with further Et_2O (3 × 5 mL). The solvent was then removed *in vacuo* using a rotatory evaporator. Purification by flash column chromatography (0–50% Et_2O/CH_2Cl_2) afforded biaryl **33** as a colorless gum (14.8 mg, 78%). R_f = 0.24 (40% Et_2O/CH_2Cl_2); IR (film)/cm^{-1} 3439 (br, OH), 2960, 1354, 1117; ^{1}H NMR (500 MHz, $CDCl_3$) δ 9.10 (s, 2 H, 2 × NAr-CH), 7.71–7.66 (m, 4 H, 4 × Ar-CH), 4.34 (d, *J* = 12.4 Hz, 1 H, $C_qC\textit{H}HOCH_2$), 4.07 (d, *J* = 12.4 Hz, 1 H, $C_qCH\textit{H}OCH_2$), 3.81–3.67 (m, 6 H, 3 × OCH_2), 1.76 (br s, 1 H, OH); ^{13}C NMR (101 MHz, $CDCl_3$) δ 155.7 (2 × NAr-CH), 155.5 (q, $^2J_{C–F}$ = 37.0 Hz, Ar-$\textit{C}_qCF_3$), 141.6 (Ar-$\textit{C}_qC_q$), 135.7 (Ar-$C_{q(pyrimidine)}$), 132.3 (Ar-$\textit{C}_q$Ar-C_q), 128.3 (2 × Ar-CH), 127.6 (2 × Ar-CH), 119.7 (q, $^1J_{C–F}$ = 275.3 Hz, CF_3), 77.2 (C_q), 69.2 ($C_q\textit{C}H_2OCH_2$), 67.4 (OCH_2), 66.8 (OCH_2), 61.6 ($C_qO\textit{C}H_2$); ^{19}F NMR (377 MHz, CDCl3) δ –70.1; HRMS (APCI) *m/z* calcd for $C_{16}H_{16}F_3N_2O_3^+$ $[M+H]^+$: 341.1108, found: 341.1106.

Notes:

The C_q *signal in the ^{13}C NMR spectrum is hidden under the $CDCl_3$ peak but was assigned with HMBC correlations.*

The OH signal in the ^1H NMR spectrum sometimes appeared as a triplet (J = 6.6 Hz).

***33** was further characterized by X-ray crystallography (see Fig. S27).*

HPLC Traces of Enantioenriched 1,4-Dioxane 20

Racemic Mixture of 20
Conditions: Chiralpak IA 3-column, 90:10 *n*-hexane:*i*-PrOH, flow rate: 1 mL·min^{-1}, 35 ºC. UV detection wavelength: 270 nm.

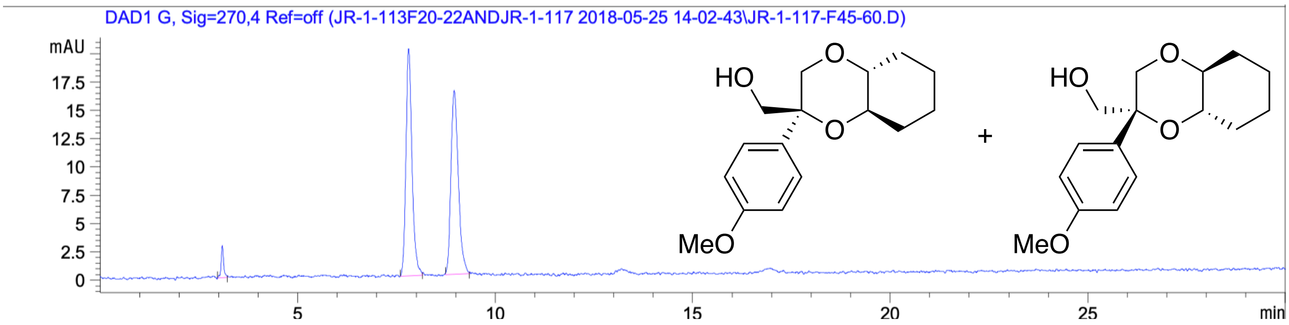

Signal 7: DAD1 G, Sig=270,4 Ref=off

Peak #	RetTime [min]	Type	Width [min]	Area [mAU*s]	Height [mAU]	Area %
1	3.094	BB	0.0640	11.98846	2.79678	2.7462
2	7.810	BB	0.1652	214.95476	20.03735	49.2397
3	8.961	BB	0.1970	209.60464	16.19790	48.0141

Totals : 436.54787 39.03203

((2*R*,4a*S*,8a*S*)-2-(4-Methoxyphenyl)octahydrobenzo[*b*][1,4]dioxin-2-yl)methanol ((–)-20)

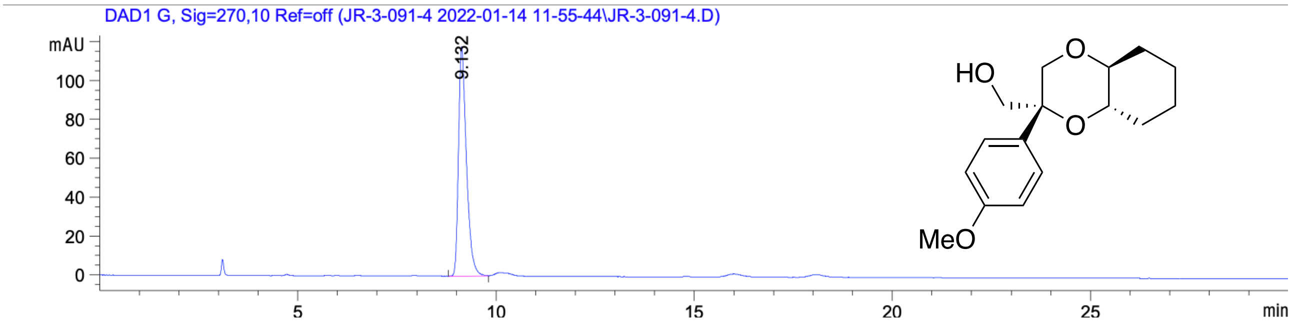

Signal 7: DAD1 G, Sig=270,10 Ref=off

Peak #	RetTime [min]	Type	Width [min]	Area [mAU*s]	Height [mAU]	Area %
1	9.132	BB	0.2108	1642.63428	117.75124	100.0000

Totals : 1642.63428 117.75124

***ee* = 100%**

X-Ray Crystallography Details

1,4-Dioxanes

Structural Analysis

X-Ray crystallography provides a means to assess the structural properties of novel compounds. This is a useful tool for drug discovery to predict the behavior of a specific motif in space, and in particular for fragment-based drug discovery to predict and/or model how a small fragment would fit and interact with a target of interest. The atomic coordinates derived from the X-ray structures provide an optimal starting point for such modellings. X-ray structures also served as unequivocal evidence for the assigned relative stereochemistry of diastereomeric products. Worth mentioning here is the unusual preference of the CH_2OH group for the equatorial position in the 1,4-dioxane products, leaving the supposedly bulkier aromatic substituent axial and thereby increasing the 3-dimensional character of the compounds.

X-Ray crystal structure of (2-(4-methoxyphenyl)-1,4-dioxan-2-yl)methanol (2)

Crystals suitable for X-ray analysis were grown by slow evaporation from $CDCl_3$ at 25 °C.

The structure of **2** was found to contain two crystallographically independent complexes (**2-A** and **2-B**) in the asymmetric unit.

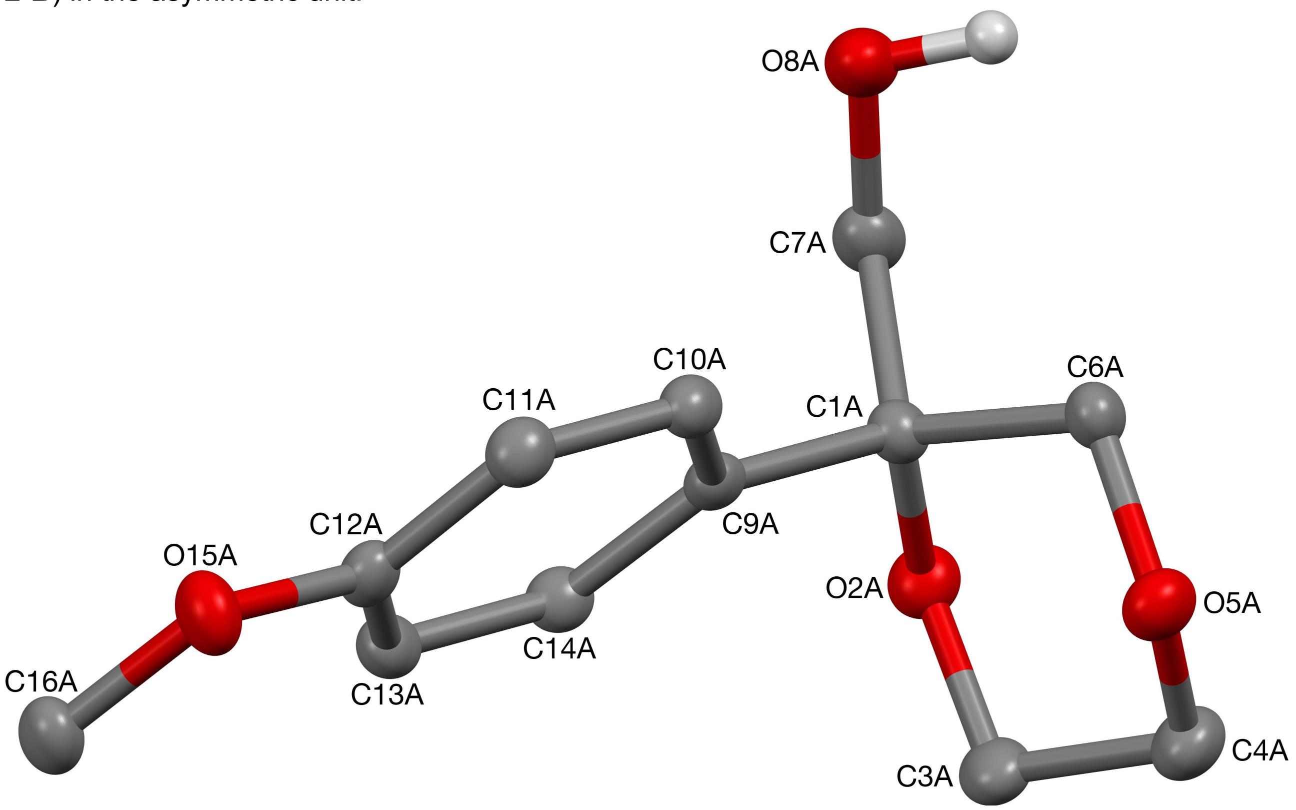

Fig. S20 The structure of **2-A**, one of the two independent molecules present in the crystal of **2-A** (50% probability ellipsoids).

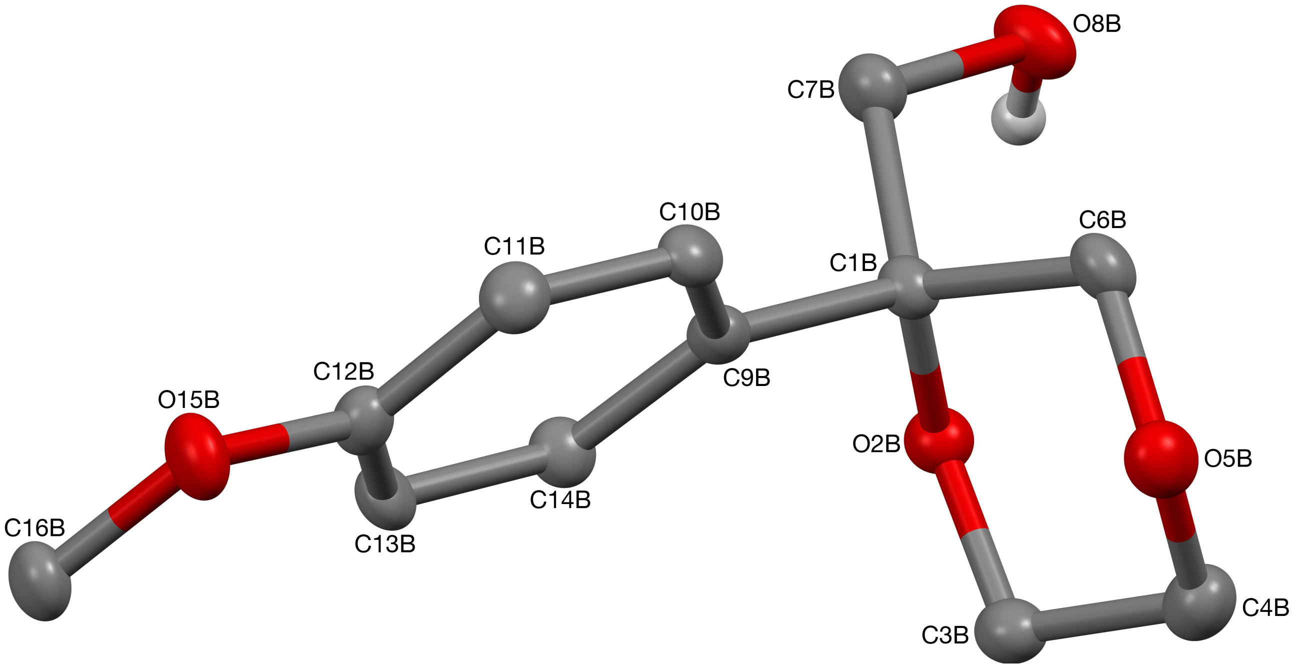

Fig. S21 The structure of **2-B**, one of the two independent molecules present in the crystal of **2-B** (50% probability ellipsoids).

Crystal data for **2**: $C_{12}H_{16}O_4$, M = 224.25, triclinic, P-1 (no. 14), a = 7.2623(5), b = 10.7162(6), c = 14.4316(11) Å, α = 104.526(6)°, β = 95.190(6)°, γ = 90.175(5)°, V = 1082.38(13) Å^3, Z = 4 [2 independent molecules], D_c = 1.376 g cm^{-3}, μ(Mo-Kα) = 0.103 mm^{-1}, T = 173 K, colorless blocks, Agilent Xcalibur 3 E diffractometer; 4277 independent measured reflections (R_{int} = 0.0193), F^2 refinement,[X1,X2] R_1(obs) = 0.0434, wR_2(all) = 0.1043 ($R_1 = \Sigma||F_o| - |F_c||/\Sigma|F_o|$; $wR_2 = \{\Sigma[w(F_o^2 - F_c^2)^2] / \Sigma[w(F_o^2)^2]\}^{1/2}$; $w^{-1} = \sigma^2(F_o^2) + (aP)^2 + bP$), 3282 independent observed absorption-corrected reflections [$|F_o| > 4\sigma(|F_o|)$, completeness to θ_{full}(25.2°) = 98.8% {completeness to 0.84 Å resolution}], 300 parameters. CCDC 2144680.

The O8–H hydrogen atom of each independent molecule was located from a ΔF map and refined freely subject to an O–H distance constraint of 0.90 Å.

X-Ray crystal structure of (3-(4-methoxyphenyl)-1,4-dioxaspiro[5.5]undecan-3-yl)methanol (16)

Crystals suitable for X-ray analysis were grown by slow evaporation from acetone at 25 °C.

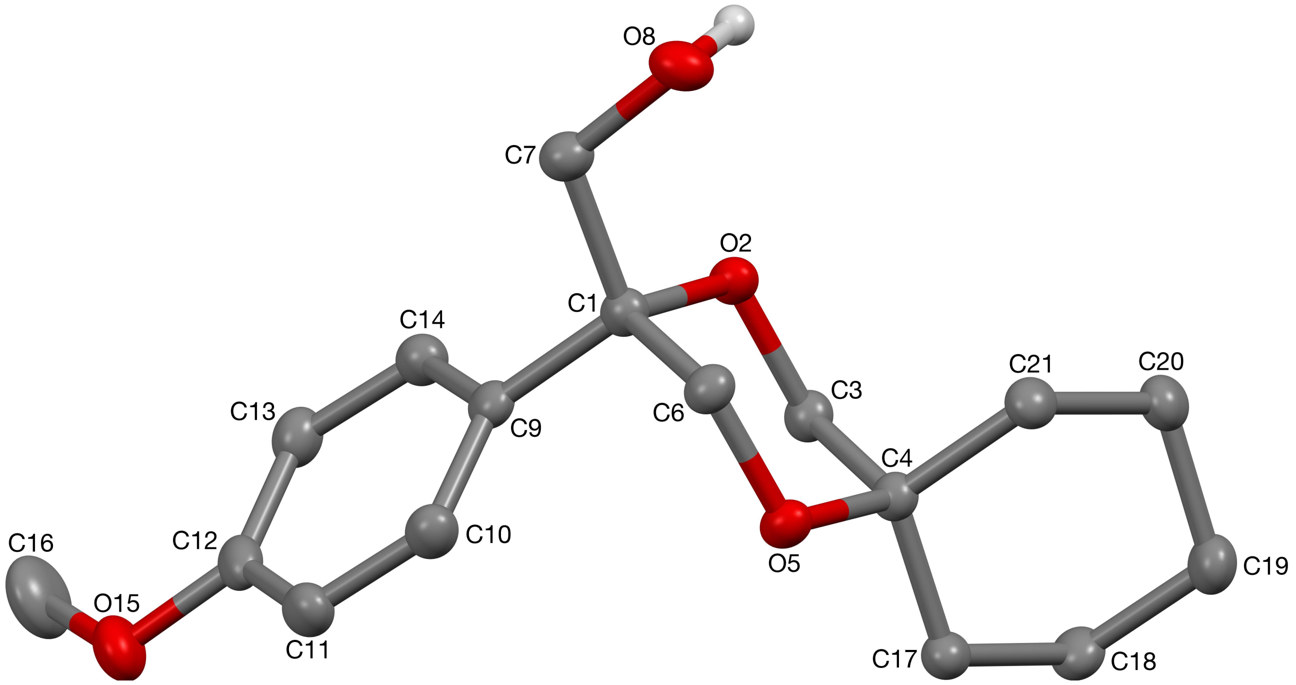

Fig. S22 X-Ray crystal structure of **16** (50% probability ellipsoids).

Crystal data for **16**: $C_{17}H_{24}O_4$, M = 292.36, monoclinic, $P2_1/c$ (no. 14), a = 12.1752(4), b = 11.6032(3), c = 11.2682(3) Å, β = 107.541(3)°, V = 1517.85(8) Å^3, Z = 4, D_c = 1.279 g cm^{-3}, μ(Mo-Kα) = 0.090 mm^{-1}, T = 173 K, colorless blocks, Agilent Xcalibur 3 E diffractometer; 3418 independent measured reflections (R_{int} = 0.0318), F^2 refinement,[X1,X2] R_1(obs) = 0.0393, wR_2(all) = 0.0953 ($R_1 = \Sigma||F_o| - |F_c||/\Sigma|F_o|$; $wR_2 = \{\Sigma[w(F_o^2 - F_c^2)^2] / \Sigma[w(F_o^2)^2]\}^{1/2}$; $w^{-1} = \sigma^2(F_o^2) + (aP)^2 + bP$), 2809 independent observed absorption-corrected reflections [$|F_o| > 4\sigma(|F_o|)$, completeness to θ_{full}(25.2°) = 100.0% {completeness to 0.84 Å resolution}], 196 parameters. CCDC 2144681.

The O8–H hydrogen atom was located from a ΔF map and refined freely subject to an O–H distance constraint of 0.90 Å.

X-Ray crystal structure of ((2*S*,5*R*,6*R*)-2-(4-methoxyphenyl)-5,6-dimethyl-1,4-dioxan-2-yl)methanol (18)

Crystals suitable for X-ray analysis were grown by slow evaporation from $CDCl_3$ at 25 °C.

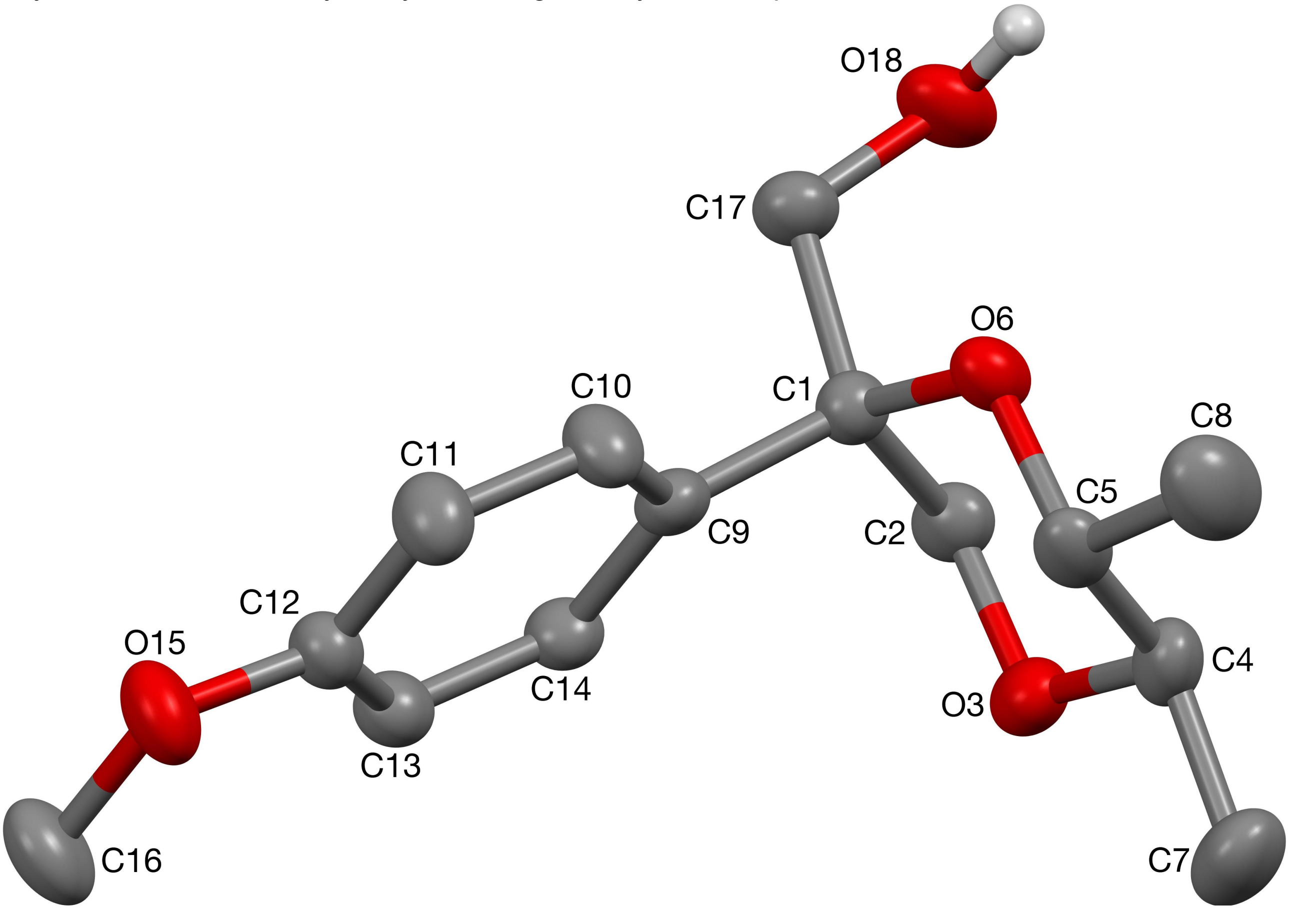

Fig. S23 X-Ray crystal structure of **18** (50% probability ellipsoids).

Crystal data for **18**: $C_{14}H_{20}O_4$, M = 252.30, monoclinic, $P2_1$ (no. 4), a = 6.2537(3), b = 7.8174(5), c = 13.8780(8) Å, β = 93.752(5)°, V = 677.02(7) Å^3, Z = 2, D_c = 1.238 g cm^{-3}, μ(Cu-Kα) = 0.735 mm^{-1}, T = 173 K, colorless platy needles, Agilent Xcalibur PX Ultra A diffractometer; 1774 independent measured reflections (R_{int} = 0.0352), F^2 refinement,[X1,X2] R_1(obs) = 0.0444, wR_2(all) = 0.1135 ($R_1 = \Sigma||F_o| - |F_c||/\Sigma|F_o|$; $wR_2 = \{\Sigma[w(F_o^2 - F_c^2)^2] / \Sigma[w(F_o^2)^2]\}^{1/2}$; $w^{-1} = \sigma^2(F_o^2) + (aP)^2 + bP$), 1410 independent observed absorption-corrected reflections [$|F_o| > 4\sigma(|F_o|)$, completeness to θ_{full}(25.2°) = 96.7% {completeness to 0.84 Å resolution}], 171 parameters. CCDC 2144682.

The O18–H hydrogen atom was located from a ΔF map and refined freely subject to an O–H distance constraint of 0.90 Å. The absolute structure of **18** could not be determined [Flack parameter x = 0.0(4)] and so arbitrary assignments were made.

X-Ray crystal structure of *all-cis*-(2-(4-methoxyphenyl)octahydrobenzo[*b*][1,4]dioxin-2-yl)methanol (21)

Crystals suitable for X-ray analysis were grown by slow evaporation from $CDCl_3$ at 25 °C.

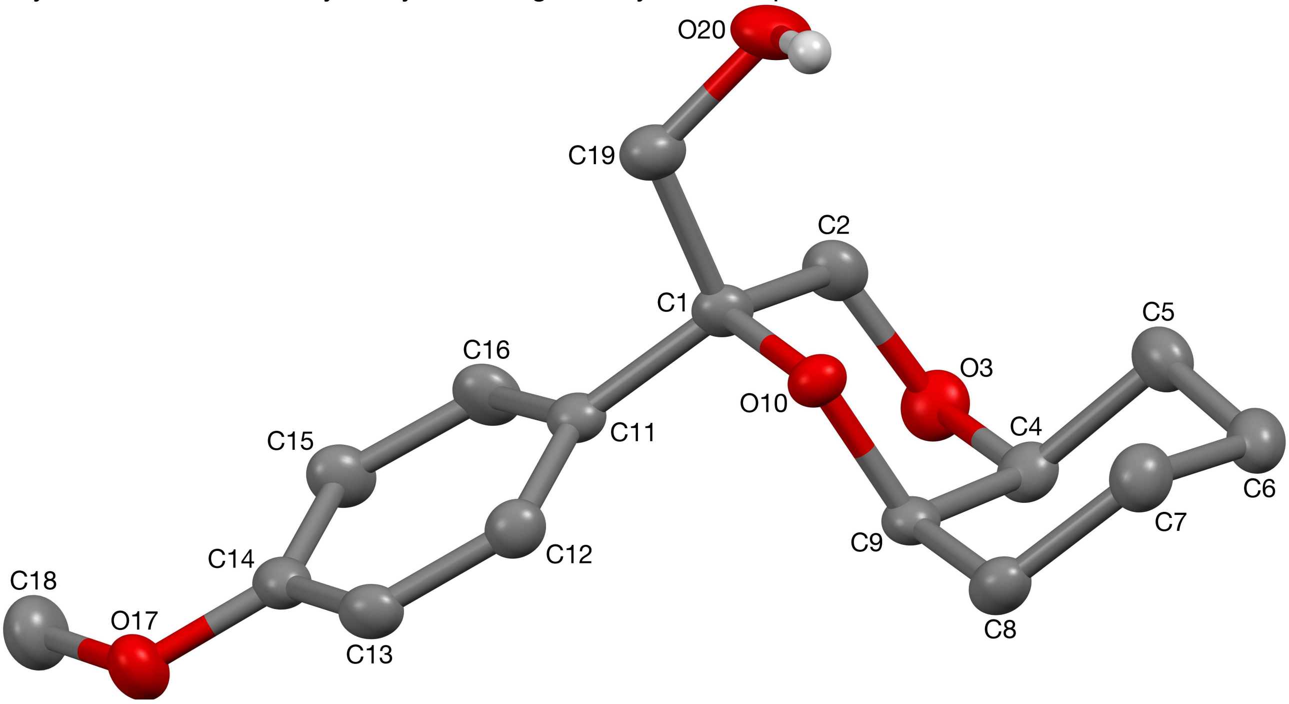

Fig. S24 X-Ray crystal structure of **21** (50% probability ellipsoids).

Crystal data for **21**: $C_{16}H_{22}O_4$, M = 278.33, monoclinic, $P2_1/c$ (no. 14), a = 11.1768(4), b = 11.2327(4), c = 11.4260(3) Å, β = 90.396(3)°, V = 1434.45(8) Å^3, Z = 4, D_c = 1.289 g cm^{-3}, μ(Mo-Kα) = 0.091 mm^{-1}, T = 173 K, colorless tablets, Agilent Xcalibur 3 E diffractometer; 3235 independent measured reflections (R_{int} = 0.0378), F^2 refinement,[X1,X2] R_1(obs) = 0.0429, wR_2(all) = 0.1106 ($R_1 = \Sigma||F_o| - |F_c||/\Sigma|F_o|$; $wR_2 = \{\Sigma[w(F_o^2 - F_c^2)^2] / \Sigma[w(F_o^2)^2]\}^{1/2}$; $w^{-1} = \sigma^2(F_o^2) + (aP)^2 + bP$), 2471 independent observed absorption-corrected reflections [$|F_o| > 4\sigma(|F_o|)$, completeness to θ_{full}(25.2°) = 99.9% {completeness to 0.84 Å resolution}], 186 parameters. CCDC 2144683.

The O20–H hydrogen atom was located from a ΔF map and refined freely subject to an O–H distance constraint of 0.90 Å.

X-Ray crystal structure of *cis-trans*-(2-(4-methoxyphenyl)octahydrobenzo[*b*][1,4]dioxin-2-yl)methanol (21')

Crystals suitable for X-ray analysis were grown by slow evaporation from acetone at 25 °C.

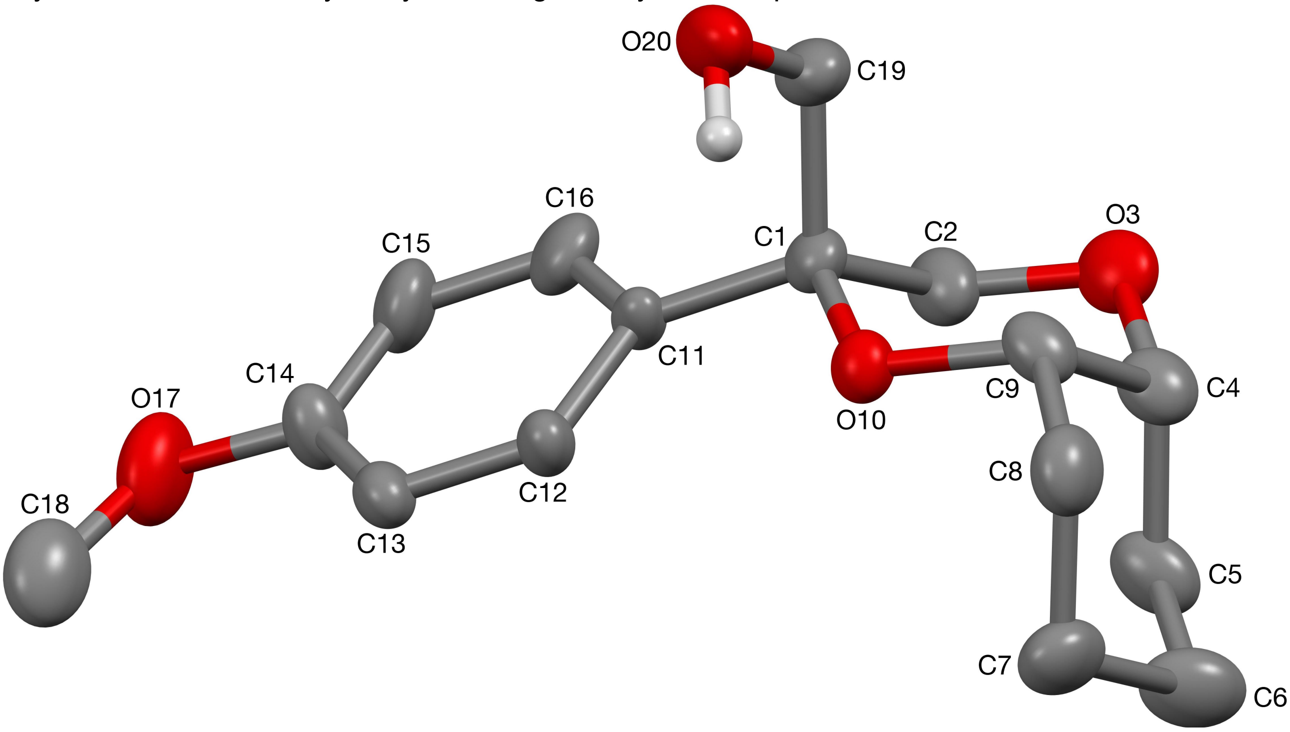

Fig. S25 X-Ray crystal structure of **21'** (50% probability ellipsoids).

Crystal data for **21'**: $C_{16}H_{22}O_4$, M = 278.33, orthorhombic, $Pna2_1$ (no. 33), a = 28.020(3), b = 6.0570(8), c = 8.4751(10) Å, V = 1438.4(3) Å^3, Z = 4, D_c = 1.285 g cm^{-3}, μ(Mo-Kα) = 0.091 mm^{-1}, T = 173 K, colorless plates, Agilent Xcalibur 3 E diffractometer; 2030 independent measured reflections (R_{int} = 0.0484), F^2 refinement,[X1,X2] R_1(obs) = 0.0561, wR_2(all) = 0.1297 ($R_1 = \Sigma||F_o| - |F_c||/\Sigma|F_o|$; $wR_2 = \{\Sigma[w(F_o^2 - F_c^2)^2] / \Sigma[w(F_o^2)^2]\}^{1/2}$; $w^{-1} = \sigma^2(F_o^2) + (aP)^2 + bP$), 1453 independent observed absorption-corrected reflections [$|F_o| > 4\sigma(|F_o|)$, completeness to θ_{full}(25.2°) = 99.4% {completeness to 0.84 Å resolution}], 187 parameters. CCDC 2144684.

The O20–H hydrogen atom was located from a ΔF map and refined freely subject to an O–H distance constraint of 0.90 Å. The absolute structure of **21'** could not be determined [Flack parameter x = 0.1(10)] and so arbitrary assignments were made.

X-Ray crystal structure of *trans-cis*-(2-(4-methoxyphenyl)hexahydro-5H-cyclopenta[b][1,4]dioxin-2-yl)methanol (23)

Crystals suitable for X-ray analysis were grown by slow evaporation from acetone at 25 °C.

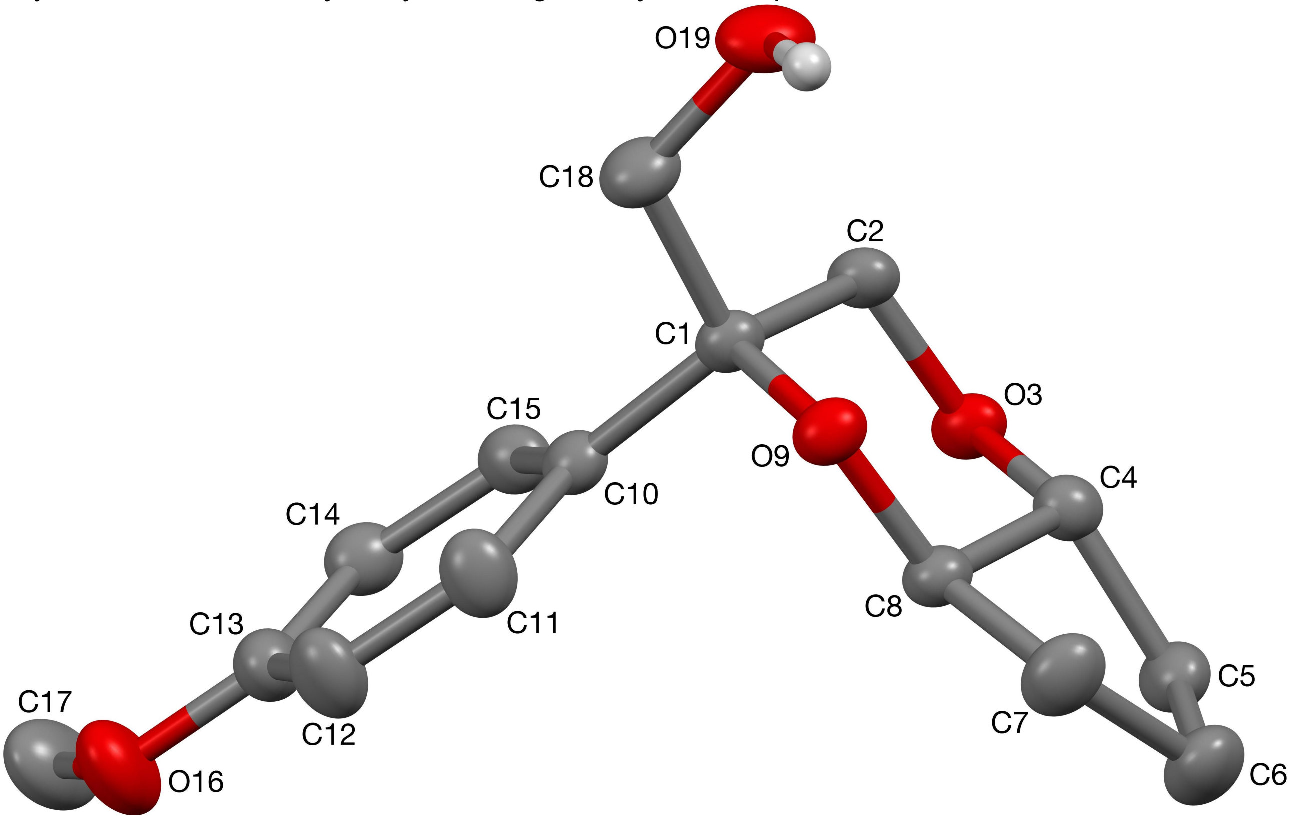

Fig. S26 X-Ray crystal structure of **23** (50% probability ellipsoids).

Crystal data for **23**: $C_{15}H_{20}O_4$, M = 264.31, monoclinic, $P2_1/c$ (no. 14), a = 6.1689(3), b = 7.8981(5), c = 28.1048(16) Å, β = 91.293(5)°, V = 1369.00(13) Å^3, Z = 4, D_c = 1.282 g cm^{-3}, μ(Mo-Kα) = 0.092 mm^{-1}, T = 173 K, colorless blocky needles, Agilent Xcalibur 3 E diffractometer; 3065 independent measured reflections (R_{int} = 0.0558), F^2 refinement,[X1,X2] R_1(obs) = 0.0805, wR_2(all) = 0.1625 ($R_1 = \Sigma||F_o| - |F_c||/\Sigma|F_o|$; $wR_2 = \{\Sigma[w(F_o^2 - F_c^2)^2] / \Sigma[w(F_o^2)^2]\}^{1/2}$; $w^{-1} = \sigma^2(F_o^2) + (aP)^2 + bP$), 2534 independent observed absorption-corrected reflections [$|F_o| > 4\sigma(|F_o|)$, completeness to θ_{full}(25.2°) = 100% {completeness to 0.84 Å resolution}], 178 parameters. CCDC 2144685.

The O19–H hydrogen atom was located from a ΔF map and refined freely subject to an O–H distance constraint of 0.90 Å.

X-Ray crystal structure of (2-(4-(2-(trifluoromethyl)pyrimidin-5-yl)phenyl)-1,4-dioxan-2-yl)methanol (33)

Crystals suitable for X-ray analysis were grown by slow evaporation from $CDCl_3$ at 25 °C.

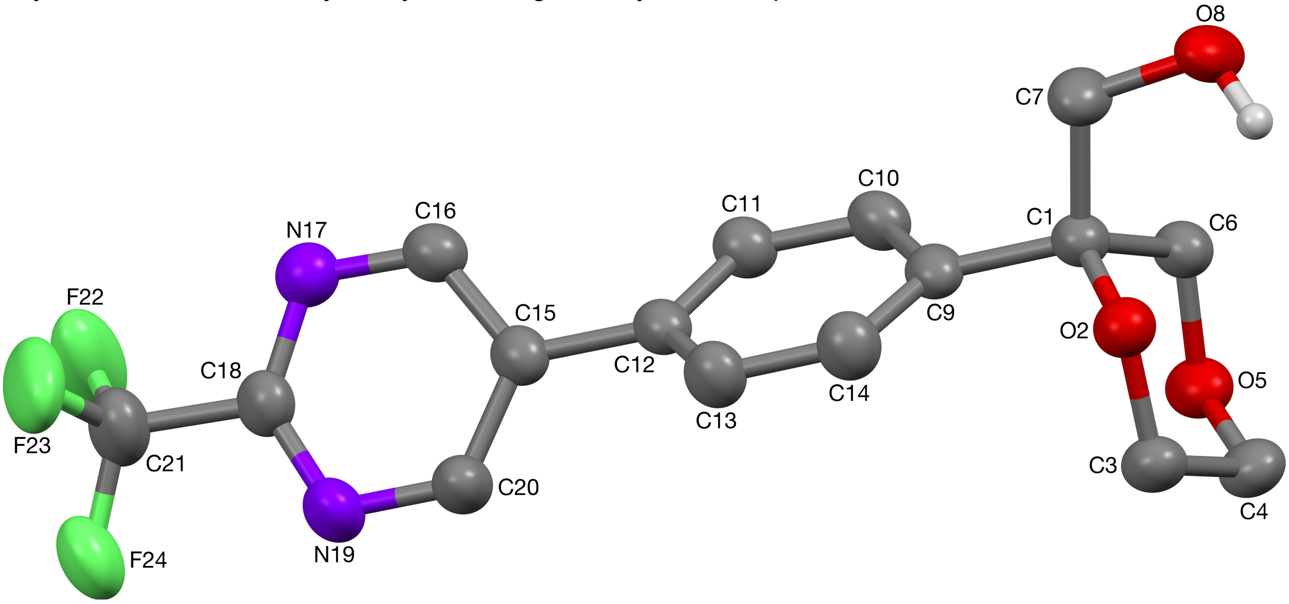

Fig. S27 X-Ray crystal structure of **33** (50% probability ellipsoids).

Crystal data for **33**: $C_{16}H_{15}F_3N_2O_3$, M = 340.30, monoclinic, $P2_1/c$ (no. 14), a = 11.1935(5), b = 15.8128(7), c = 8.8989(5) Å, β = 101.891(5)°, V = 1541.31(14) Å^3, Z = 4, D_c = 1.466 g cm^{-3}, μ(Cu-Kα) = 1.090 mm^{-1}, T = 173 K, colorless shards, Agilent Xcalibur PX Ultra A diffractometer; 3006 independent measured reflections (R_{int} = 0.0334), F^2 refinement,[X1,X2] R_1(obs) = 0.0457, wR_2(all) = 0.1250 ($R_1 = \Sigma||F_o| - |F_c||/\Sigma|F_o|$; $wR_2 = \{\Sigma[w(F_o^2 - F_c^2)^2] / \Sigma[w(F_o^2)^2]\}^{1/2}$; $w^{-1} = \sigma^2(F_o^2) + (aP)^2 + bP$), 2322 independent observed absorption-corrected reflections [$|F_o| > 4\sigma(|F_o|)$, completeness to θ_{full}(25.2°) = 99.3% {completeness to 0.84 Å resolution}], 255 parameters. CCDC 2144686.

The C1-based $C_4H_6O_2$ ring and the C7-based CH_2OH substituent in the structure of **33** were found to be disordered. Two orientations were identified of *ca*. 92 and 8% occupancy, their geometries were optimized, the thermal parameters of adjacent atoms were restrained to be similar, and only the non-hydrogen atoms of the major occupancy orientation were refined anisotropically (those of the minor occupancy orientation were refined isotropically). The O8–H hydrogen atom of the major occupancy orientation was located from a ΔF map and refined freely subject to an O–H distance constraint of 0.90 Å. The O8–H hydrogen atom of the minor occupancy orientation could not be located and so was added in an idealized position with an O–H distance of 0.90 Å and allowed to rotate about the C–O vector to find the best fit with the observed electron density (the SHELX HFIX/AFIX 147 command).

Oxetane Sulfide S8

X-Ray crystal structure of (S8)

Crystals suitable for X-ray analysis were grown by slow evaporation from Et_2O at 25 °C.

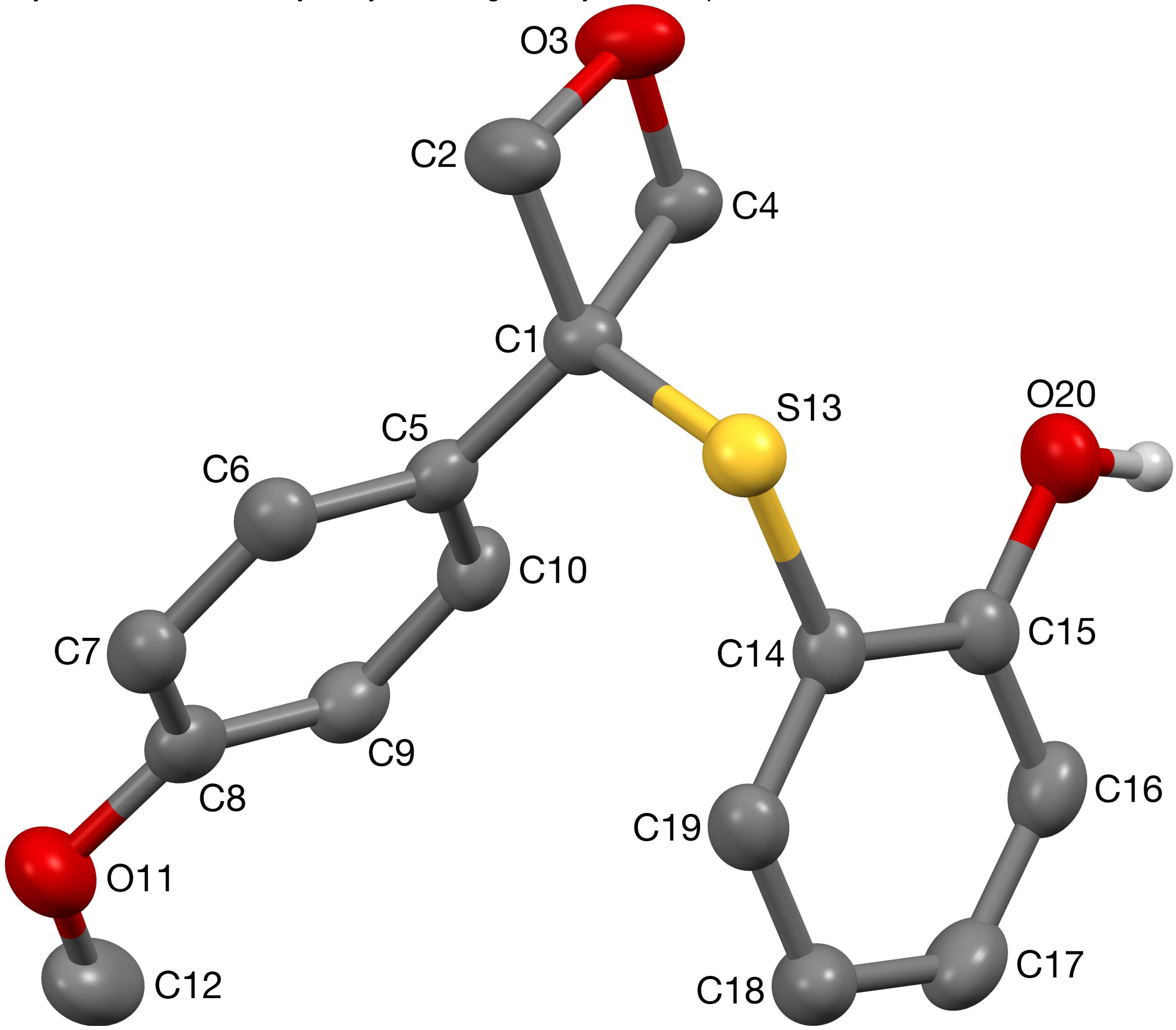

Fig. S28 X-Ray crystal structure of **S8** (50% probability ellipsoids).

Crystal data for **S8**: $C_{16}H_{16}O_3S$, M = 288.35, orthorhombic, $P2_12_12_1$ (no. 19), a = 5.5987(3), b = 11.9892(5), c = 21.3186(9) Å, V = 1430.99(11) Å^3, Z = 4, D_c = 1.338 g cm^{-3}, μ(Cu-Kα) = 2.049 mm^{-1}, T = 173 K, colorless needles, Agilent Xcalibur PX Ultra A diffractometer; 2193 independent measured reflections (R_{int} = 0.0483), F^2 refinement,[X1,X2] R_1(obs) = 0.0468, wR_2(all) = 0.1139 ($R_1 = \Sigma||F_o| - |F_c||/\Sigma|F_o|$; $wR_2 = \{\Sigma[w(F_o^2 - F_c^2)^2] / \Sigma[w(F_o^2)^2]\}^{1/2}$; $w^{-1} = \sigma^2(F_o^2) + (aP)^2 + bP$), 1860 independent observed absorption-corrected reflections [$|F_o| > 4\sigma(|F_o|)$, completeness to θ_{full}(25.2°) = 98.3% {completeness to 0.84 Å resolution}], 186 parameters. CCDC 2144687.

The absolute structure of **S8** was determined by use of the Flack parameter [x = –0.06(4)]. The O20–H hydrogen atom in the structure of **S8** was located from a ΔF map and refined freely subject to an O–H distance constraint of 0.90 Å.

Most relevant bond lengths and angles:

Bond lengths

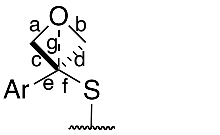

a = C(2)–O(3) = 1.44 Å
b = C(4)–O(3) = 1.45 Å
c = C(1)–C(2) = 1.54 Å
d = C(1)–C(4) = 1.53 Å
e = C(1)–C(5) = 1.50 Å
f = C(1)–S(13) = 1.84 Å
g[a] = C(1)–O(3) = 2.14 Å

Bond angles

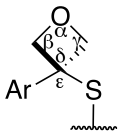

α = C(2)–O(3)–C(4) = 91.0 °
β = O(3)–C(2)–C(1) = 91.7 °
γ = O(3)–C(4)–C(1) = 91.7 °
δ = C(2)–C(1)–C(4) = 84.5 °
ε = S(13)–C(1)–C(5) = 111.5 °

Torsion angles

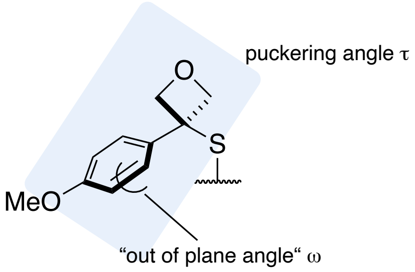

τ = O(3)–C(2)–C(4)–C(1) = 8.3 °
ω = O(3)–C(1)–C(11)–C(16) = 87.7 °

Fig. S29 Most relevant bond lengths and angles in the crystal structure of **S8**. [a]g is the through-space distance between C(1) and O(3), not a bond length. See Fig. S2 for further details.

References

[X1] SHELXTL v5.1, Bruker AXS, Madison, WI, 1998.
[X2] SHELX-2013, G.M. Sheldrick, *Acta Cryst.*, 2015, **C71**, 3-8.

^{1}H, ^{13}C and ^{19}F NMR Spectra of Selected Compounds

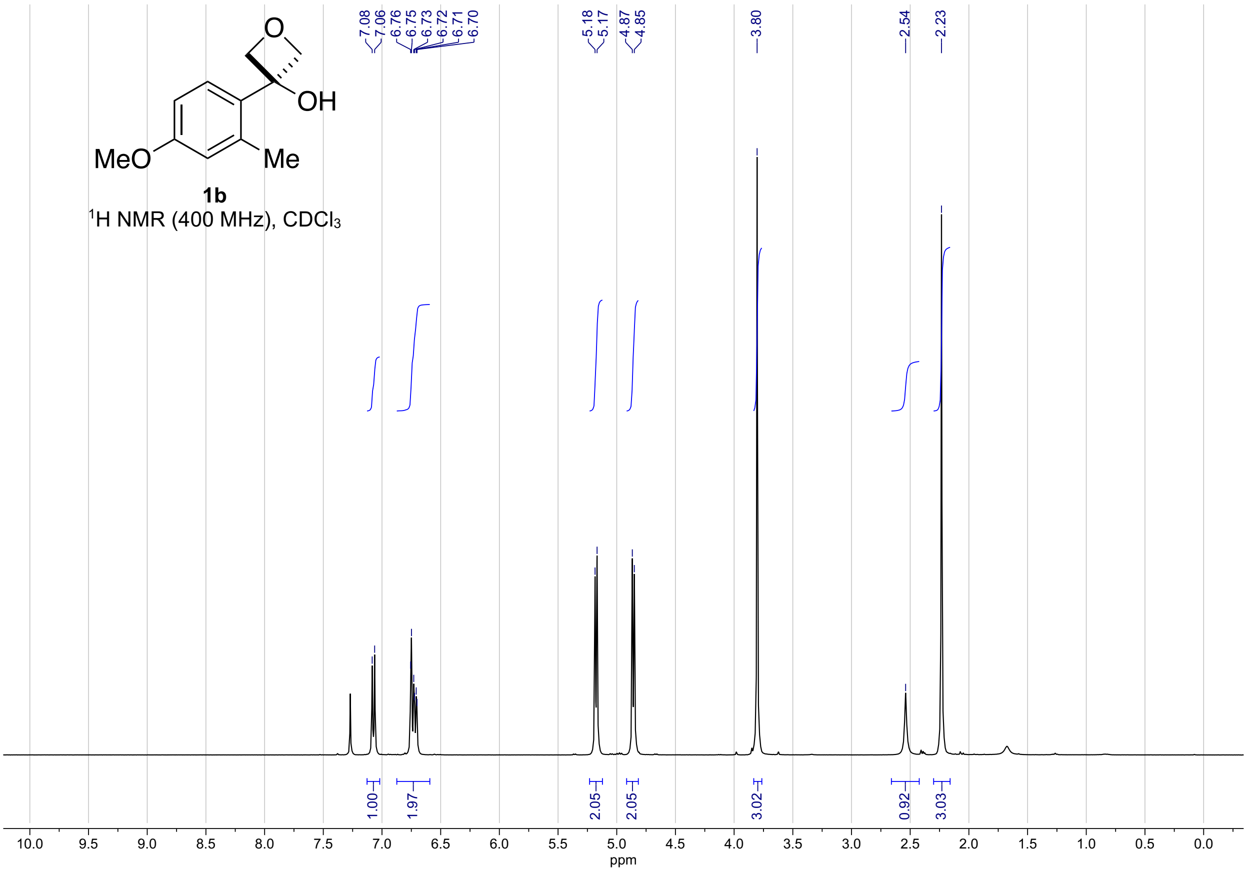
7.08
7.06
6.76
6.75
6.73
6.72
6.71
6.70
5.18
5.17
4.87
4.85
3.80
2.54
2.23
O
OH
MeO
Me
1b
¹H NMR (400 MHz), CDCl₃
1.00
1.97
2.05
2.05
3.02
0.92
3.03
ppm

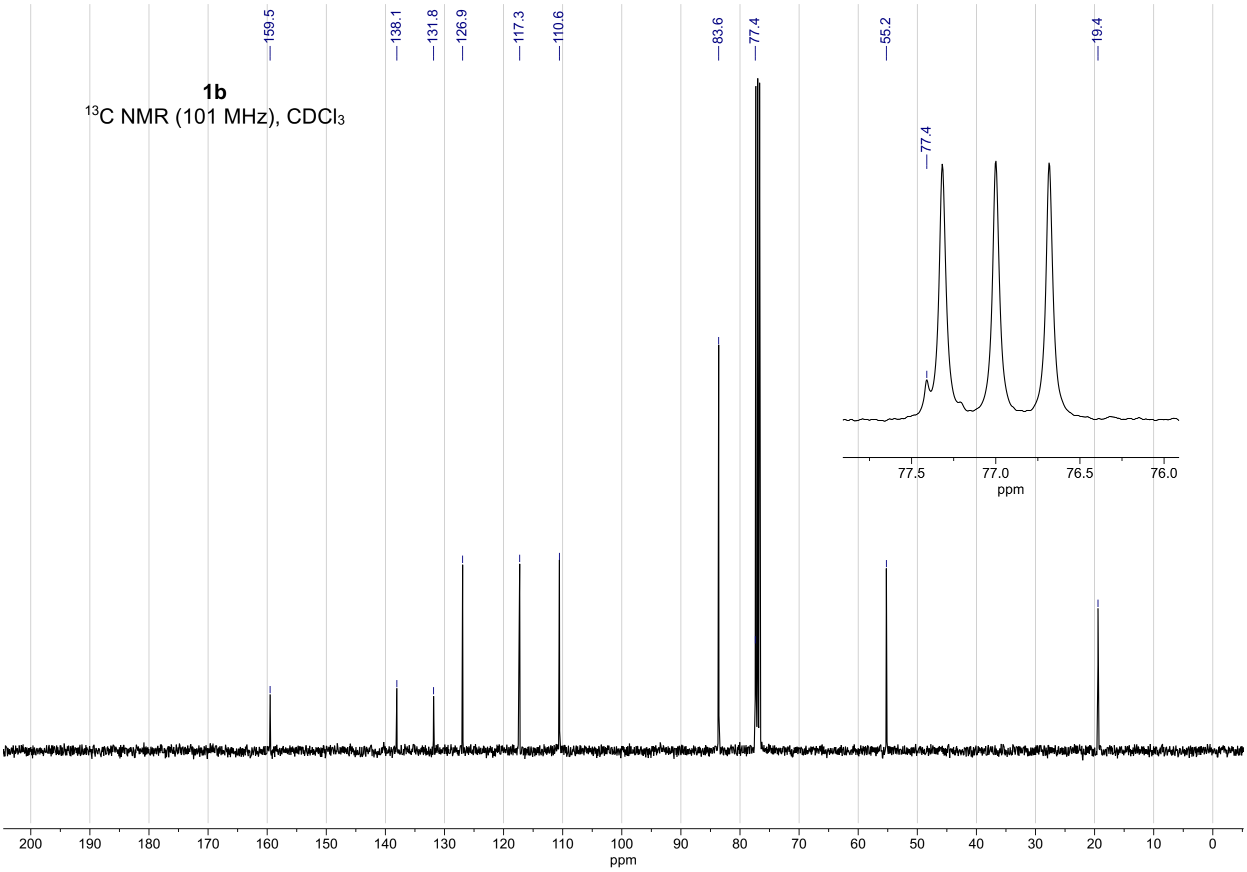
159.5
138.1
131.8
126.9
117.3
110.6
83.6
77.4
55.2
19.4
1b
¹³C NMR (101 MHz), CDCl₃
77.4
ppm
ppm

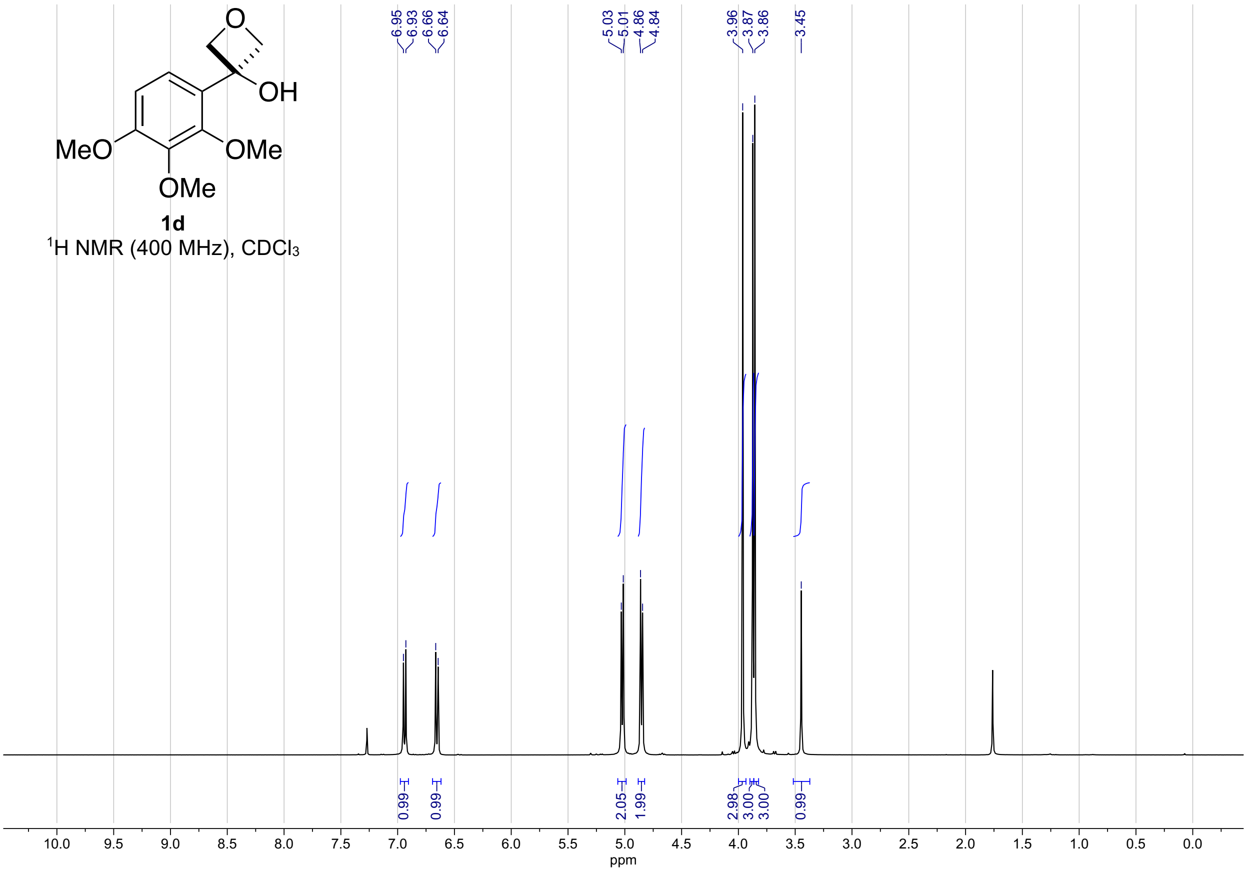
OH
MeO
OMe
OMe
1d
^{1}H NMR (400 MHz), CDCl$_3$
6.95
6.93
6.66
6.64
5.03
5.01
4.86
4.84
3.96
3.87
3.86
3.45
0.99
0.99
2.05
1.99
2.98
3.00
3.00
0.99
10.0 9.5 9.0 8.5 8.0 7.5 7.0 6.5 6.0 5.5 5.0 4.5 4.0 3.5 3.0 2.5 2.0 1.5 1.0 0.5 0.0
ppm

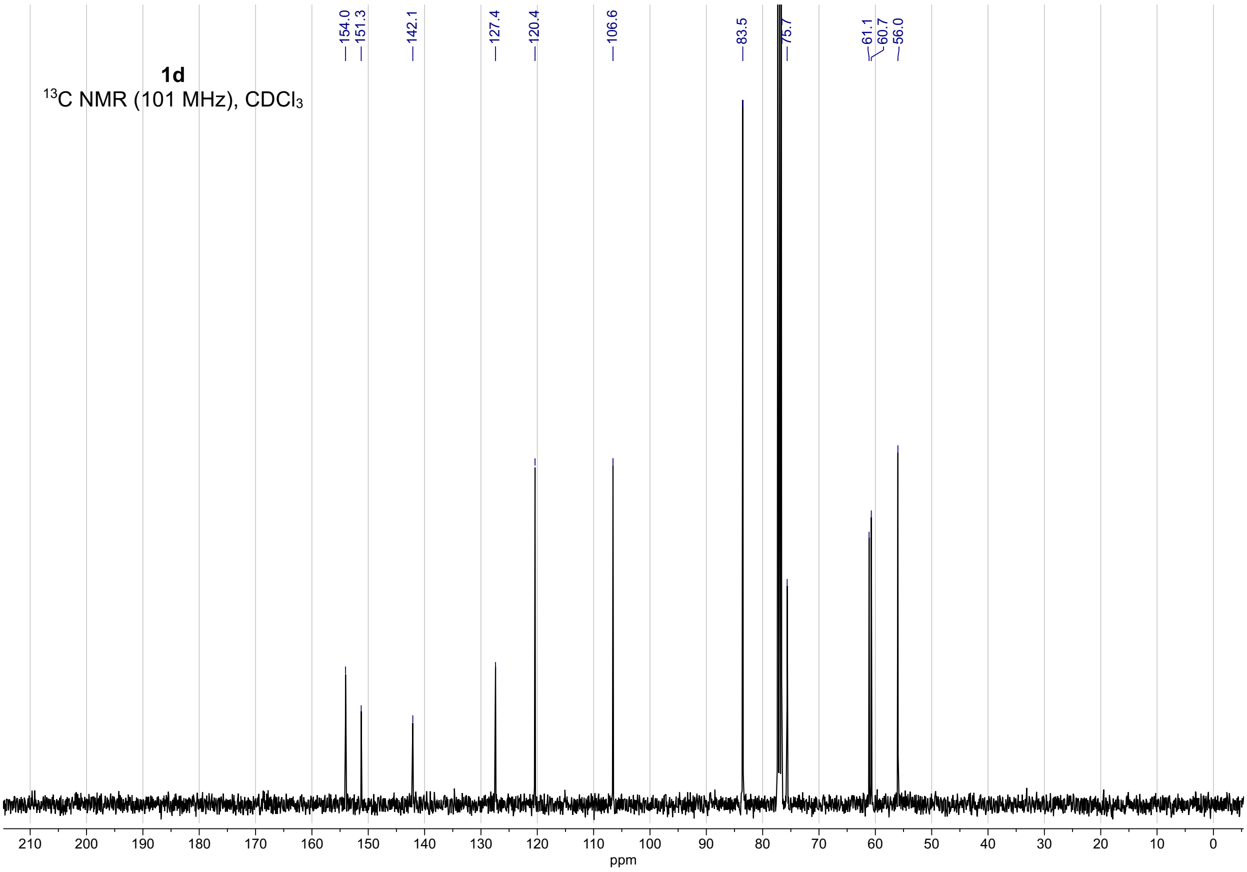
1d
^{13}C NMR (101 MHz), CDCl$_3$
154.0
151.3
142.1
127.4
120.4
106.6
83.5
75.7
61.1
60.7
56.0
210 200 190 180 170 160 150 140 130 120 110 100 90 80 70 60 50 40 30 20 10 0
ppm

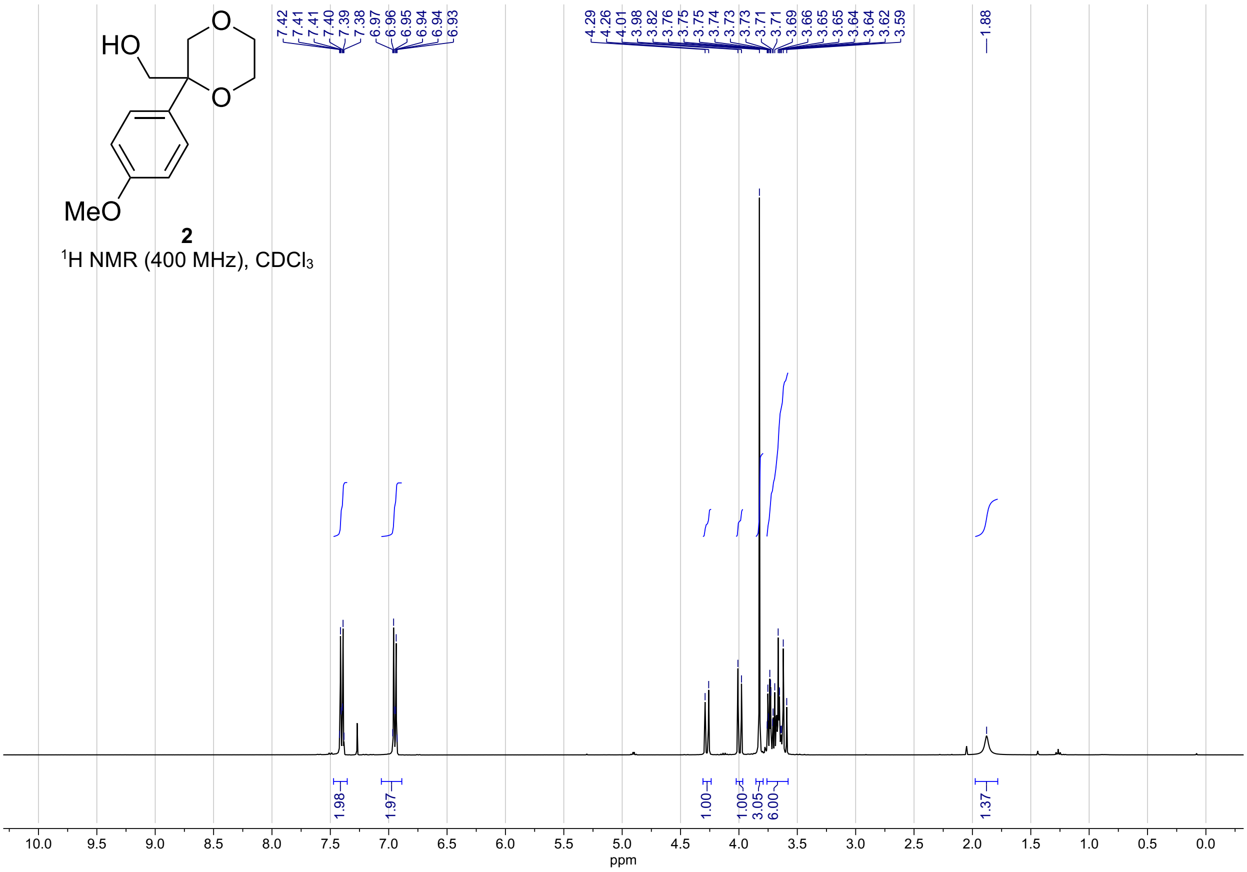
HO
O
O
MeO
2
1H NMR (400 MHz), CDCl3
7.42
7.41
7.41
7.40
7.39
7.38
6.97
6.96
6.95
6.94
6.94
6.93
4.29
4.26
4.01
3.98
3.82
3.76
3.75
3.75
3.74
3.73
3.73
3.71
3.71
3.69
3.66
3.65
3.65
3.64
3.64
3.62
3.59
1.88
1.98
1.97
1.00
1.00
3.05
6.00
1.37
10.0
9.5
9.0
8.5
8.0
7.5
7.0
6.5
6.0
5.5
5.0
4.5
4.0
3.5
3.0
2.5
2.0
1.5
1.0
0.5
0.0
ppm

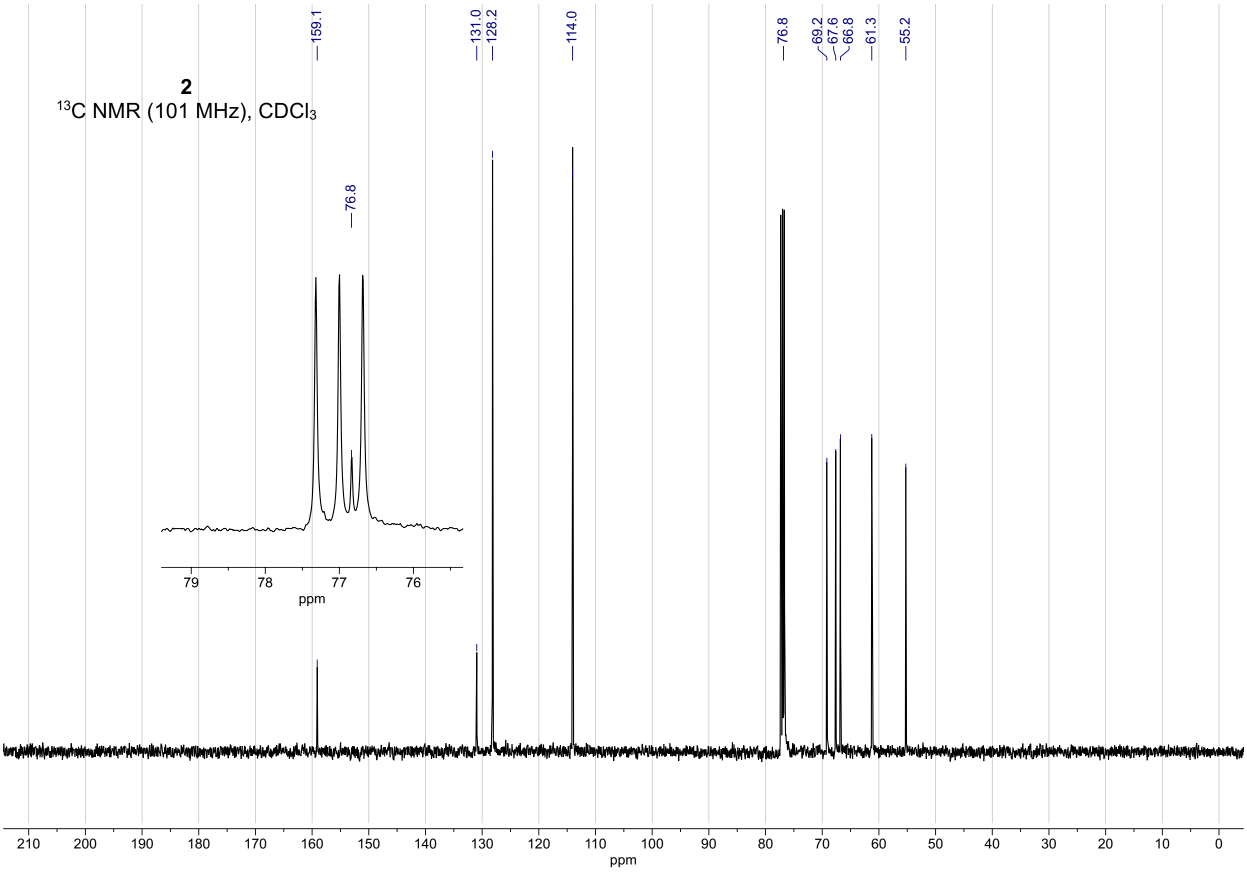
2
13C NMR (101 MHz), CDCl3
159.1
131.0
128.2
114.0
76.8
69.2
67.6
66.8
61.3
55.2
76.8
79
78
77
76
ppm
210
200
190
180
170
160
150
140
130
120
110
100
90
80
70
60
50
40
30
20
10
0
ppm

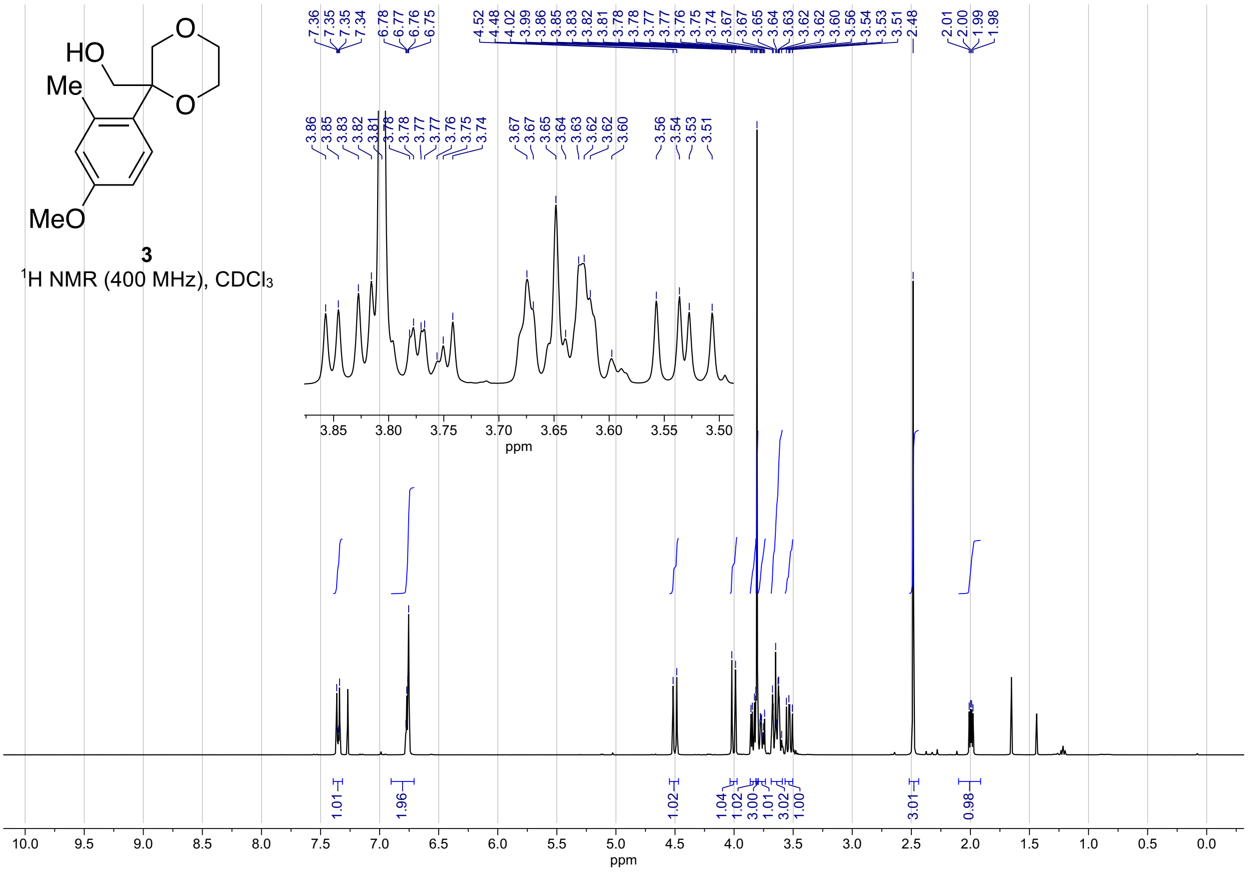

3
^{1}H NMR (400 MHz), $CDCl_3$

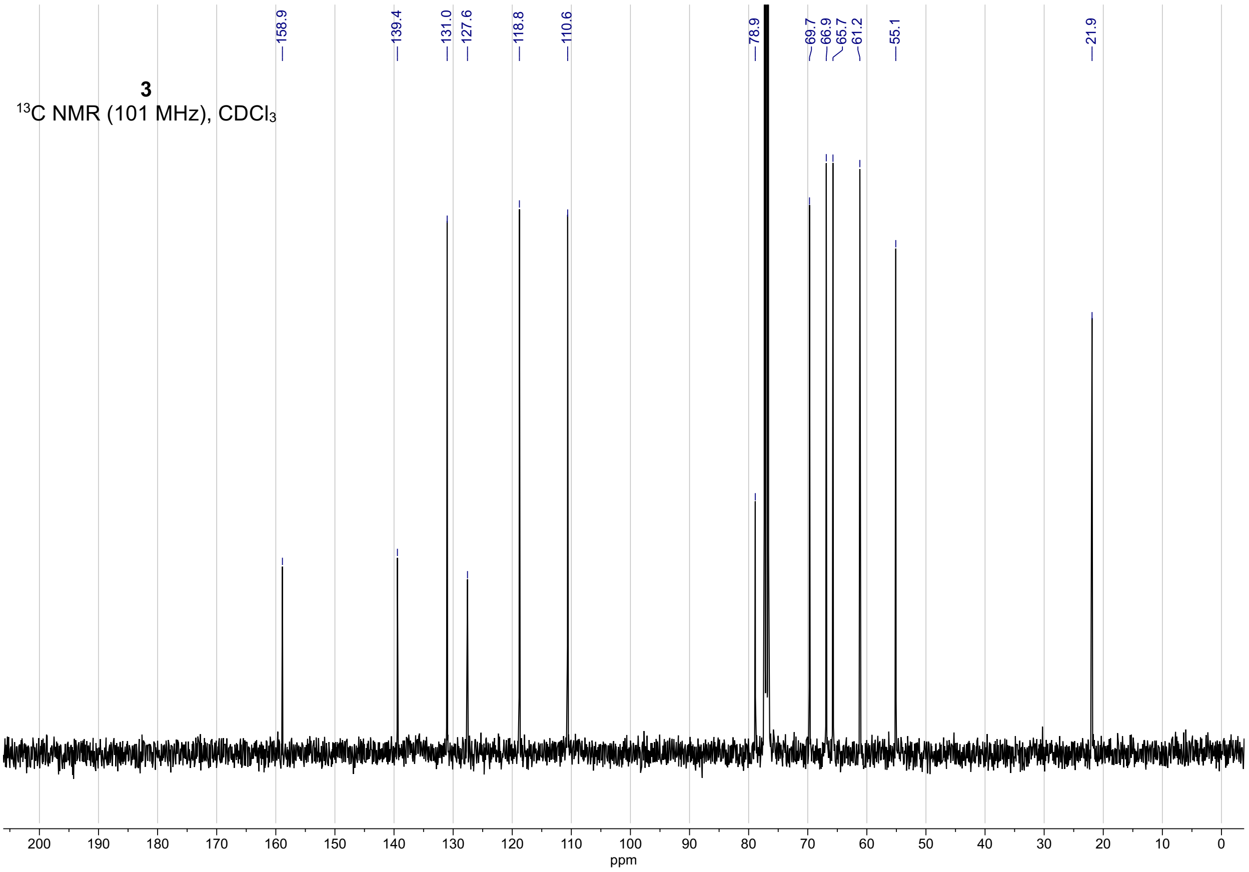

3
^{13}C NMR (101 MHz), $CDCl_3$

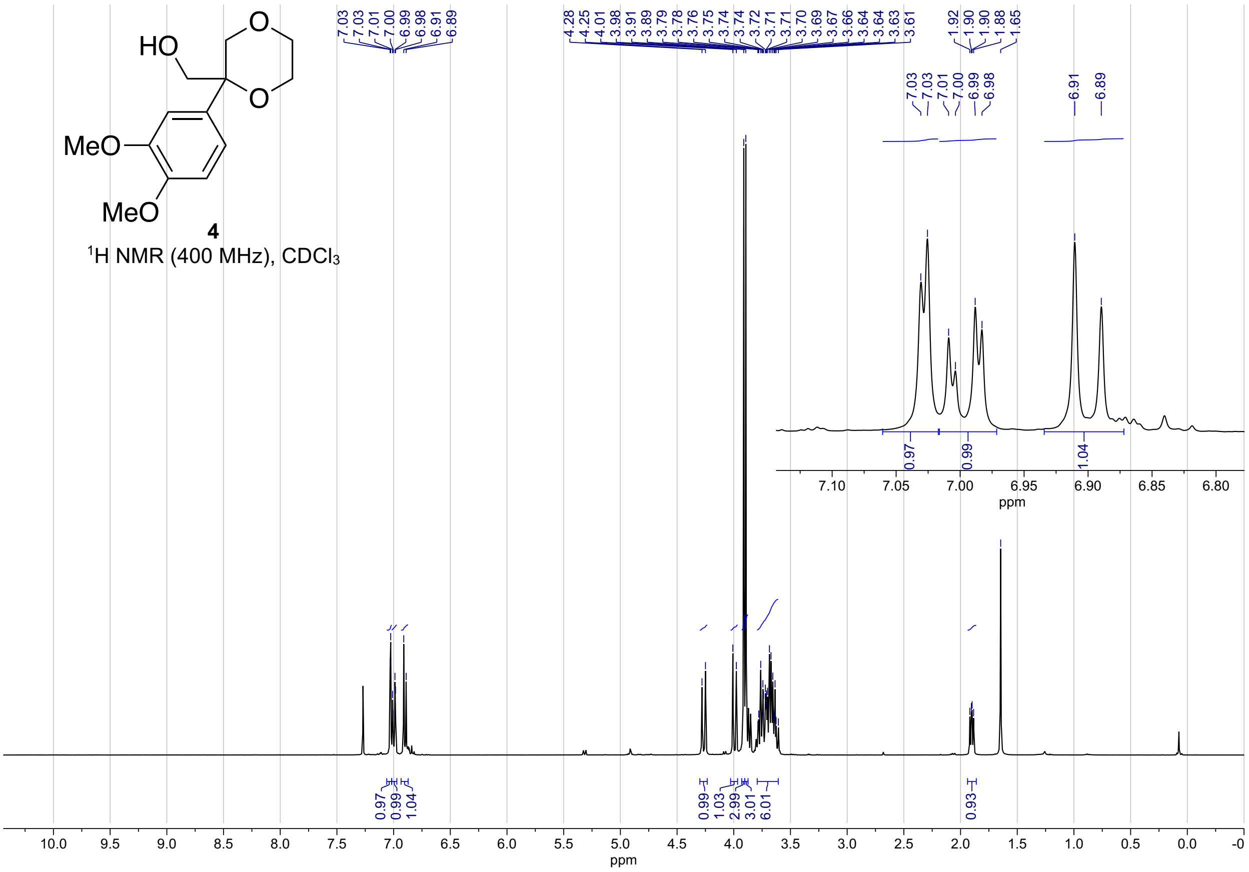

4
^{1}H NMR (400 MHz), $CDCl_3$

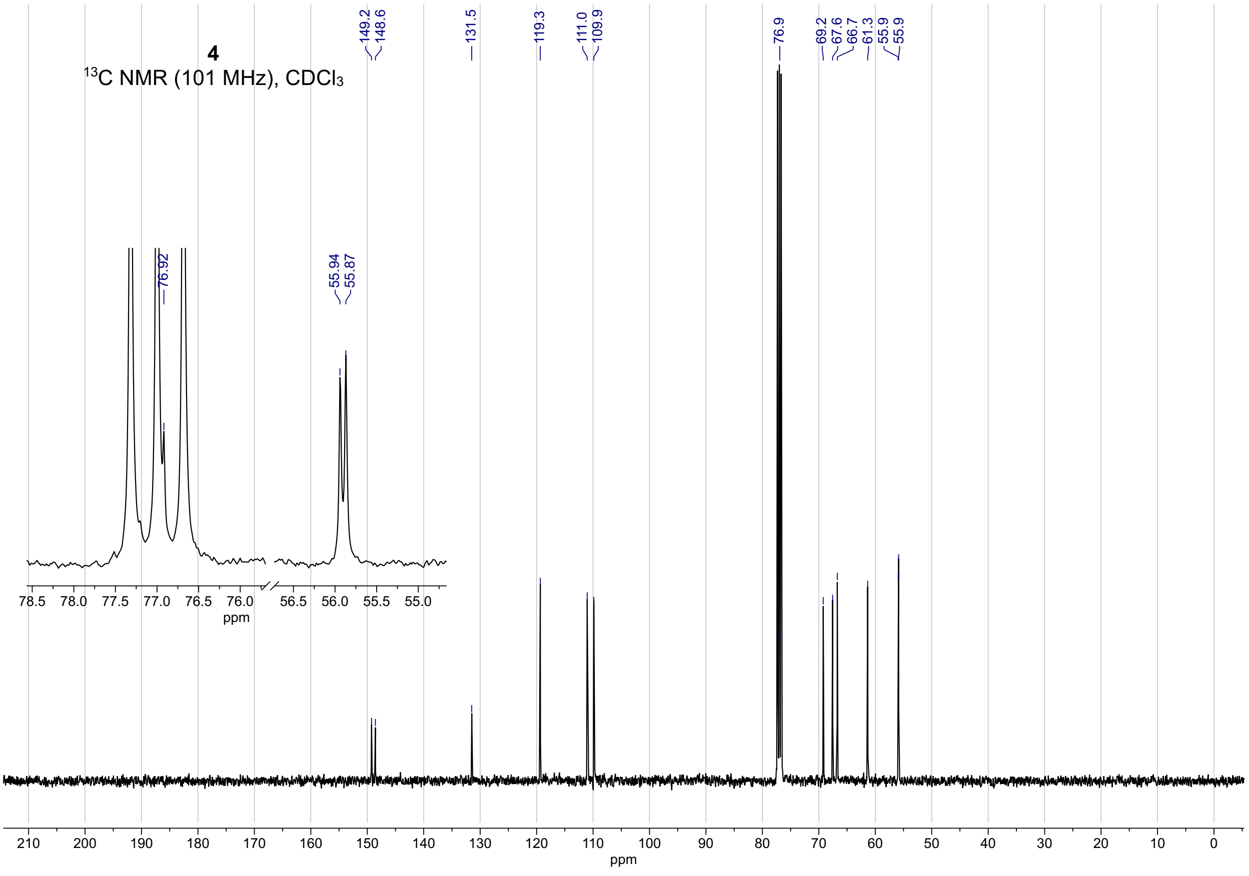

4
^{13}C NMR (101 MHz), $CDCl_3$

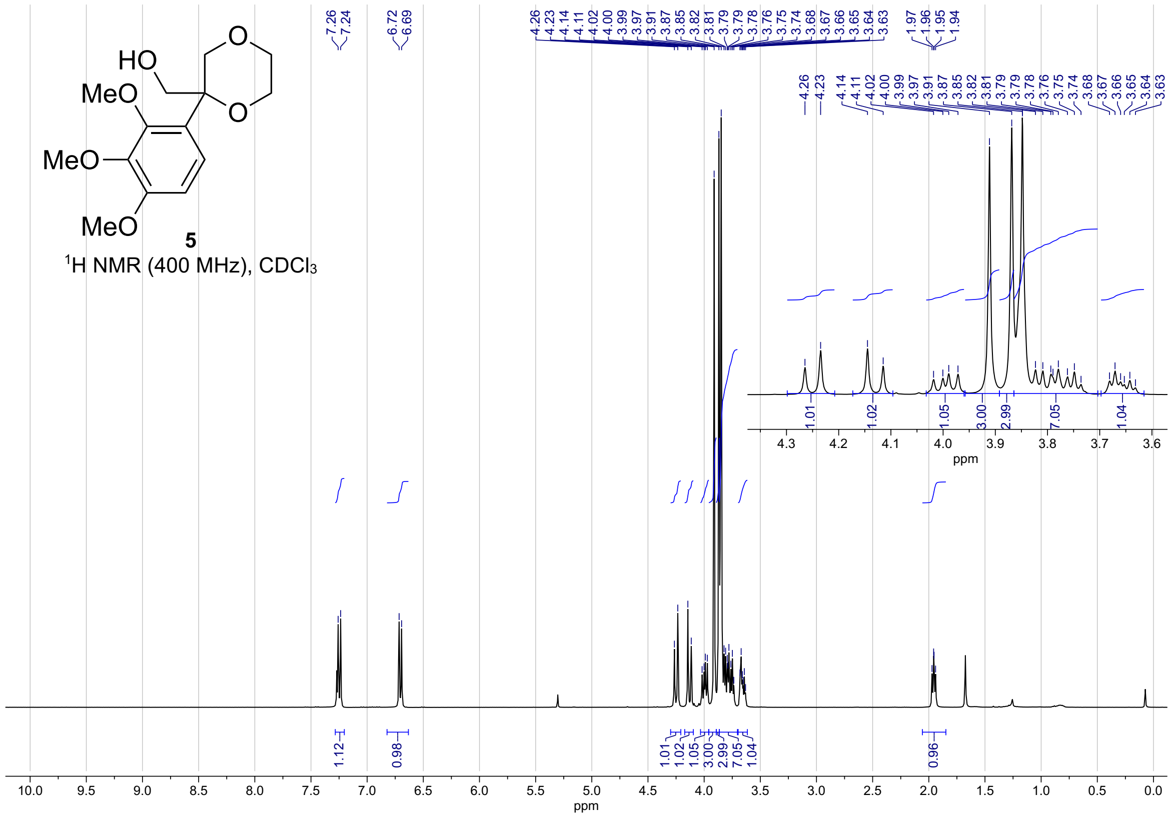
HO
MeO
MeO
MeO
O
O
5
^{1}H NMR (400 MHz), CDCl$_3$
ppm

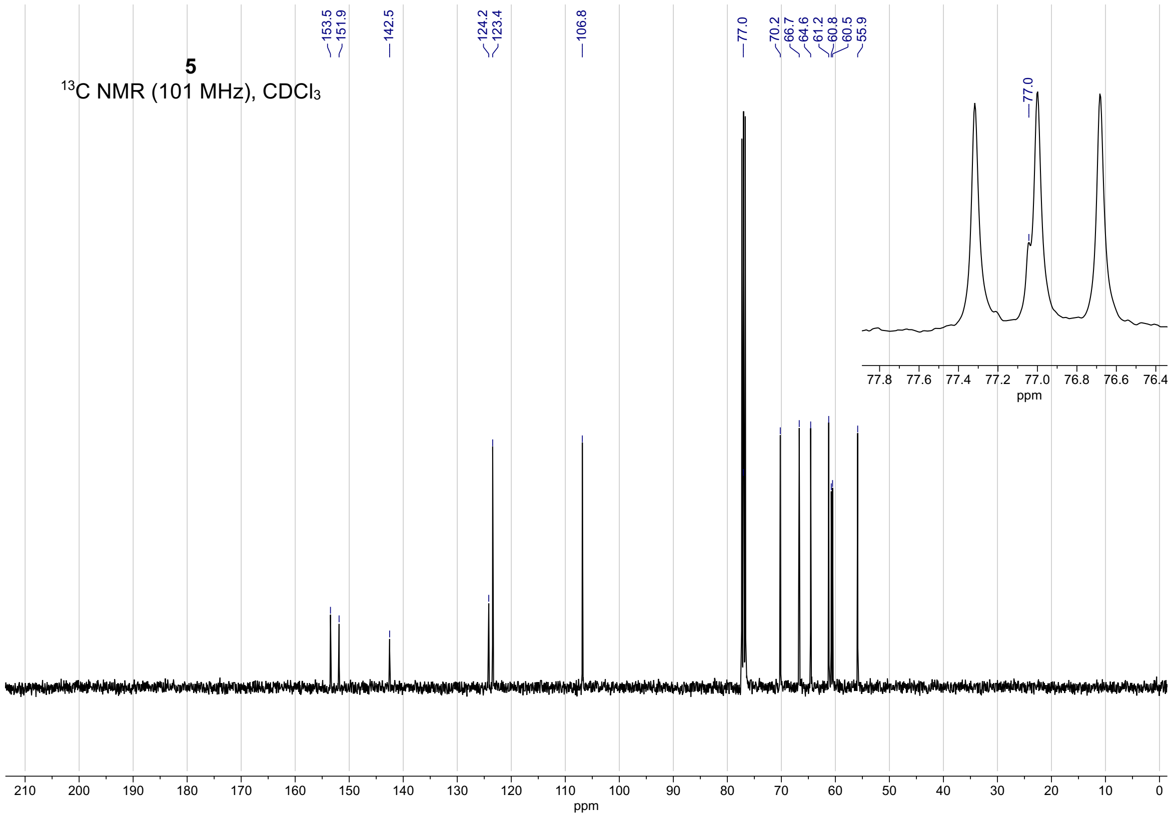
5
^{13}C NMR (101 MHz), CDCl$_3$
153.5
151.9
142.5
124.2
123.4
106.8
77.0
70.2
66.7
64.6
61.2
60.8
60.5
55.9
ppm

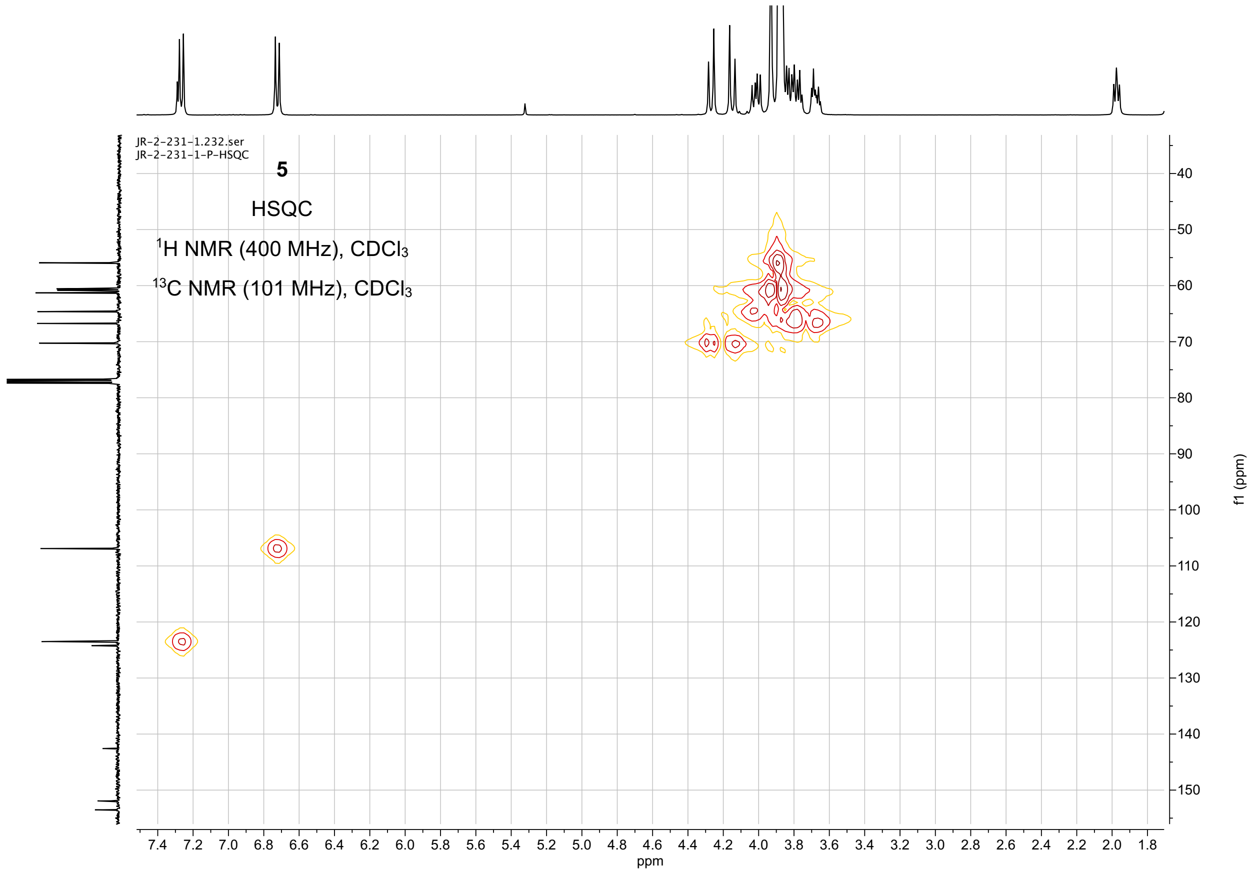
JR-2-231-1.232.ser
JR-2-231-1-P-HSQC
5
HSQC
^{1}H NMR (400 MHz), CDCl3
^{13}C NMR (101 MHz), CDCl3
ppm
f1 (ppm)

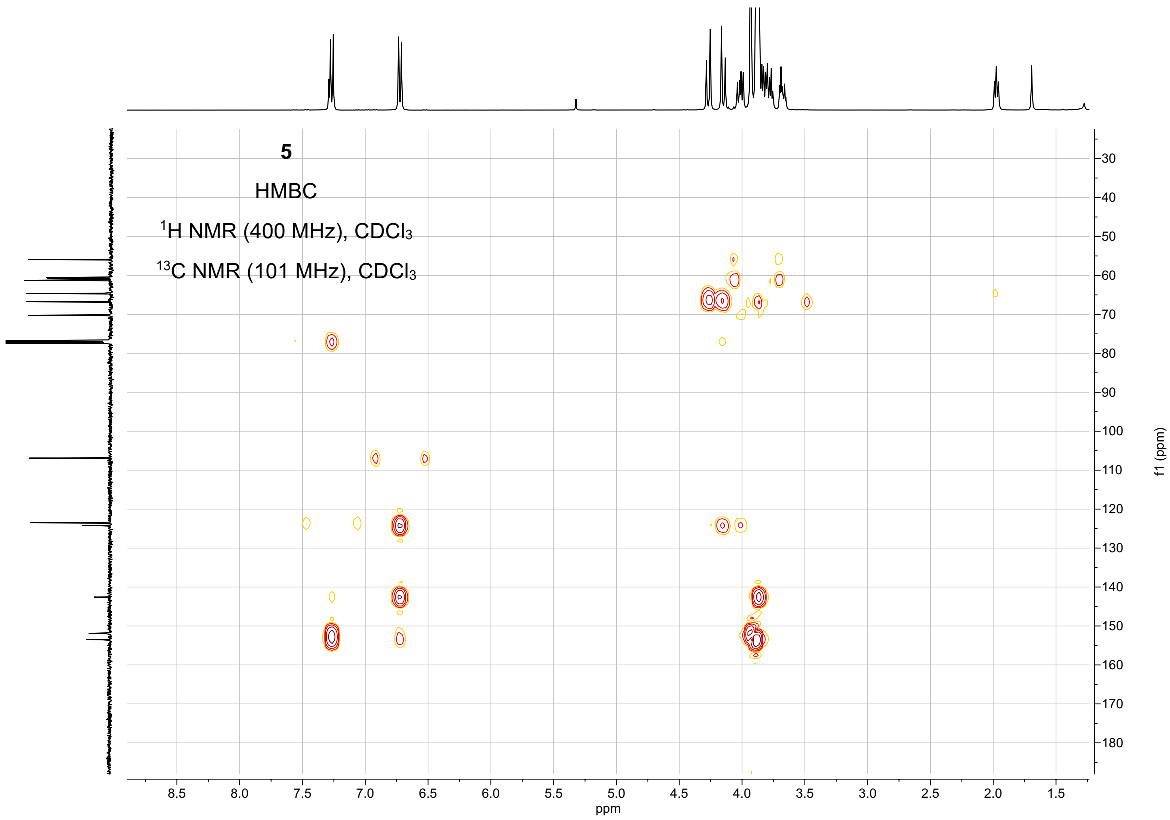
5
HMBC
^{1}H NMR (400 MHz), CDCl3
^{13}C NMR (101 MHz), CDCl3
ppm
f1 (ppm)

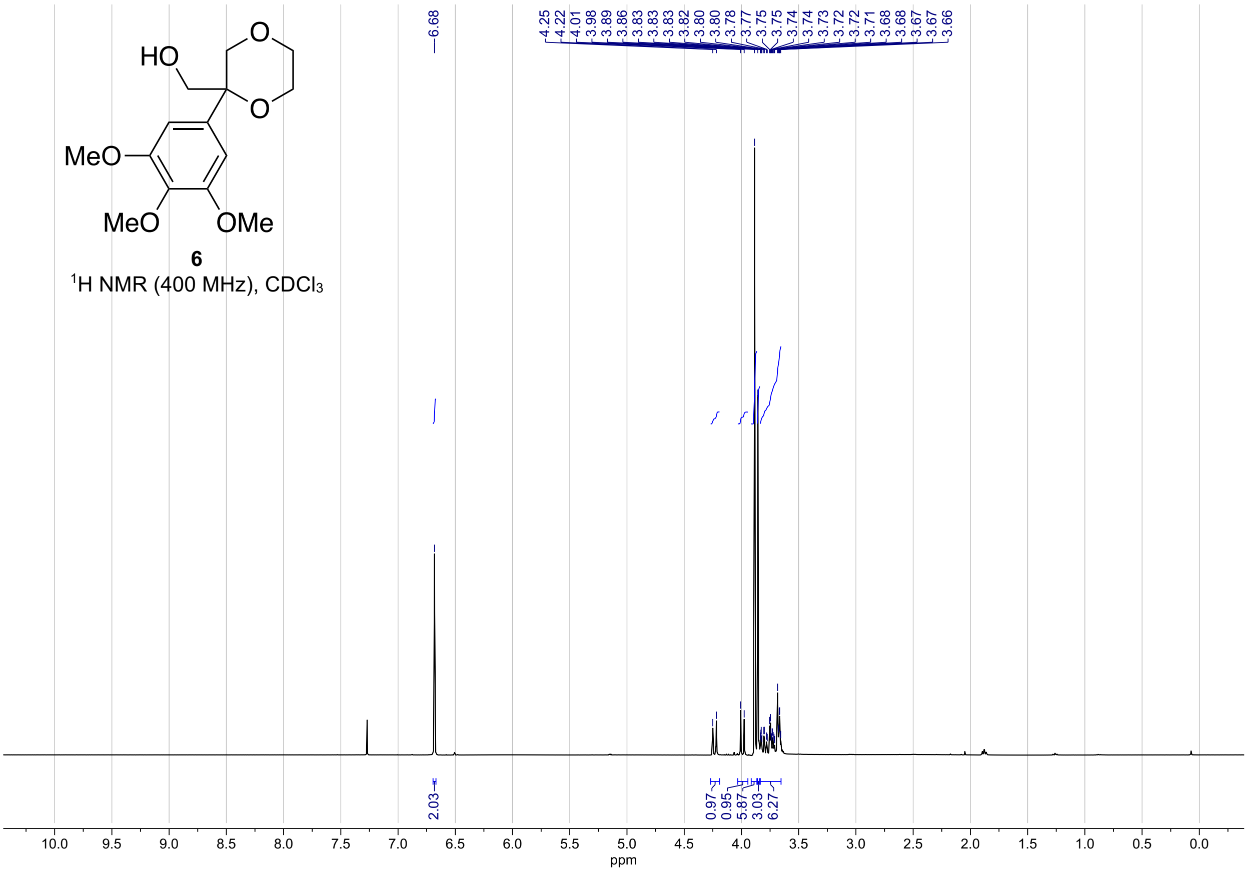
HO
O
O
MeO
MeO
OMe
6
^{1}H NMR (400 MHz), CDCl$_3$
6.68
4.25 4.22 4.01 3.98 3.89 3.86 3.83 3.83 3.83 3.82 3.80 3.80 3.78 3.77 3.75 3.75 3.74 3.74 3.73 3.72 3.72 3.71 3.68 3.68 3.67 3.67 3.66
2.03
0.97 0.95 5.87 3.03 6.27
ppm

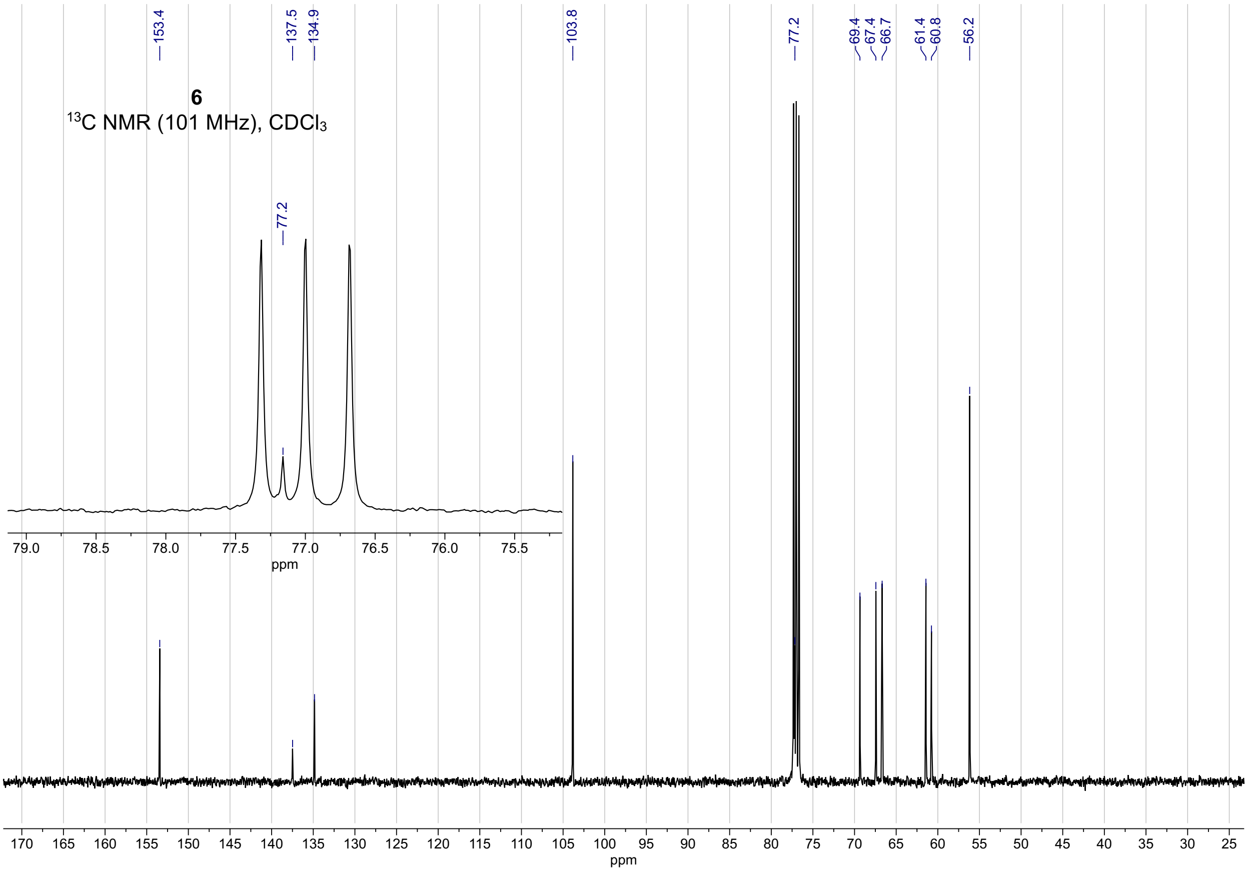
153.4
137.5
134.9
103.8
77.2
69.4 67.4 66.7
61.4 60.8
56.2
6
^{13}C NMR (101 MHz), CDCl$_3$
77.2
ppm
ppm

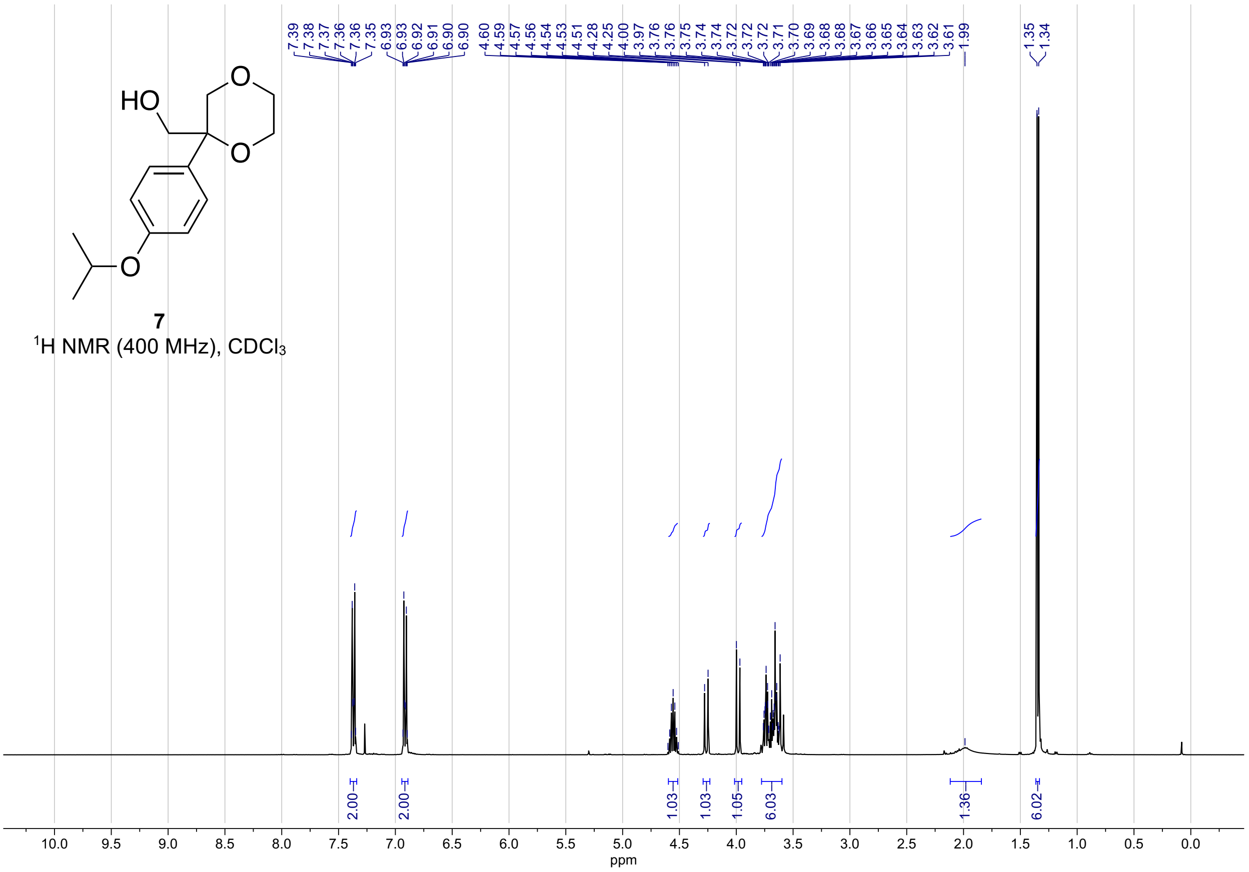

7
^{1}H NMR (400 MHz), $CDCl_3$

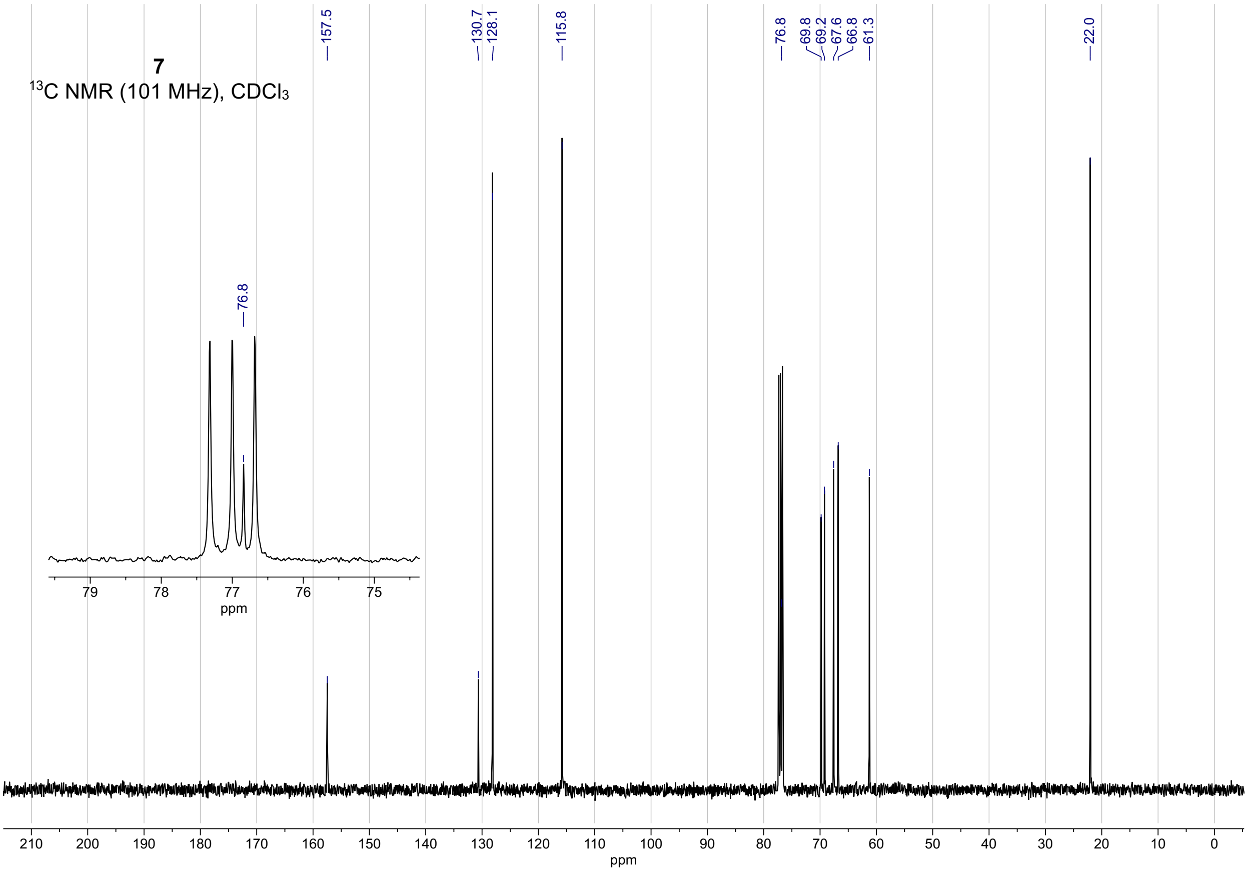

7
^{13}C NMR (101 MHz), $CDCl_3$

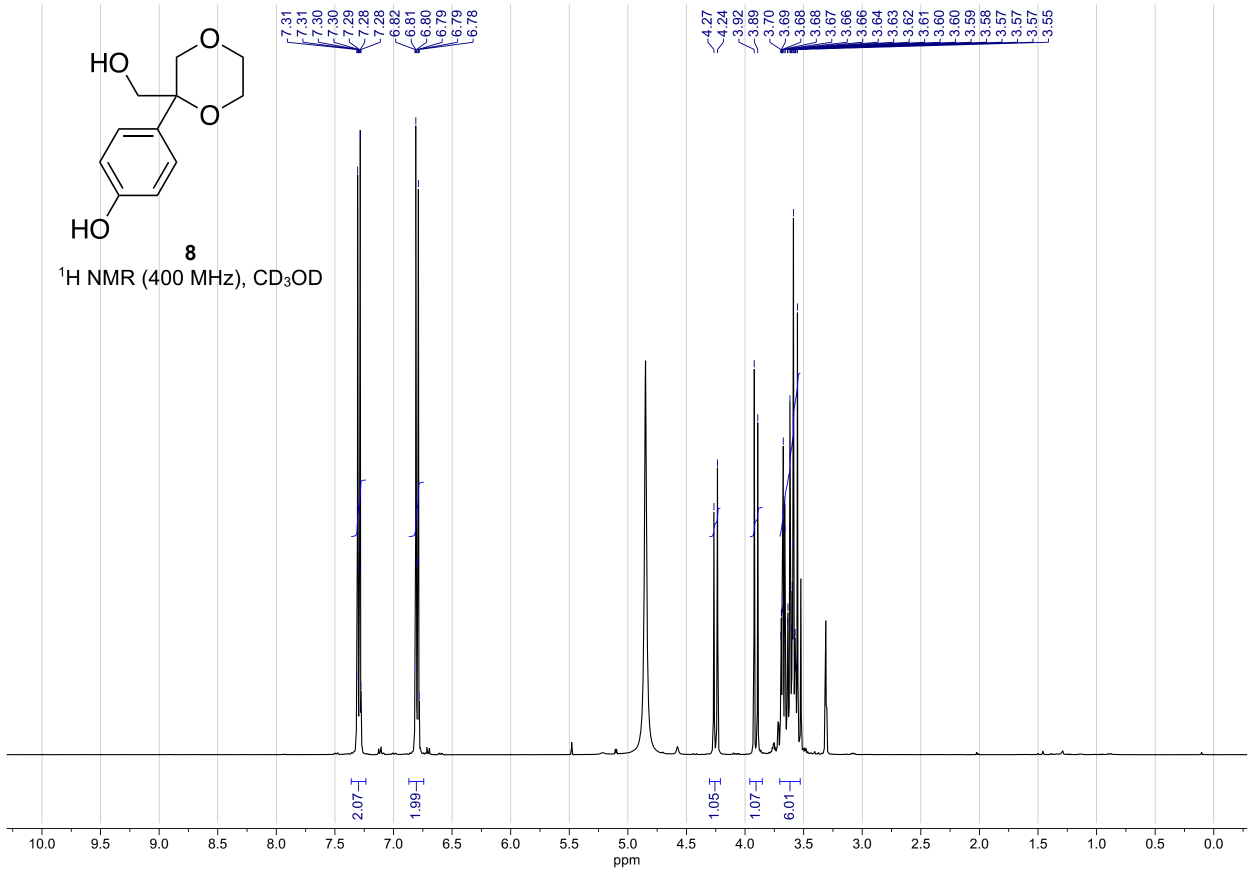
HO
O
O
HO
8
1H NMR (400 MHz), CD3OD
7.31 7.31 7.30 7.30 7.29 7.28 7.28 6.82 6.81 6.80 6.79 6.79 6.78
4.27 4.24 3.92 3.89 3.70 3.69 3.68 3.68 3.67 3.66 3.66 3.64 3.63 3.62 3.61 3.60 3.60 3.59 3.58 3.57 3.57 3.55
2.07 1.99 1.05 1.07 6.01
ppm

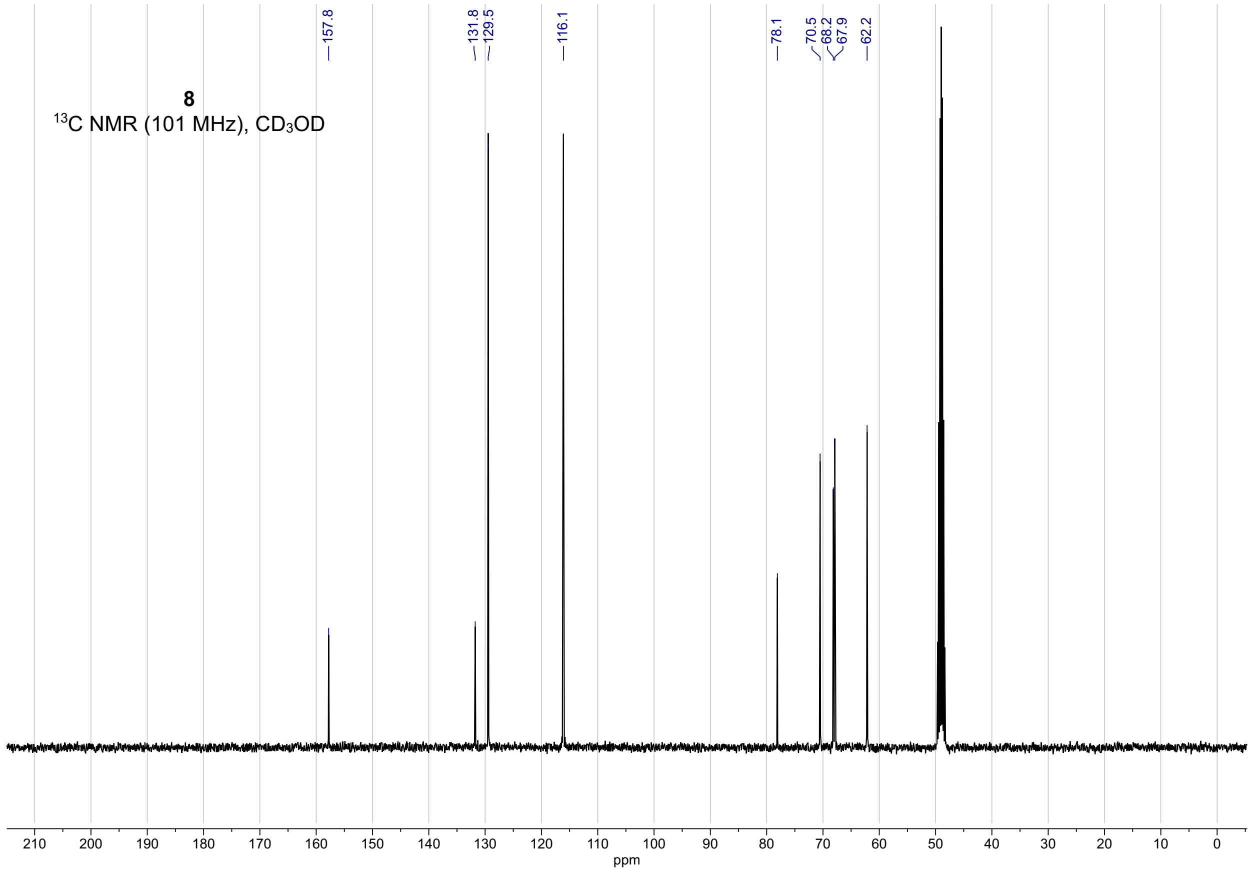
8
13C NMR (101 MHz), CD3OD
157.8 131.8 129.5 116.1 78.1 70.5 68.2 67.9 62.2
ppm

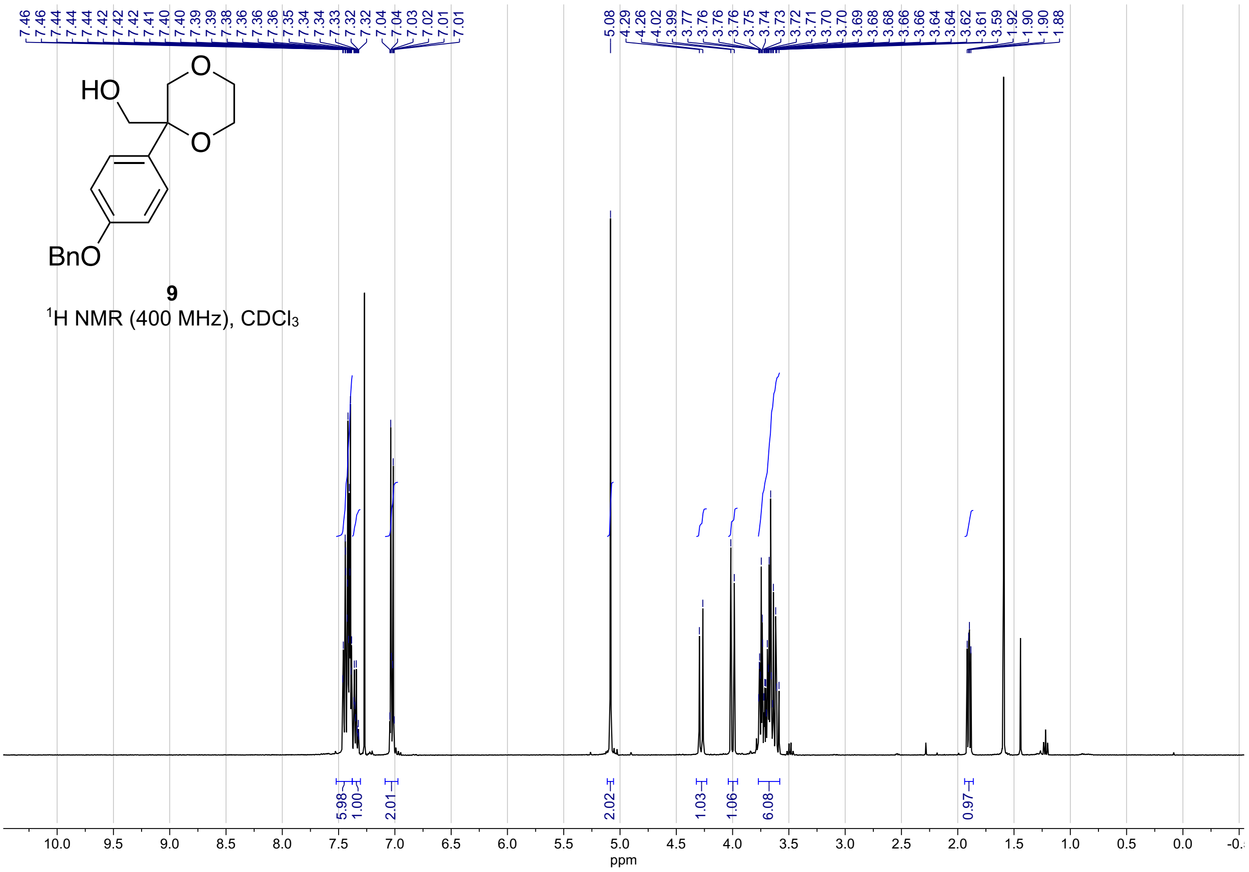

9

^{1}H NMR (400 MHz), $CDCl_3$

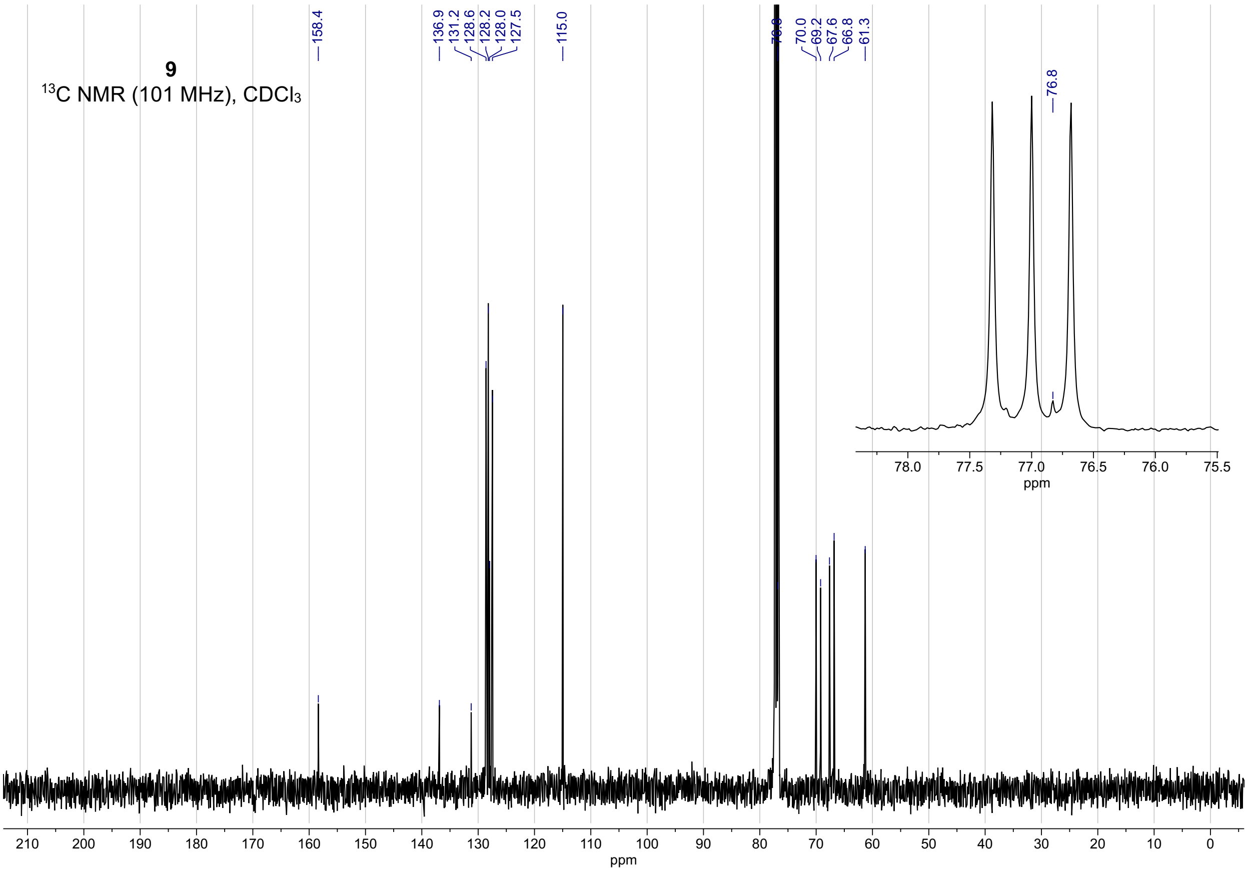

9

^{13}C NMR (101 MHz), $CDCl_3$

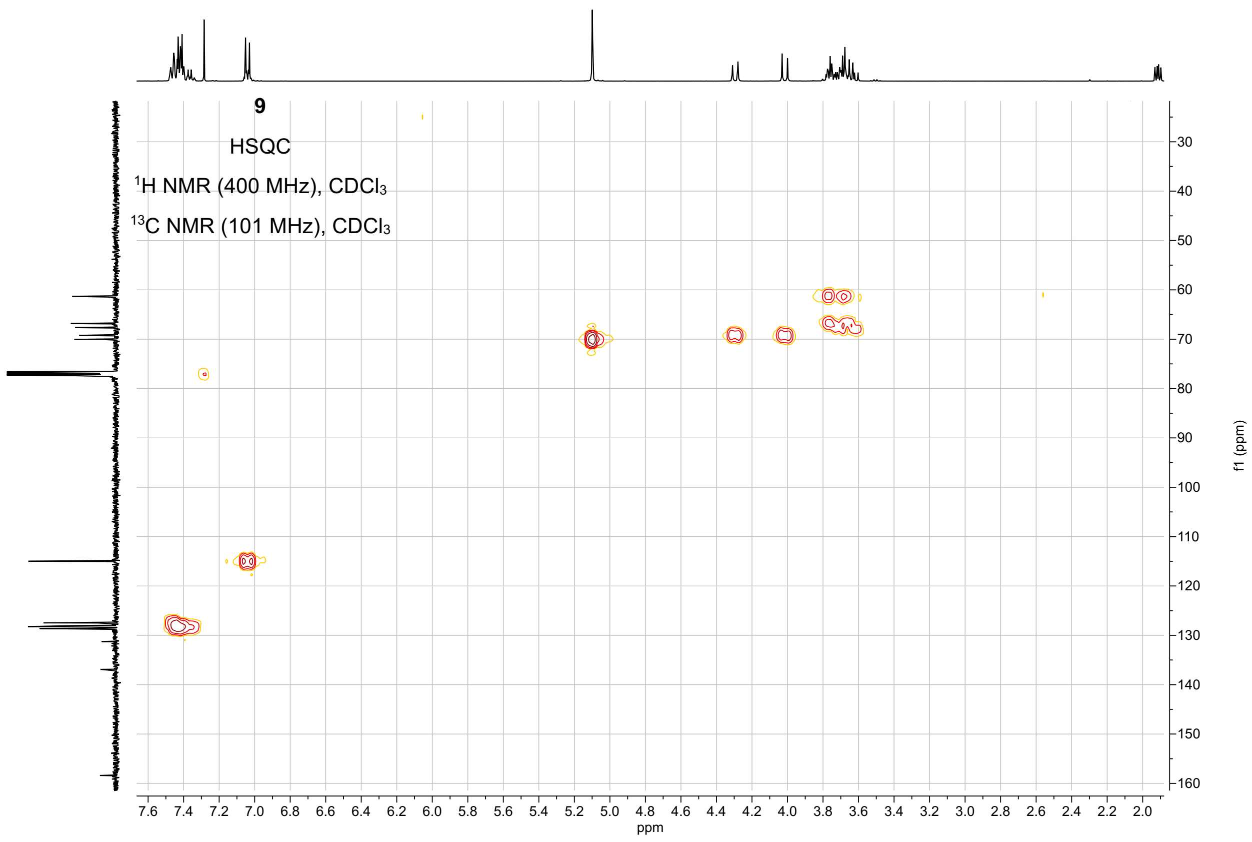

9
HSQC
1H NMR (400 MHz), CDCl3
13C NMR (101 MHz), CDCl3
ppm
f1 (ppm)

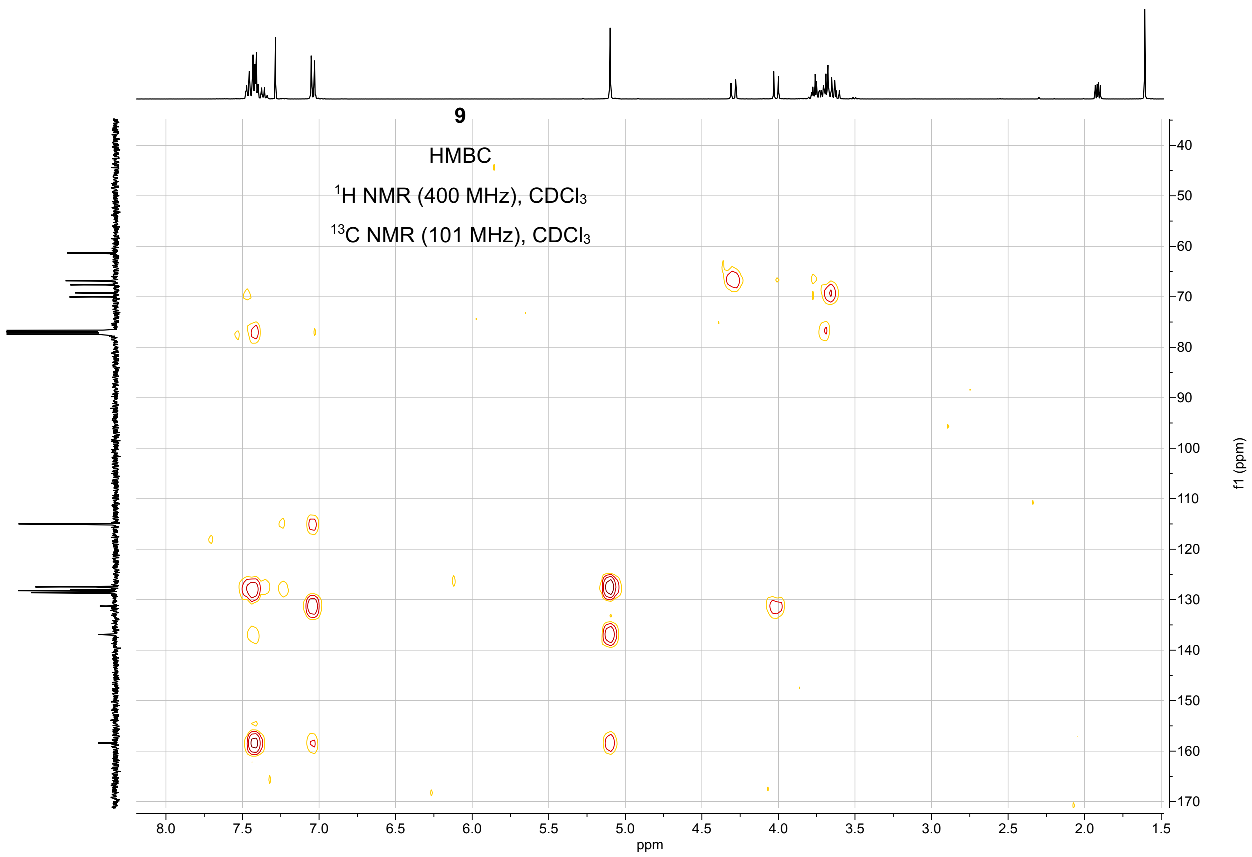

9
HMBC
1H NMR (400 MHz), CDCl3
13C NMR (101 MHz), CDCl3
ppm
f1 (ppm)

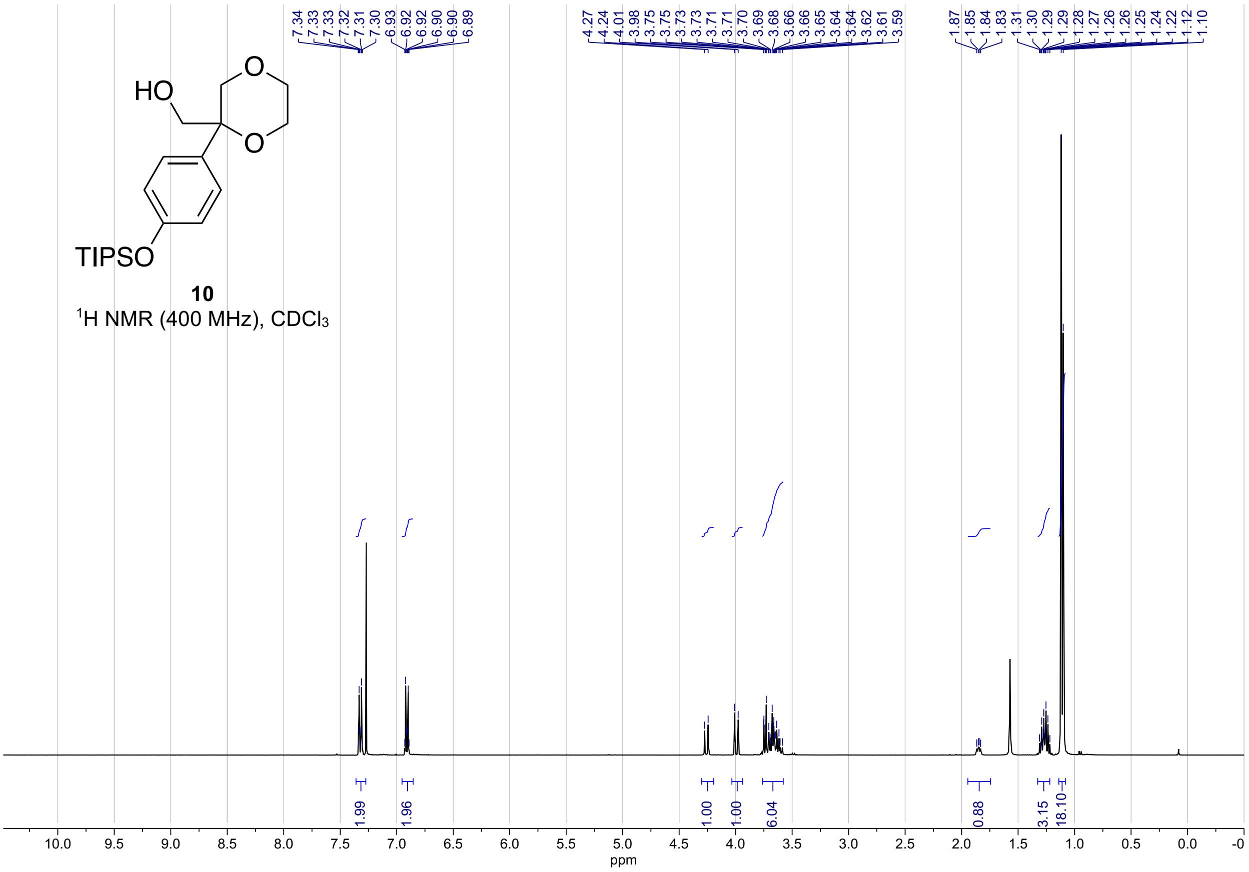

10

^{1}H NMR (400 MHz), $CDCl_3$

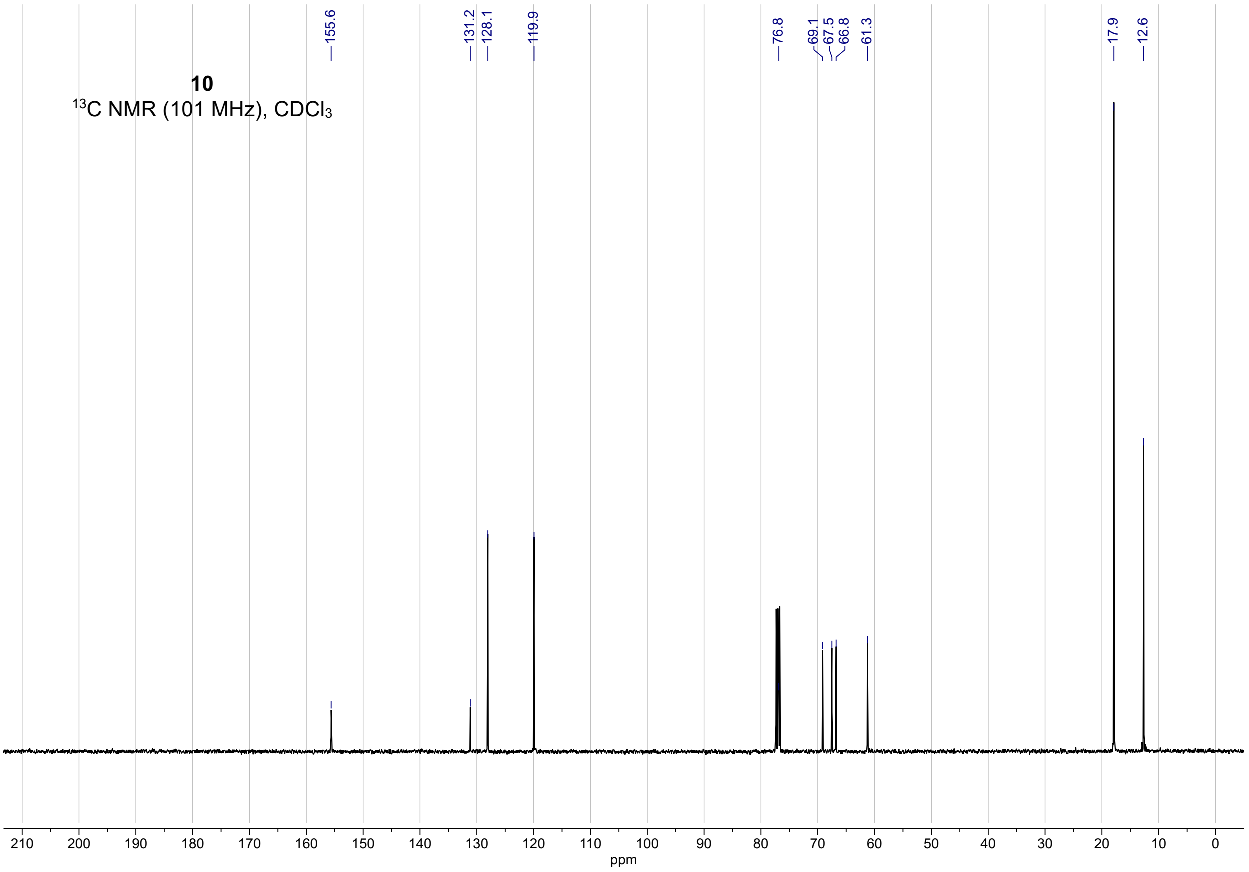

10

^{13}C NMR (101 MHz), $CDCl_3$

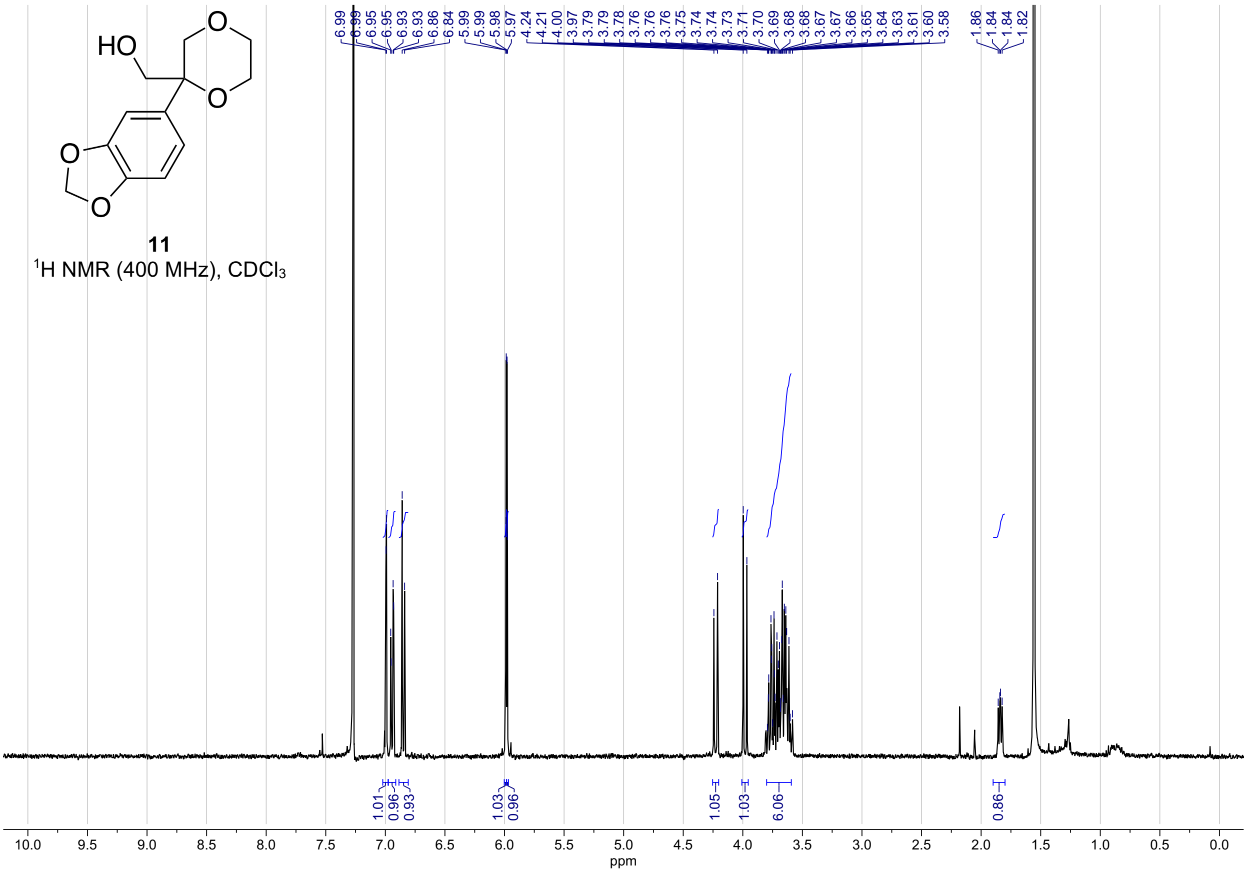
HO
O
O
O
O
11
^{1}H NMR (400 MHz), CDCl$_3$
6.99 6.99 6.95 6.93 6.93 6.86 6.84 5.99 5.99 5.98 5.97 4.24 4.21 4.00 3.97 3.79 3.79 3.78 3.76 3.76 3.75 3.74 3.74 3.73 3.71 3.70 3.69 3.68 3.68 3.67 3.67 3.66 3.65 3.64 3.63 3.61 3.60 3.58 1.86 1.84 1.84 1.82
1.01 0.96 0.93 1.03 0.96 1.05 1.03 6.06 0.86
ppm

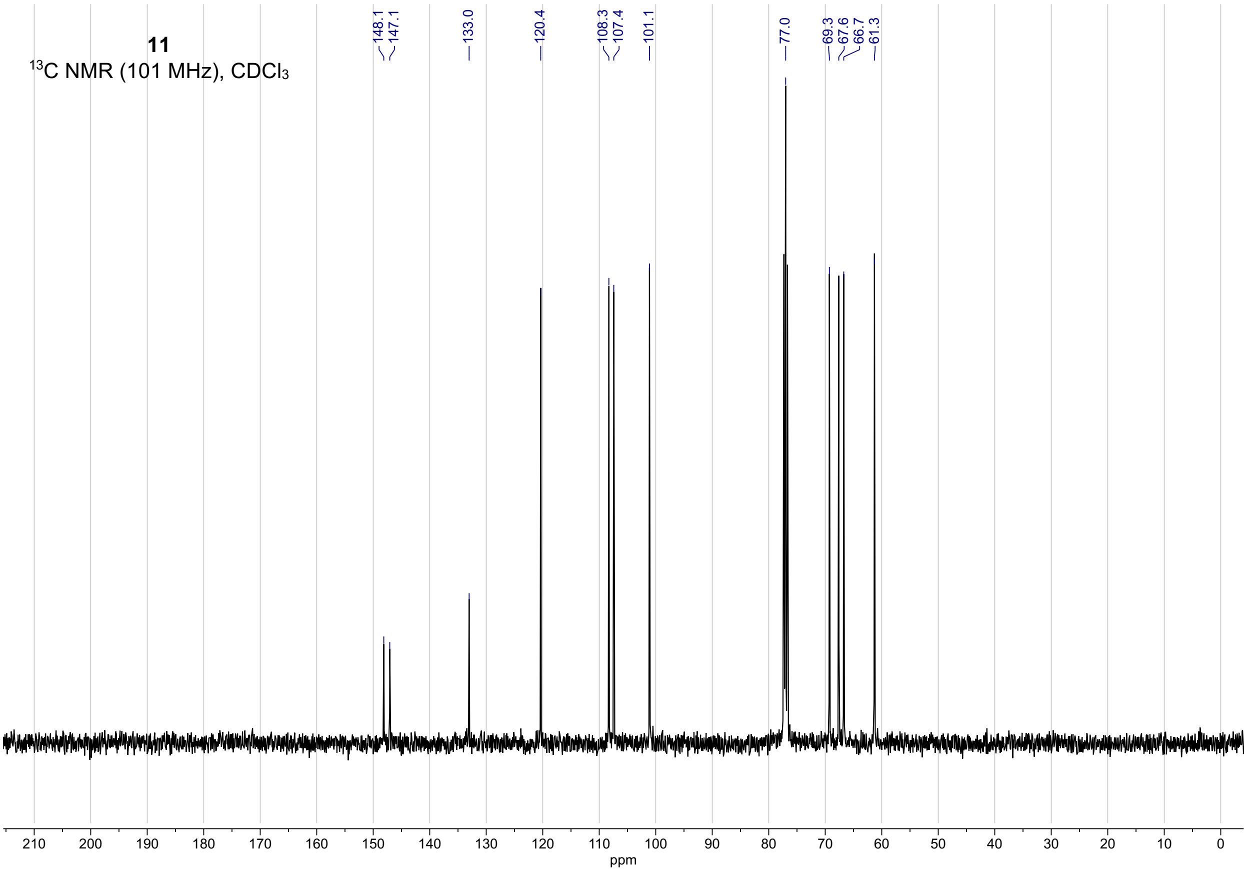
11
^{13}C NMR (101 MHz), CDCl$_3$
148.1 147.1 133.0 120.4 108.3 107.4 101.1 77.0 69.3 67.6 66.7 61.3
ppm

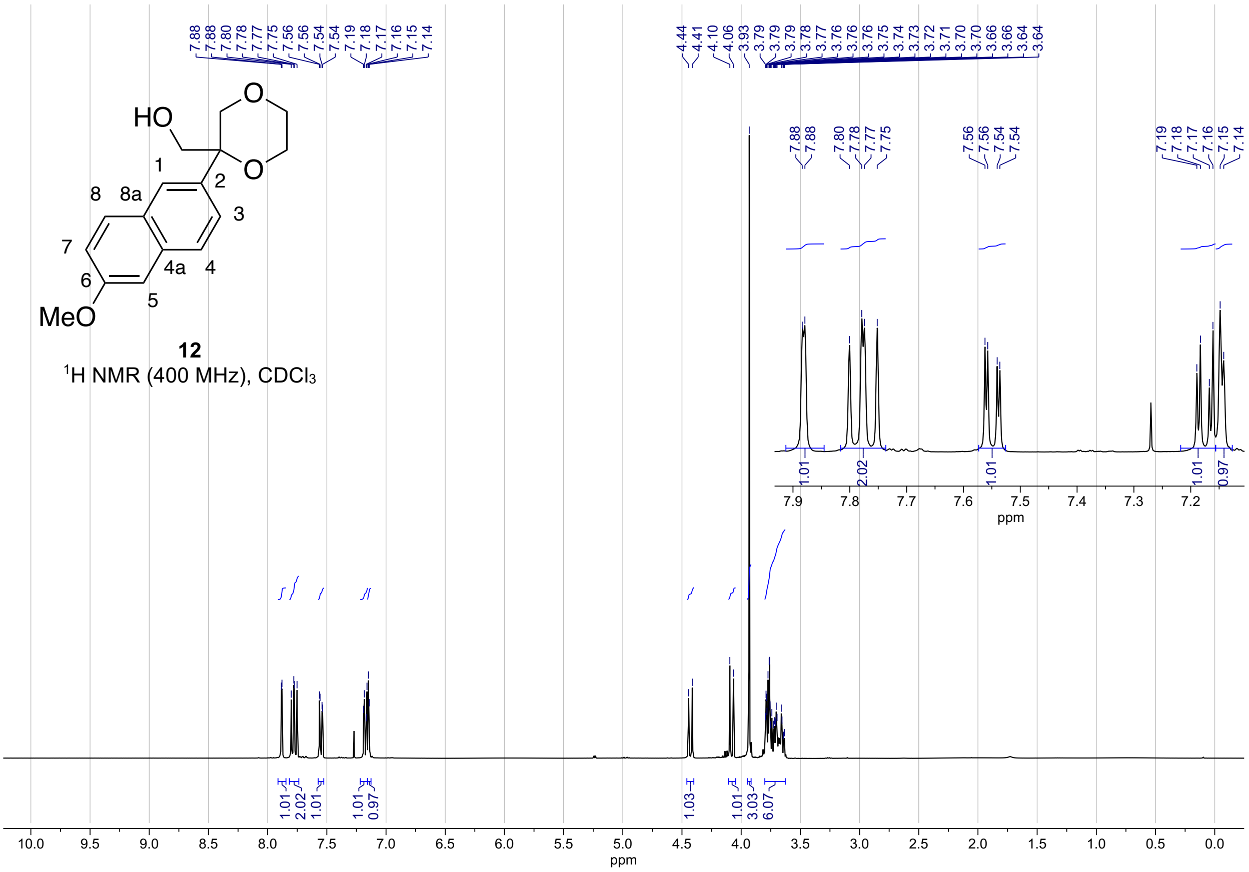

12
^{1}H NMR (400 MHz), $CDCl_3$

12
^{13}C NMR (101 MHz), $CDCl_3$

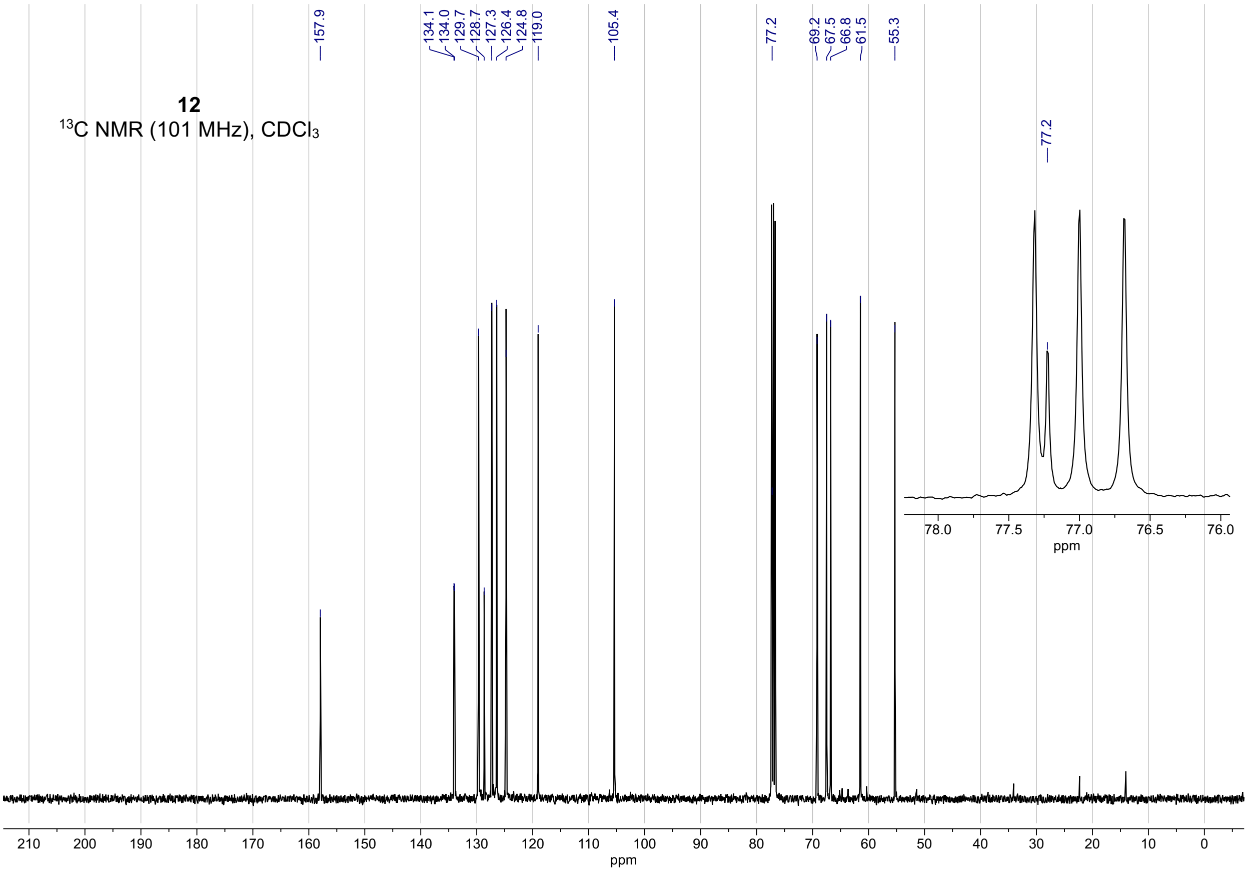

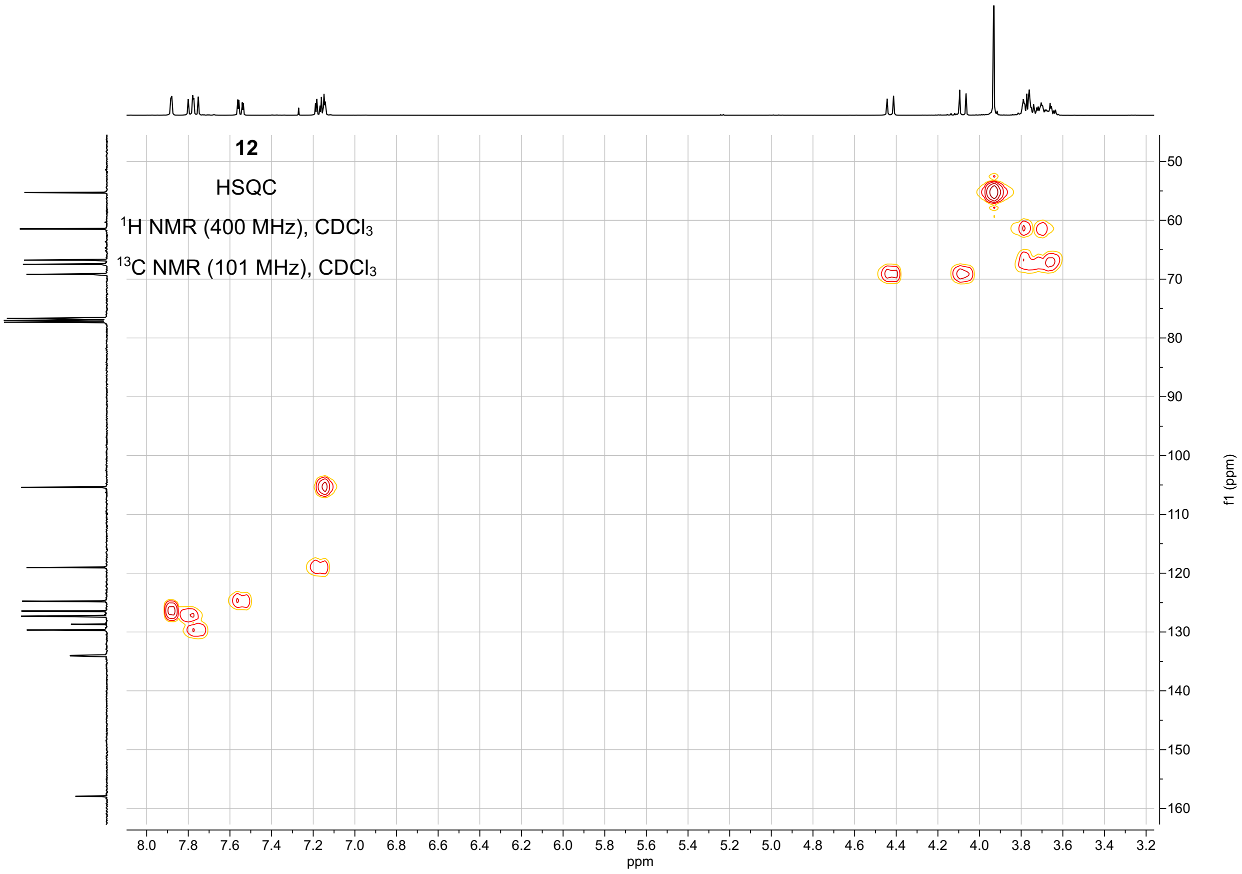
12
HSQC
^{1}H NMR (400 MHz), $CDCl_3$
^{13}C NMR (101 MHz), $CDCl_3$
ppm
f1 (ppm)

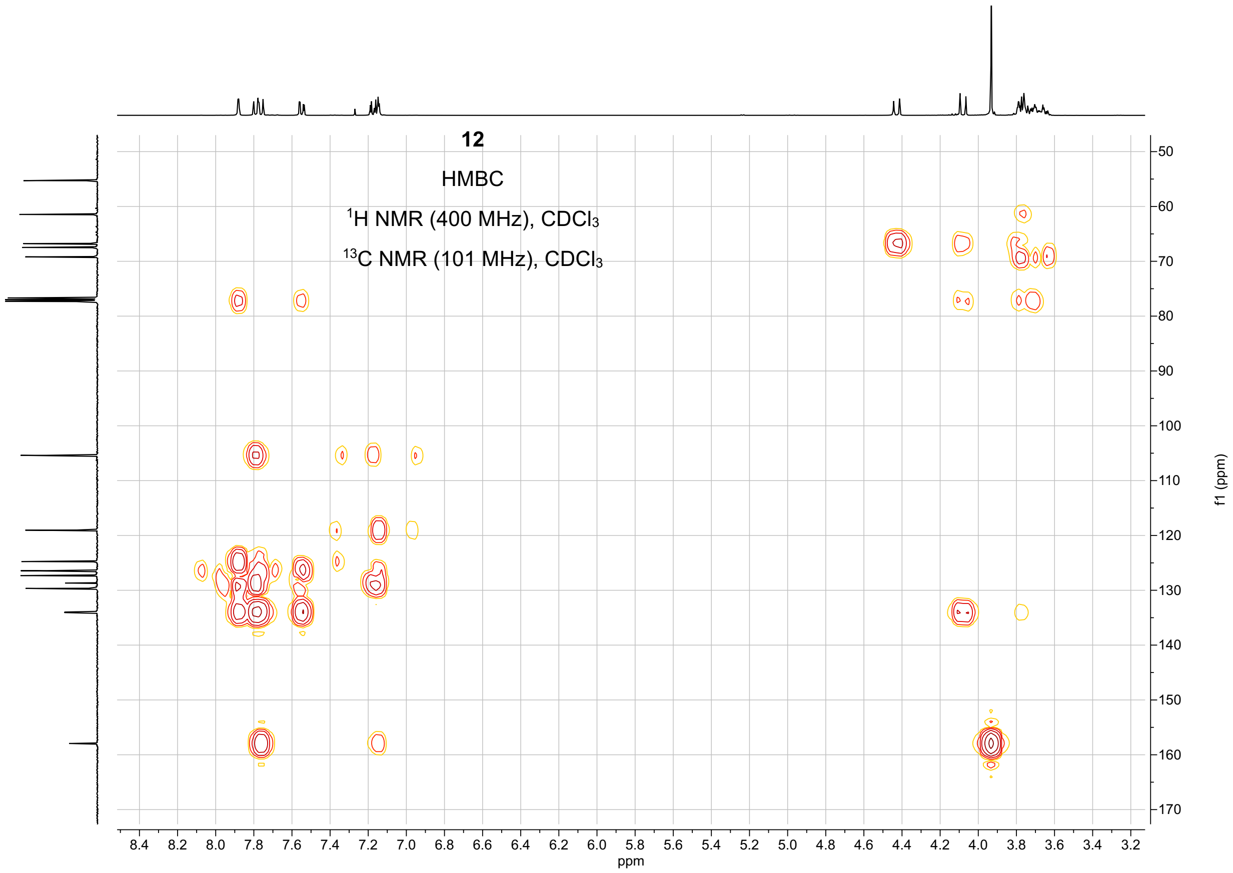
12
HMBC
^{1}H NMR (400 MHz), $CDCl_3$
^{13}C NMR (101 MHz), $CDCl_3$
ppm
f1 (ppm)

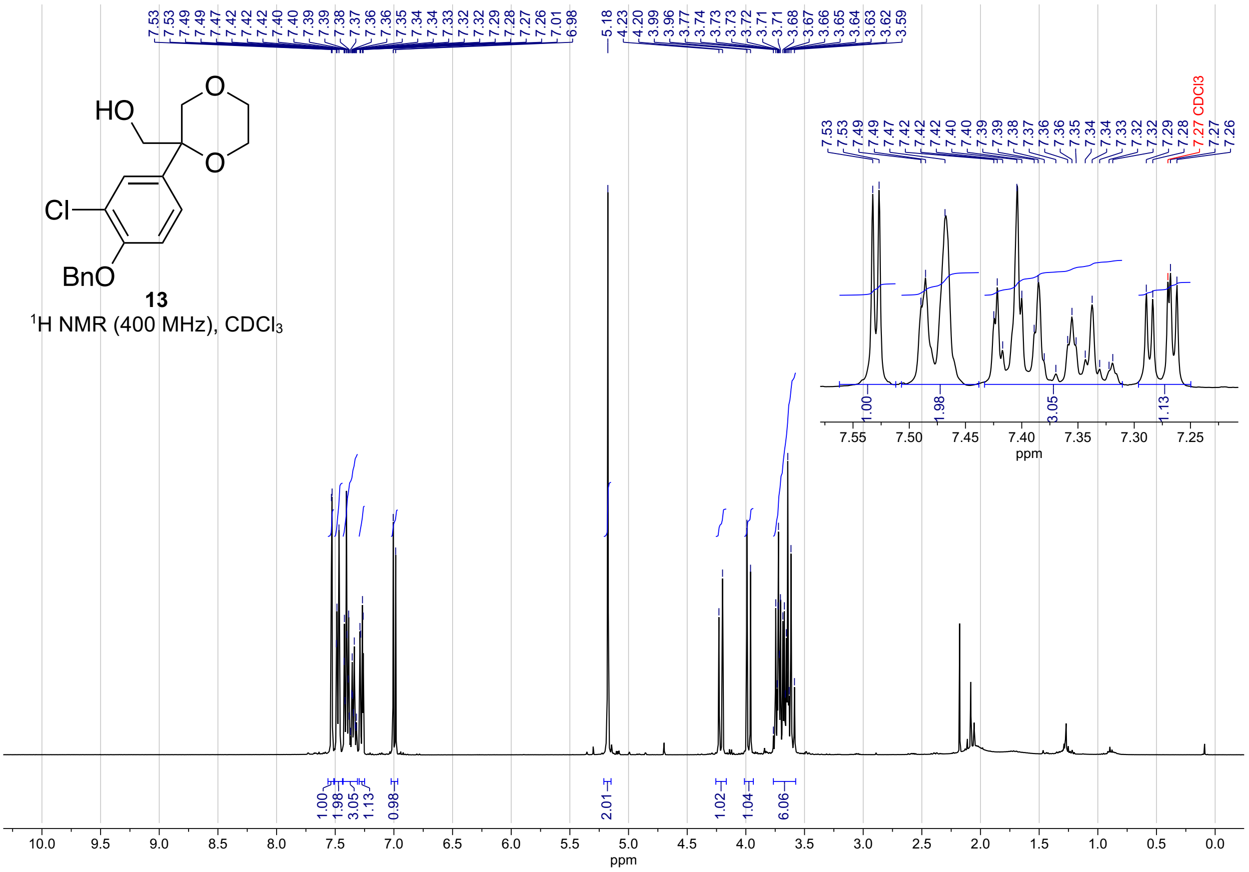

13
^{1}H NMR (400 MHz), $CDCl_3$

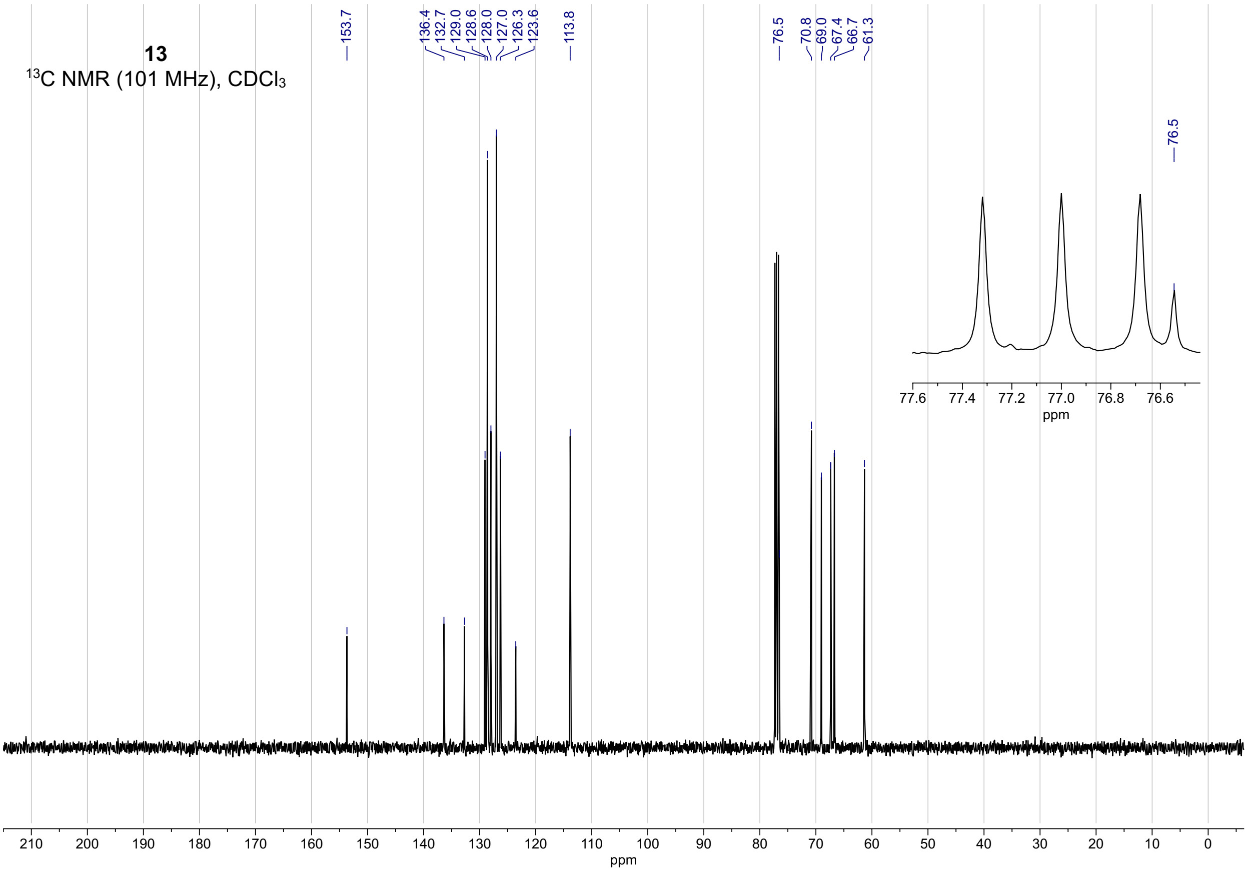

13
^{13}C NMR (101 MHz), $CDCl_3$

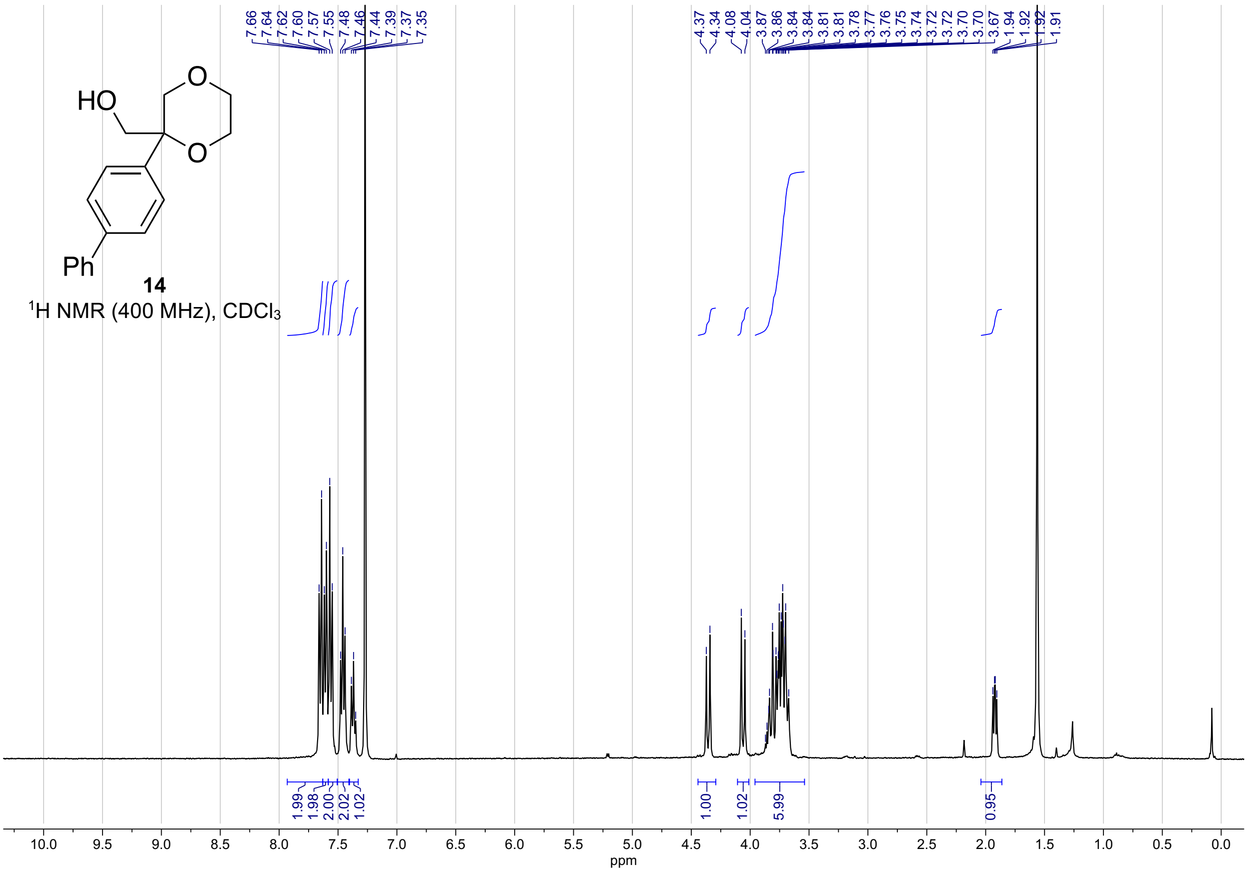
HO
O
O
Ph
14
^{1}H NMR (400 MHz), CDCl$_3$
ppm

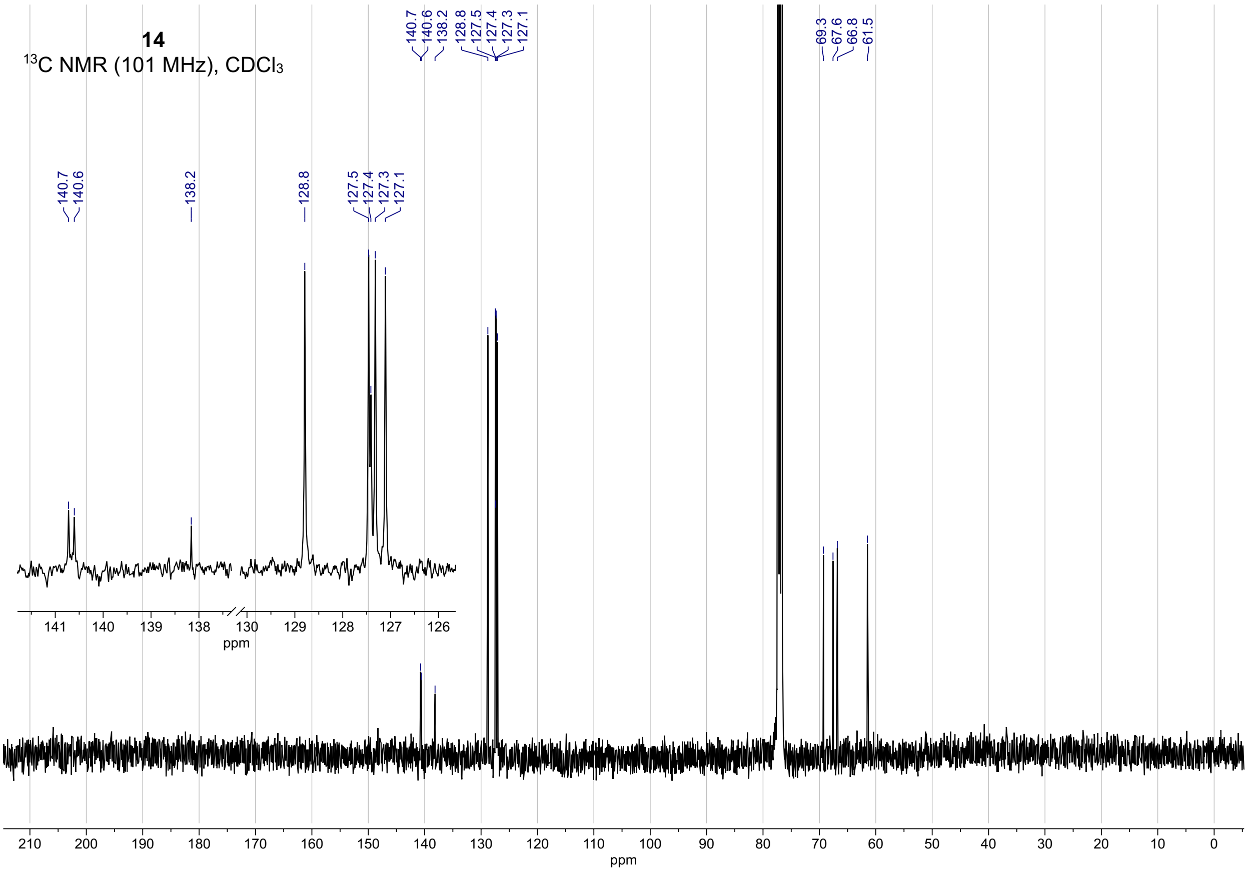
14
^{13}C NMR (101 MHz), CDCl$_3$
ppm

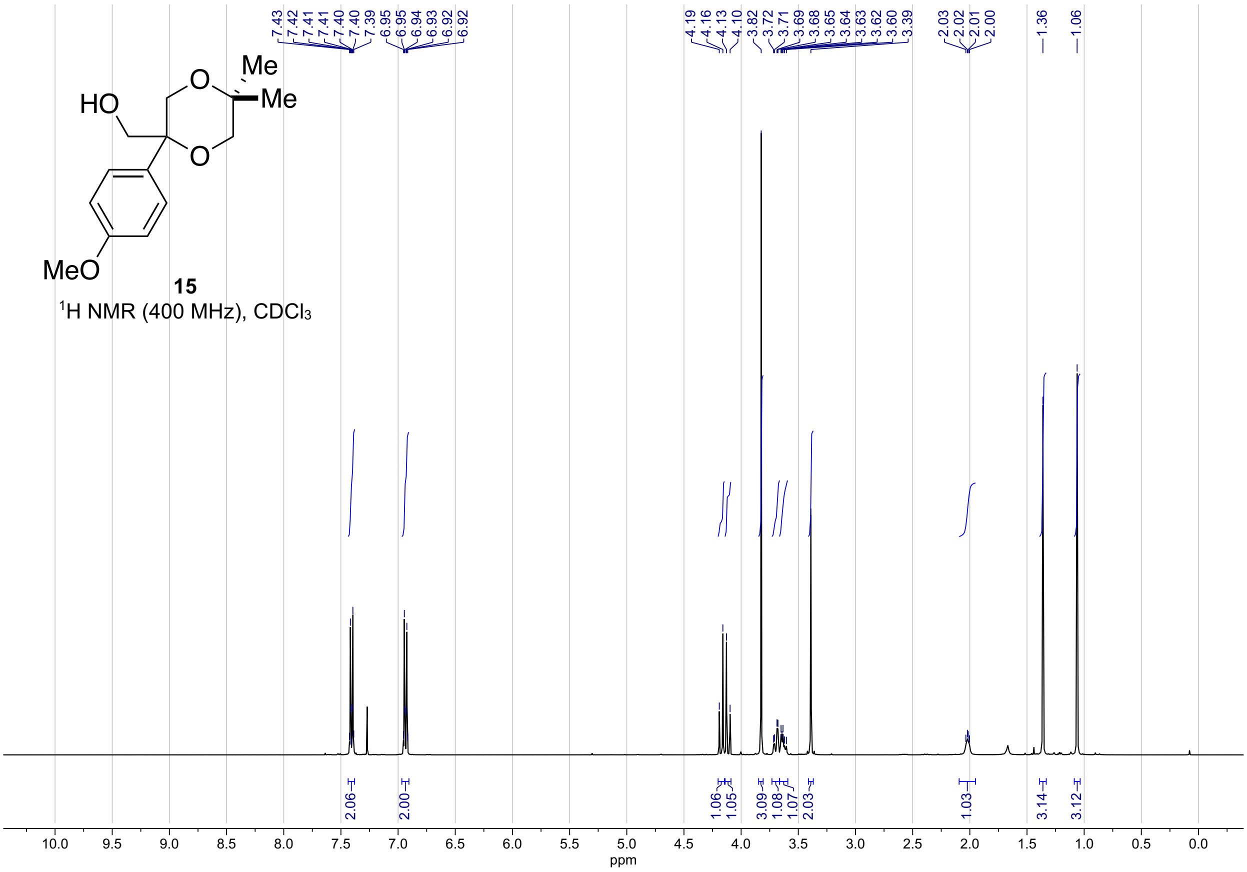

HO
O
O
Me
Me
MeO
15
^{1}H NMR (400 MHz), CDCl$_3$
7.43 7.42 7.41 7.41 7.40 7.40 7.39 6.95 6.95 6.94 6.93 6.92 6.92 6.92
4.19 4.16 4.13 4.10 3.82 3.72 3.71 3.69 3.68 3.65 3.64 3.63 3.62 3.60 3.39
2.03 2.02 2.01 2.00
1.36
1.06
2.06 2.00 1.06 1.05 3.09 1.08 1.07 2.03 1.03 3.14 3.12
ppm

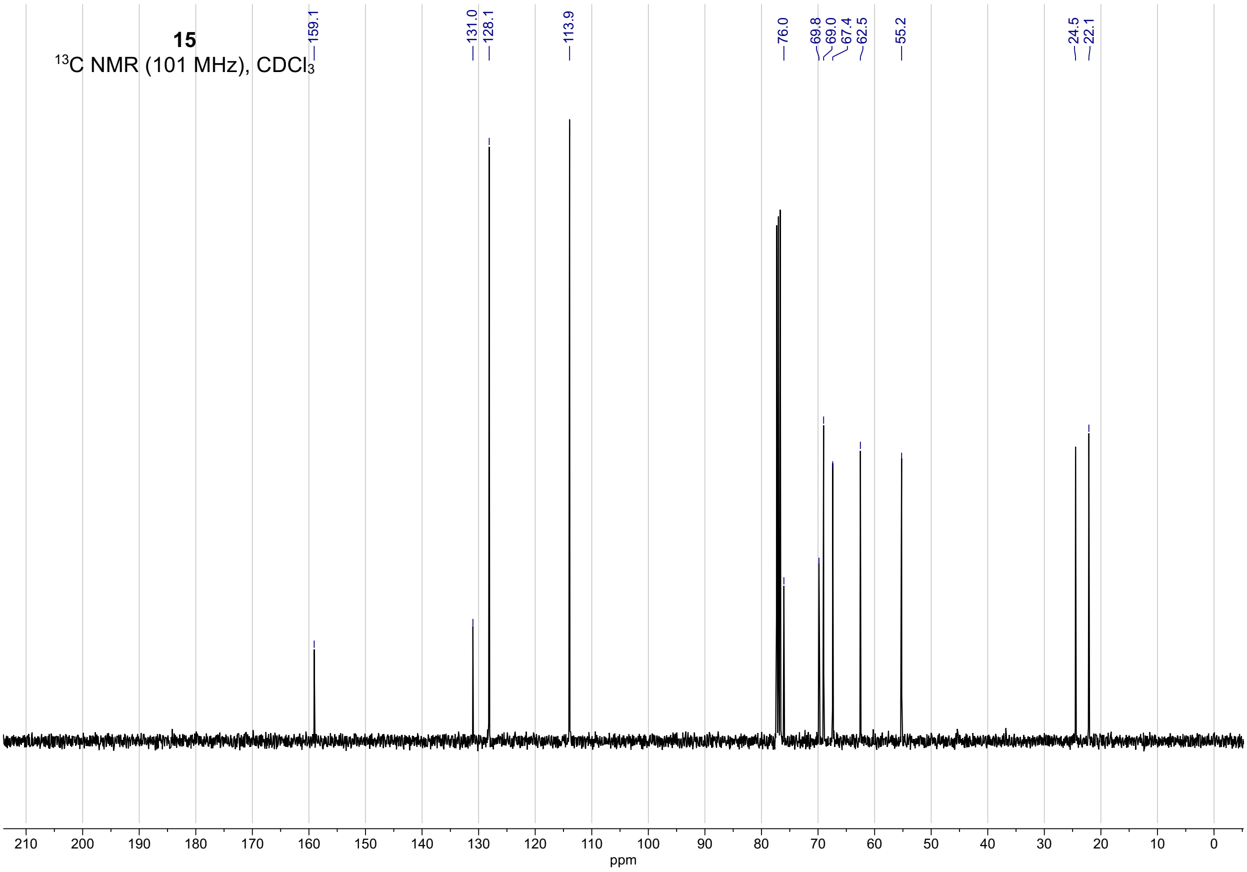

15
^{13}C NMR (101 MHz), CDCl$_3$
159.1
131.0 128.1
113.9
76.0
69.8 69.0 67.4 62.5
55.2
24.5 22.1
ppm

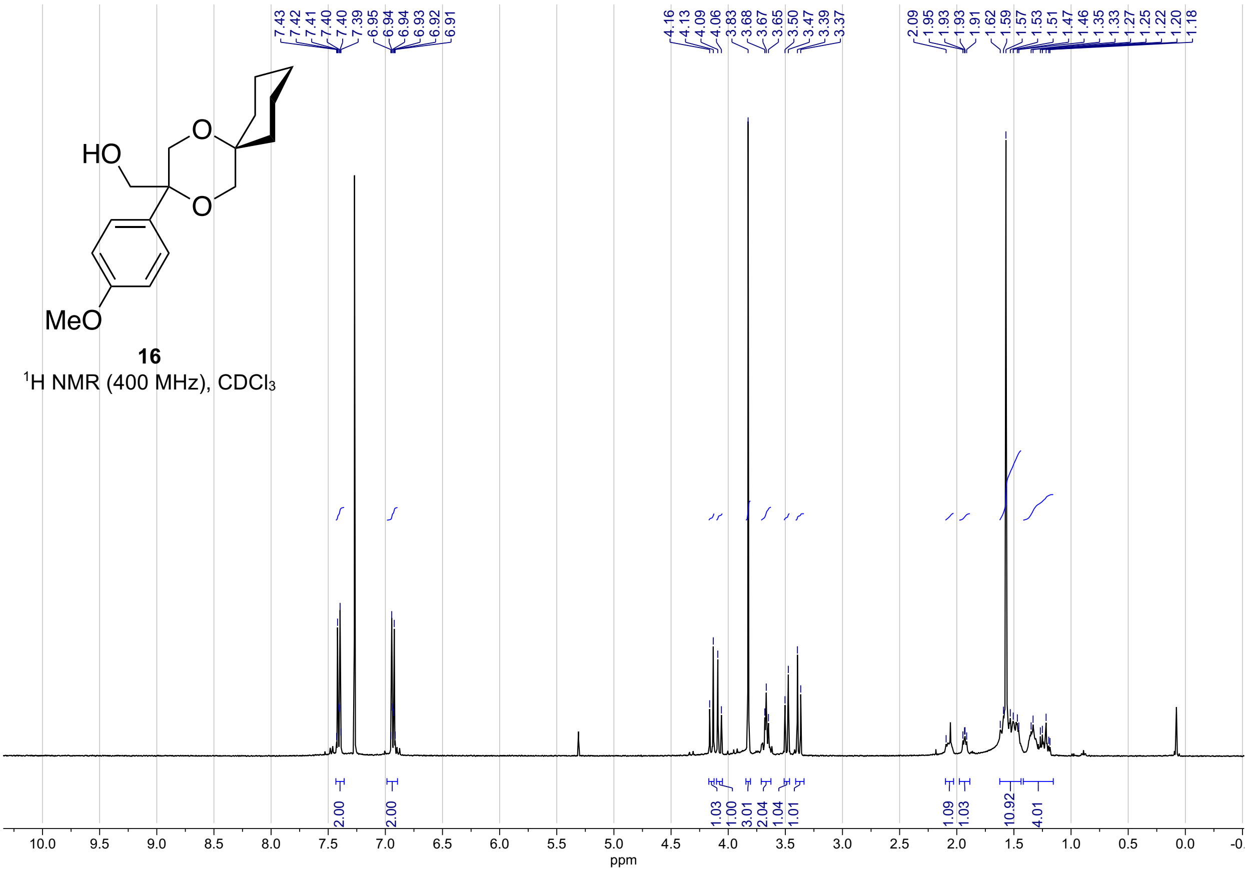

16
^{1}H NMR (400 MHz), $CDCl_3$

16
^{13}C NMR (101 MHz), $CDCl_3$

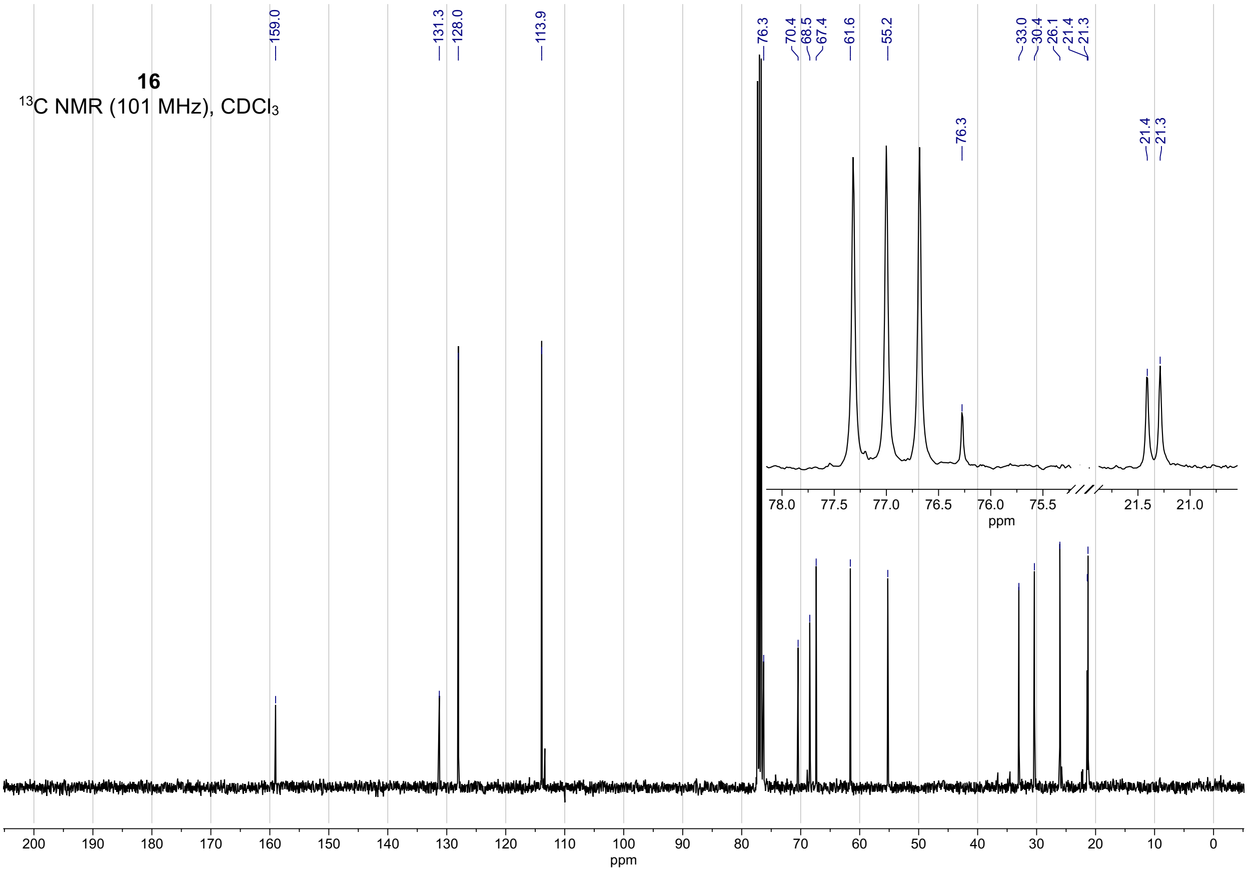

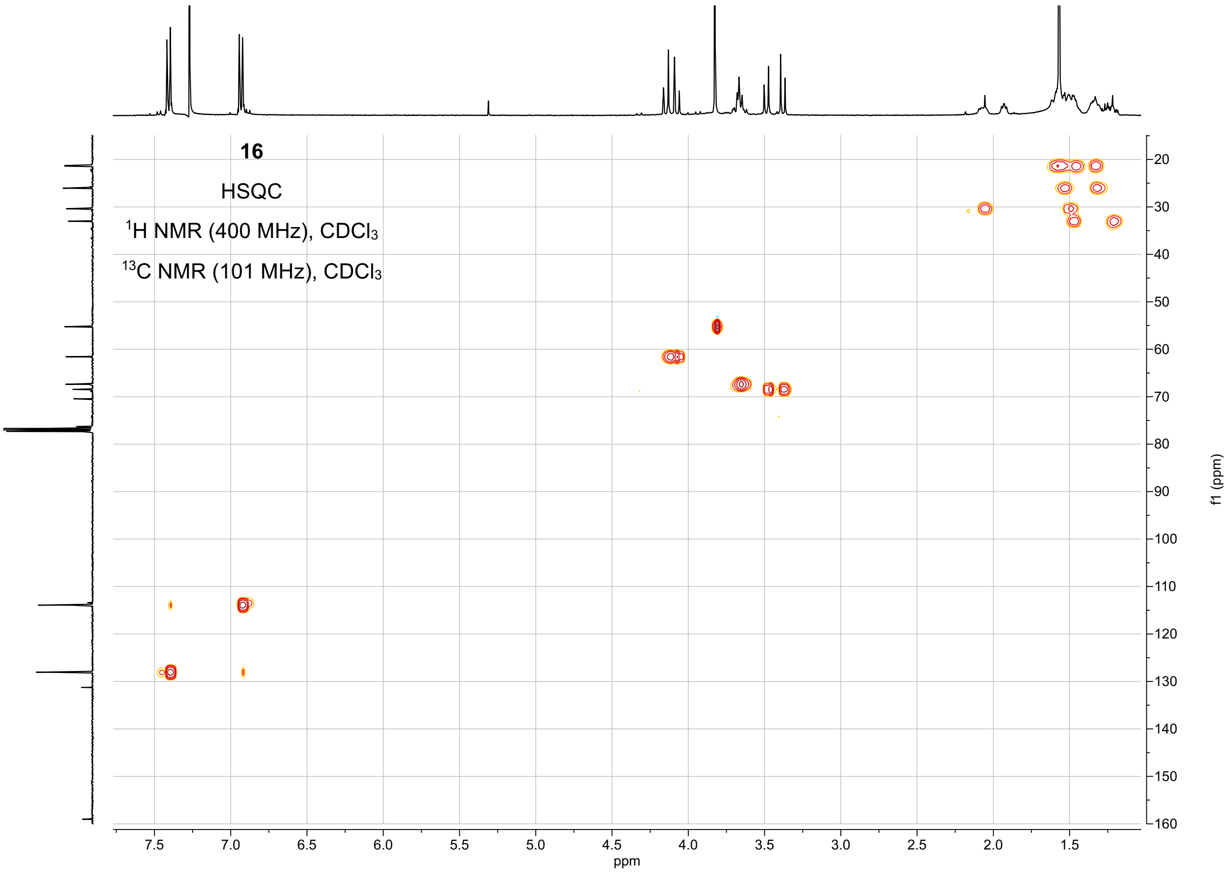
16
HSQC
1H NMR (400 MHz), CDCl3
13C NMR (101 MHz), CDCl3
f1 (ppm)
ppm

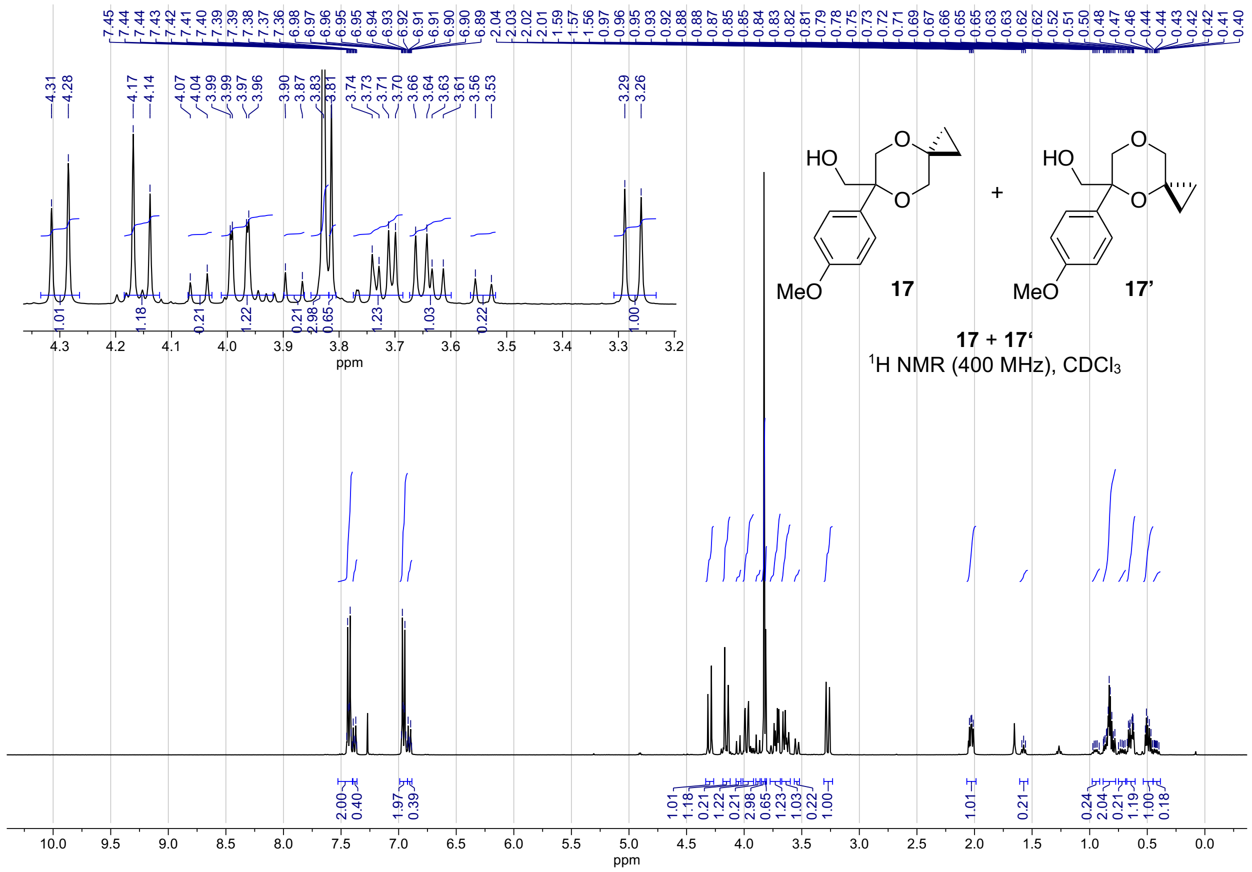

17 + 17'
^{1}H NMR (400 MHz), $CDCl_3$

17 + 17'
^{13}C NMR (101 MHz), $CDCl_3$

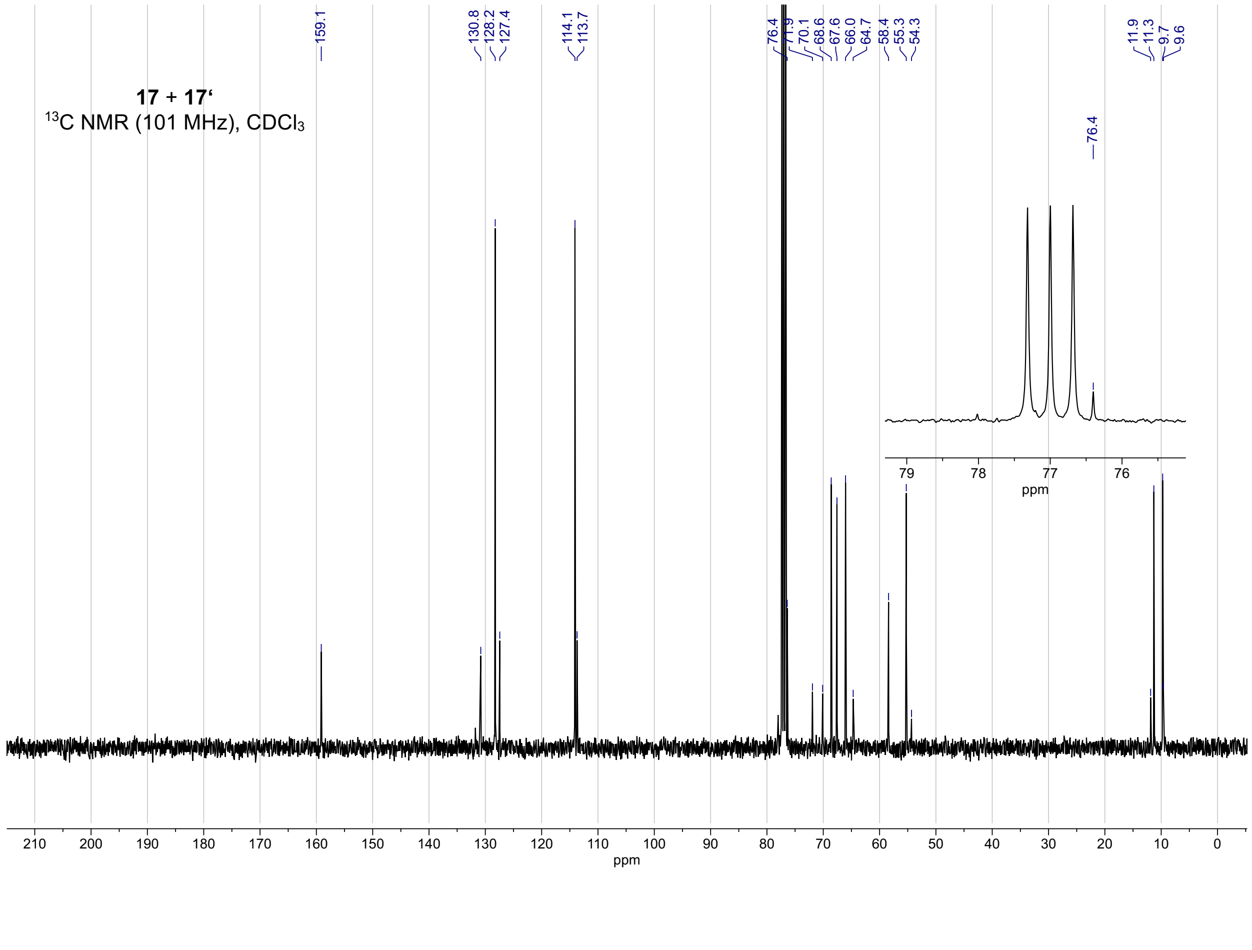

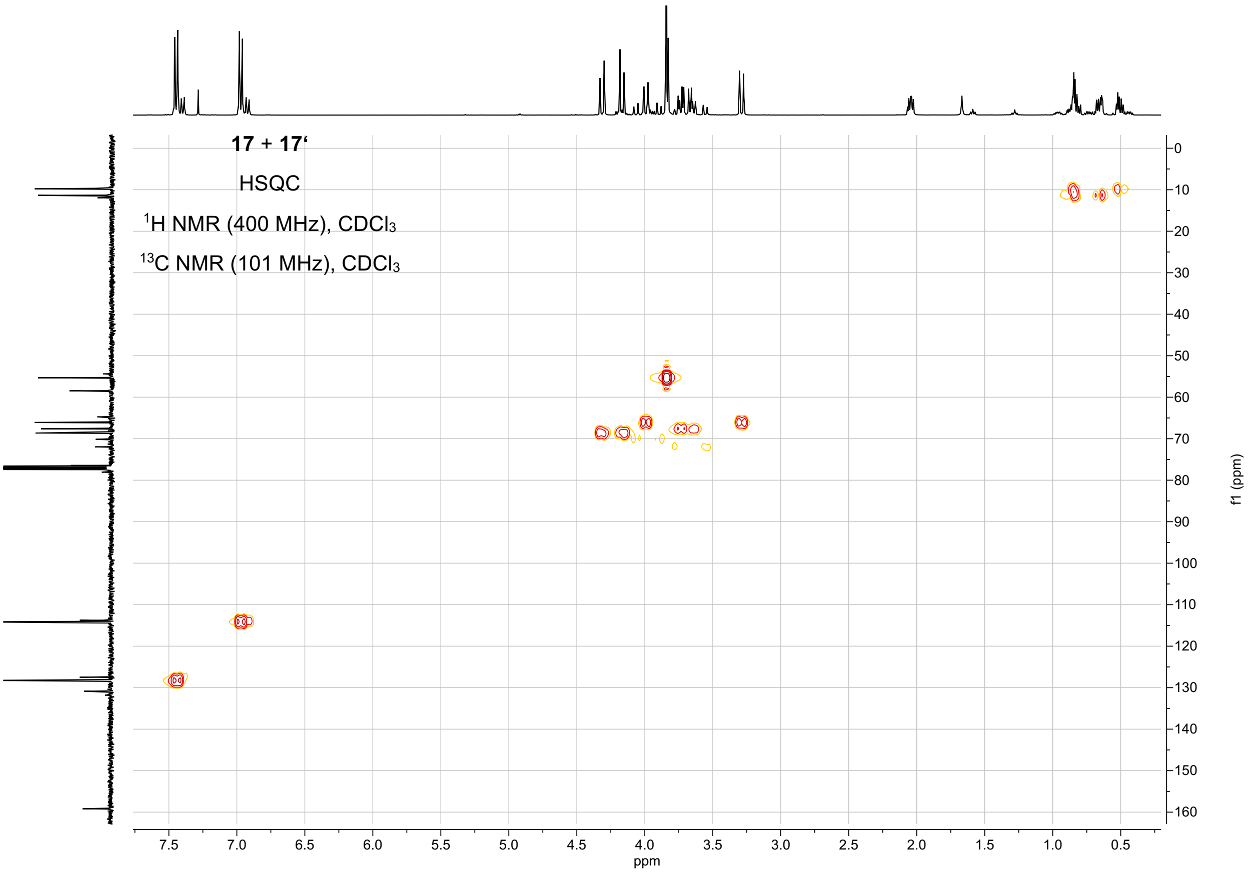
17 + 17'
HSQC
¹H NMR (400 MHz), CDCl3
¹³C NMR (101 MHz), CDCl3
ppm
f1 (ppm)
7.5
7.0
6.5
6.0
5.5
5.0
4.5
4.0
3.5
3.0
2.5
2.0
1.5
1.0
0.5
0
10
20
30
40
50
60
70
80
90
100
110
120
130
140
150
160

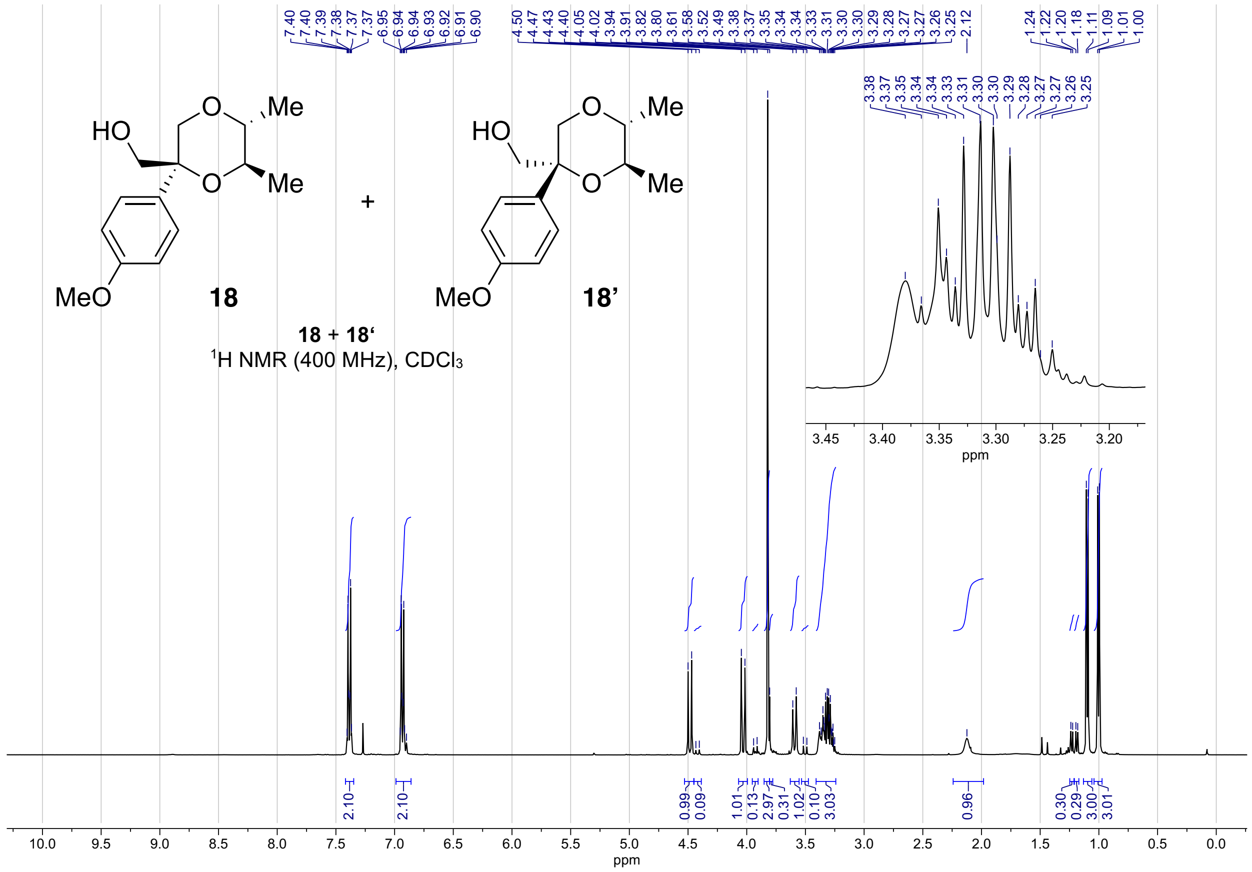
7.40 7.40 7.39 7.38 7.37 7.37 6.95 6.94 6.94 6.93 6.92 6.91 6.90
4.50 4.47 4.43 4.40 4.05 4.02 3.94 3.91 3.82 3.80 3.61 3.58 3.52 3.49 3.38 3.37 3.35 3.34 3.34 3.33 3.31 3.30 3.30 3.29 3.28 3.27 3.27 3.26 3.25 2.12
1.24 1.22 1.20 1.18 1.11 1.09 1.01 1.00
HO
O
Me
Me
MeO
18
+
18'
18 + 18'
¹H NMR (400 MHz), CDCl₃
3.38 3.37 3.35 3.34 3.34 3.33 3.31 3.30 3.30 3.29 3.28 3.27 3.27 3.26 3.25
2.10 2.10 0.99 0.09 1.01 0.13 2.97 0.31 1.02 0.10 3.03 0.96 0.30 0.29 3.00 3.01
ppm

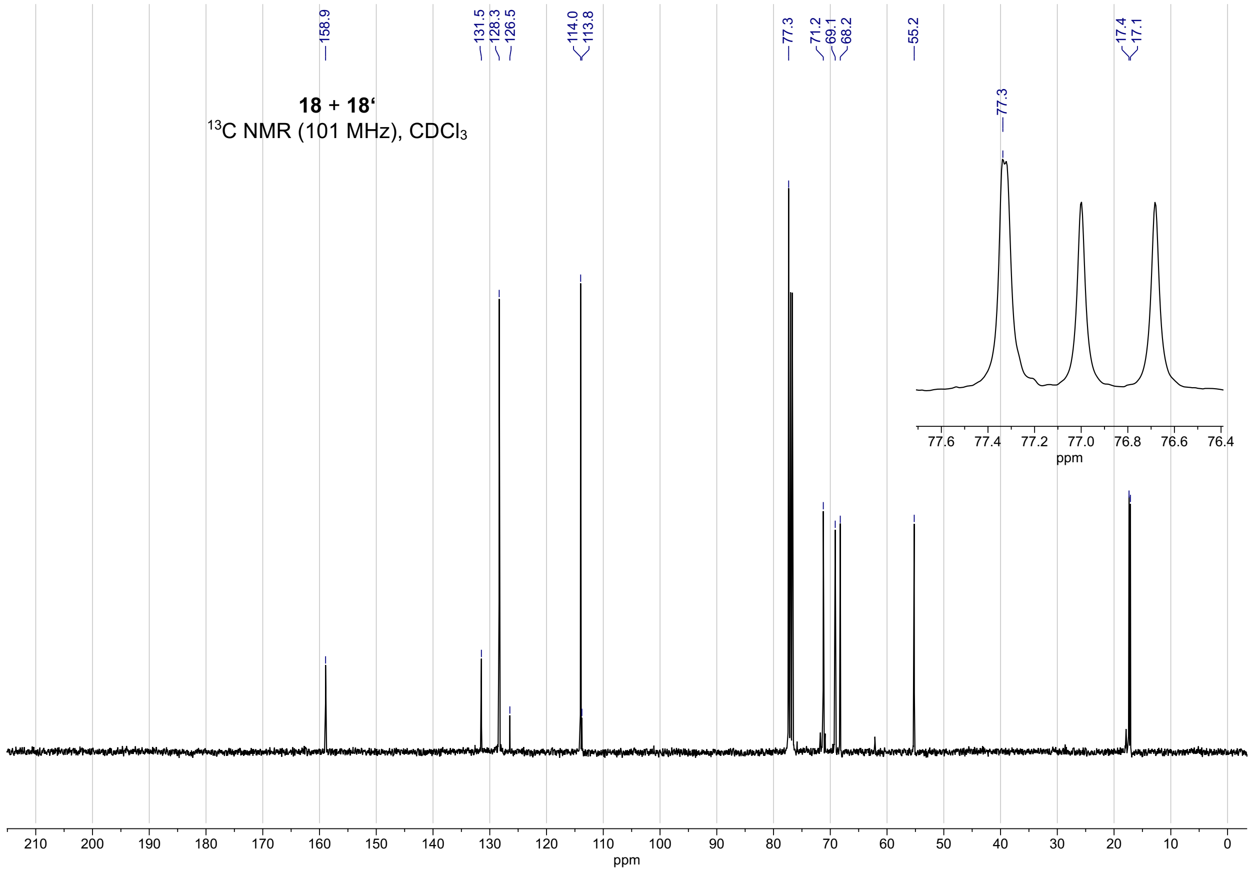
158.9
131.5 128.3 126.5
114.0 113.8
77.3
71.2 69.1 68.2
55.2
17.4 17.1
18 + 18'
¹³C NMR (101 MHz), CDCl₃
77.3
ppm

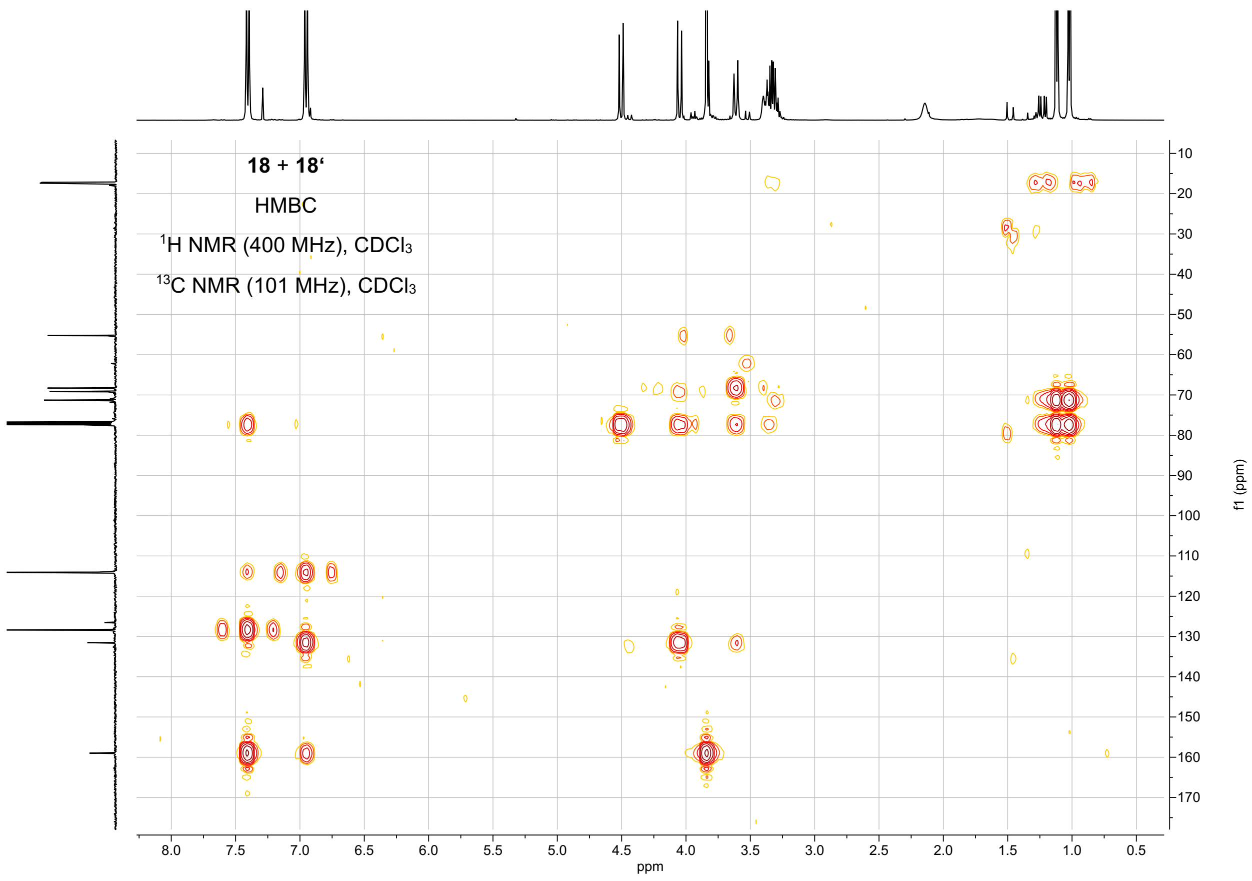
18 + 18'
HMBC
^{1}H NMR (400 MHz), CDCl3
^{13}C NMR (101 MHz), CDCl3
ppm
f1 (ppm)

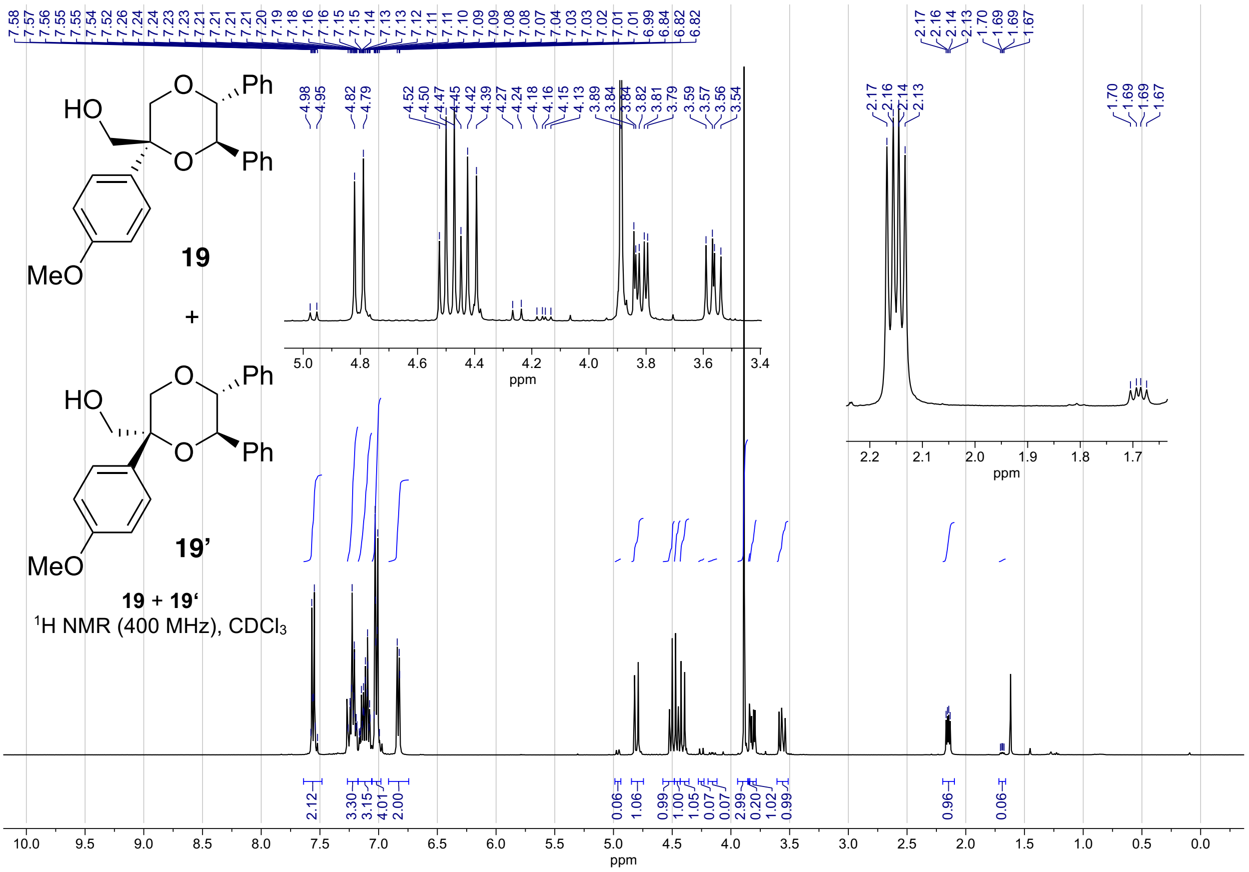
HO
Ph
Ph
MeO
19
+
HO
Ph
Ph
MeO
19'
19 + 19'
1H NMR (400 MHz), CDCl3
ppm

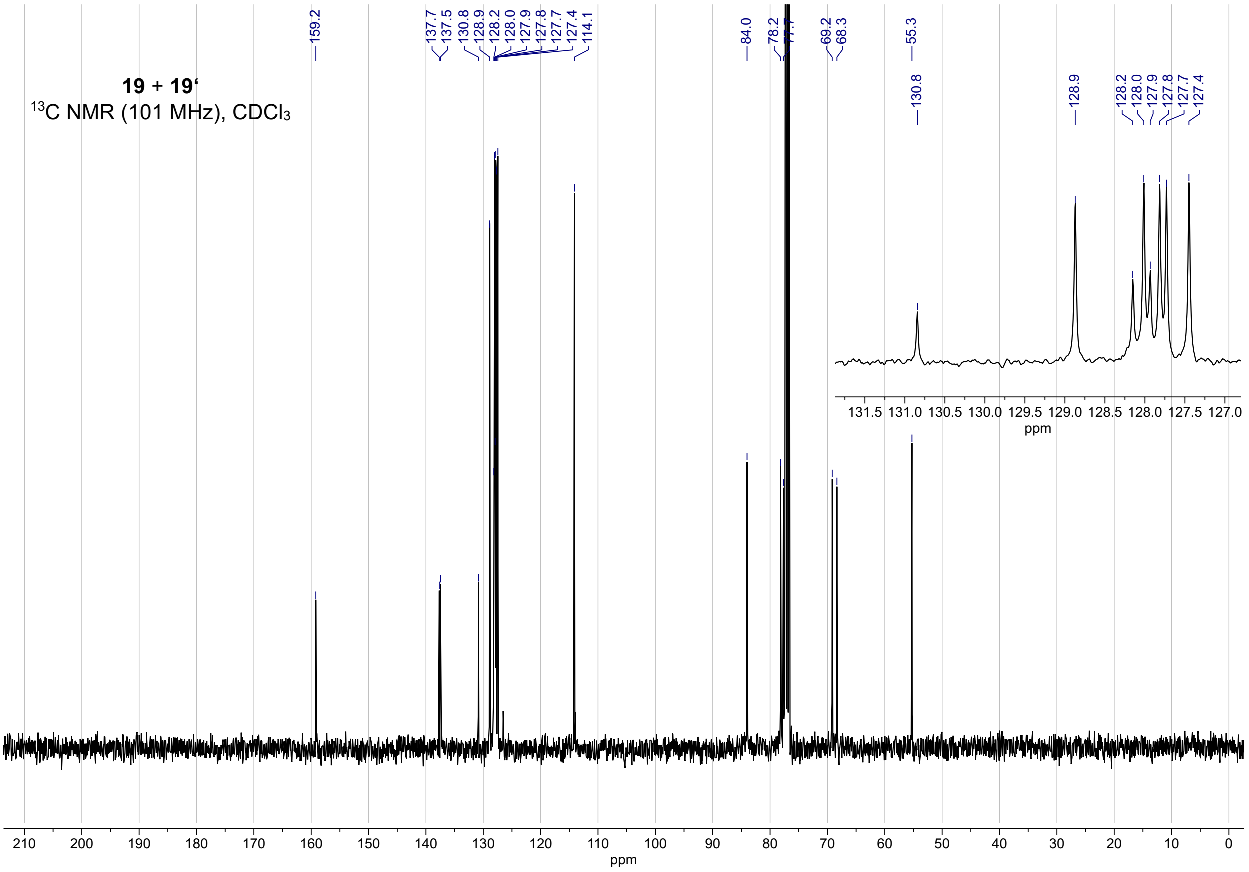
19 + 19'
13C NMR (101 MHz), CDCl3
ppm

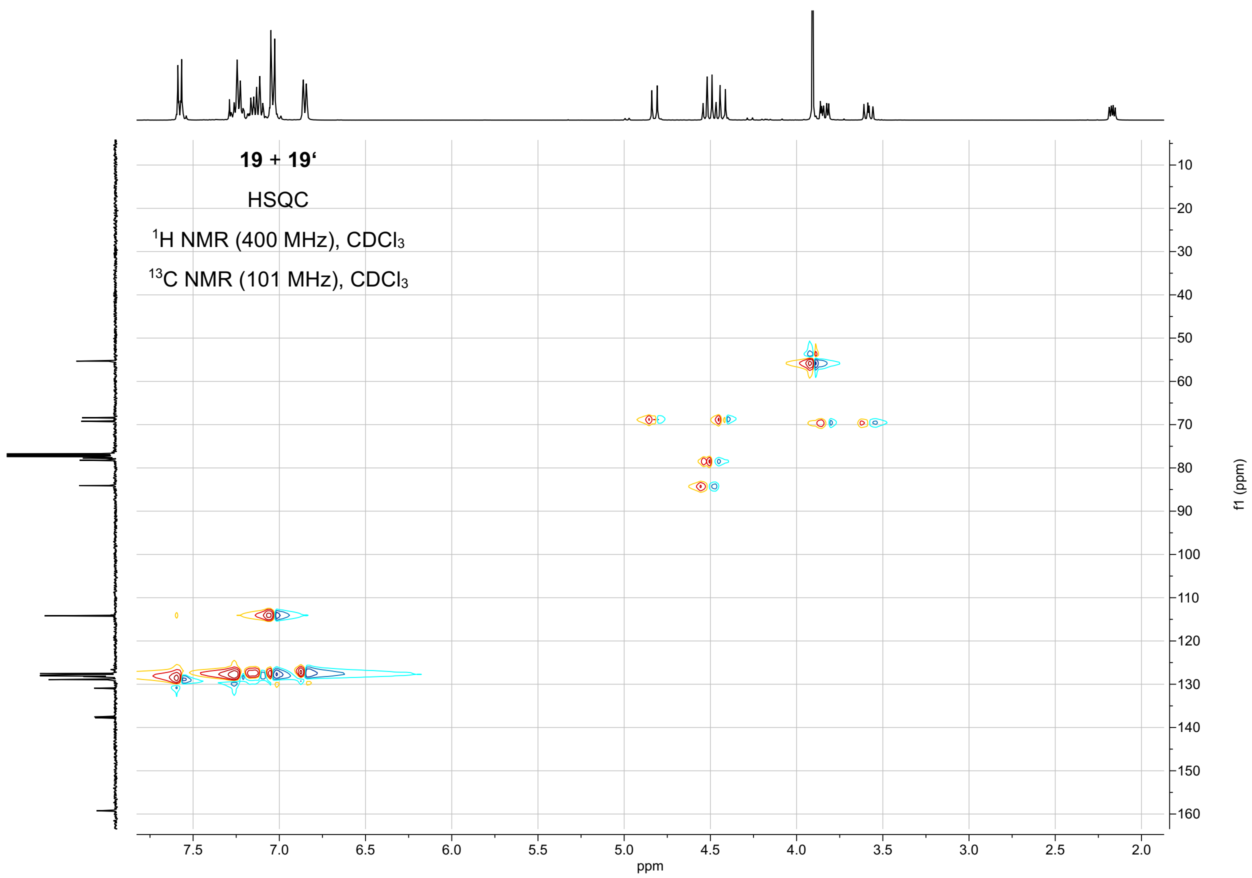
19 + 19'
HSQC
^{1}H NMR (400 MHz), CDCl$_3$
^{13}C NMR (101 MHz), CDCl$_3$
ppm
f1 (ppm)

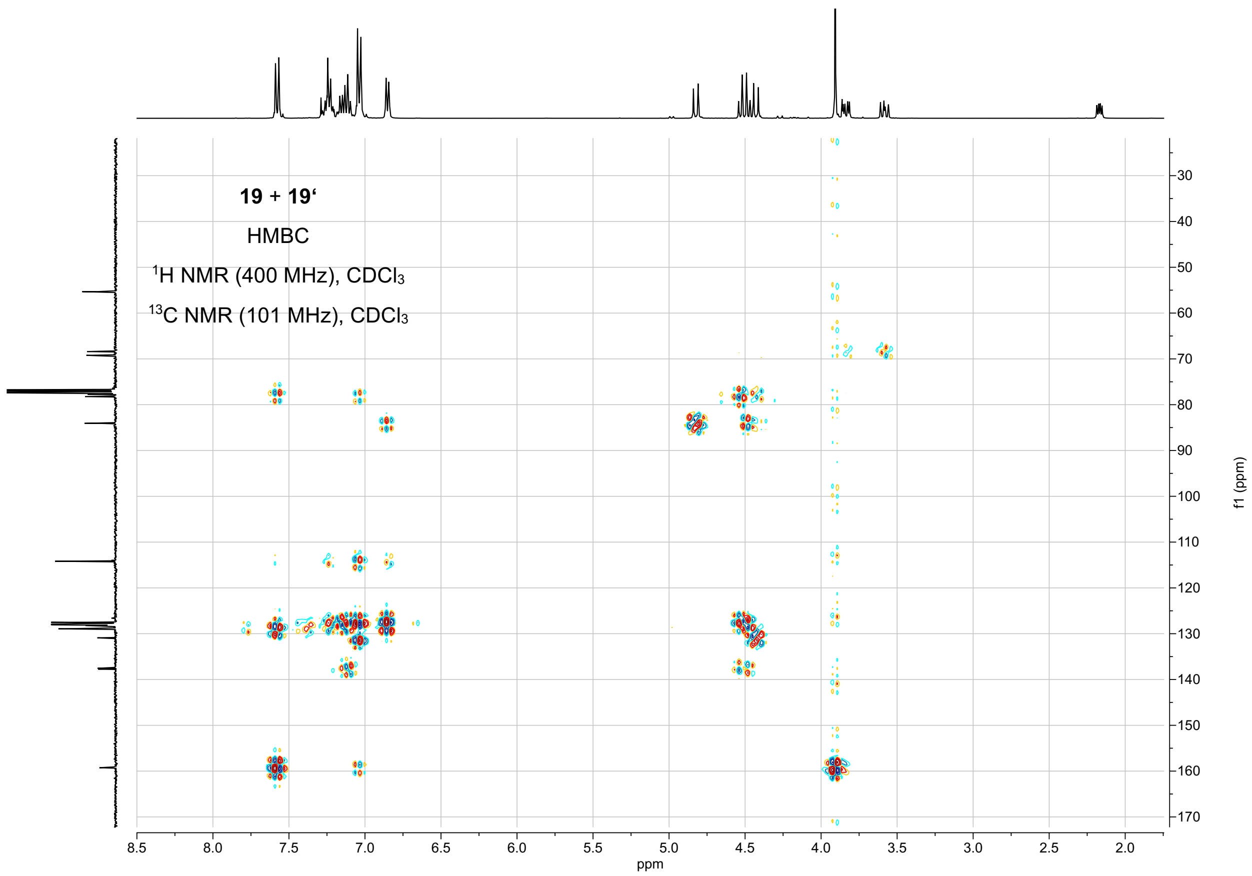
19 + 19'
HMBC
^{1}H NMR (400 MHz), CDCl$_3$
^{13}C NMR (101 MHz), CDCl$_3$
ppm
f1 (ppm)

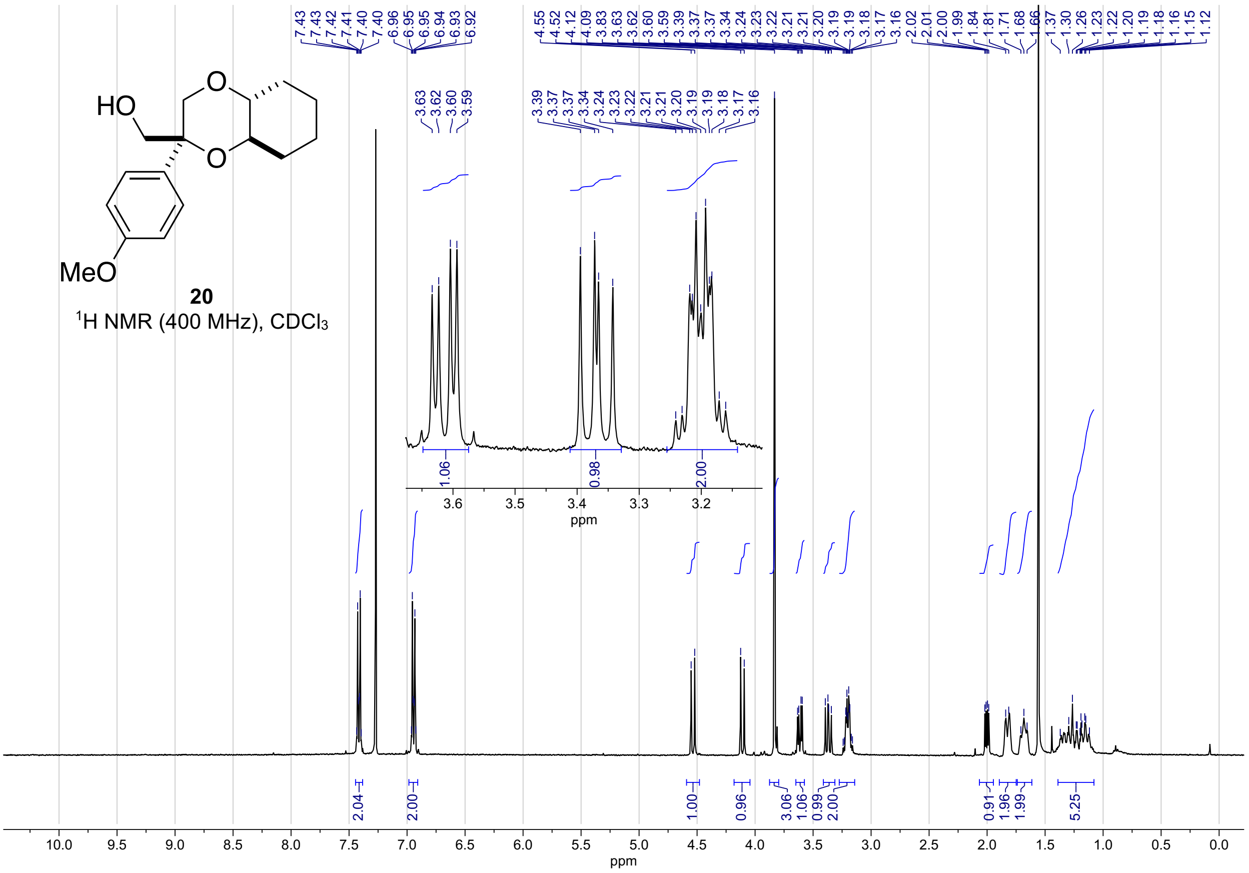
HO
O
O
MeO
20
^{1}H NMR (400 MHz), CDCl$_3$
ppm

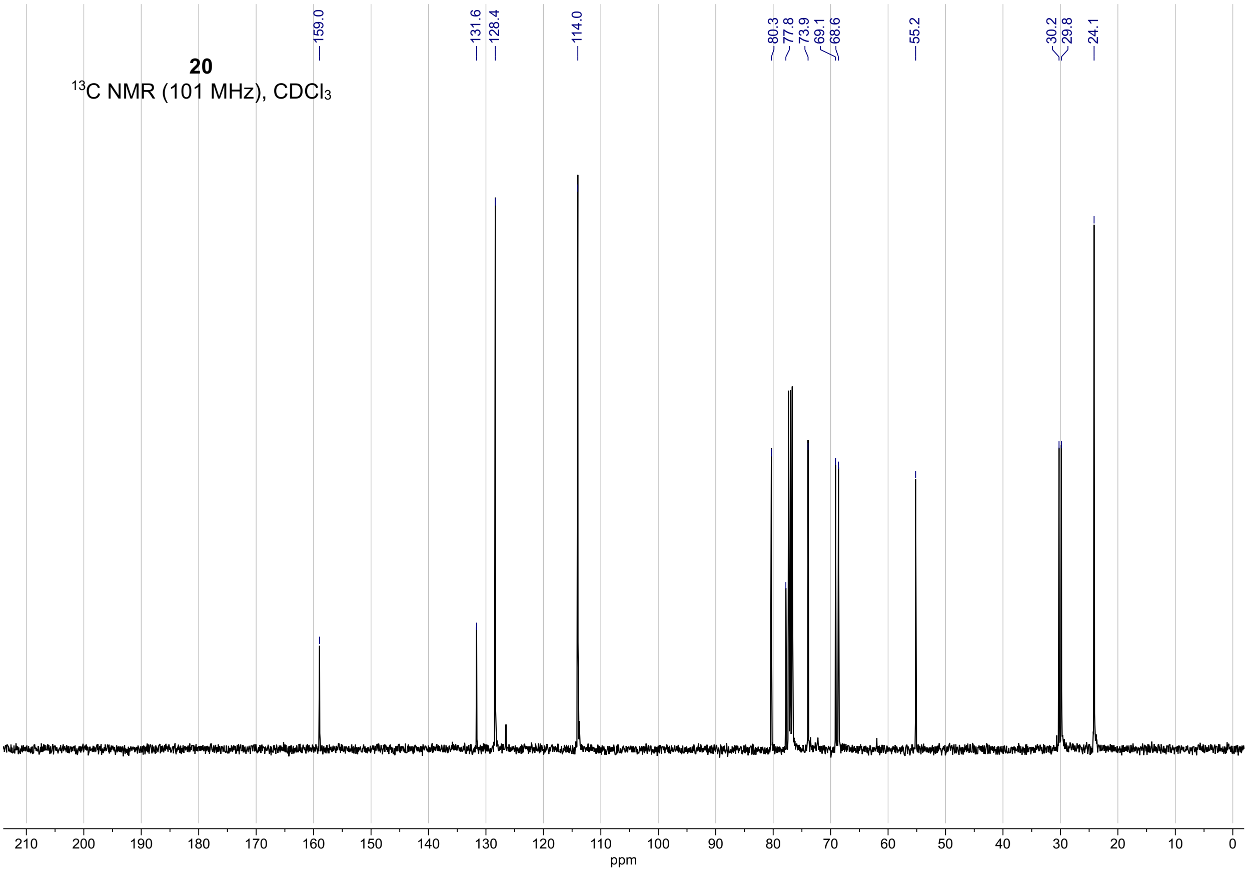
20
^{13}C NMR (101 MHz), CDCl$_3$
159.0
131.6
128.4
114.0
80.3
77.8
73.9
69.1
68.6
55.2
30.2
29.8
24.1
ppm

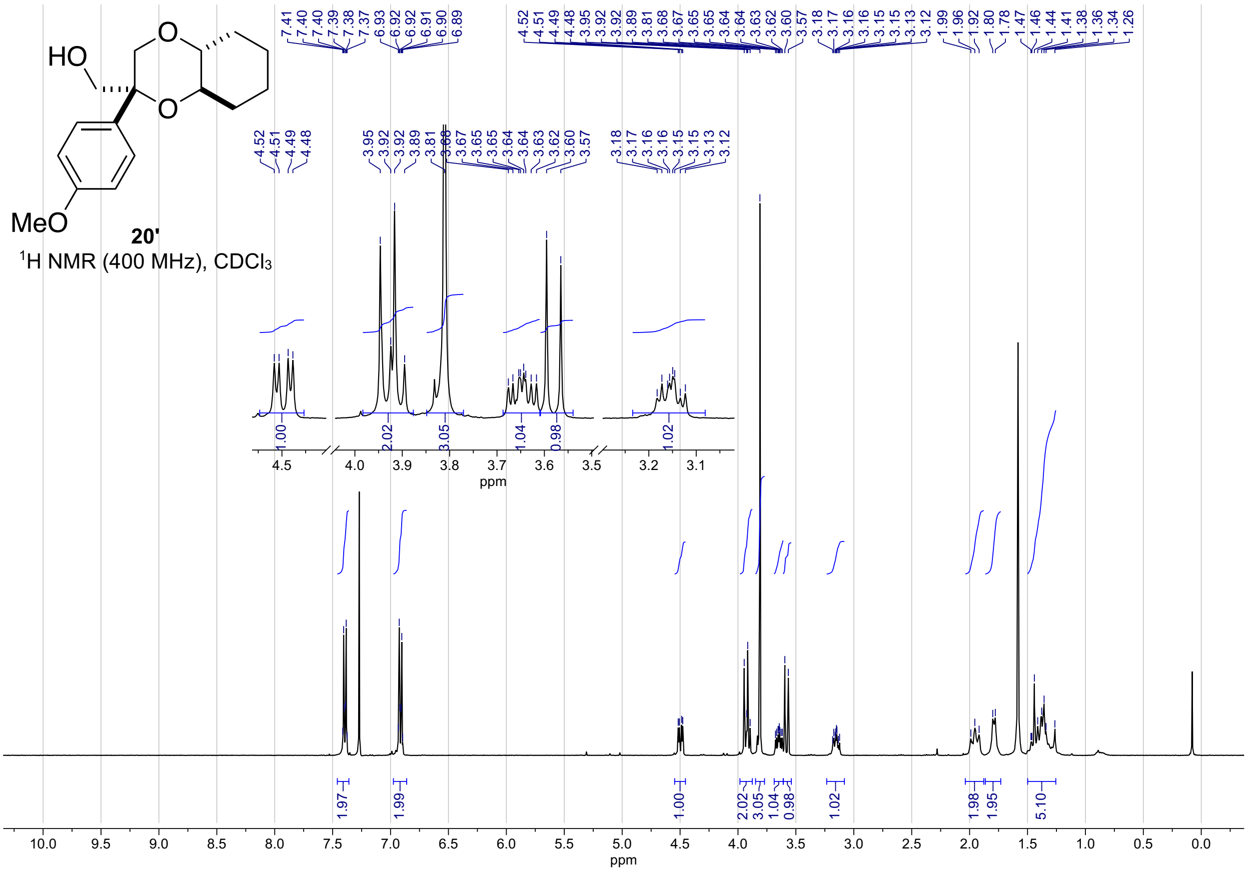
HO
O
O
MeO
20'
¹H NMR (400 MHz), CDCl3
ppm

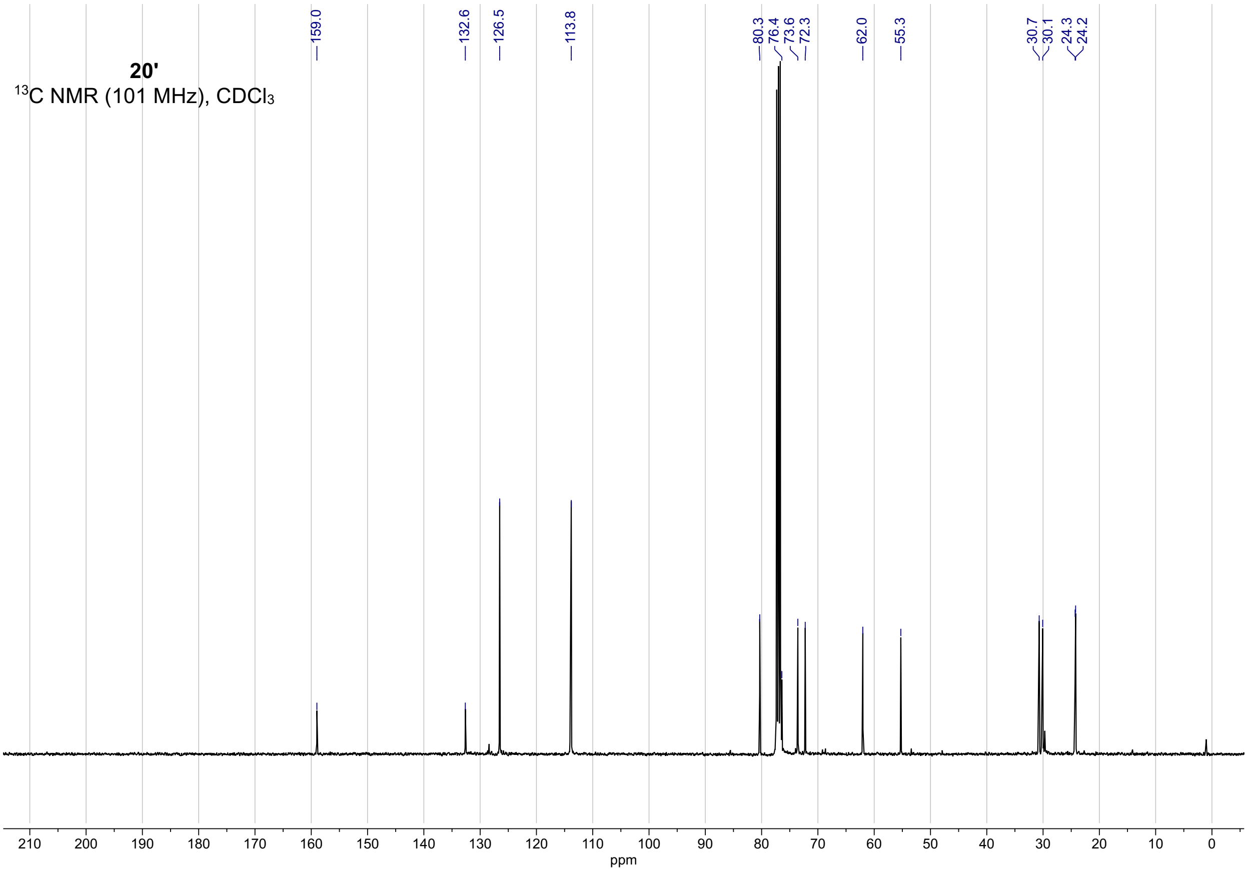
20'
¹³C NMR (101 MHz), CDCl3
159.0
132.6
126.5
113.8
80.3
76.4
73.6
72.3
62.0
55.3
30.7
30.1
24.3
24.2
ppm

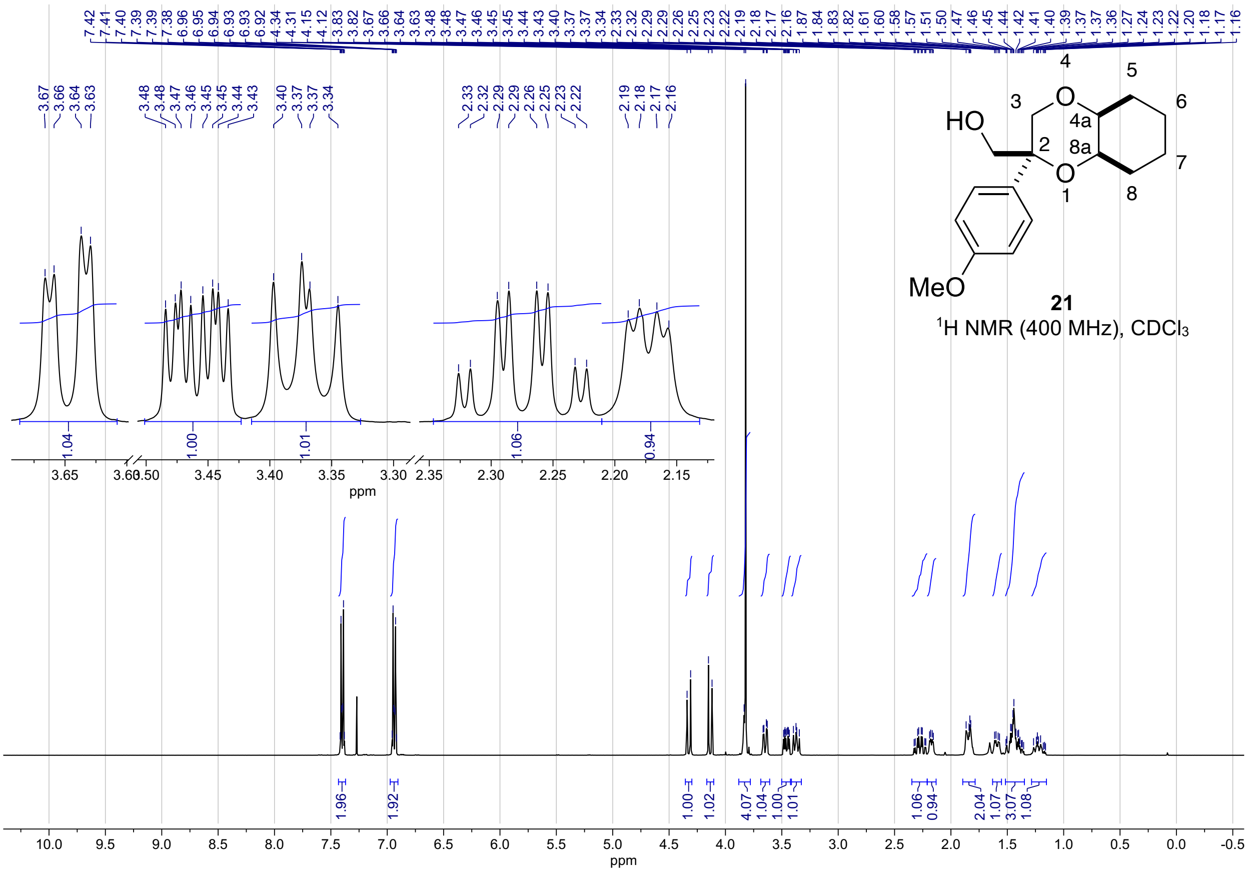
HO
MeO
21
^{1}H NMR (400 MHz), $CDCl_3$
ppm

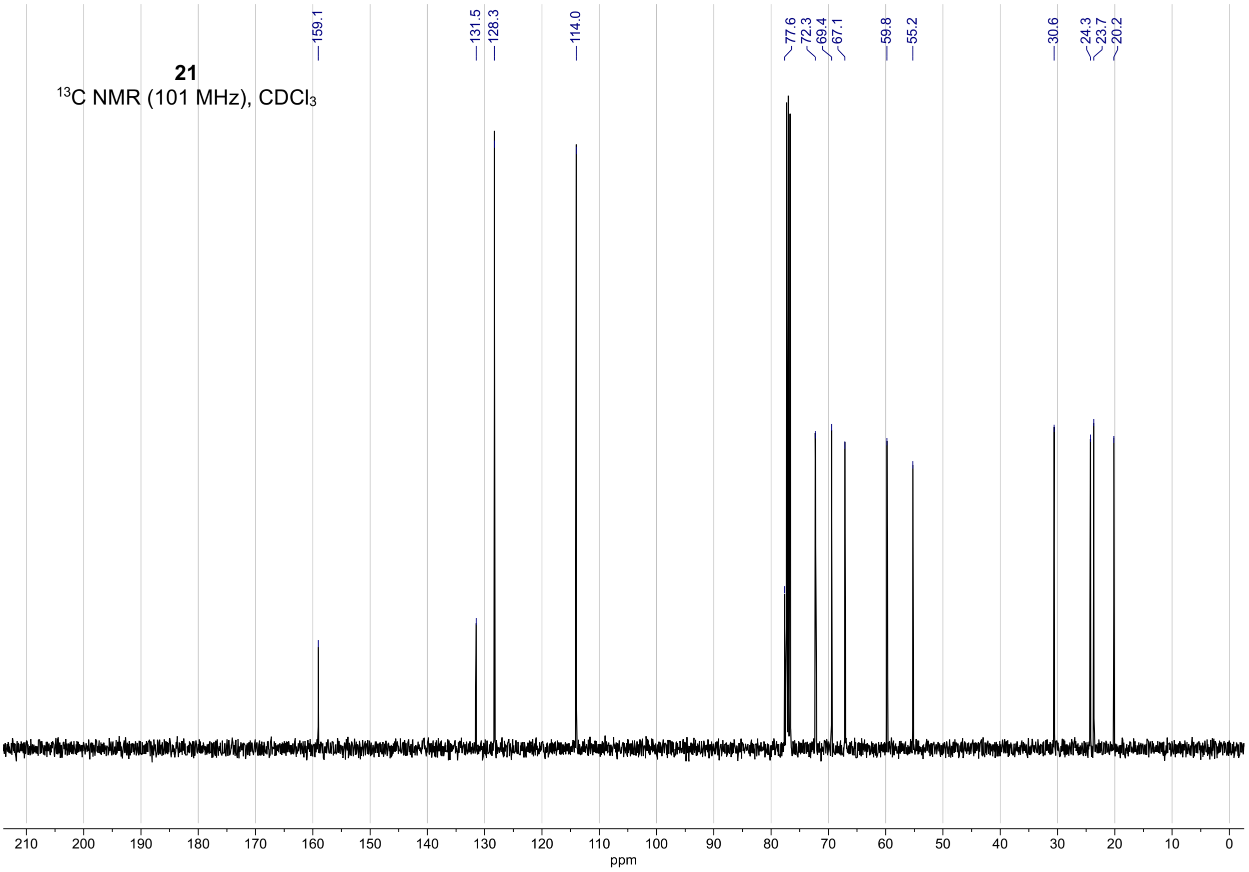
21
^{13}C NMR (101 MHz), $CDCl_3$
ppm

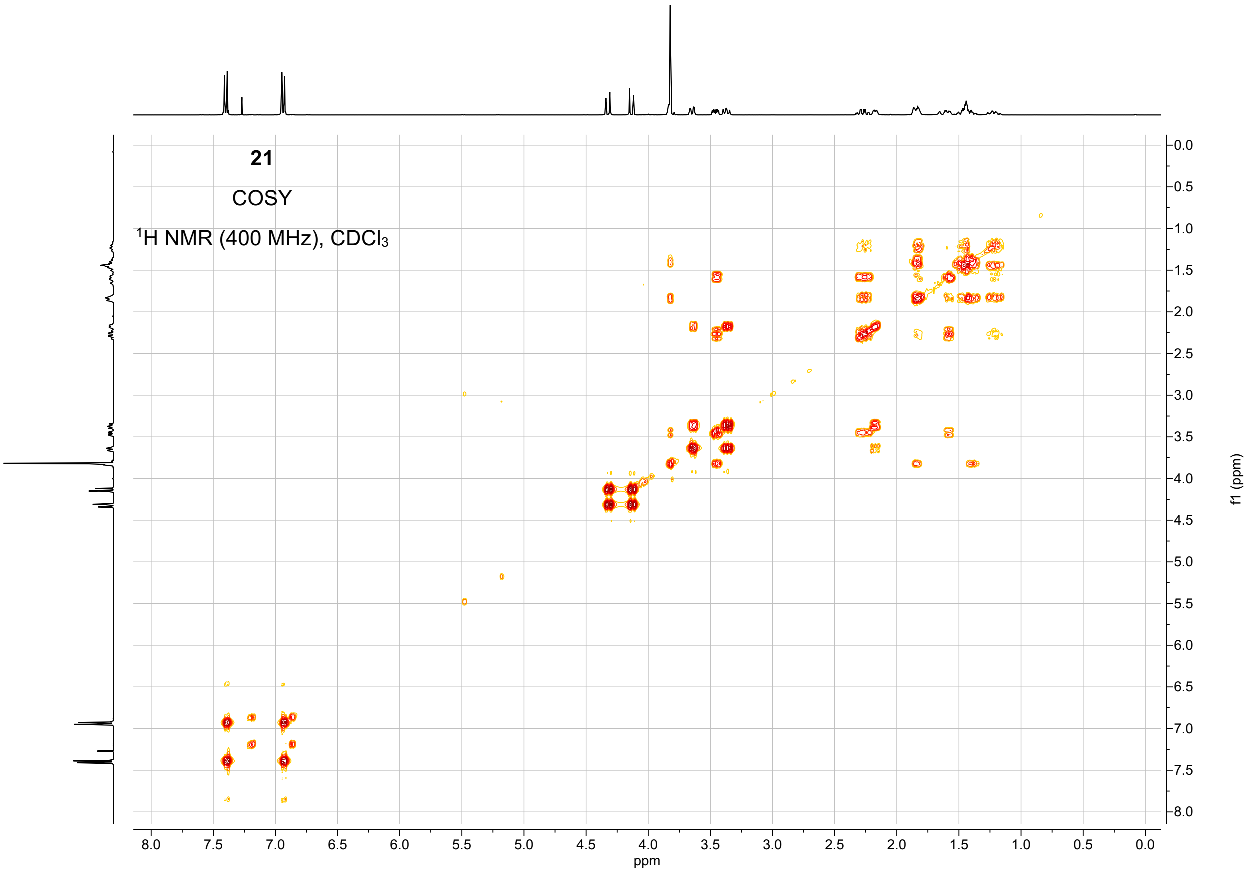

21
COSY
¹H NMR (400 MHz), CDCl3
ppm
f1 (ppm)

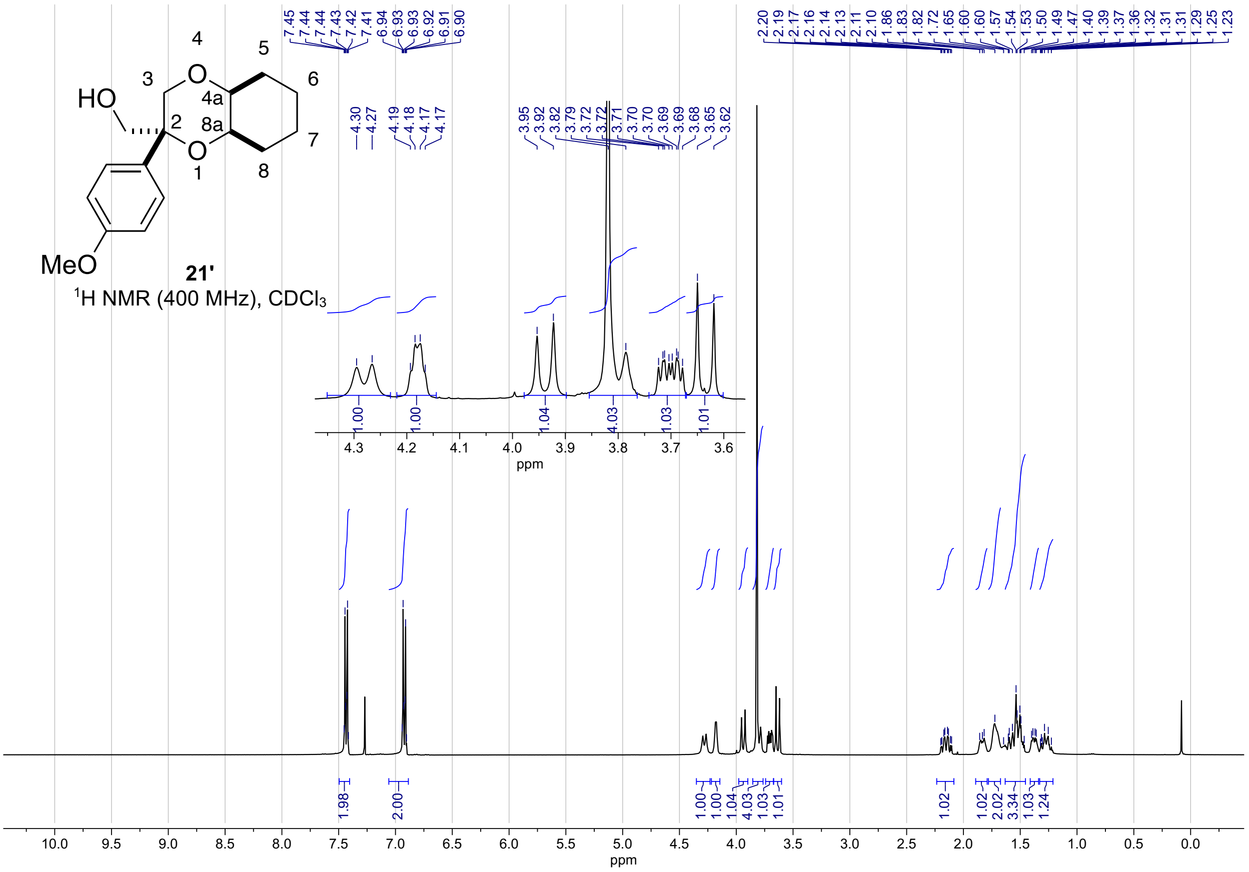

21'
^{1}H NMR (400 MHz), $CDCl_3$

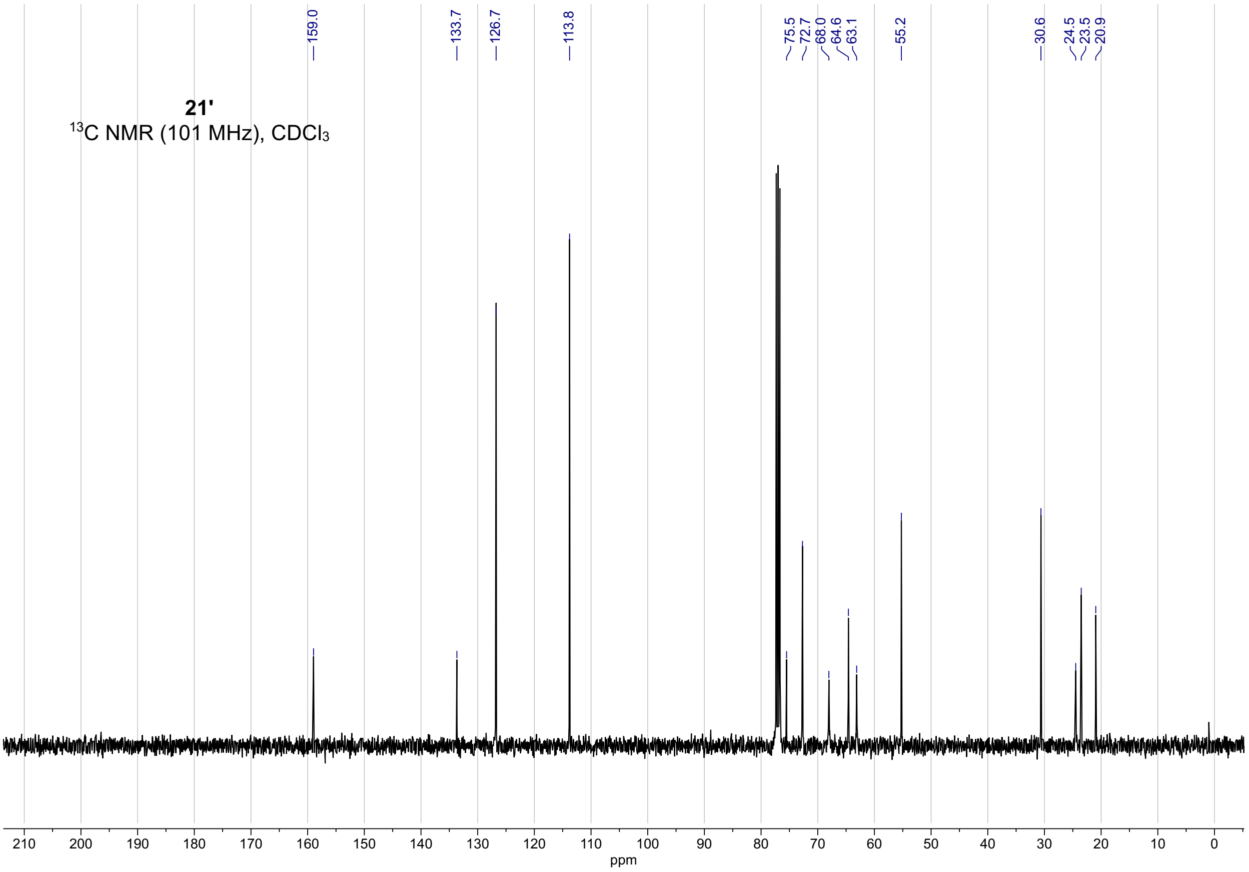

21'
^{13}C NMR (101 MHz), $CDCl_3$

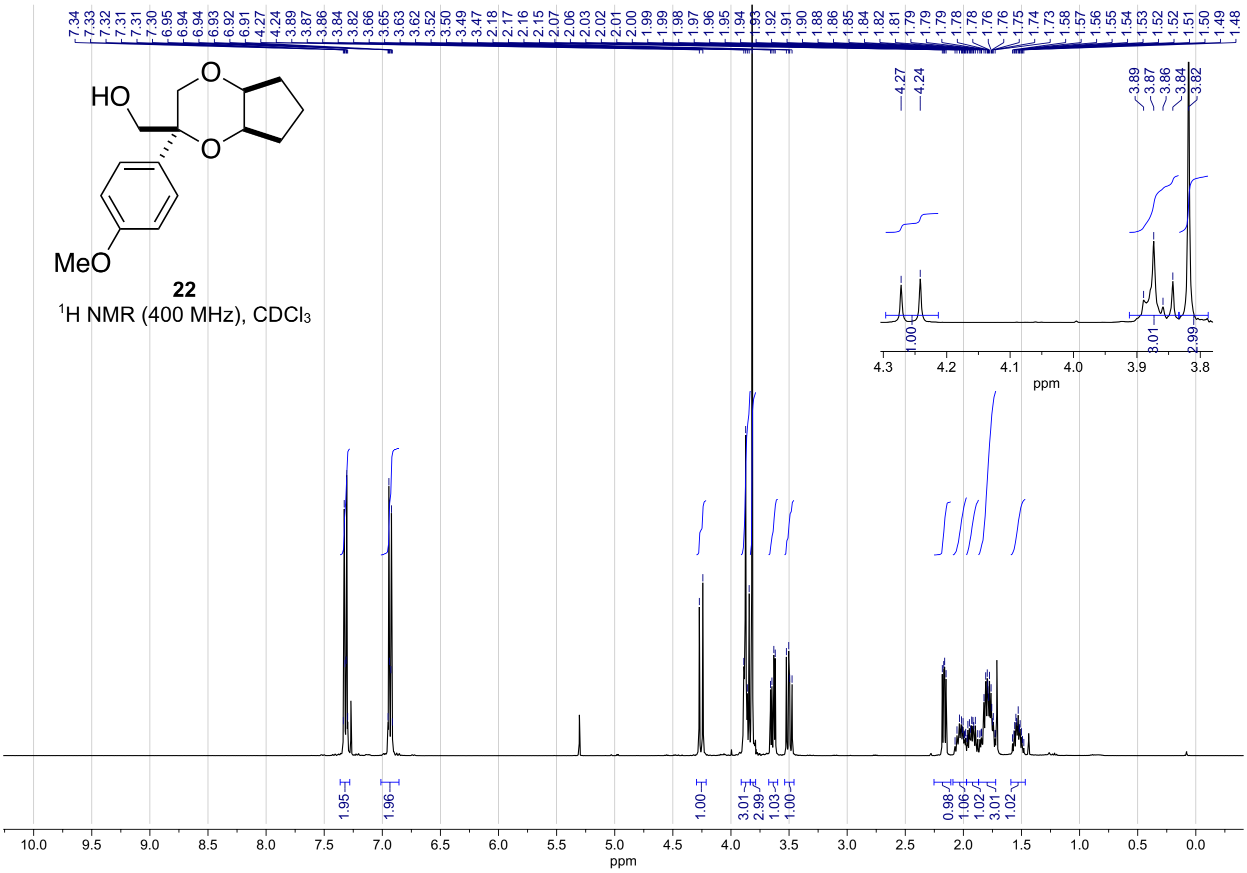

22
^{1}H NMR (400 MHz), $CDCl_3$

22
^{13}C NMR (101 MHz), $CDCl_3$

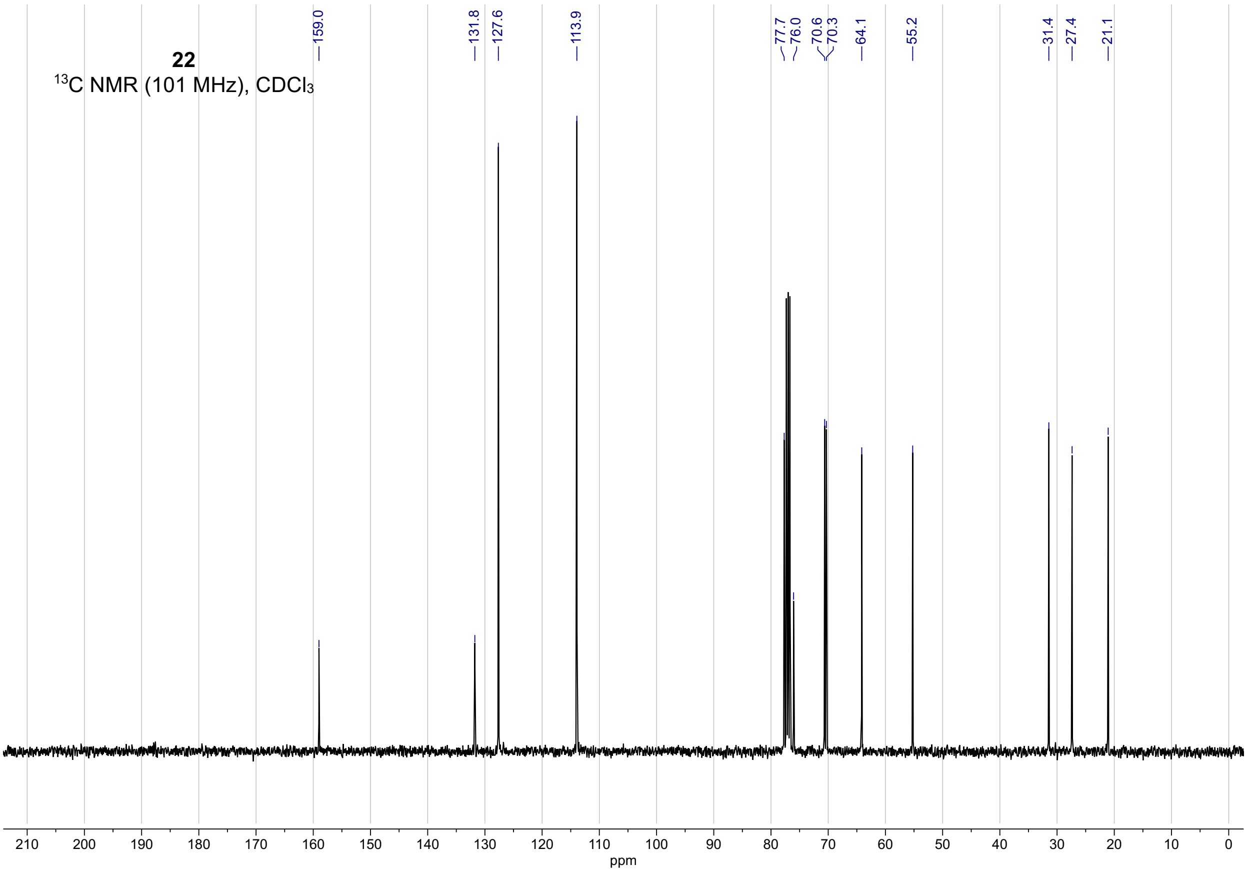

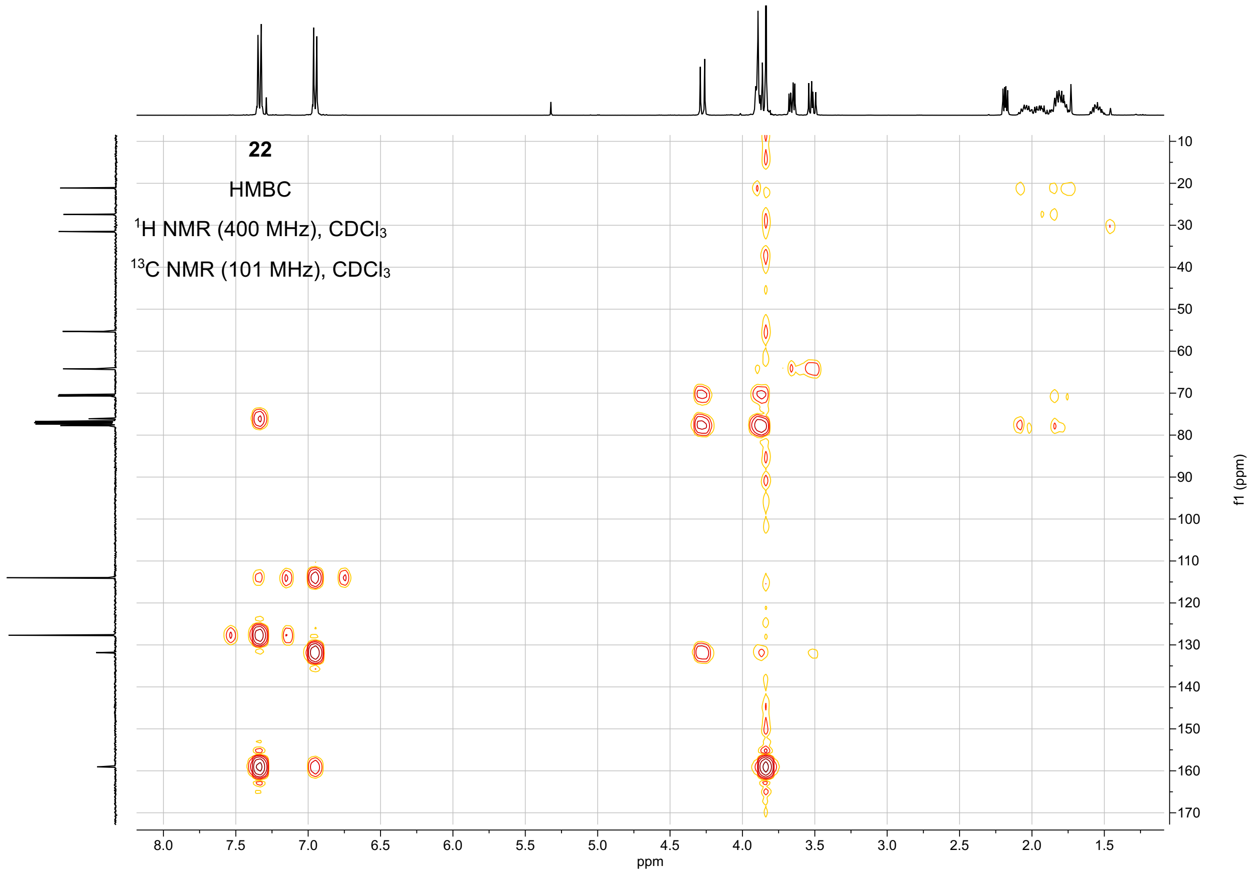
22
HMBC
^{1}H NMR (400 MHz), CDCl$_3$
^{13}C NMR (101 MHz), CDCl$_3$
8.0
7.5
7.0
6.5
6.0
5.5
5.0
4.5
4.0
3.5
3.0
2.5
2.0
1.5
ppm
f1 (ppm)
10
20
30
40
50
60
70
80
90
100
110
120
130
140
150
160
170

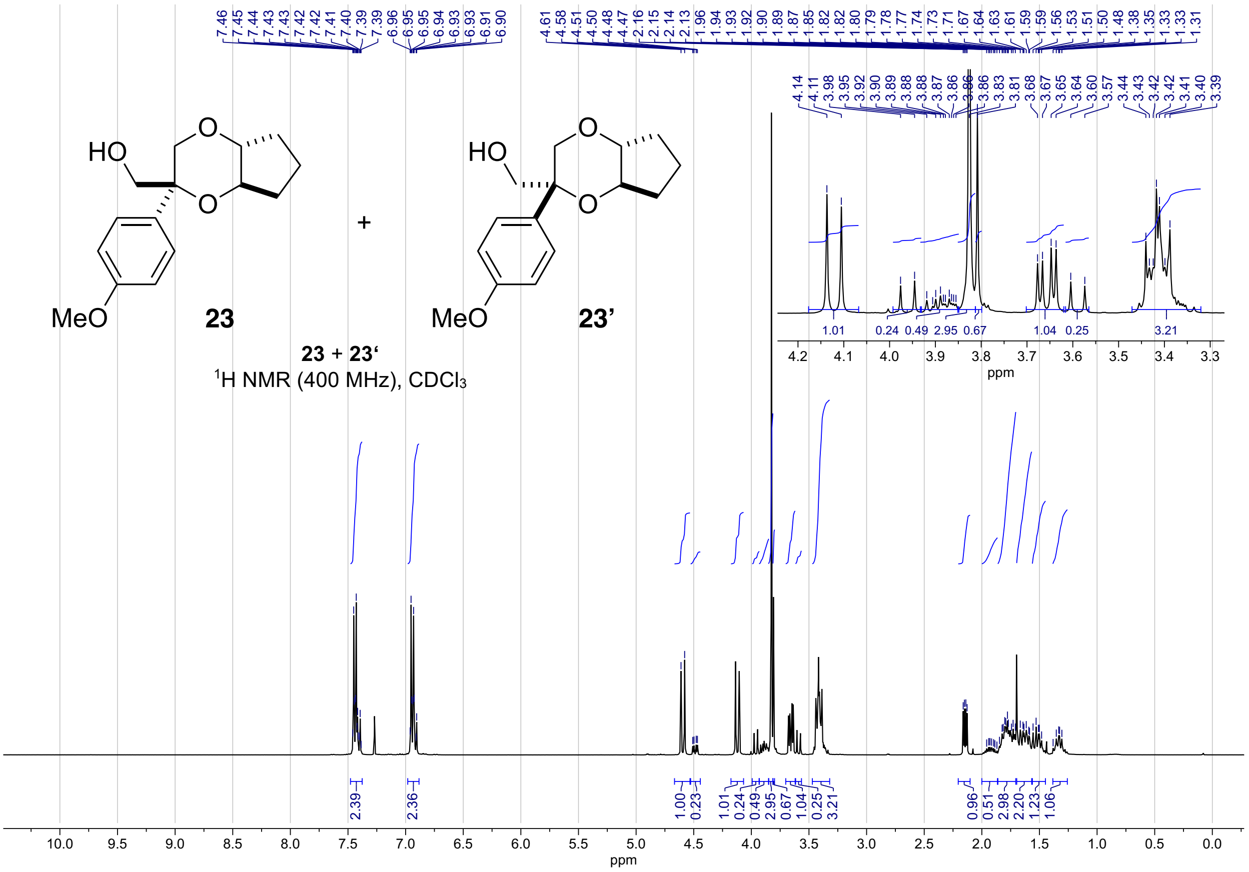
23 + 23'
^{1}H NMR (400 MHz), CDCl$_3$
23
23'
HO
MeO
ppm

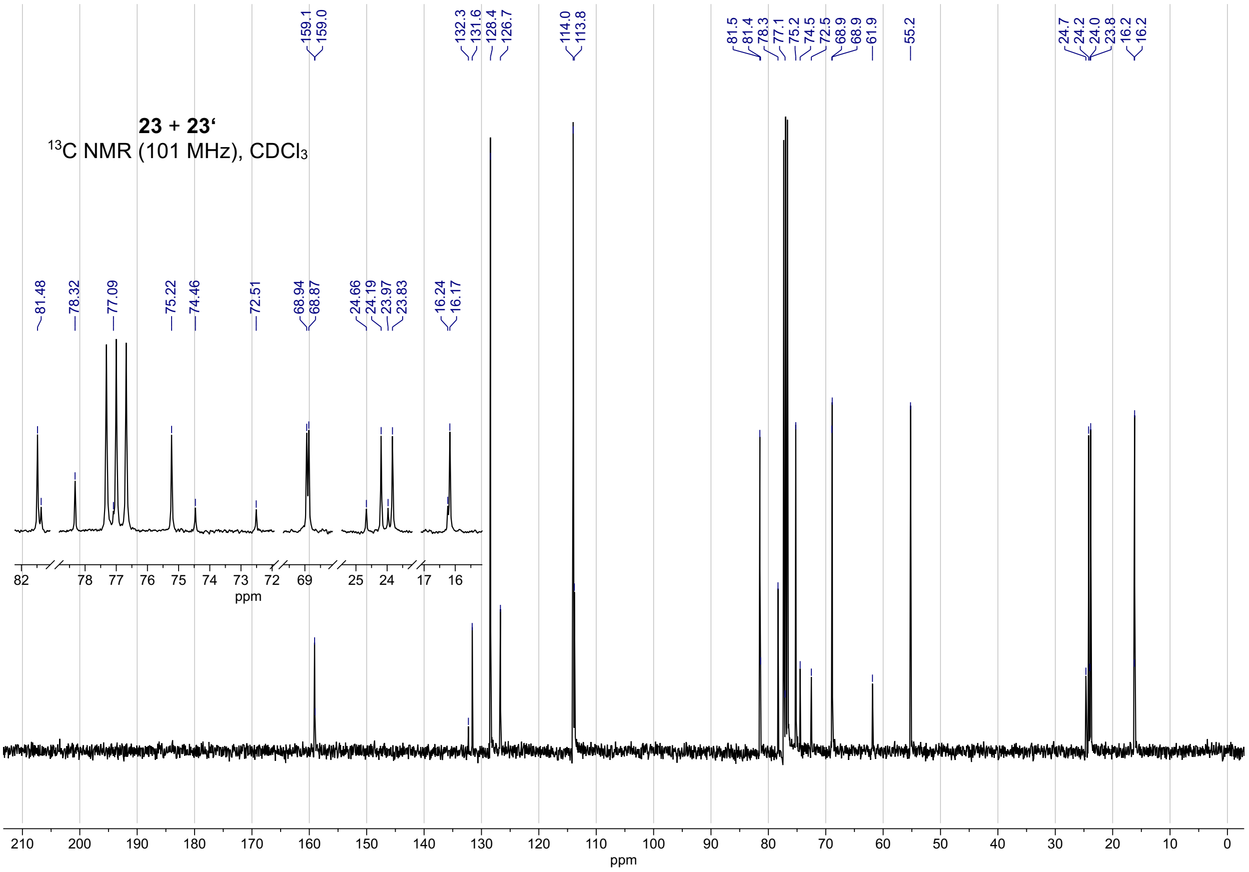
23 + 23'
^{13}C NMR (101 MHz), CDCl$_3$
ppm

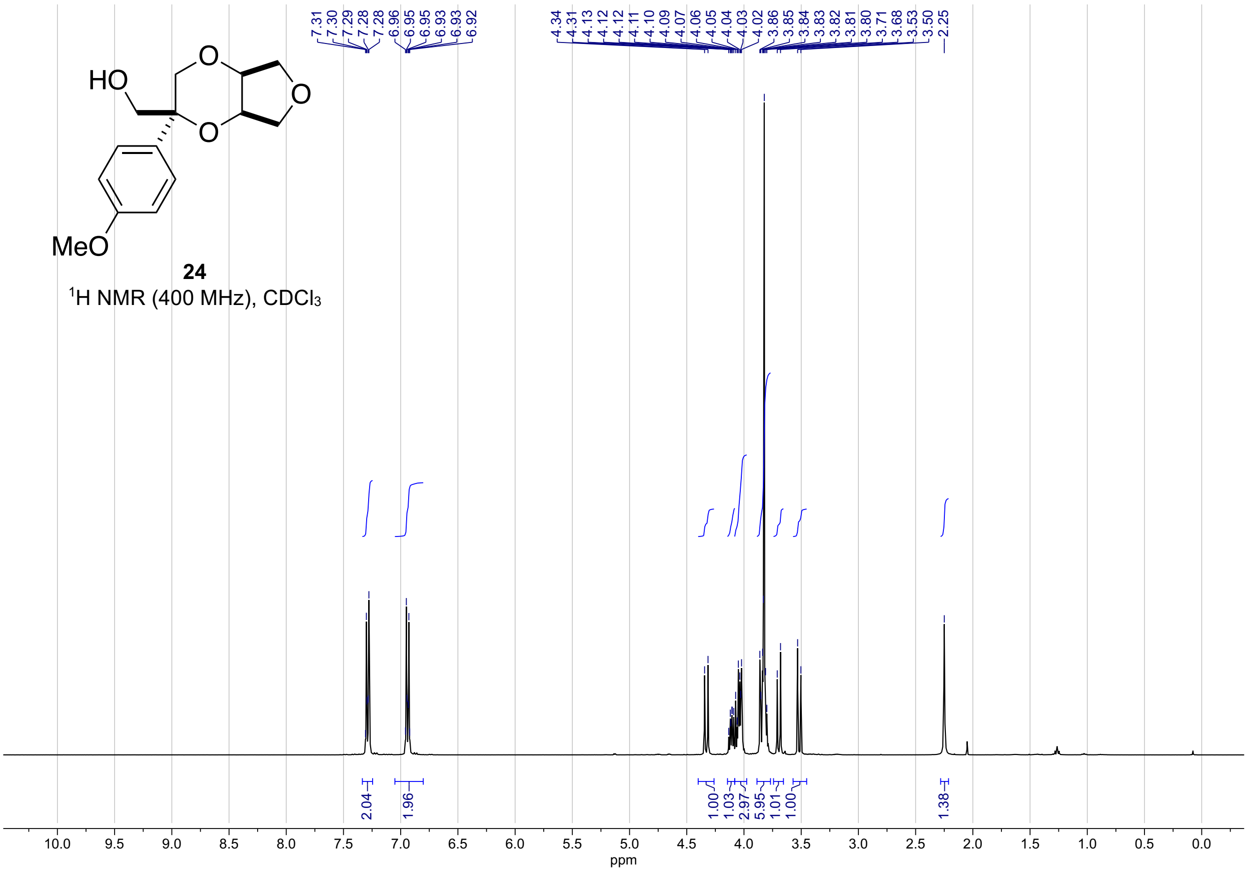
HO
O
O
O
MeO
24
^{1}H NMR (400 MHz), CDCl$_3$
7.31
7.30
7.29
7.28
7.28
6.96
6.95
6.95
6.93
6.93
6.92
4.34
4.31
4.13
4.12
4.12
4.11
4.10
4.09
4.07
4.06
4.05
4.04
4.03
4.02
3.86
3.85
3.84
3.83
3.82
3.81
3.80
3.71
3.68
3.53
3.50
2.25
2.04
1.96
1.00
1.03
2.97
5.95
1.01
1.00
1.38
10.0 9.5 9.0 8.5 8.0 7.5 7.0 6.5 6.0 5.5 5.0 4.5 4.0 3.5 3.0 2.5 2.0 1.5 1.0 0.5 0.0
ppm

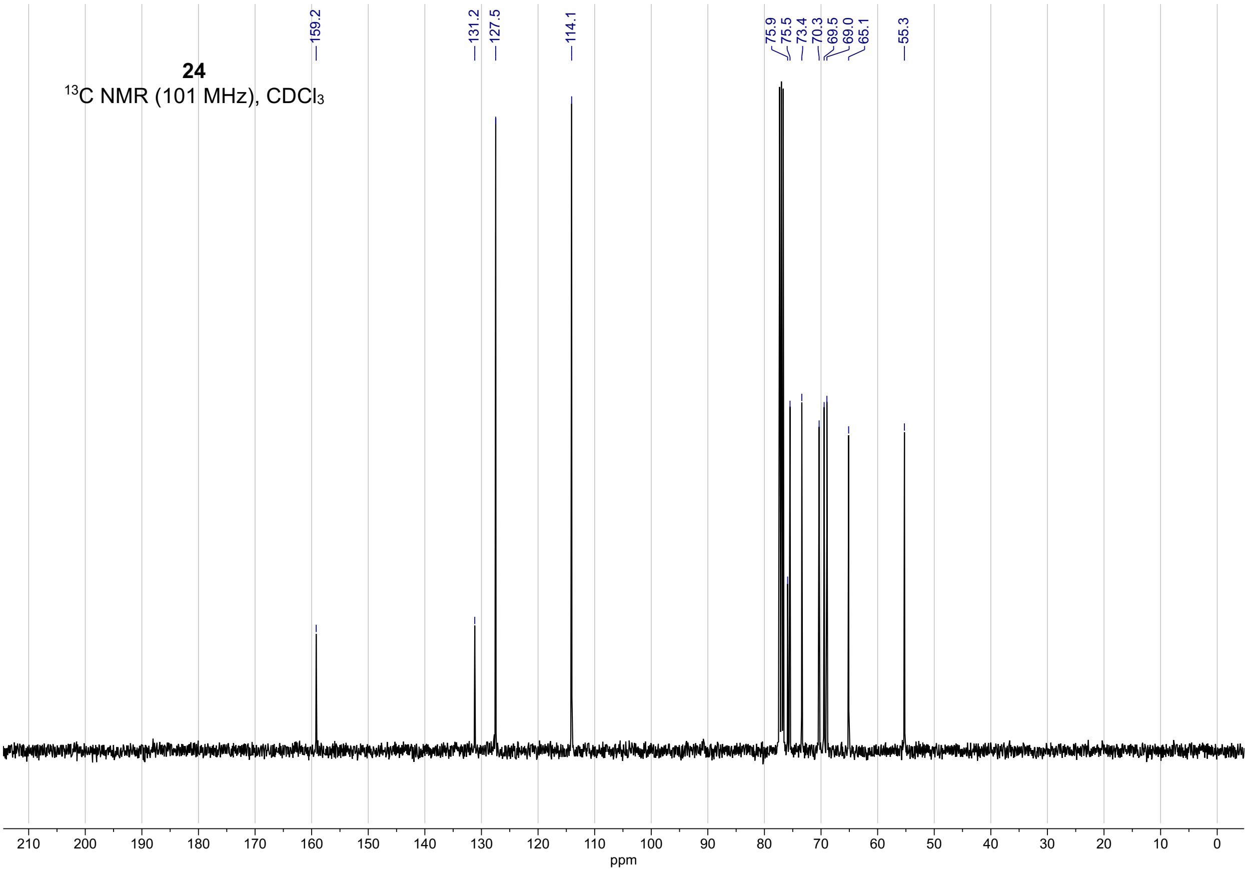
24
^{13}C NMR (101 MHz), CDCl$_3$
159.2
131.2
127.5
114.1
75.9
75.5
73.4
70.3
69.5
69.0
65.1
55.3
210 200 190 180 170 160 150 140 130 120 110 100 90 80 70 60 50 40 30 20 10 0
ppm

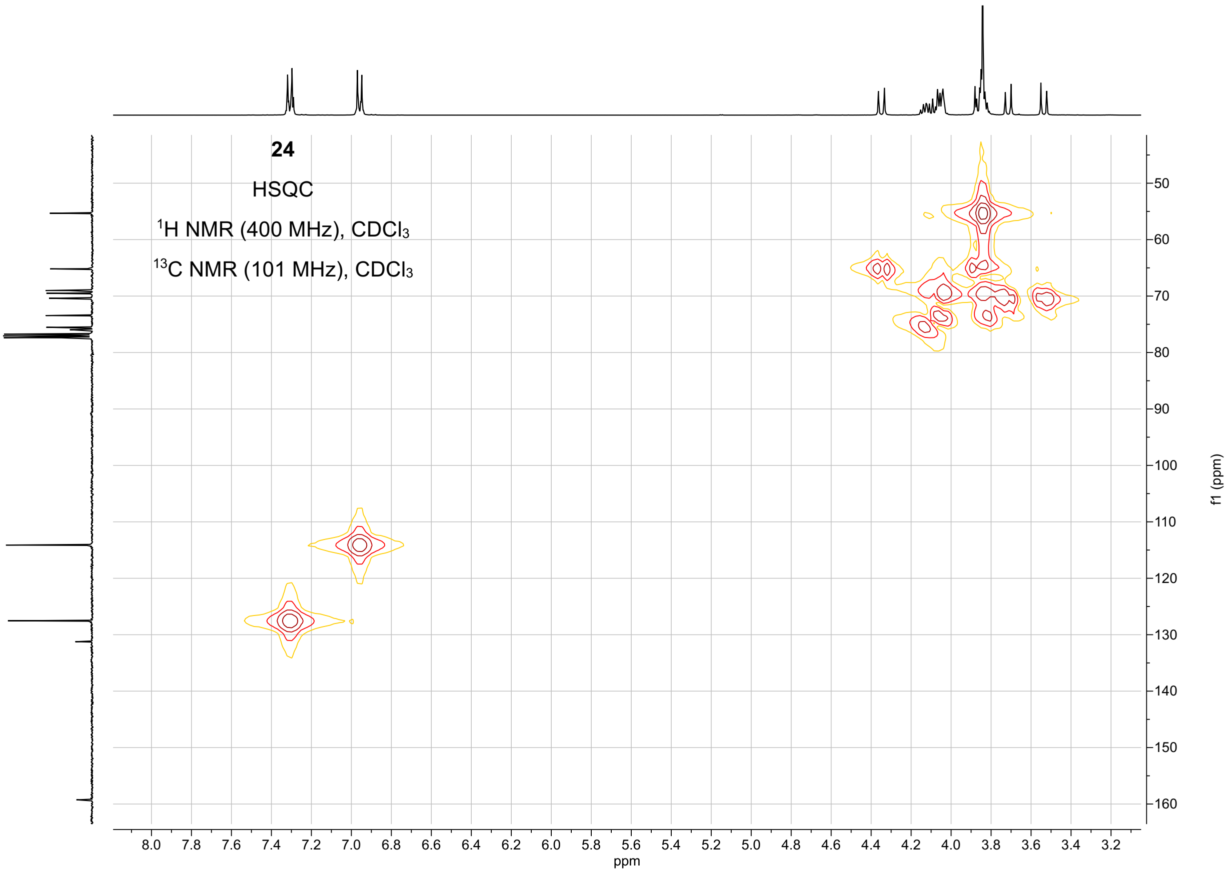
24
HSQC
^{1}H NMR (400 MHz), $CDCl_3$
^{13}C NMR (101 MHz), $CDCl_3$
ppm
f1 (ppm)

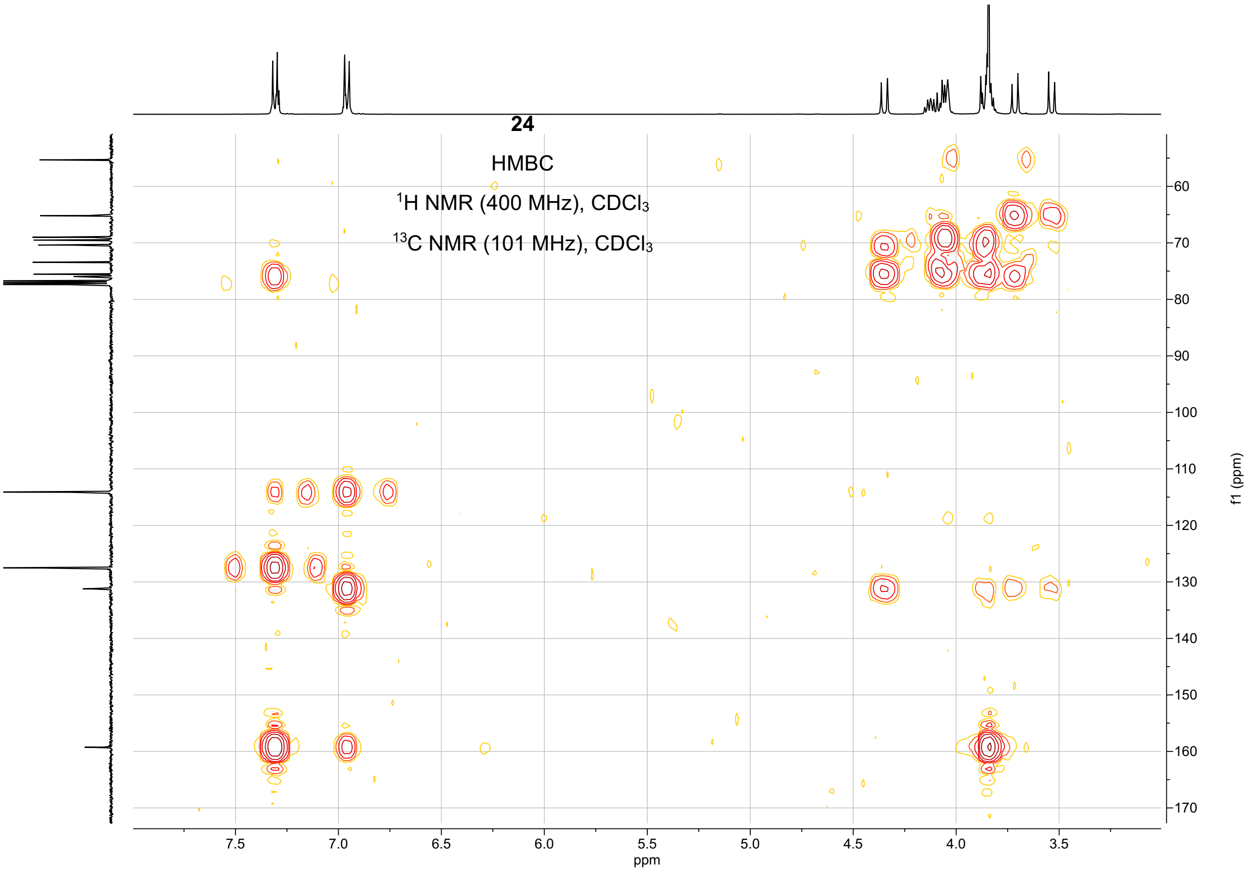
24
HMBC
^{1}H NMR (400 MHz), $CDCl_3$
^{13}C NMR (101 MHz), $CDCl_3$
ppm
f1 (ppm)

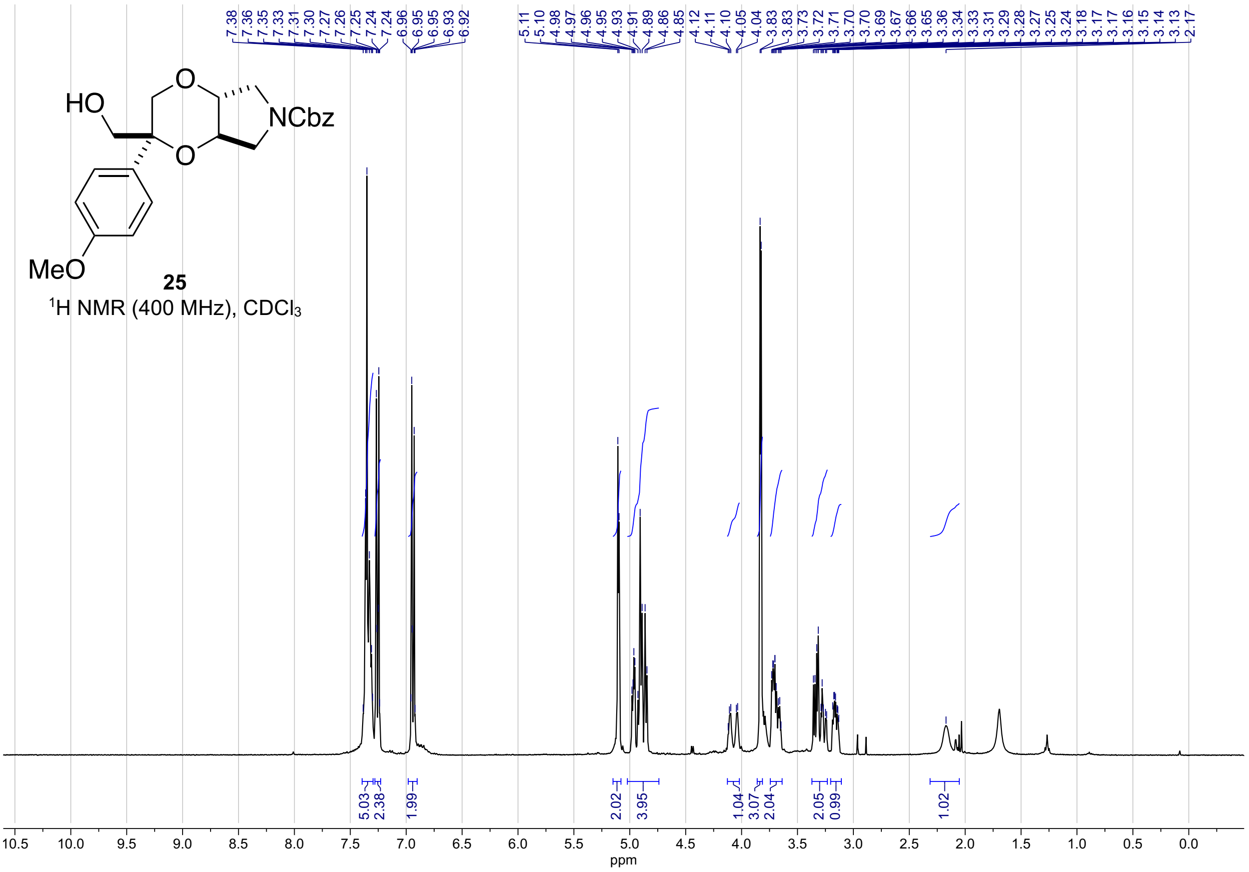

25

^{1}H NMR (400 MHz), $CDCl_3$

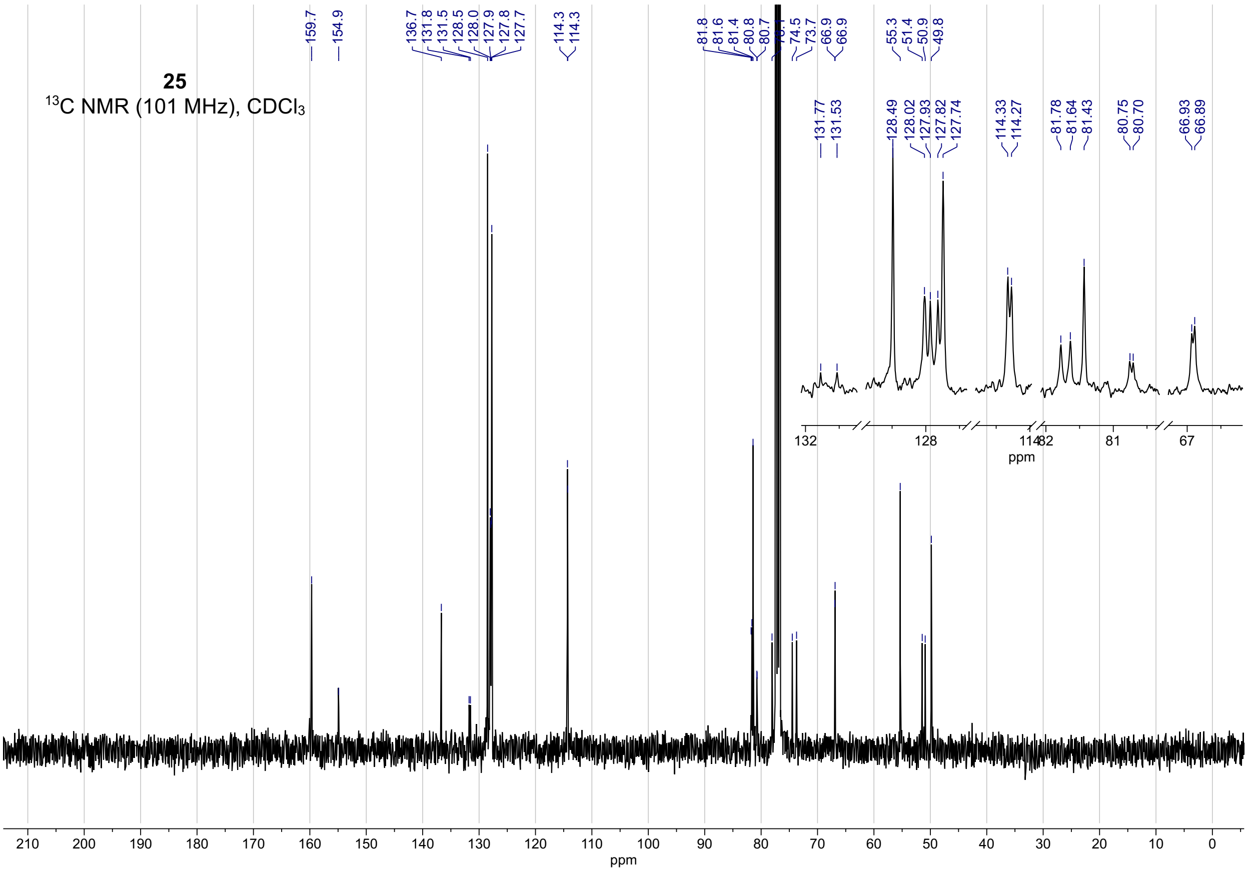

25

^{13}C NMR (101 MHz), $CDCl_3$

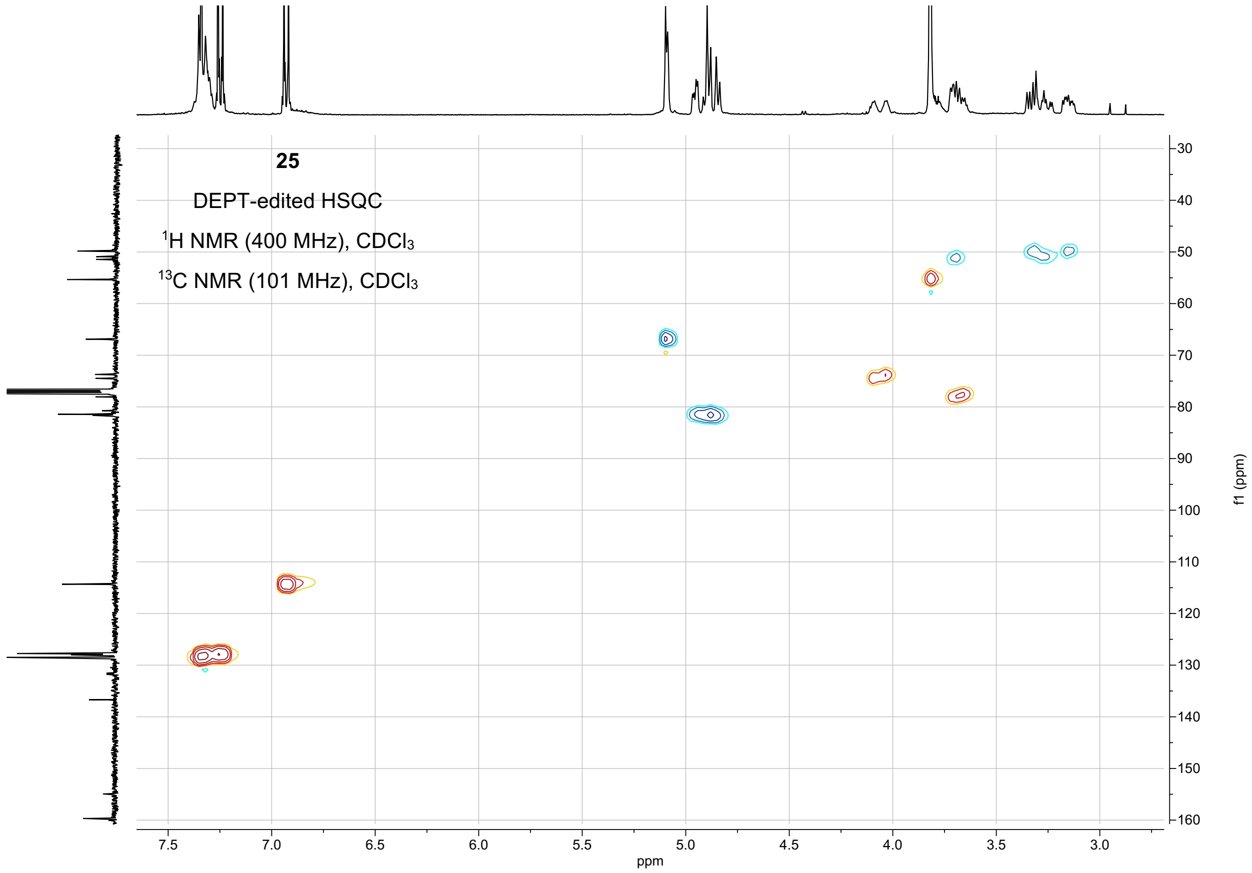
25
DEPT-edited HSQC
1H NMR (400 MHz), CDCl3
13C NMR (101 MHz), CDCl3
ppm
f1 (ppm)

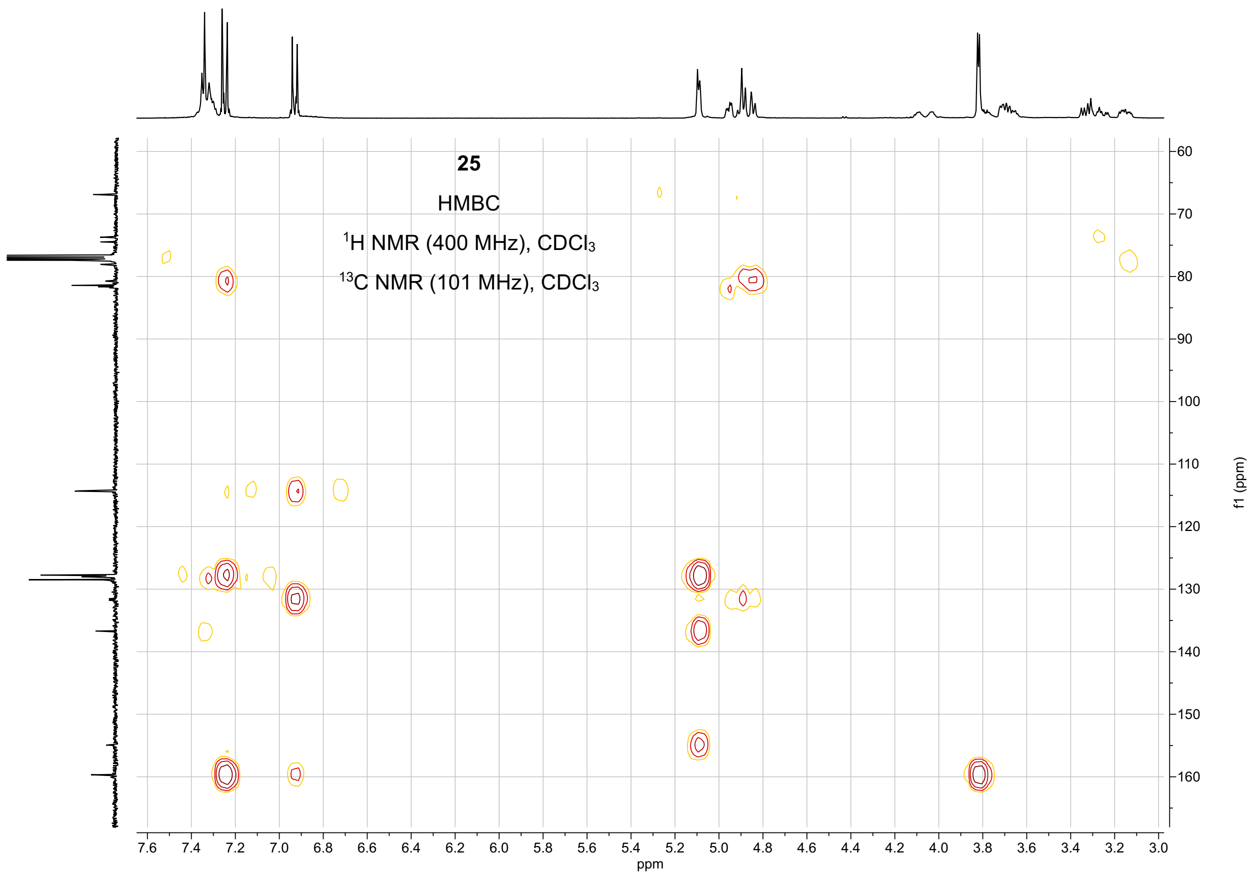
25
HMBC
1H NMR (400 MHz), CDCl3
13C NMR (101 MHz), CDCl3
ppm
f1 (ppm)

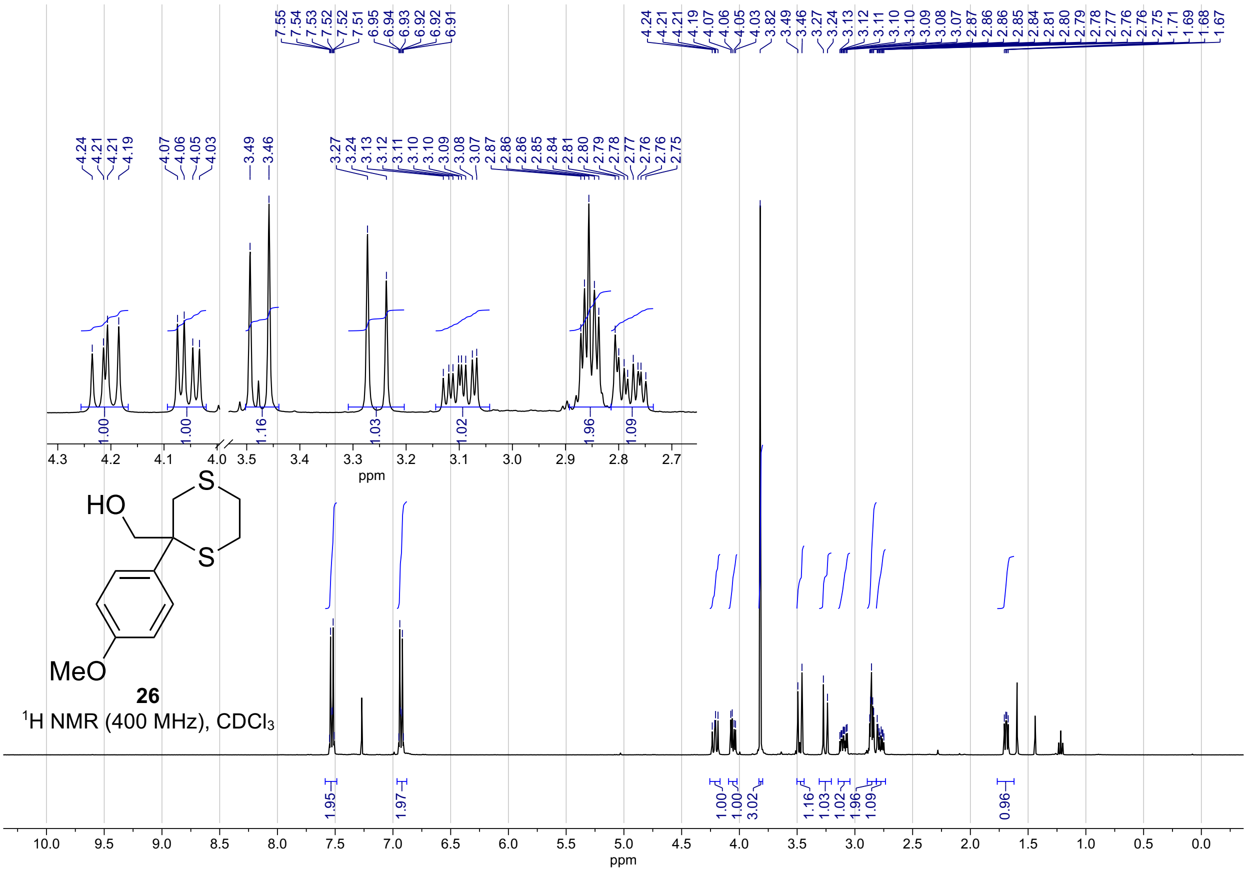
HO
S
S
MeO
26
^{1}H NMR (400 MHz), $CDCl_3$
ppm

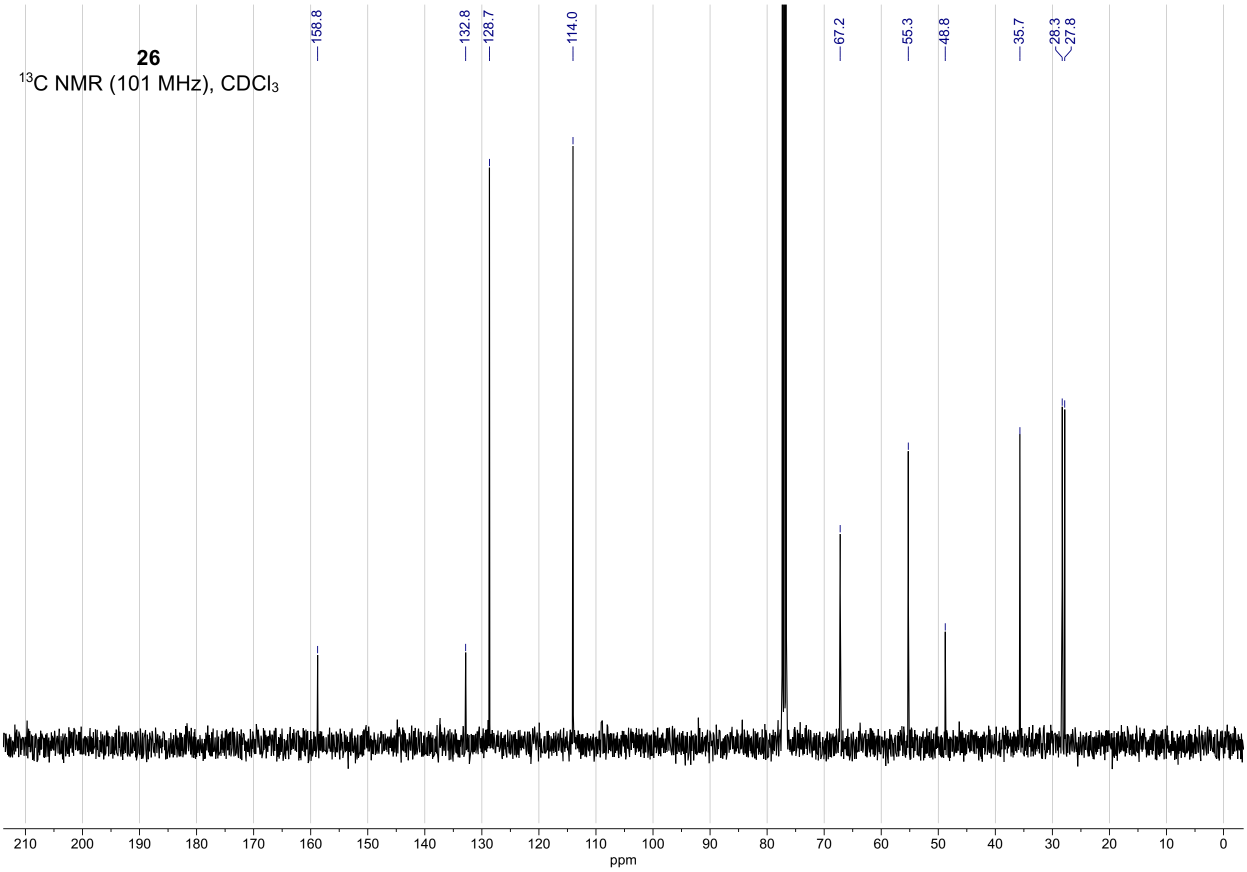
26
^{13}C NMR (101 MHz), $CDCl_3$
158.8
132.8
128.7
114.0
67.2
55.3
48.8
35.7
28.3
27.8
ppm

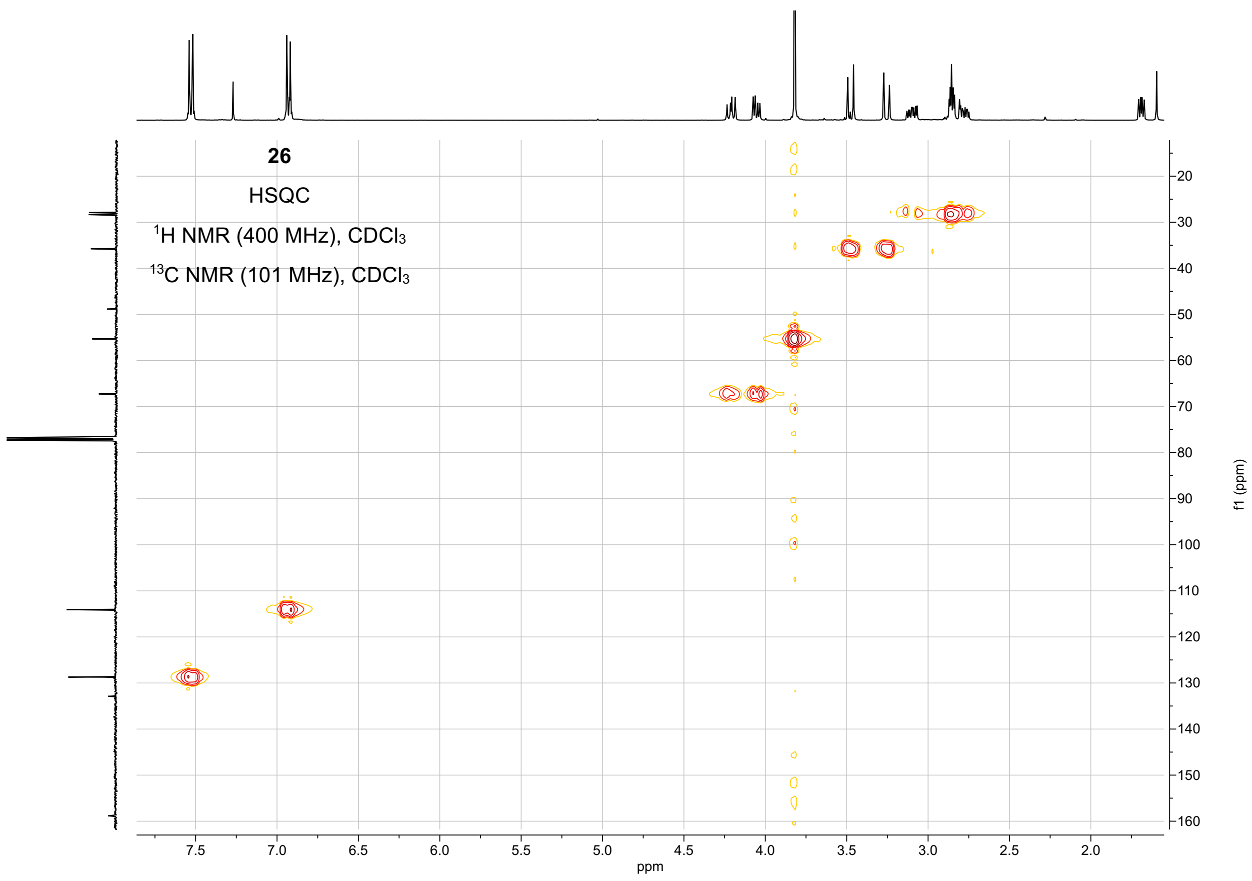
26
HSQC
1H NMR (400 MHz), CDCl3
13C NMR (101 MHz), CDCl3
ppm
f1 (ppm)

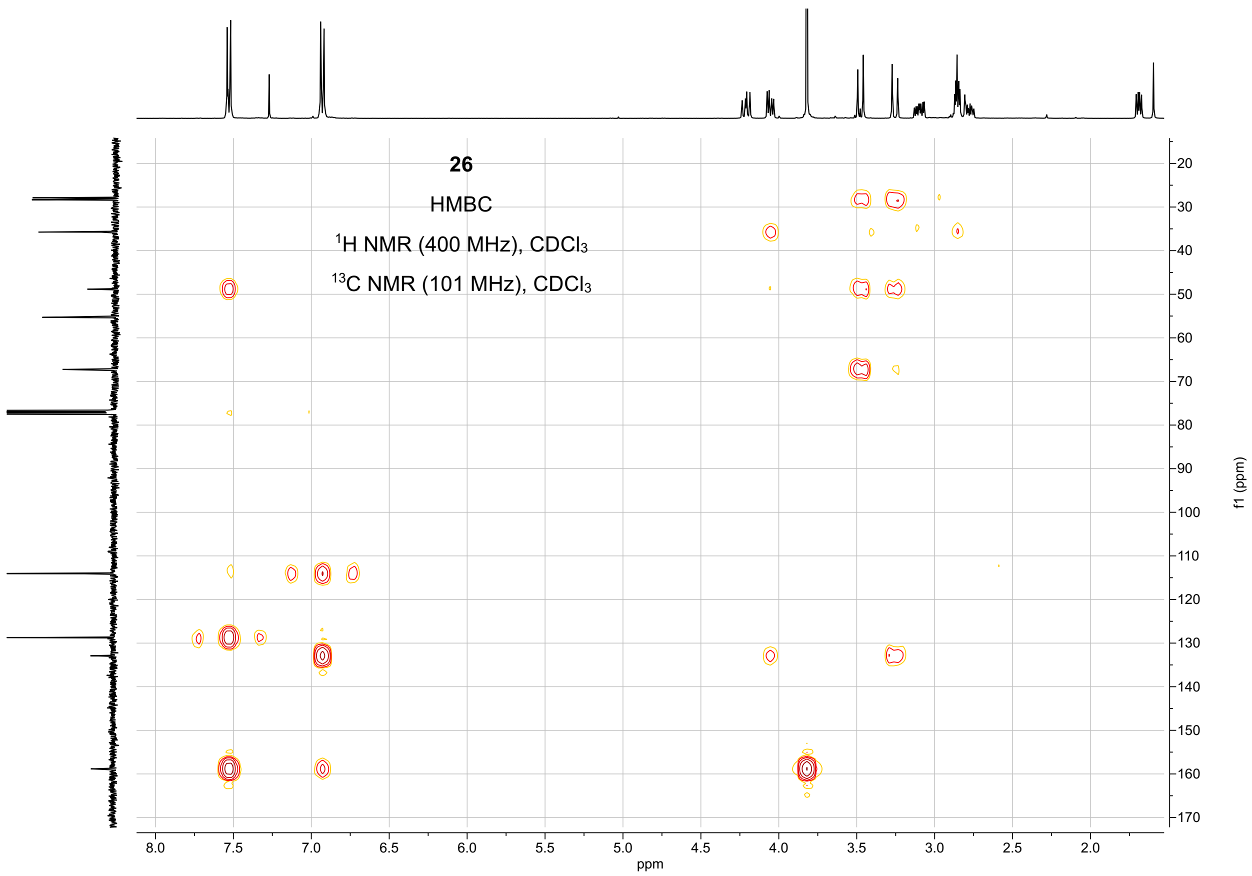
26
HMBC
1H NMR (400 MHz), CDCl3
13C NMR (101 MHz), CDCl3
ppm
f1 (ppm)

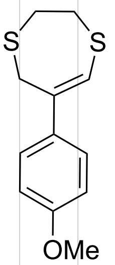

S1
^{1}H NMR (400 MHz), $CDCl_3$

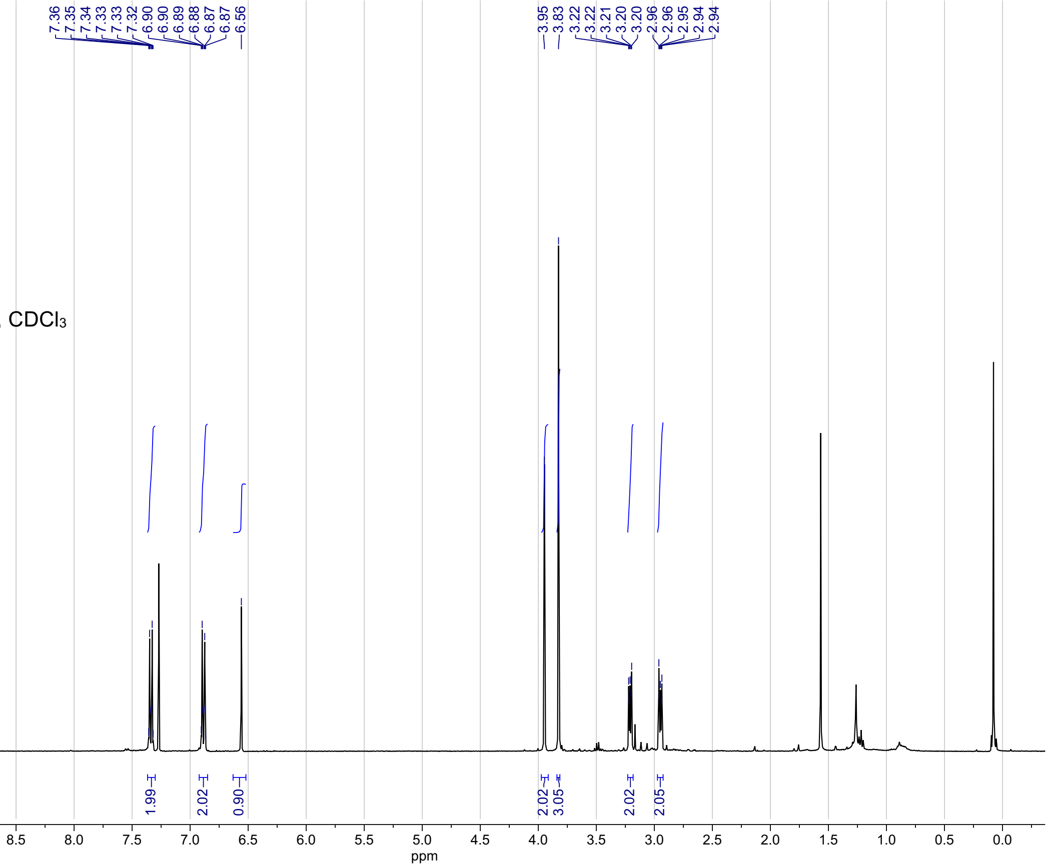

S1
^{13}C NMR (101 MHz), $CDCl_3$

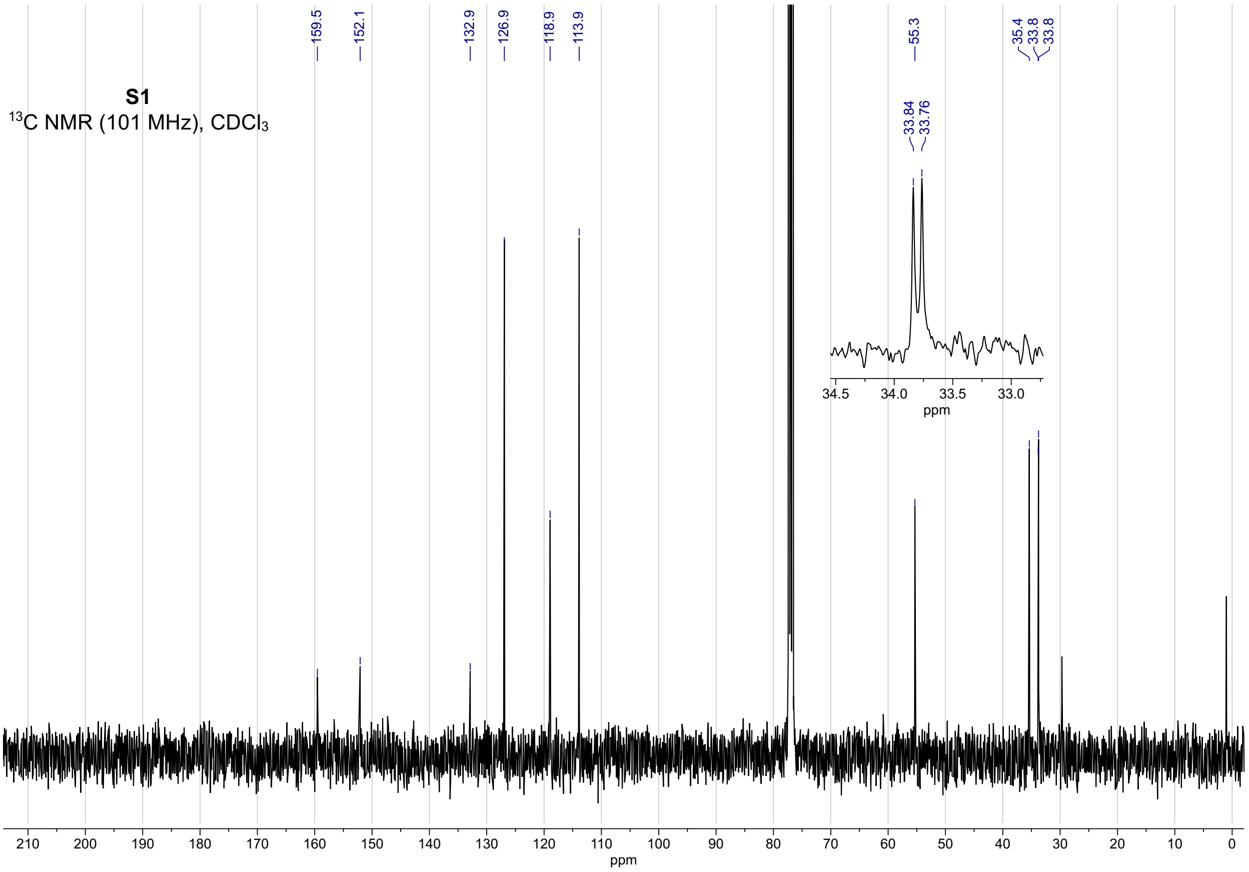

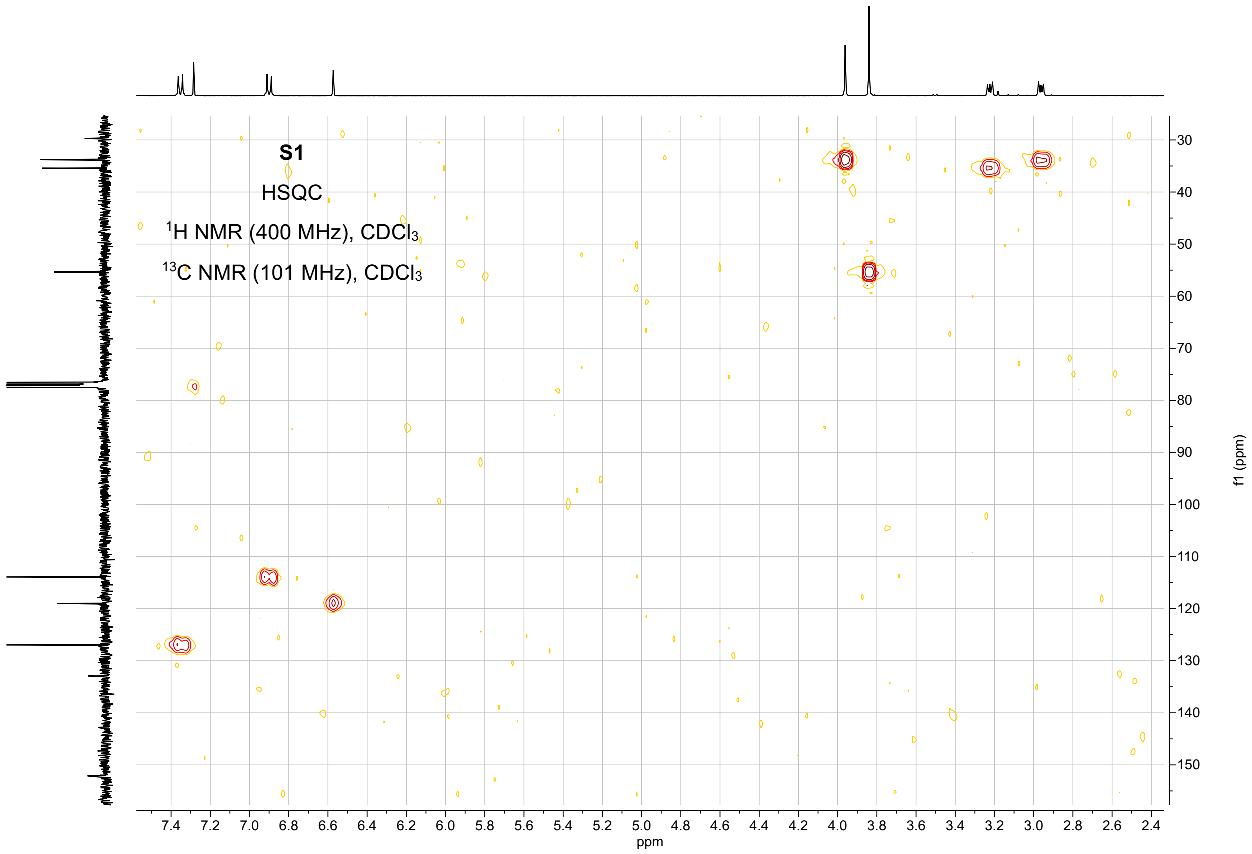
S1
HSQC
¹H NMR (400 MHz), CDCl₃
¹³C NMR (101 MHz), CDCl₃
ppm
f1 (ppm)

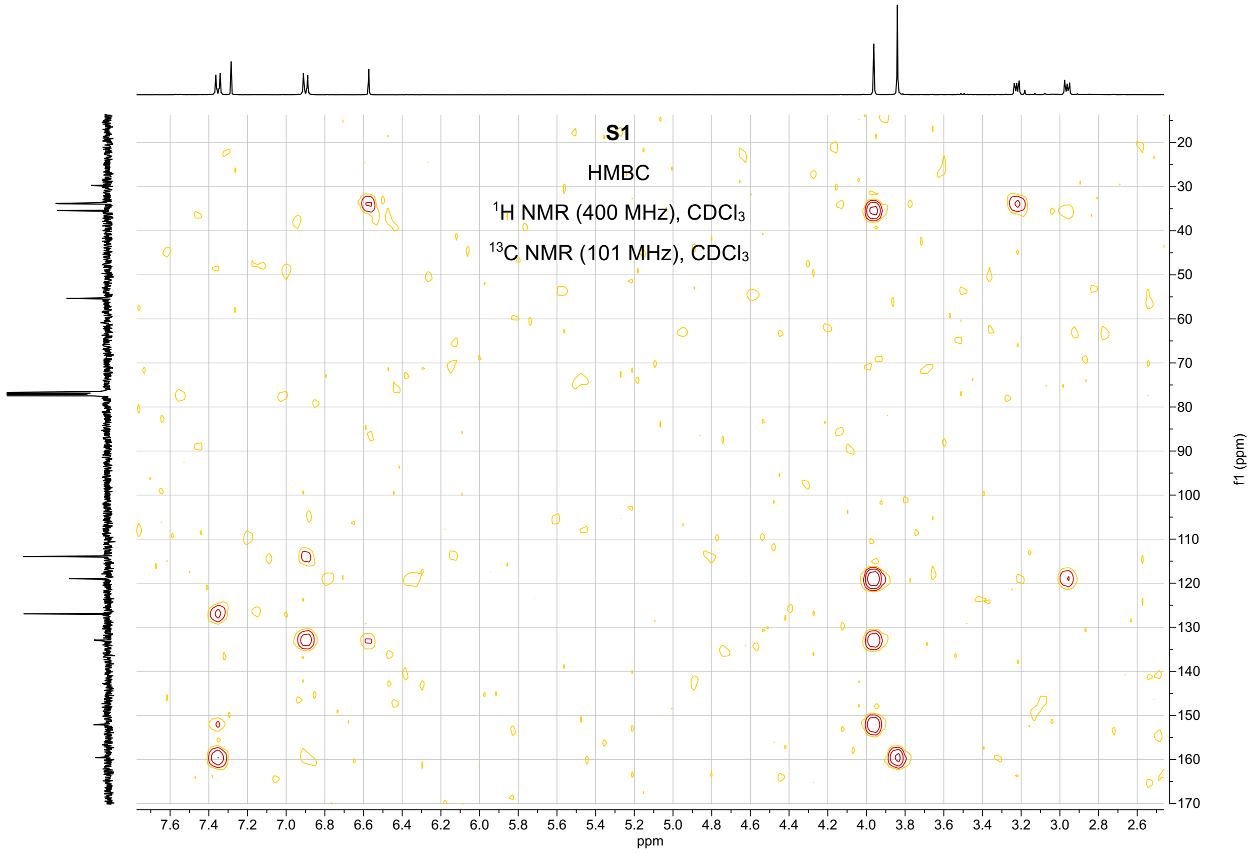
S1
HMBC
¹H NMR (400 MHz), CDCl₃
¹³C NMR (101 MHz), CDCl₃
ppm
f1 (ppm)

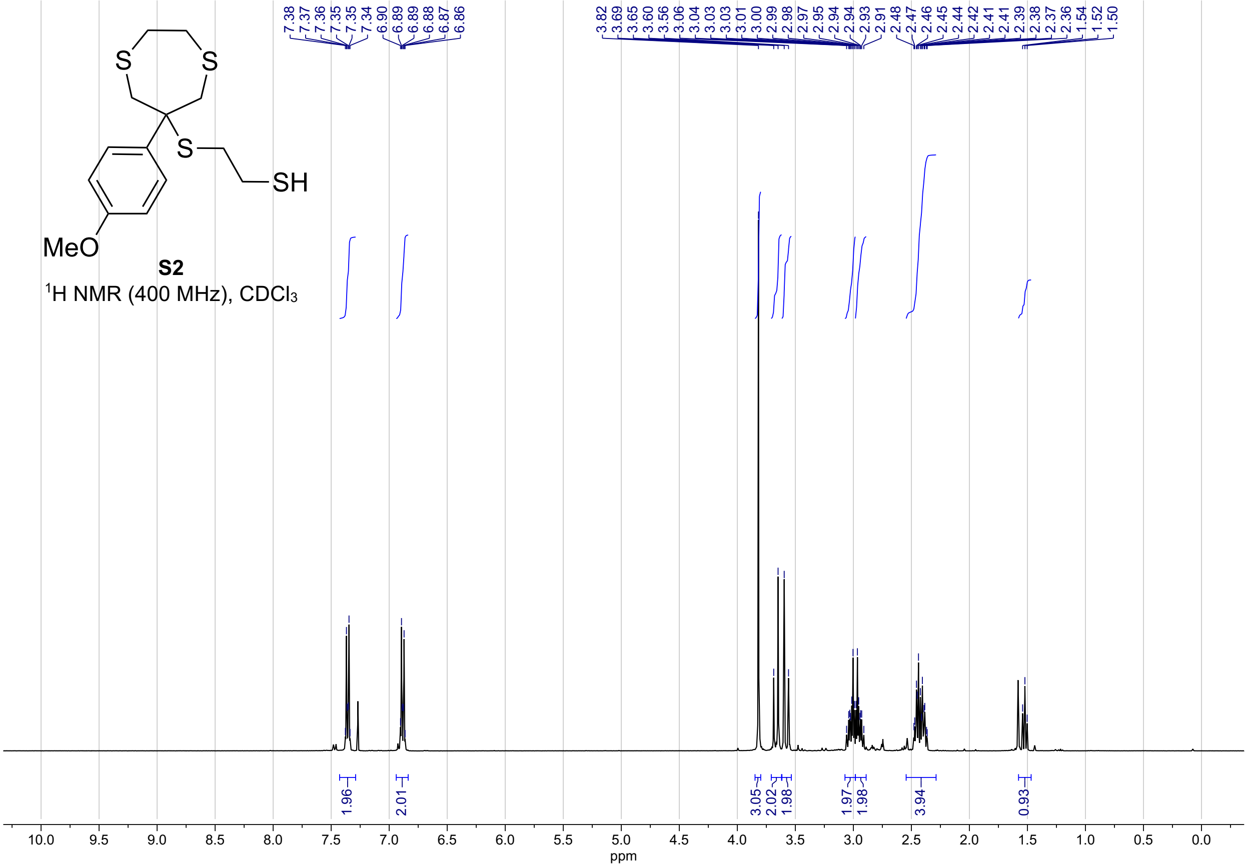

S2
^{1}H NMR (400 MHz), $CDCl_3$

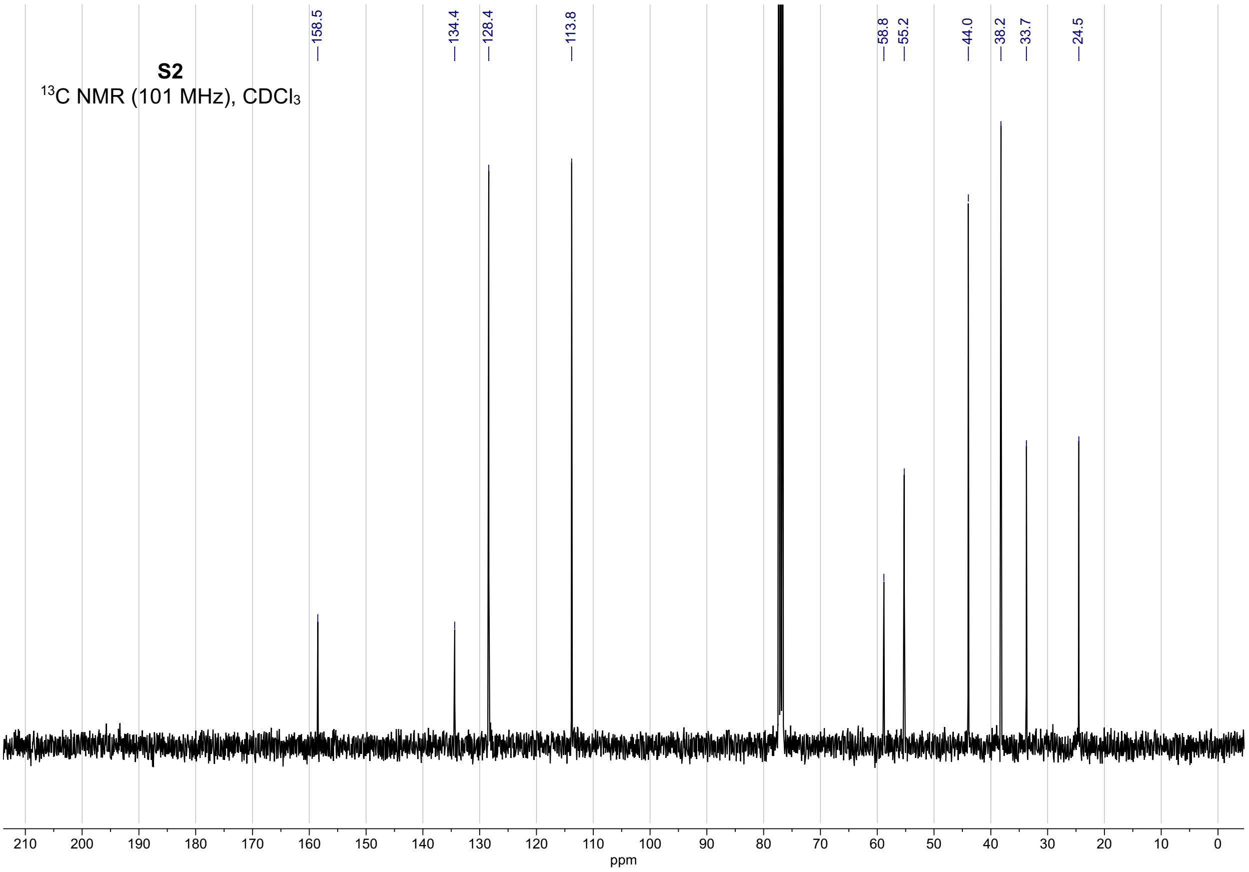

S2
^{13}C NMR (101 MHz), $CDCl_3$

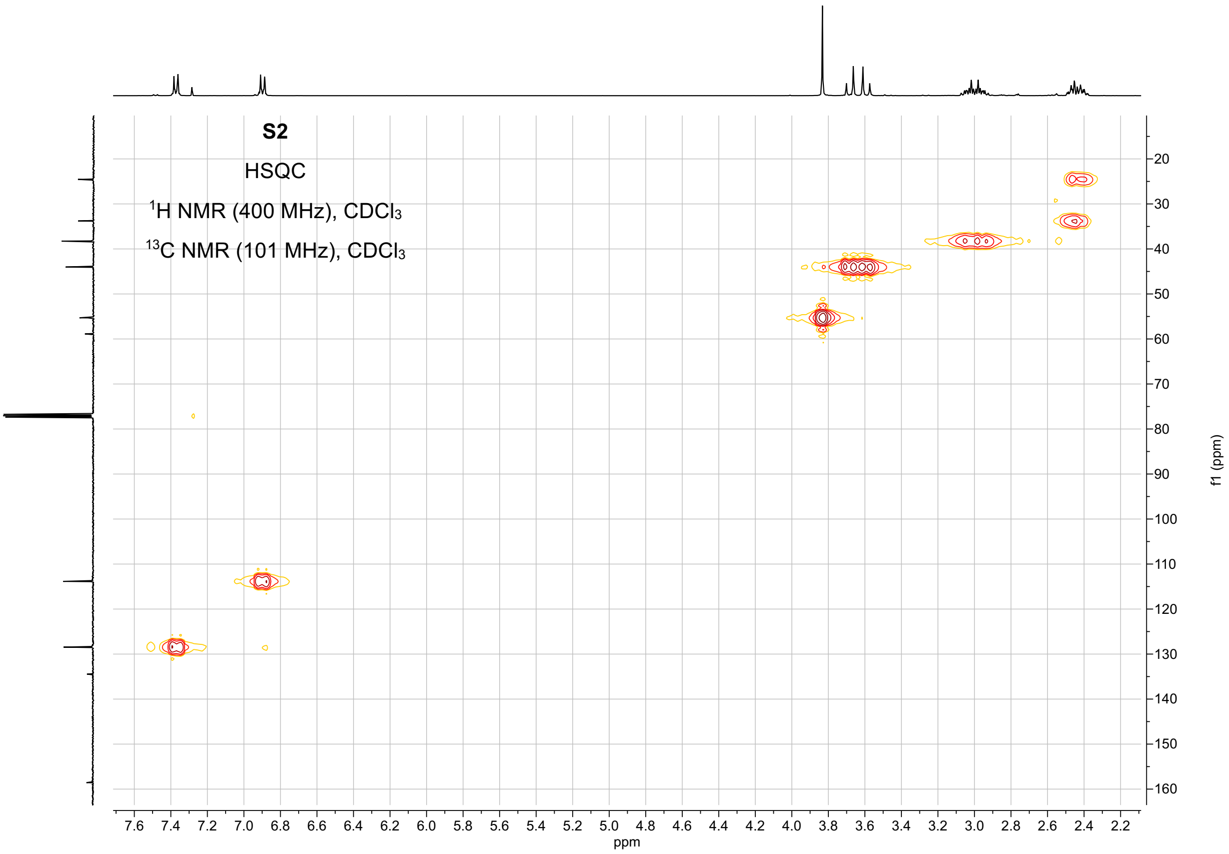
S2
HSQC
^{1}H NMR (400 MHz), $CDCl_3$
^{13}C NMR (101 MHz), $CDCl_3$
ppm
f1 (ppm)

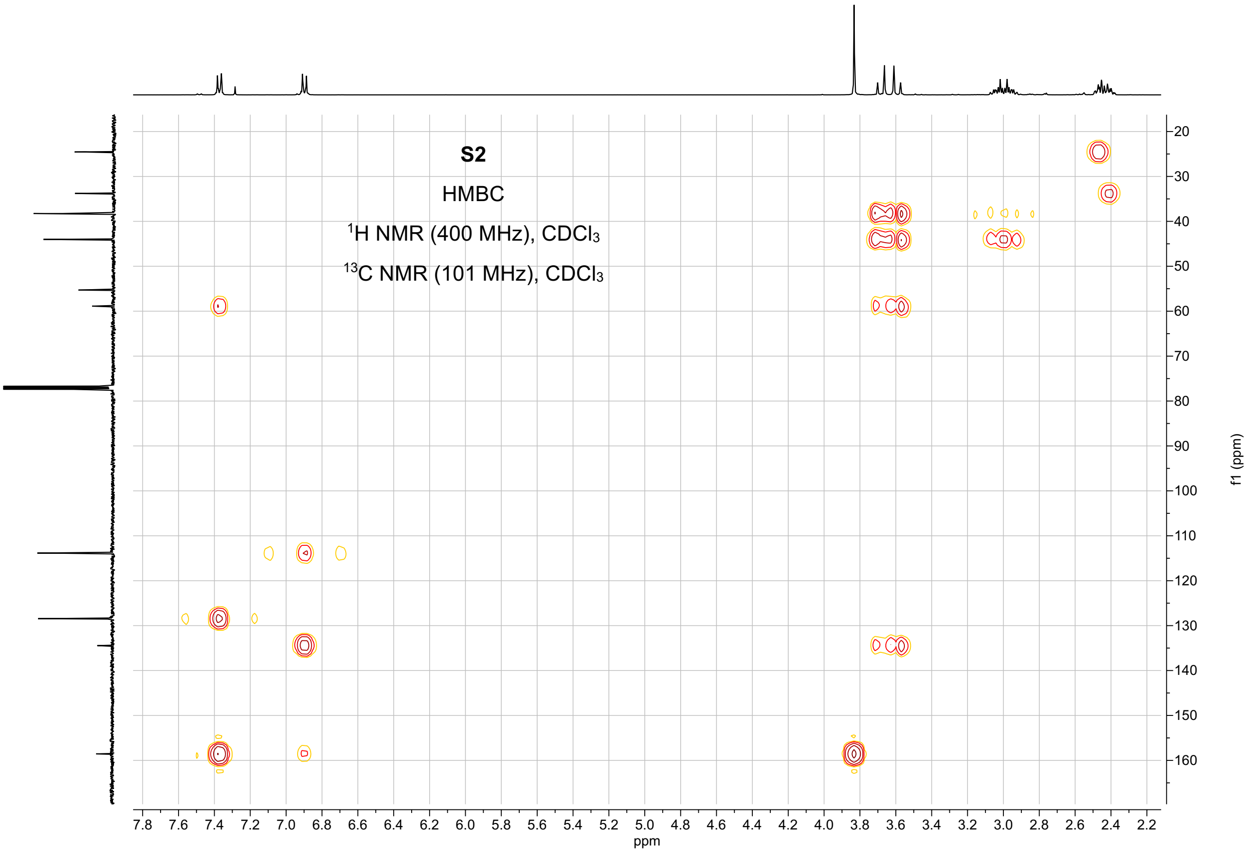
S2
HMBC
^{1}H NMR (400 MHz), $CDCl_3$
^{13}C NMR (101 MHz), $CDCl_3$
ppm
f1 (ppm)

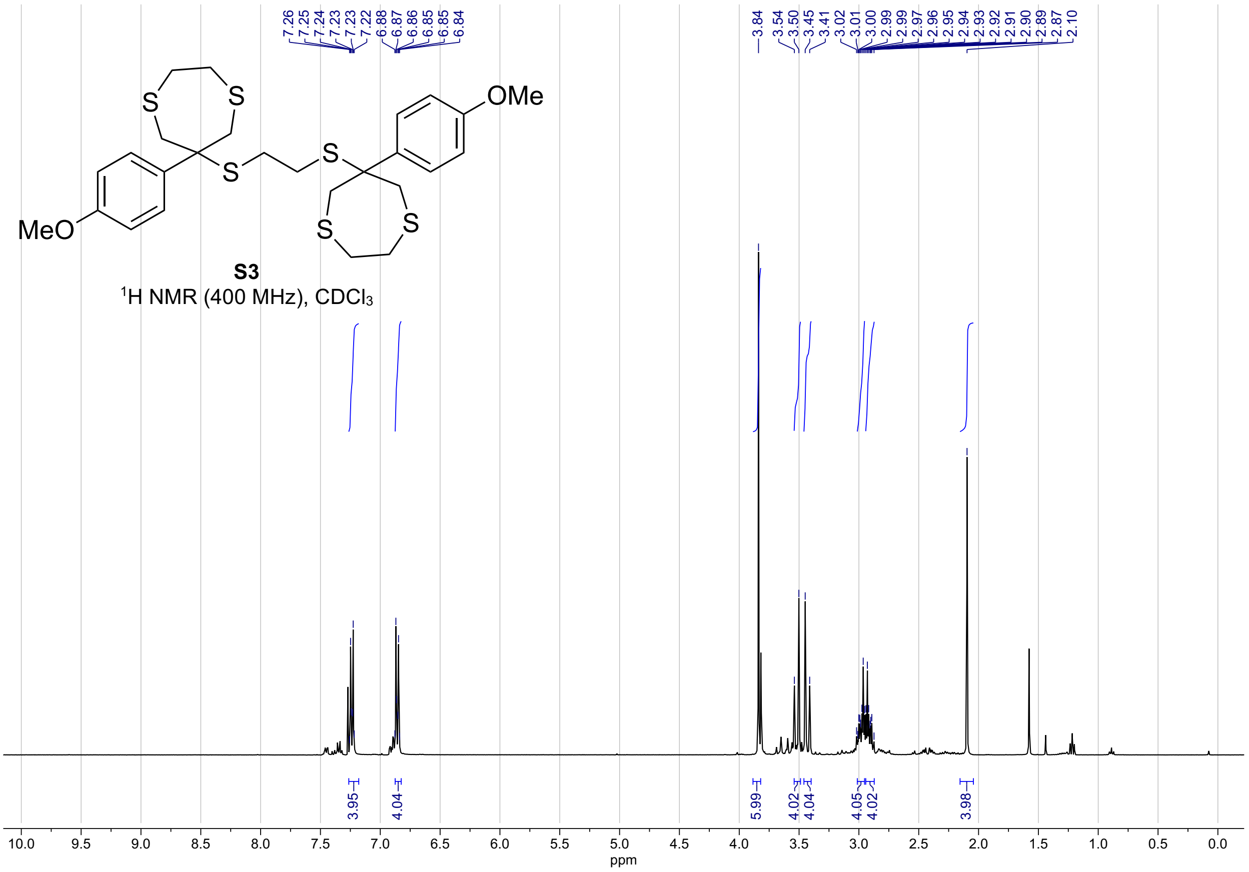
S3
¹H NMR (400 MHz), CDCl3
OMe
MeO
S
7.26
7.25
7.24
7.23
7.23
7.22
6.88
6.87
6.86
6.85
6.85
6.84
3.84
3.54
3.50
3.45
3.41
3.02
3.01
3.00
2.99
2.99
2.97
2.96
2.95
2.94
2.93
2.92
2.91
2.90
2.89
2.87
2.10
3.95
4.04
5.99
4.02
4.04
4.05
4.02
3.98
ppm

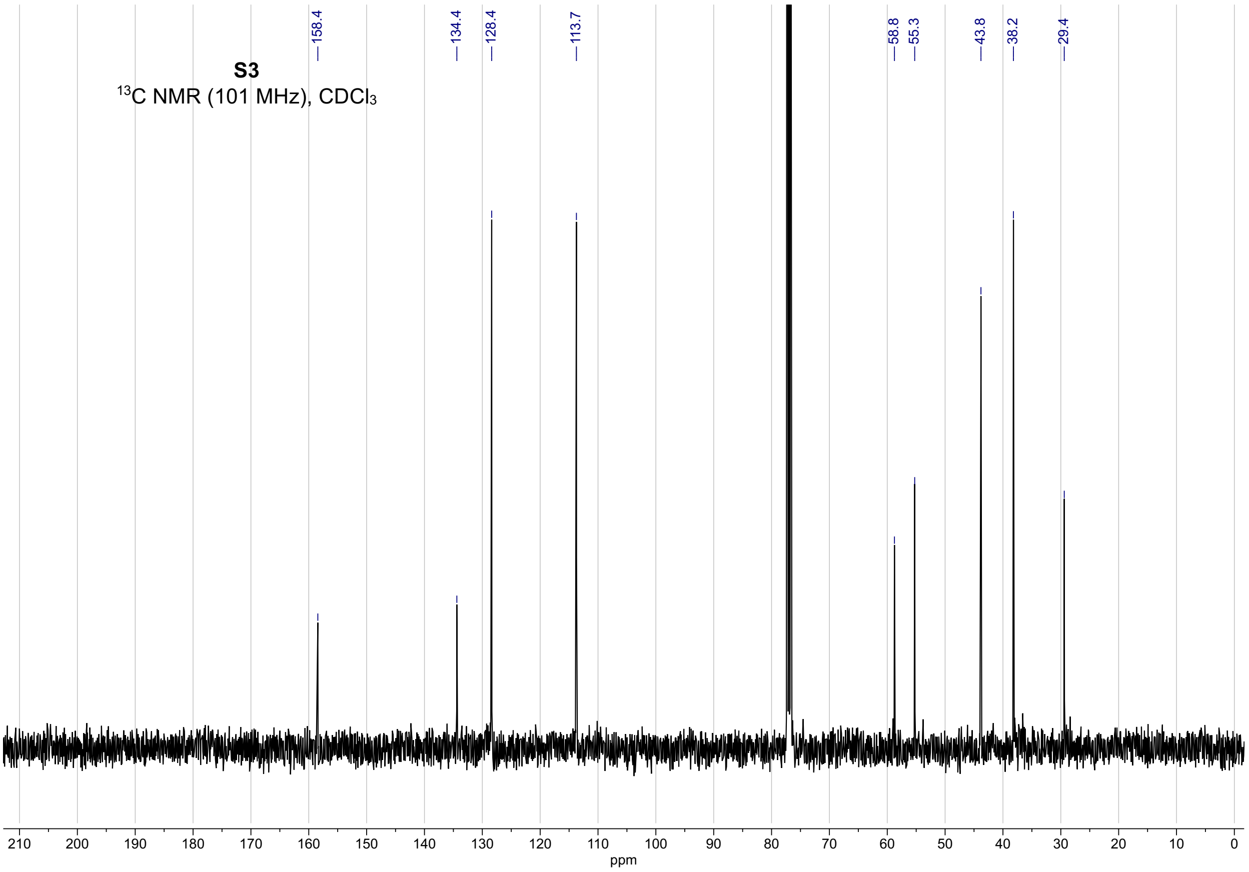
S3
¹³C NMR (101 MHz), CDCl3
158.4
134.4
128.4
113.7
58.8
55.3
43.8
38.2
29.4
ppm

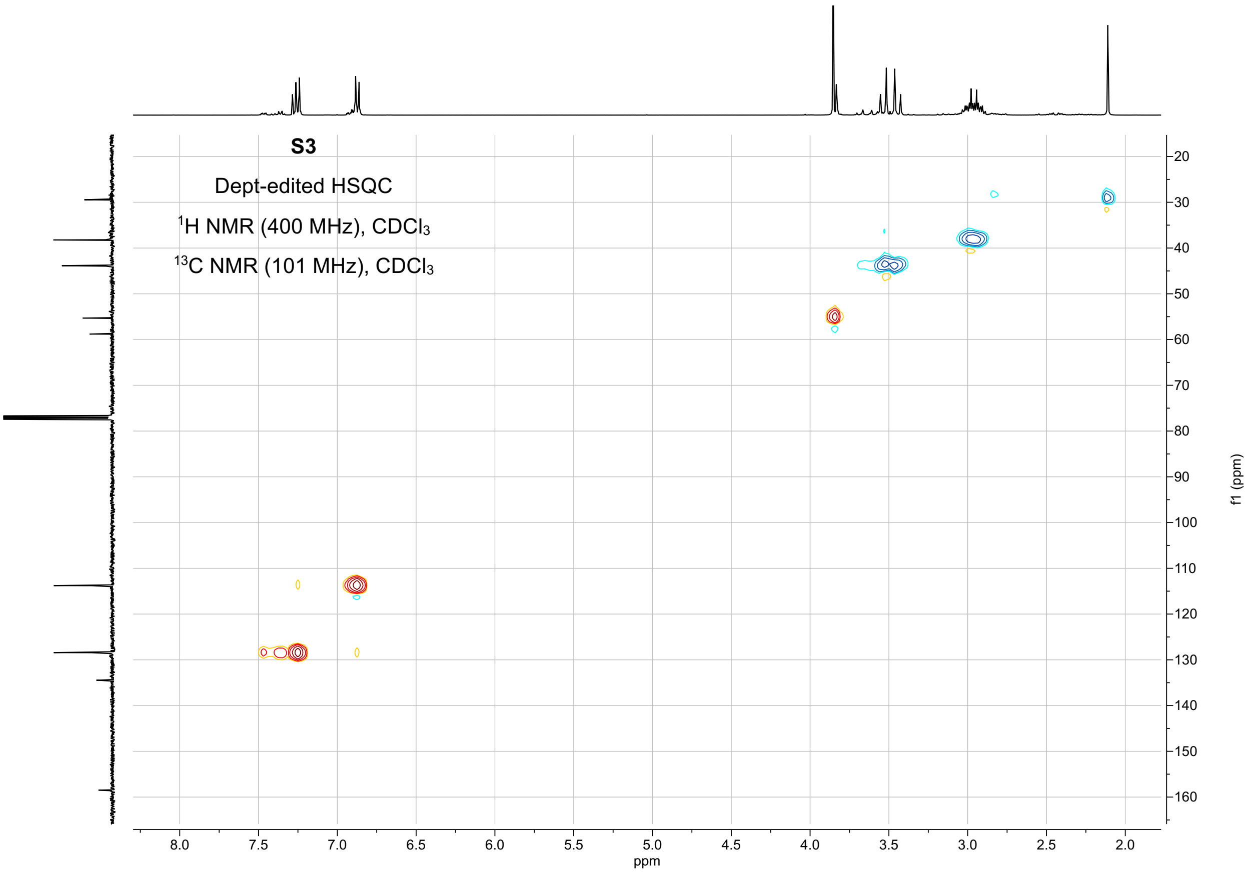
S3
Dept-edited HSQC
^{1}H NMR (400 MHz), $CDCl_3$
^{13}C NMR (101 MHz), $CDCl_3$
ppm
f1 (ppm)

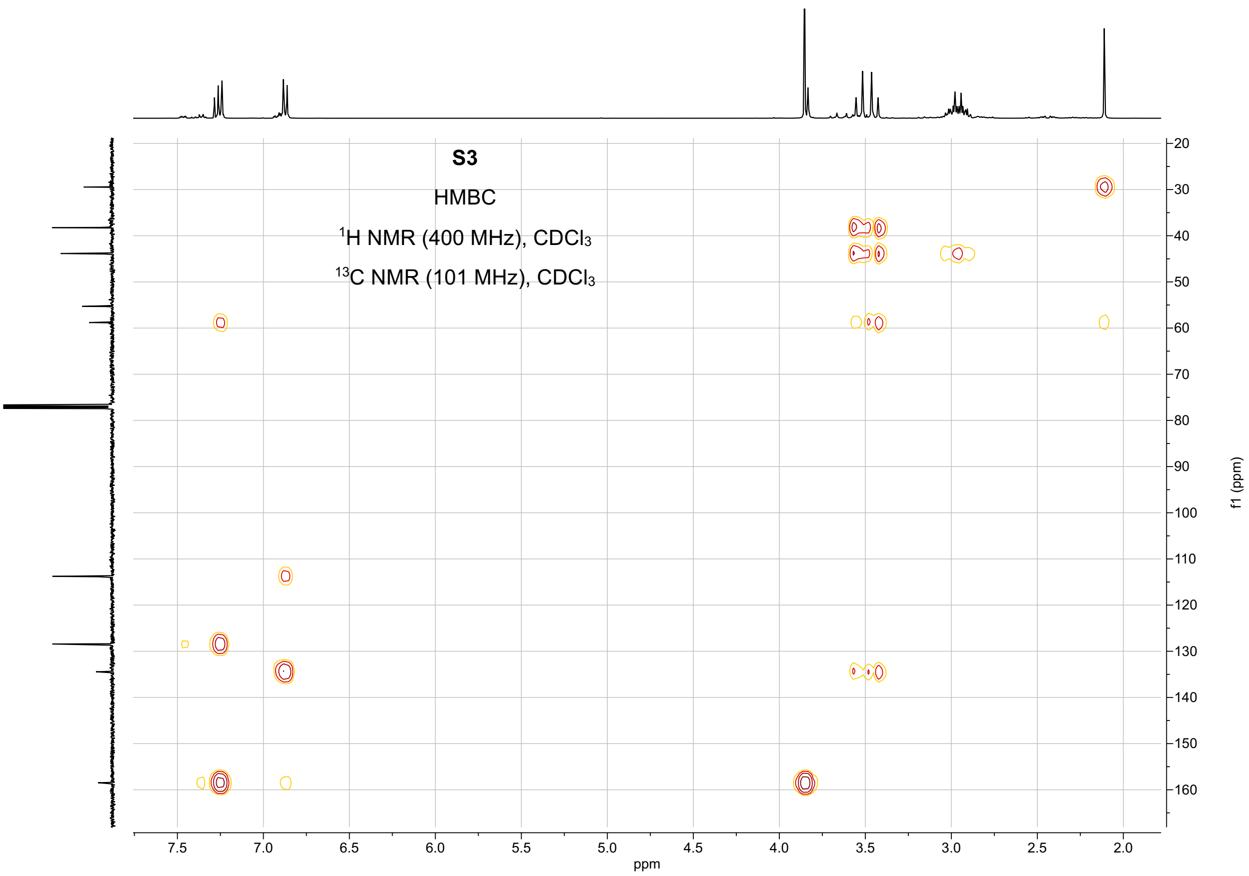
S3
HMBC
^{1}H NMR (400 MHz), $CDCl_3$
^{13}C NMR (101 MHz), $CDCl_3$
ppm
f1 (ppm)

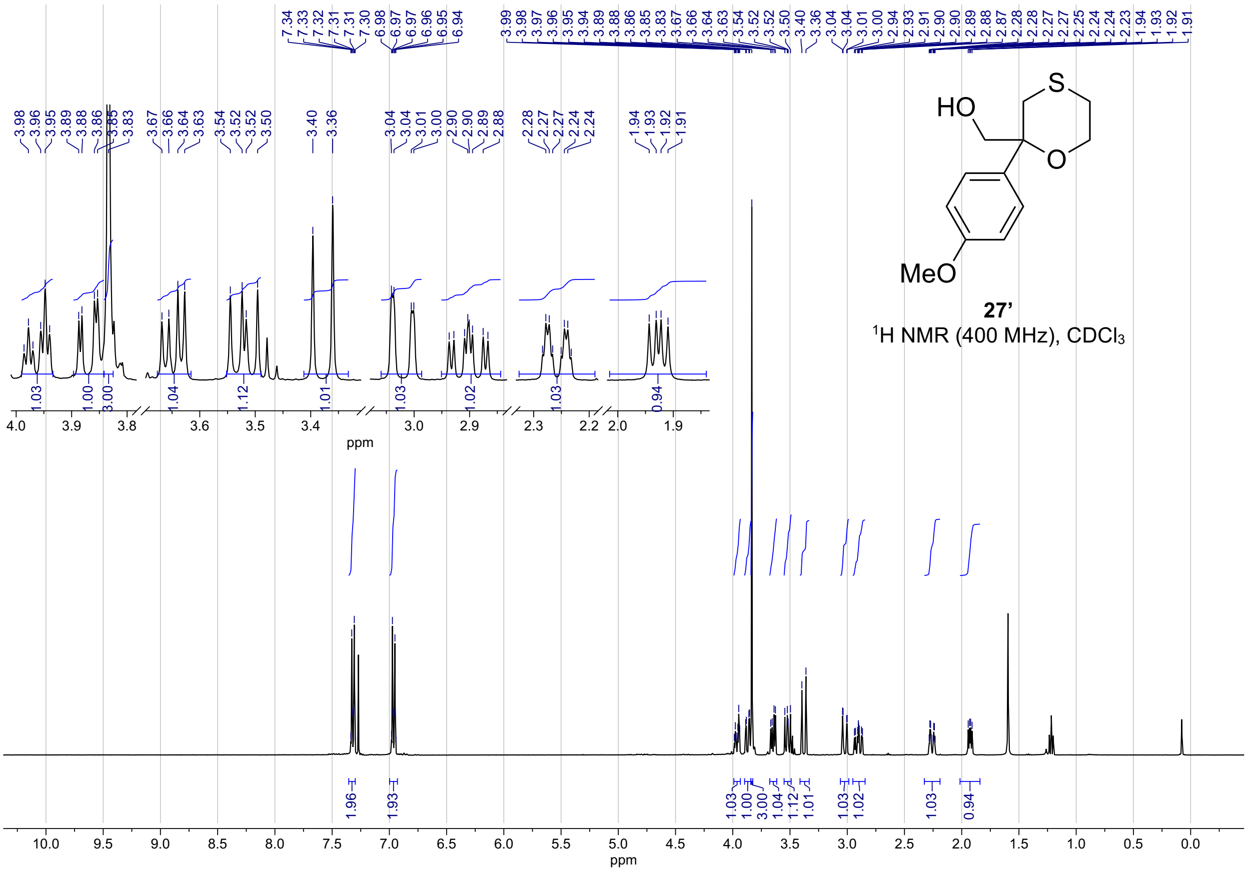
HO
S
O
MeO
27'
1H NMR (400 MHz), CDCl3
ppm

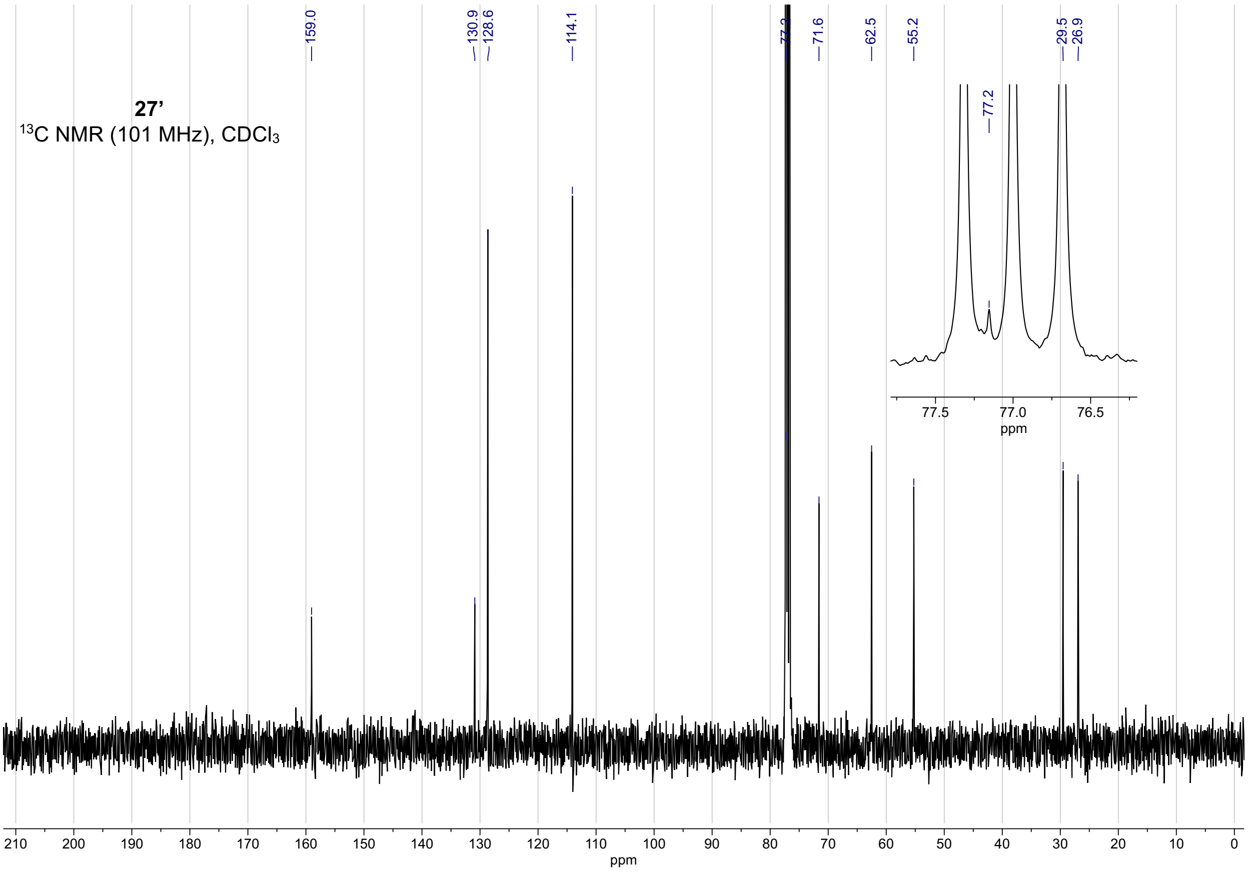
27'
13C NMR (101 MHz), CDCl3
159.0
130.9
128.6
114.1
71.6
62.5
55.2
29.5
26.9
77.2
ppm

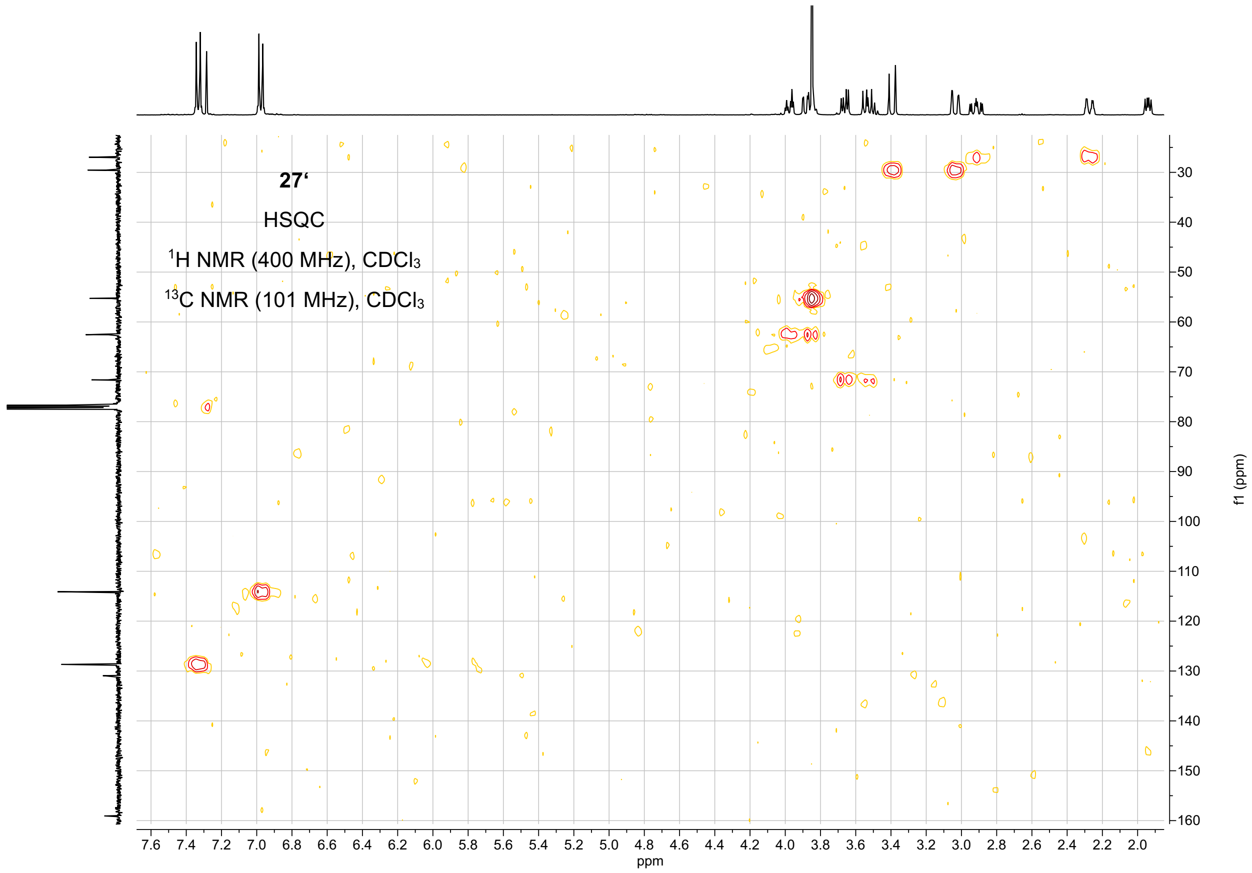
27'
HSQC
1H NMR (400 MHz), CDCl3
13C NMR (101 MHz), CDCl3
ppm
f1 (ppm)

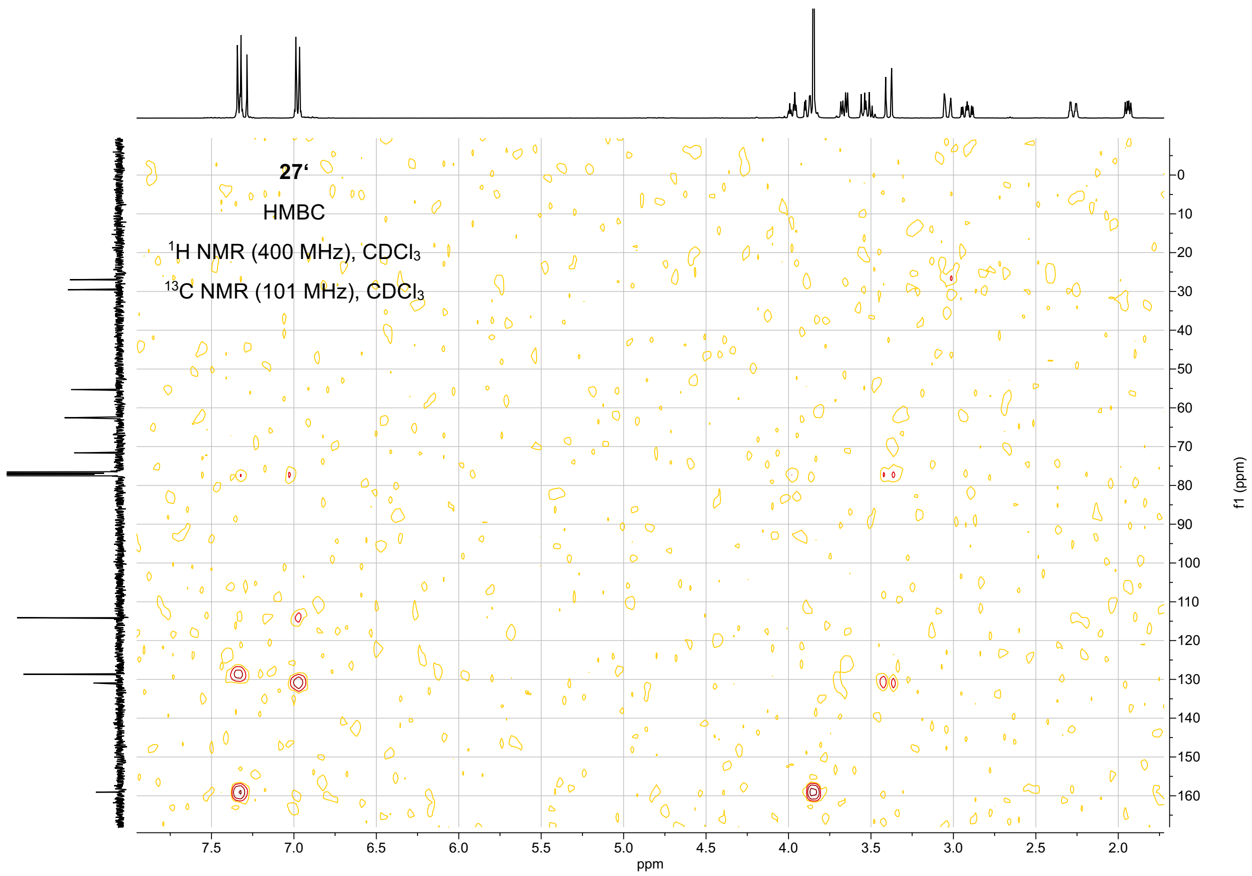
27'
HMBC
1H NMR (400 MHz), CDCl3
13C NMR (101 MHz), CDCl3
ppm
f1 (ppm)

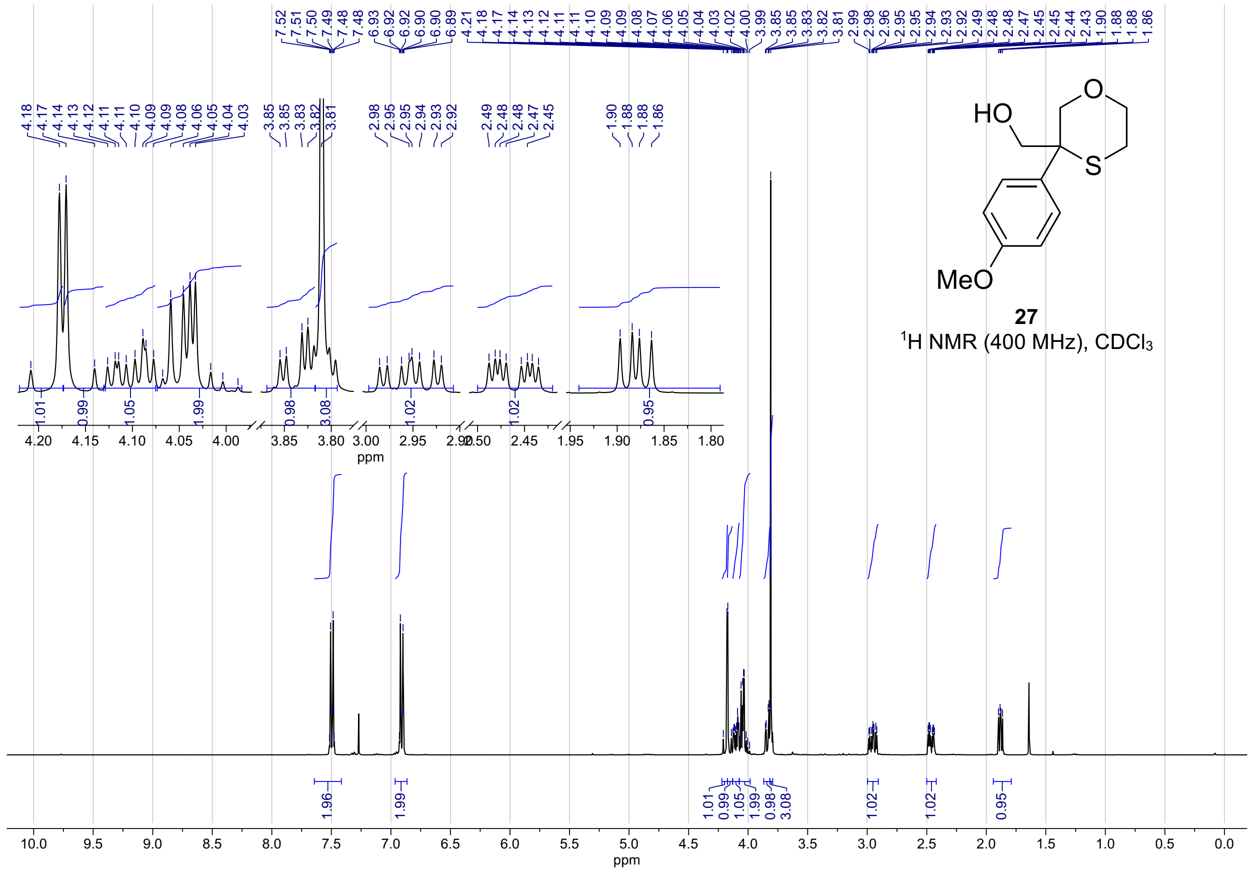

27
^{1}H NMR (400 MHz), $CDCl_3$

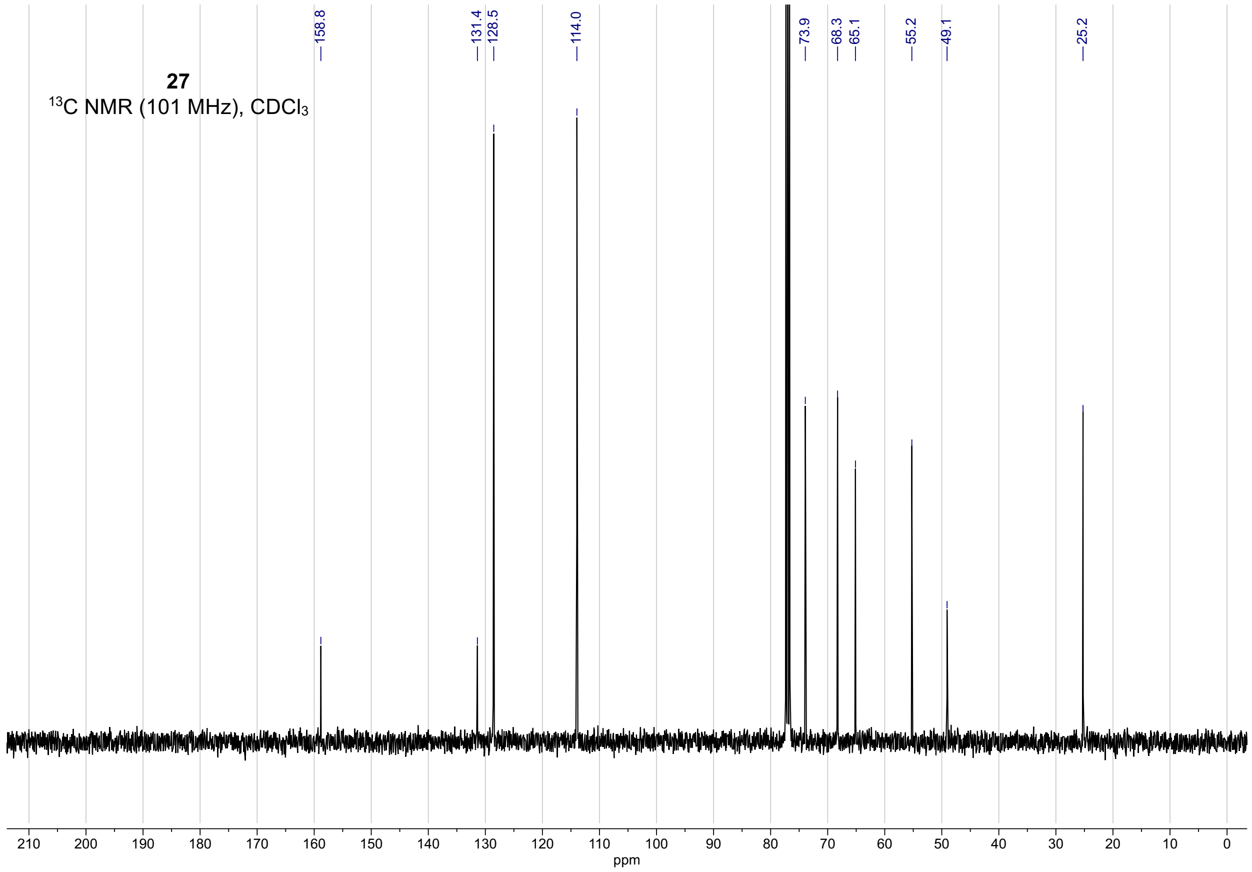

27
^{13}C NMR (101 MHz), $CDCl_3$

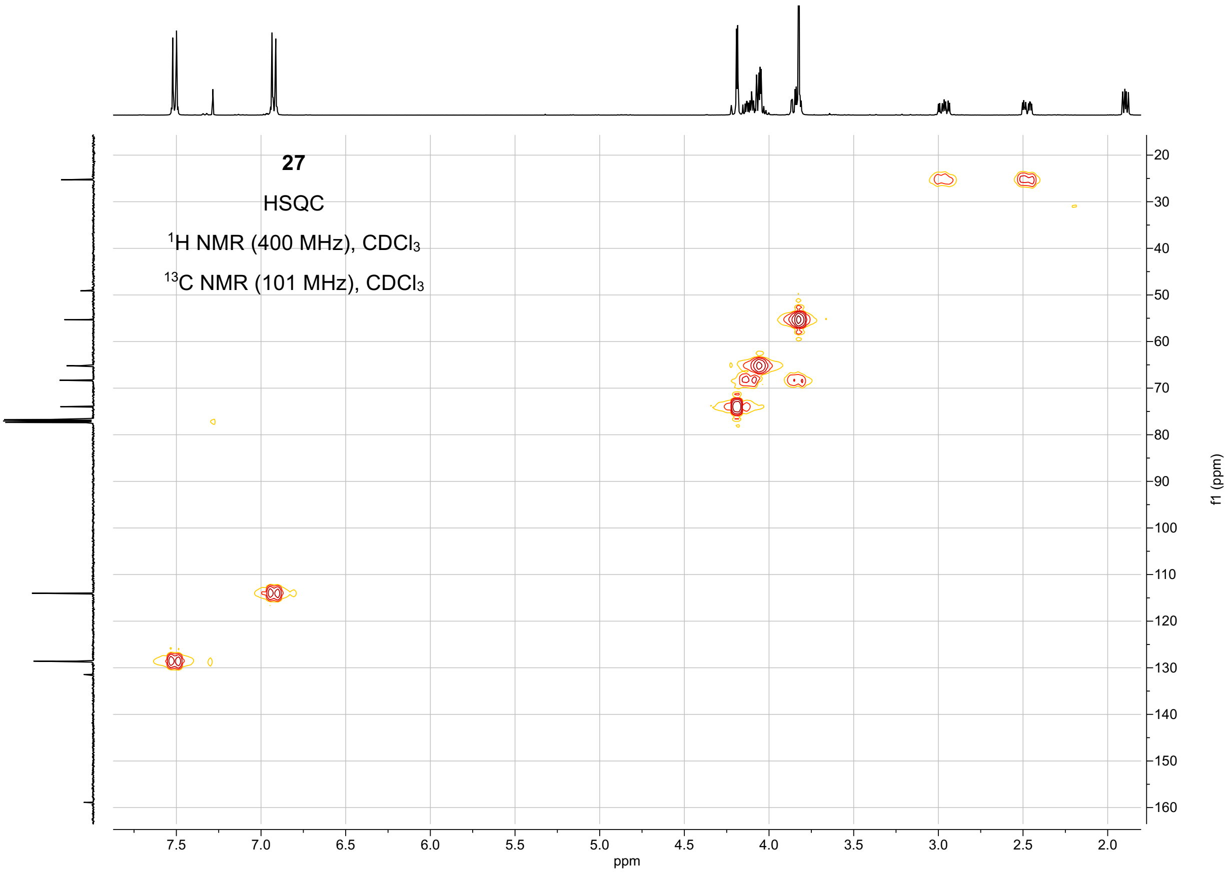
27
HSQC
1H NMR (400 MHz), CDCl3
13C NMR (101 MHz), CDCl3
ppm
f1 (ppm)

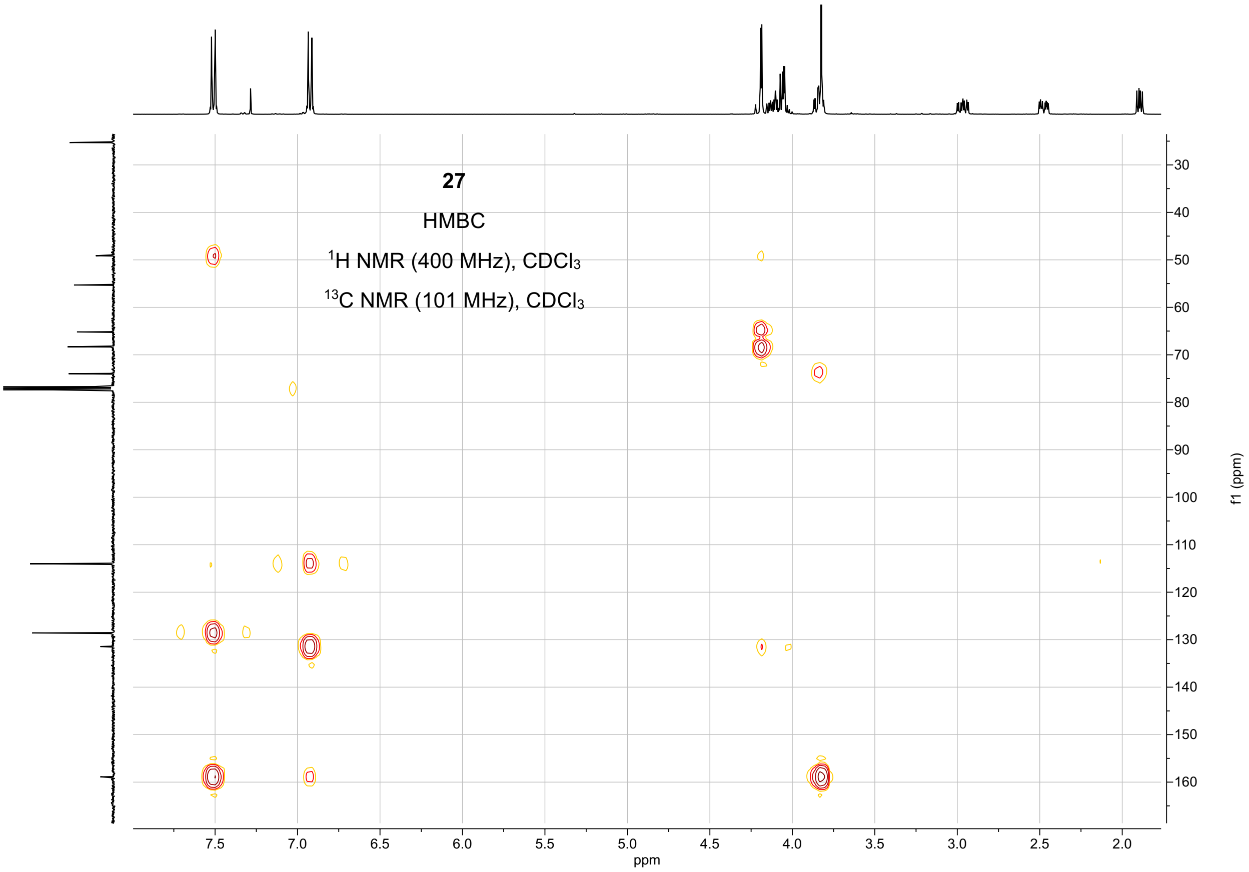
27
HMBC
1H NMR (400 MHz), CDCl3
13C NMR (101 MHz), CDCl3
ppm
f1 (ppm)

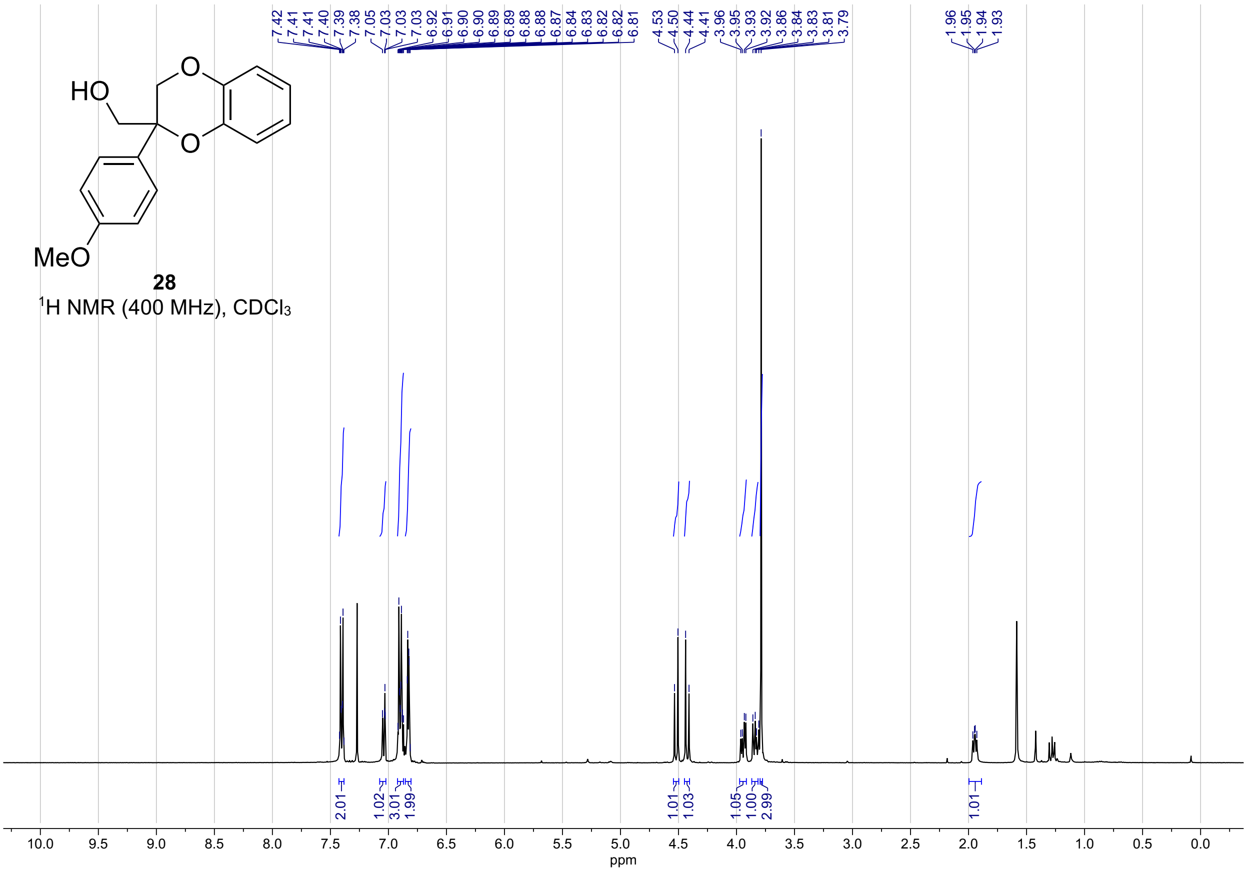

7.42 7.41 7.40 7.39 7.38 7.05 7.03 7.03 7.03 6.92 6.91 6.90 6.90 6.89 6.89 6.88 6.88 6.87 6.84 6.83 6.83 6.82 6.82 6.81
4.53 4.50 4.44 4.41 3.96 3.95 3.93 3.92 3.86 3.84 3.83 3.81 3.79
1.96 1.95 1.94 1.93
HO
O
O
MeO
28
1H NMR (400 MHz), CDCl3
2.01 1.02 3.01 1.99 1.01 1.03 1.05 1.00 2.99 1.01
ppm

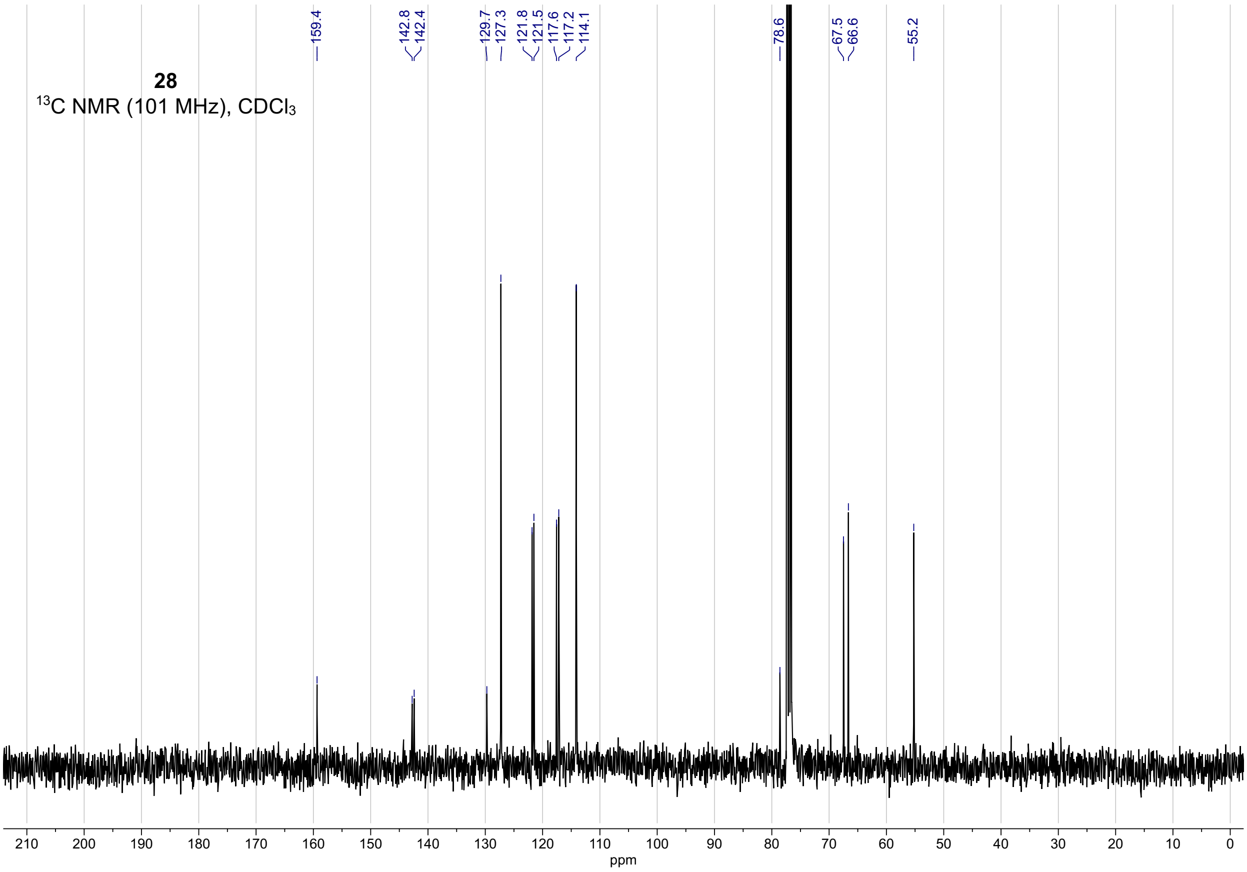

159.4 142.8 142.4 129.7 127.3 121.8 121.5 117.6 117.2 114.1 78.6 67.5 66.6 55.2
28
13C NMR (101 MHz), CDCl3
ppm

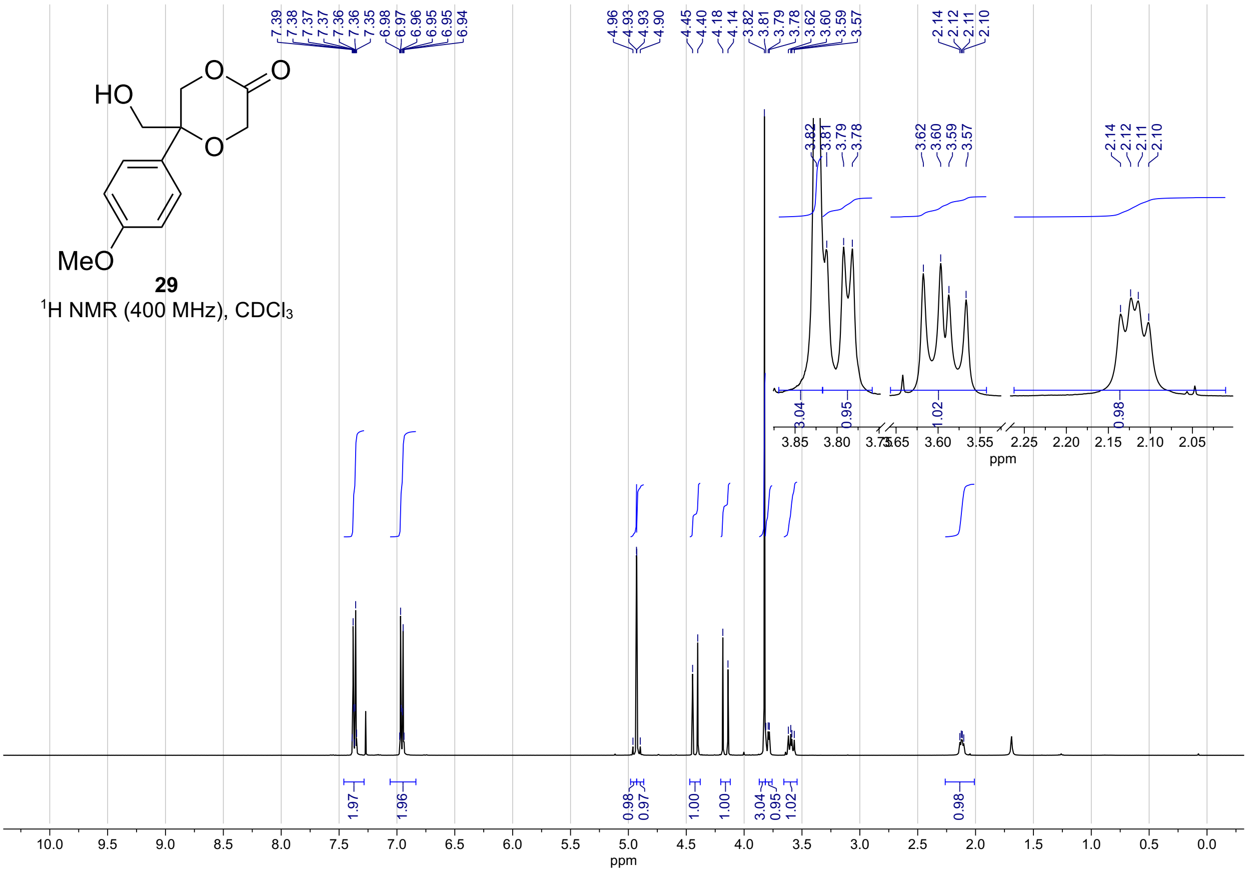
HO
O
O
O
MeO
29
^{1}H NMR (400 MHz), CDCl3
ppm

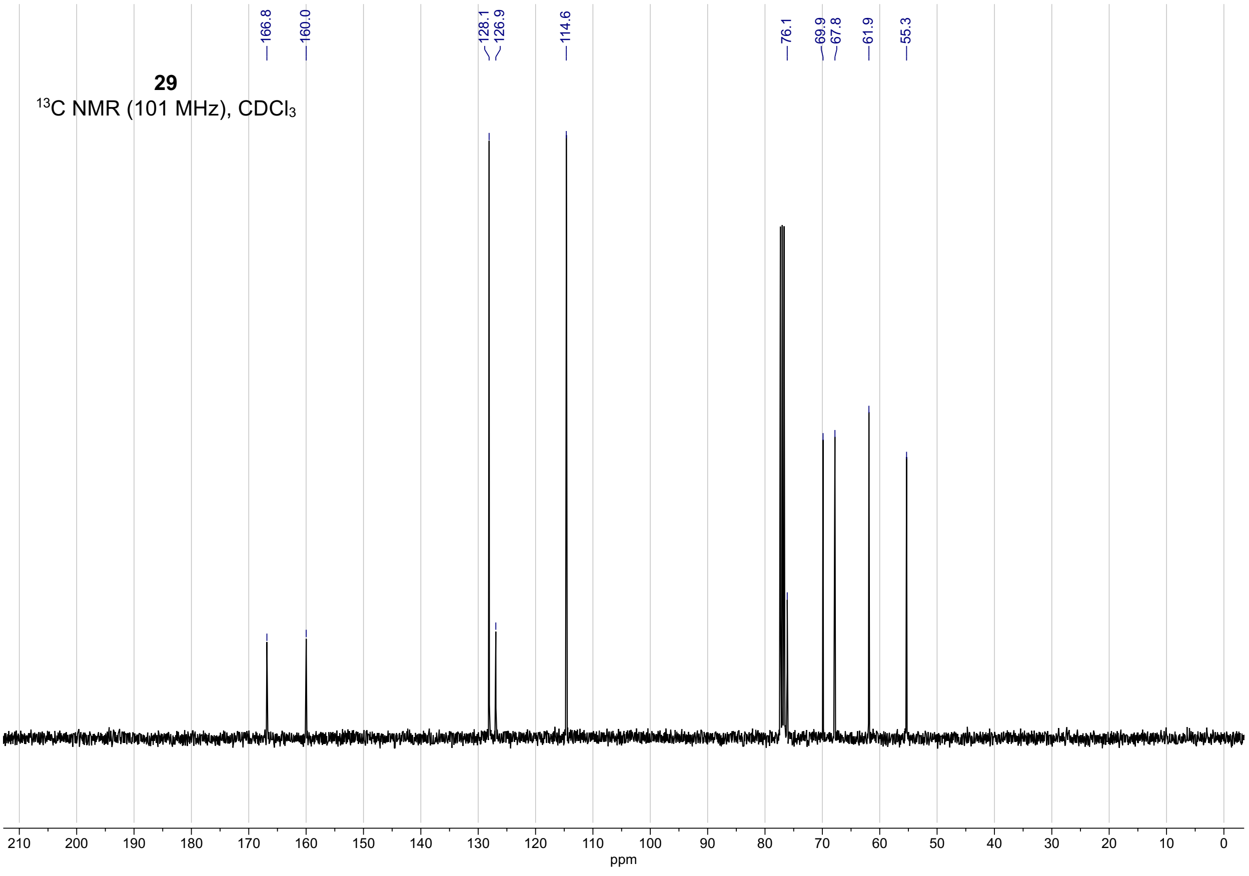
166.8
160.0
128.1
126.9
114.6
76.1
69.9
67.8
61.9
55.3
29
^{13}C NMR (101 MHz), CDCl3
ppm

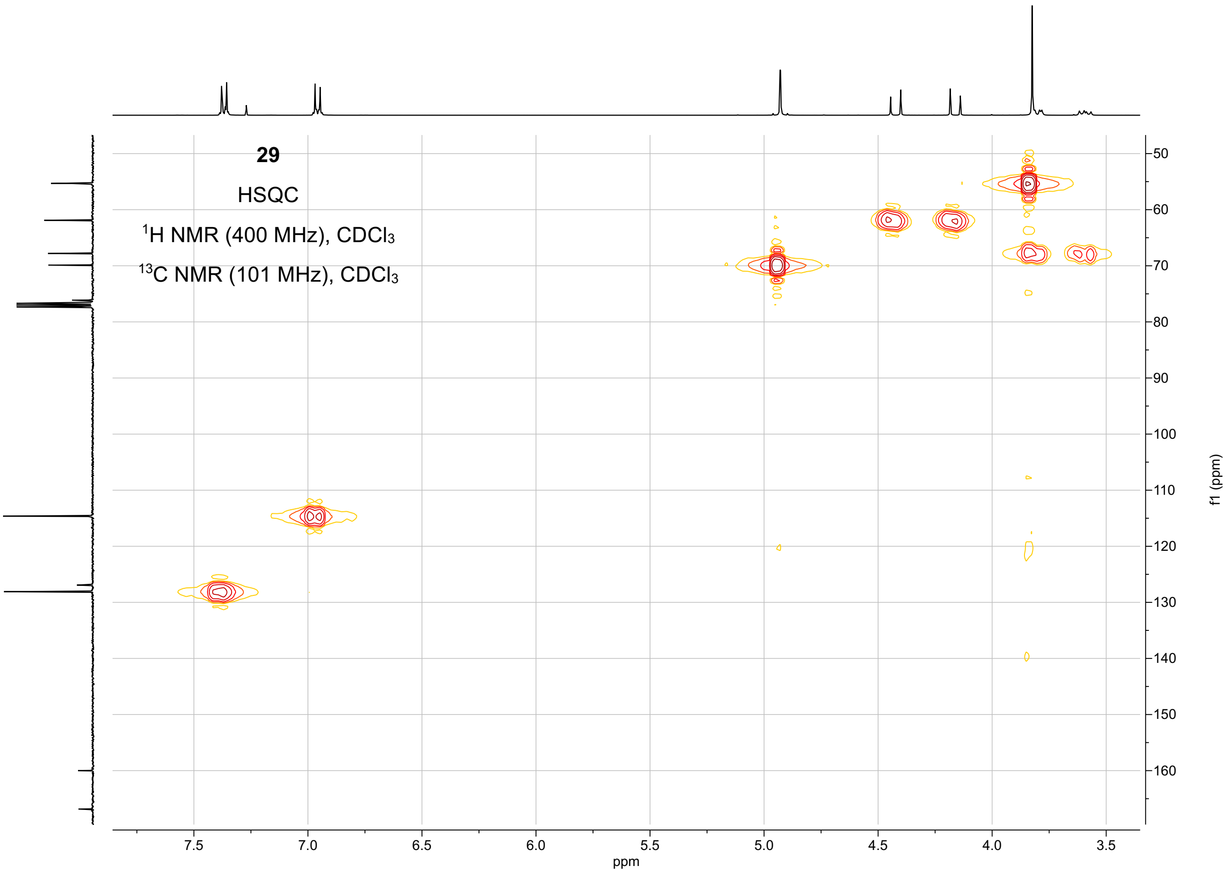
29
HSQC
^{1}H NMR (400 MHz), CDCl3
^{13}C NMR (101 MHz), CDCl3
ppm
f1 (ppm)
7.5 7.0 6.5 6.0 5.5 5.0 4.5 4.0 3.5
50 60 70 80 90 100 110 120 130 140 150 160

S9 + S10
^{1}H NMR (400 MHz), $CDCl_3$

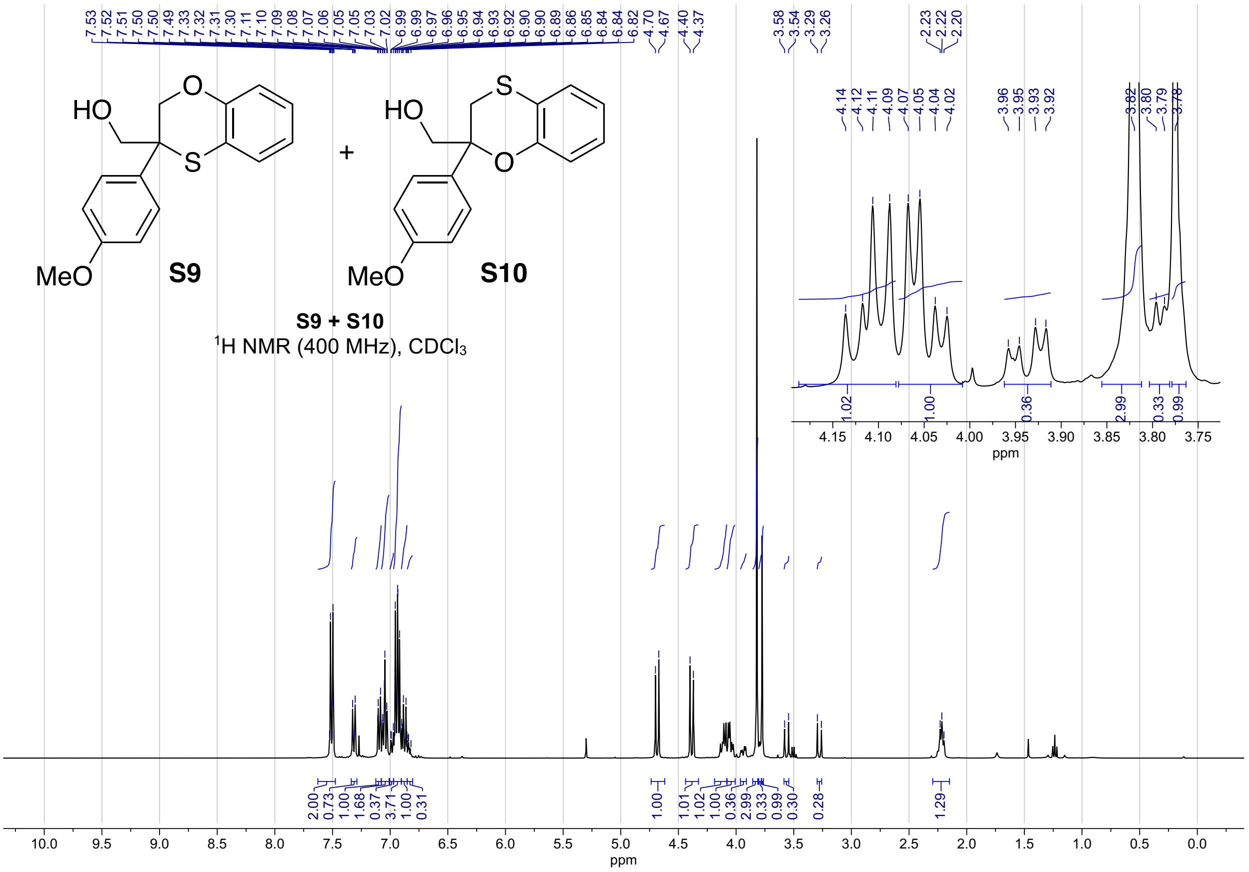

S9 + S10
^{13}C NMR (101 MHz), $CDCl_3$

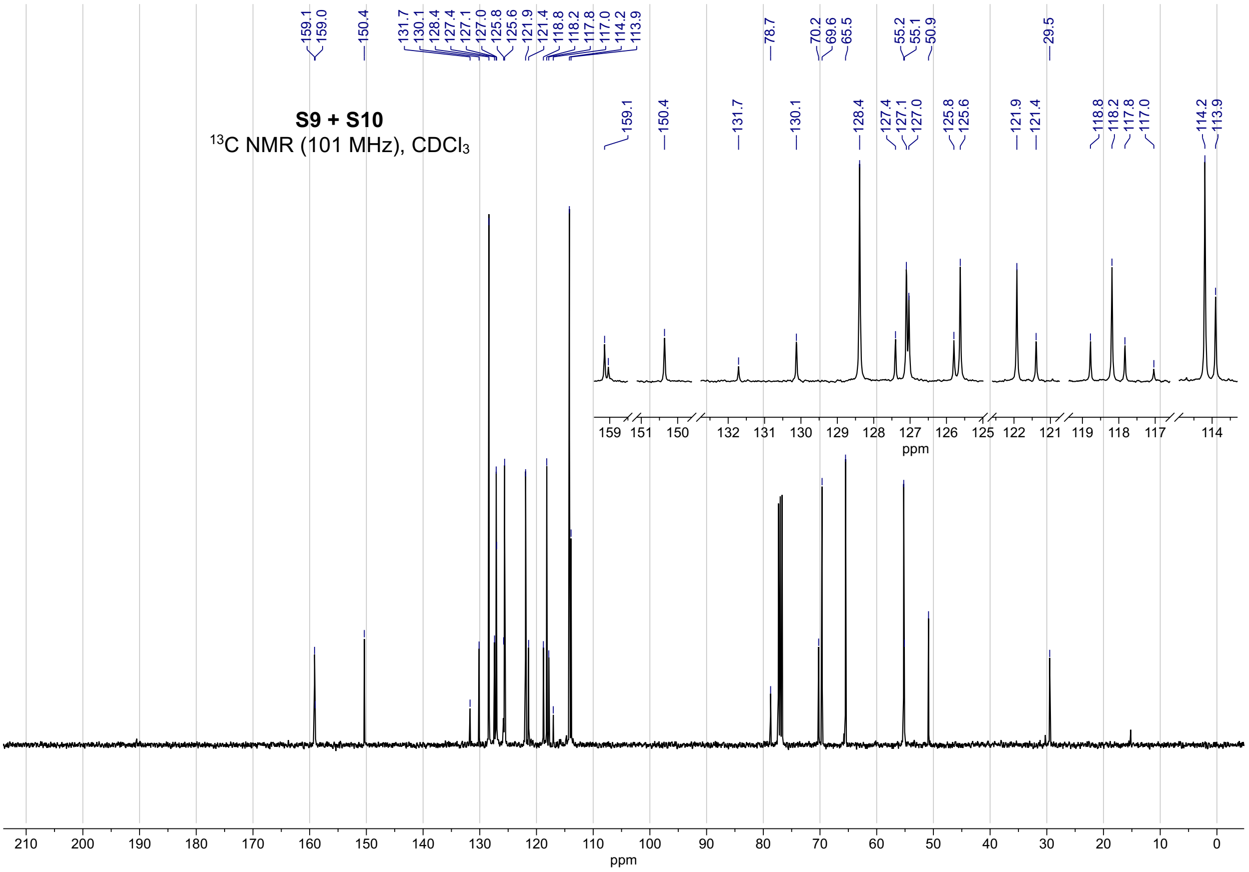

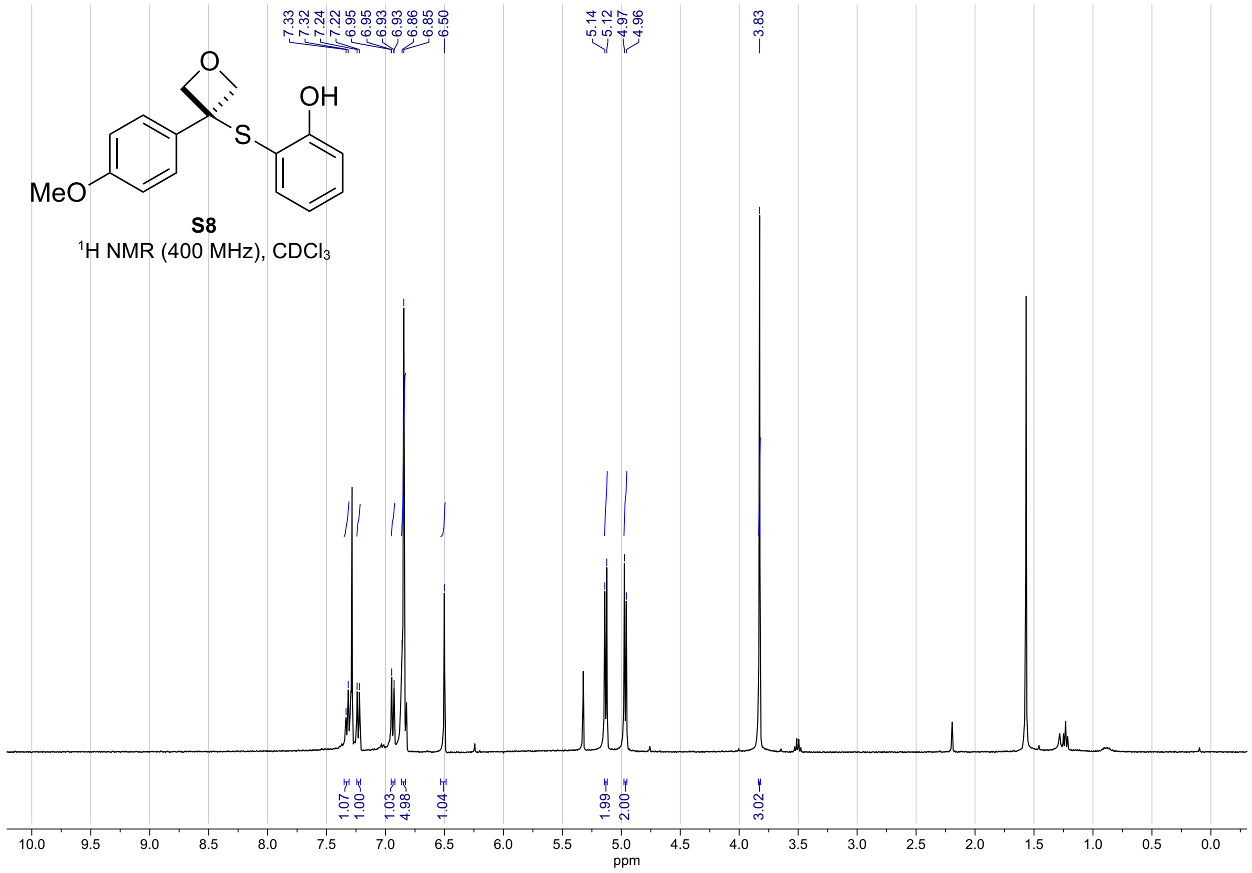

S8
^{1}H NMR (400 MHz), $CDCl_3$

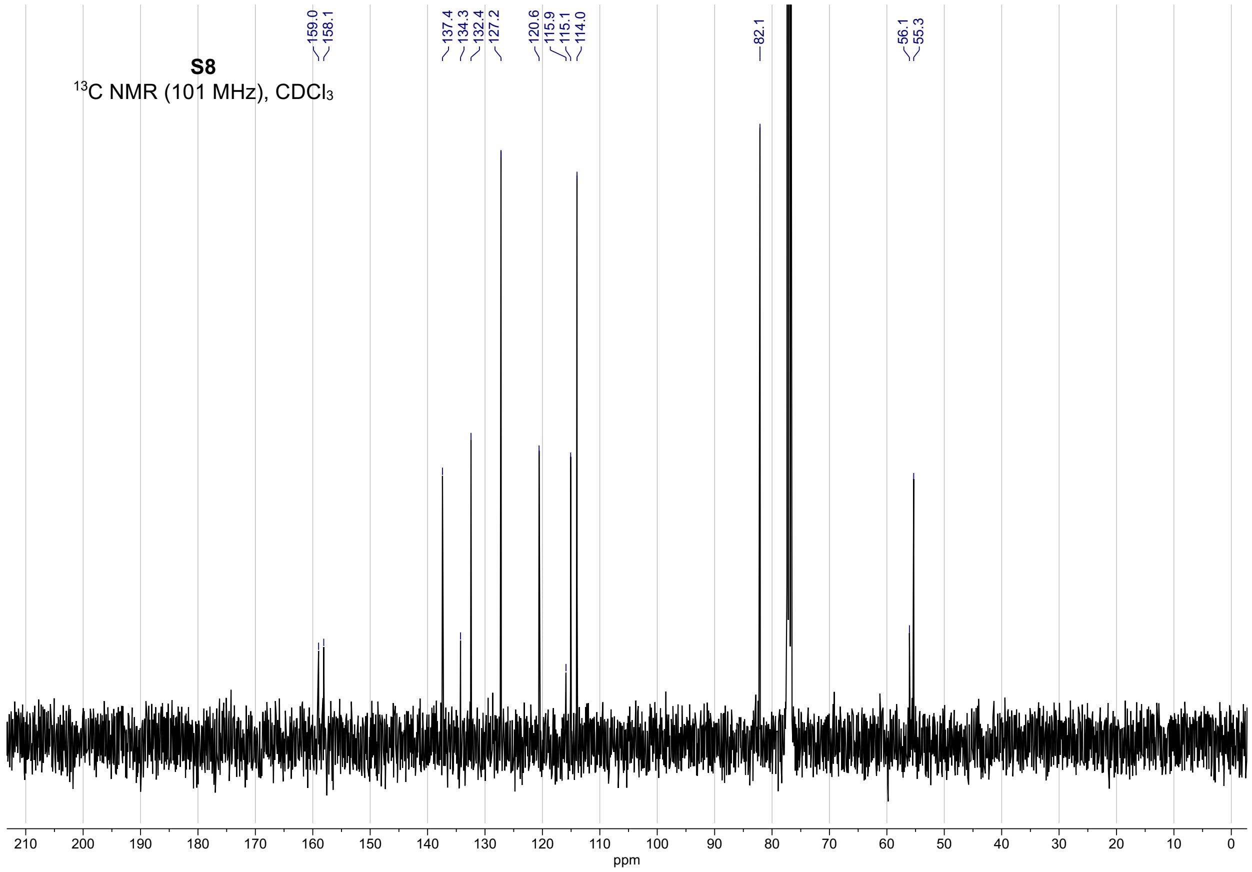

S8
^{13}C NMR (101 MHz), $CDCl_3$

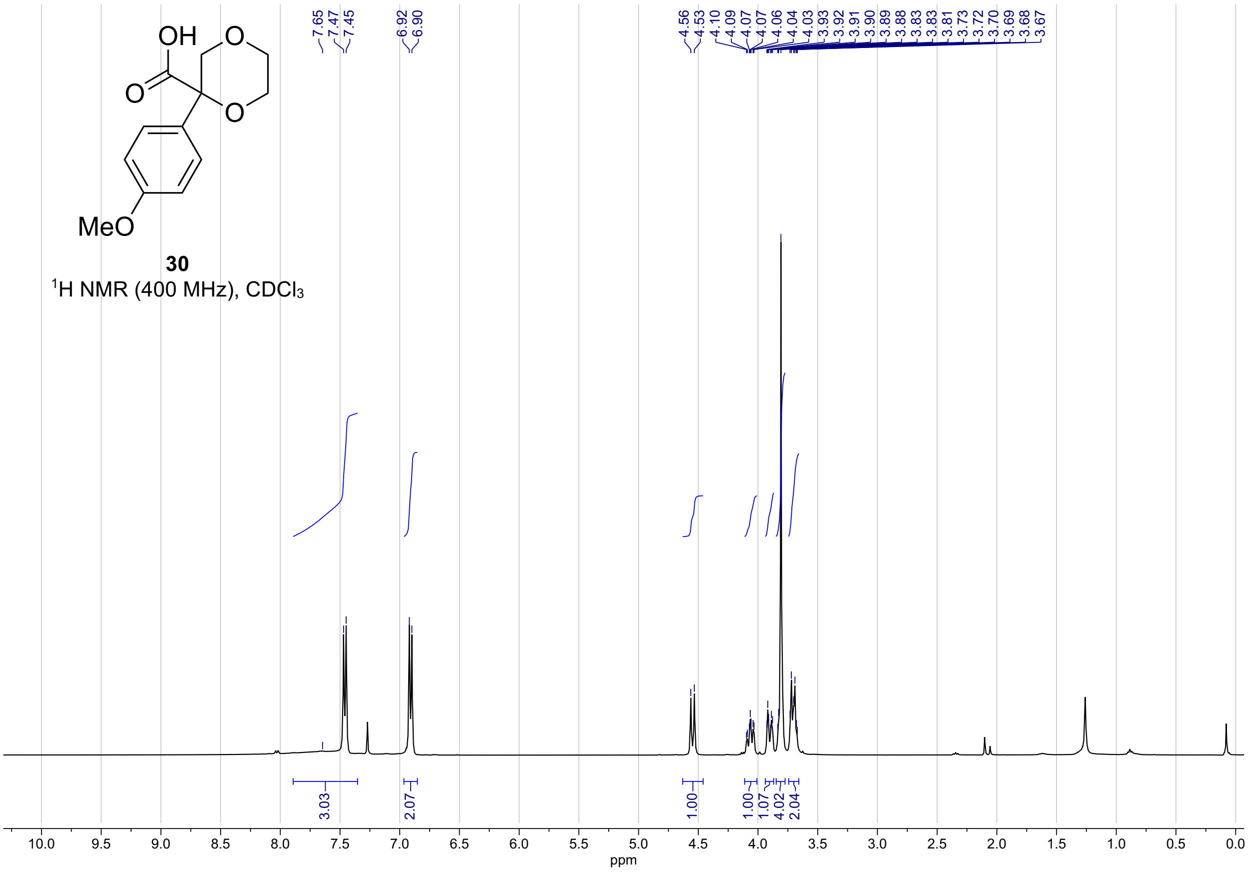

30
^{1}H NMR (400 MHz), $CDCl_3$

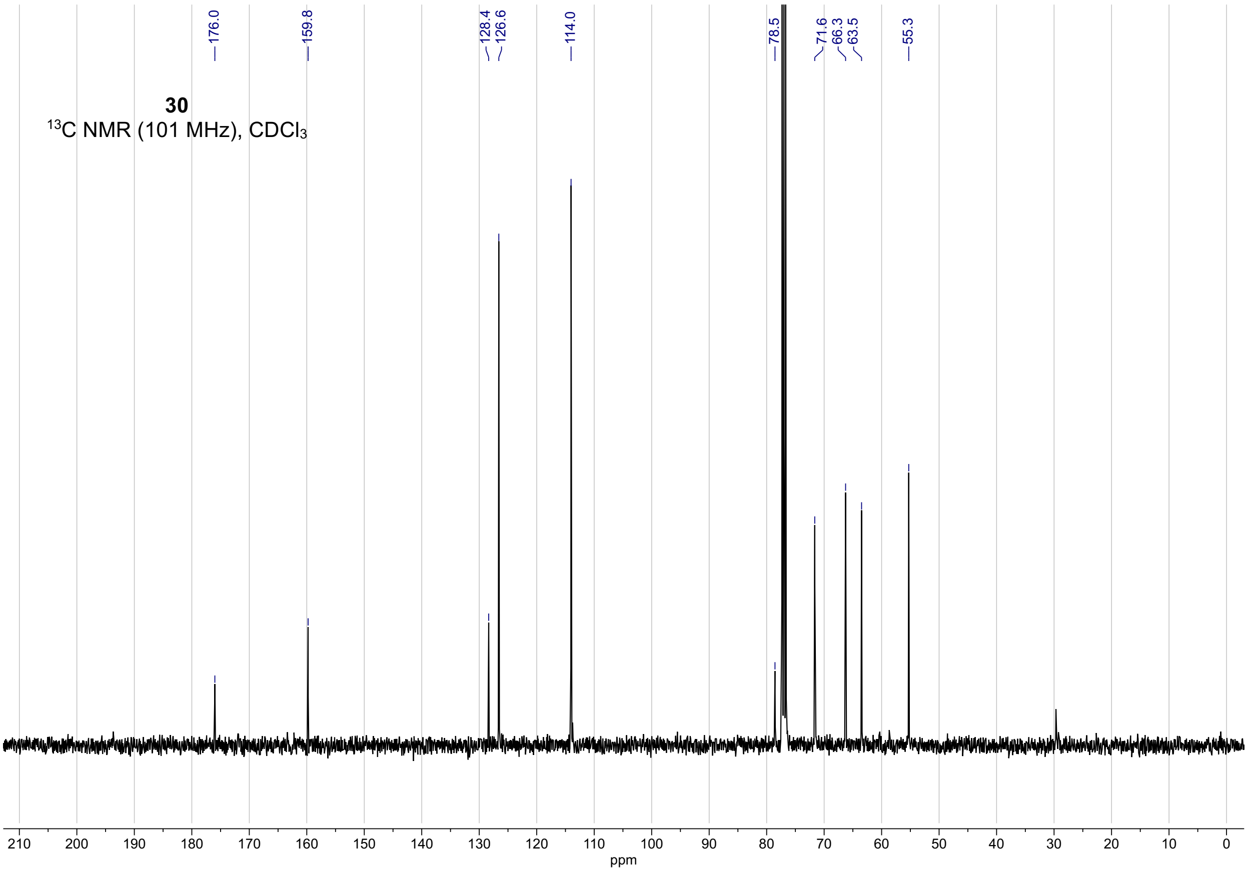

30
^{13}C NMR (101 MHz), $CDCl_3$

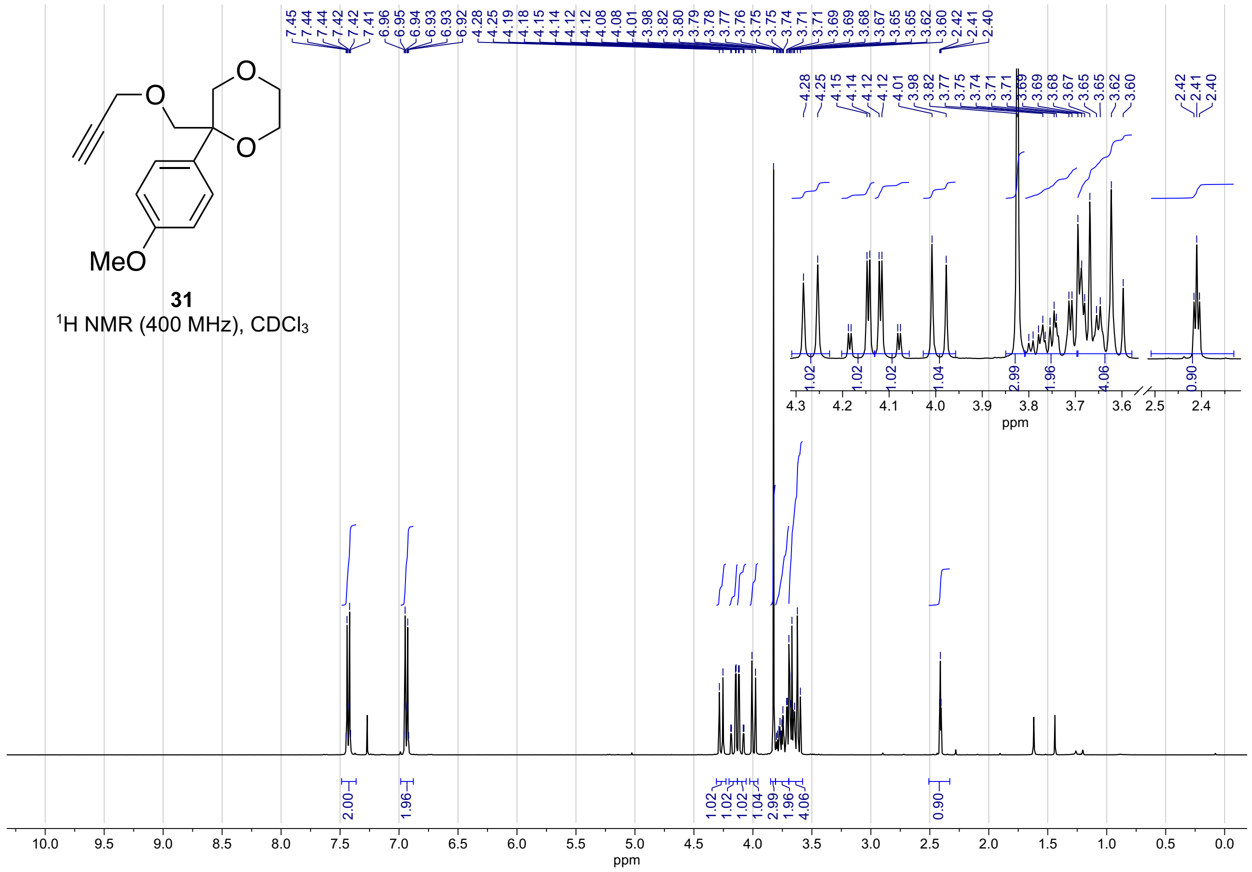

31
^{1}H NMR (400 MHz), $CDCl_3$

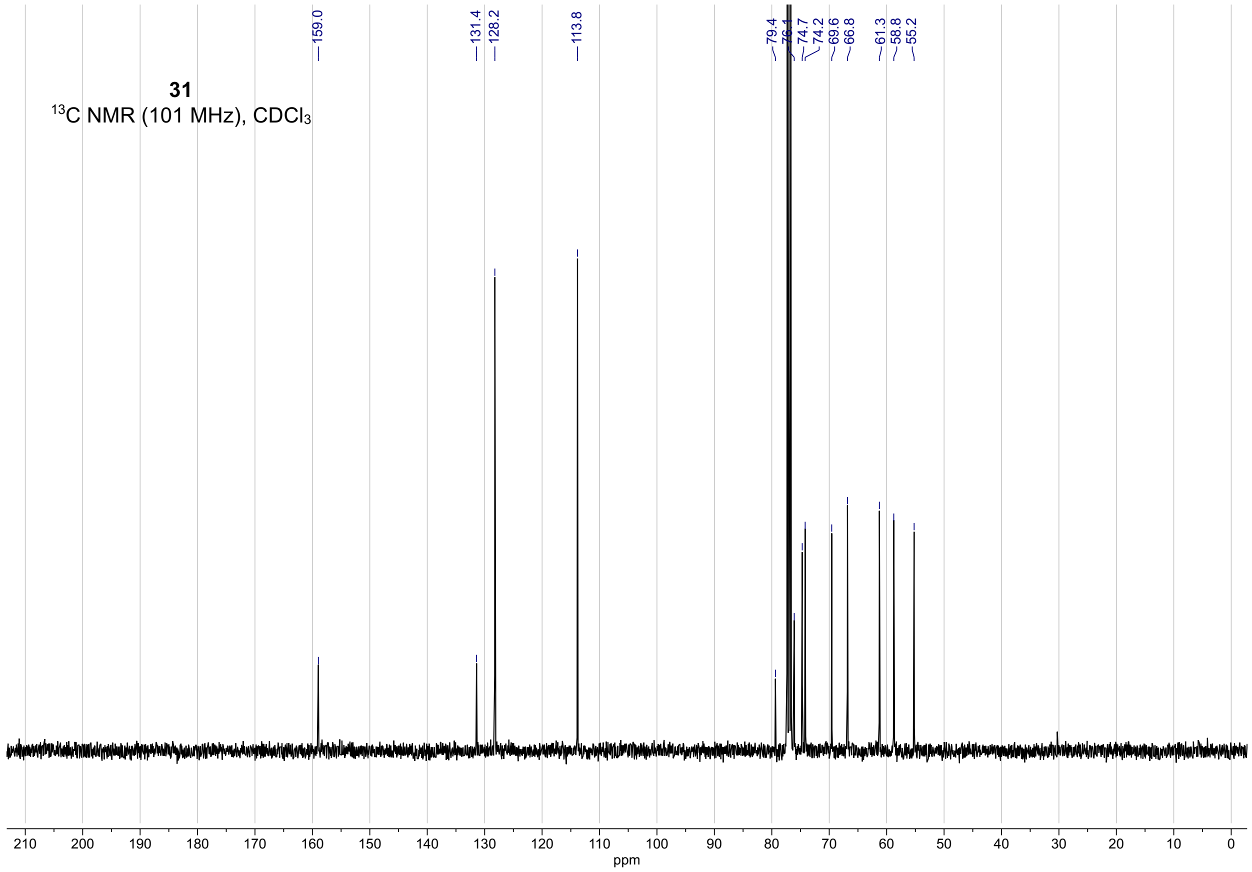

31
^{13}C NMR (101 MHz), $CDCl_3$

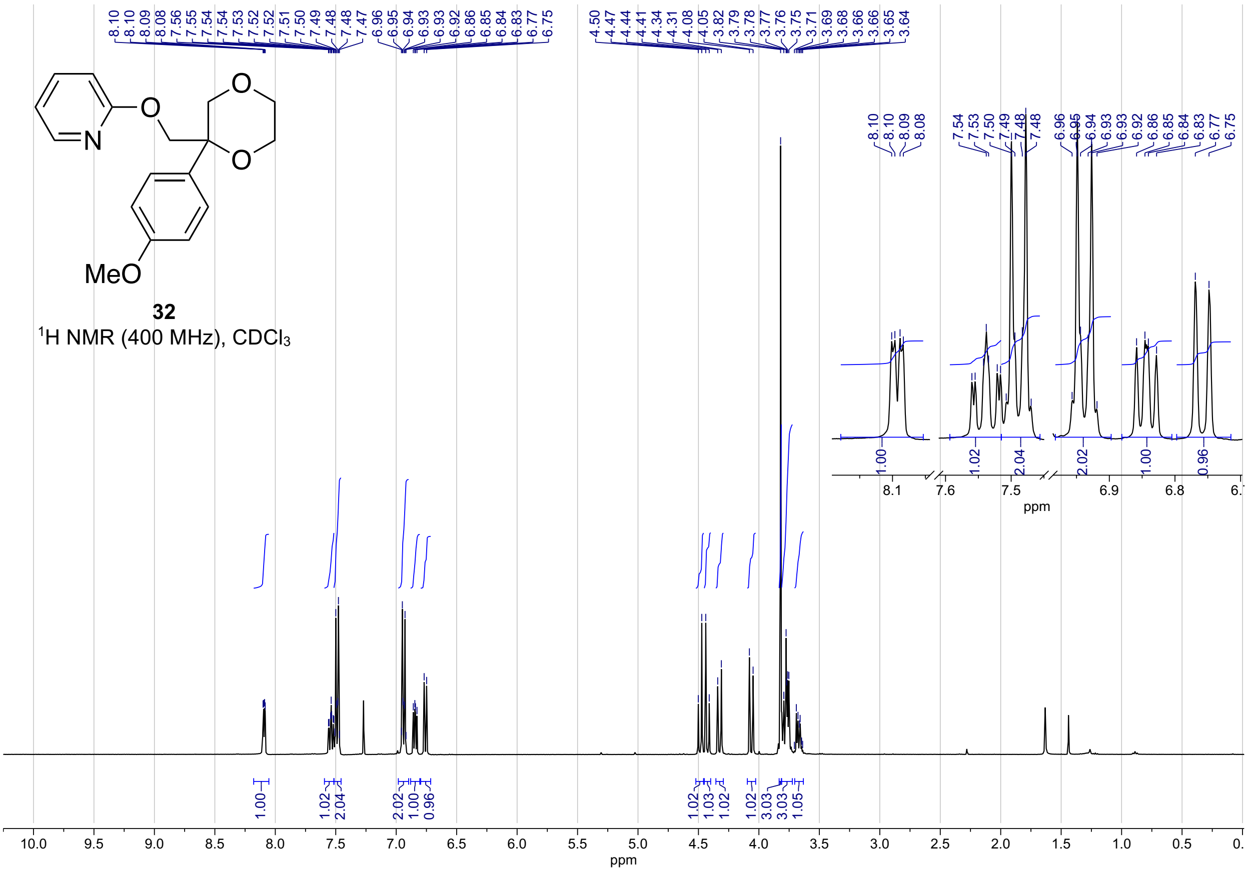

32

^{1}H NMR (400 MHz), $CDCl_3$

32

^{13}C NMR (101 MHz), $CDCl_3$

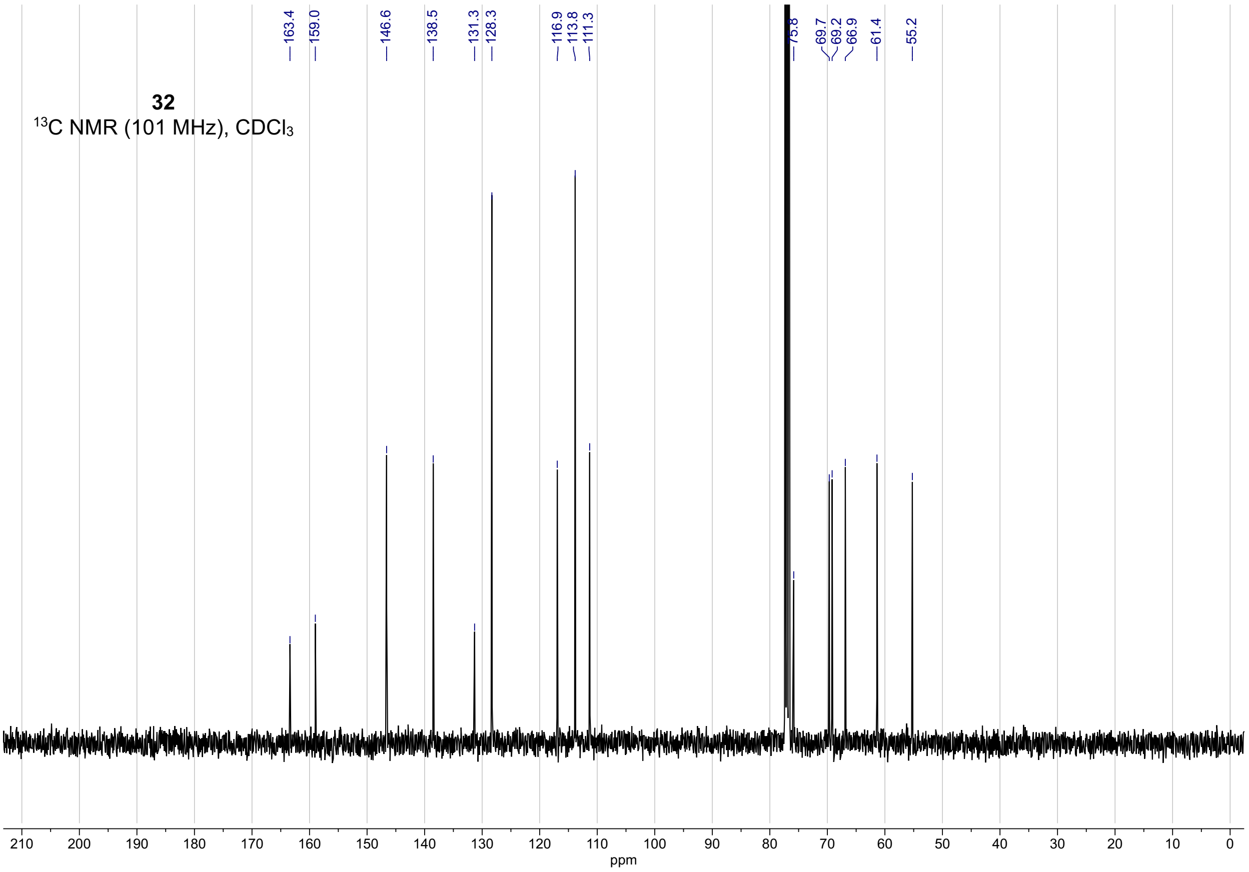

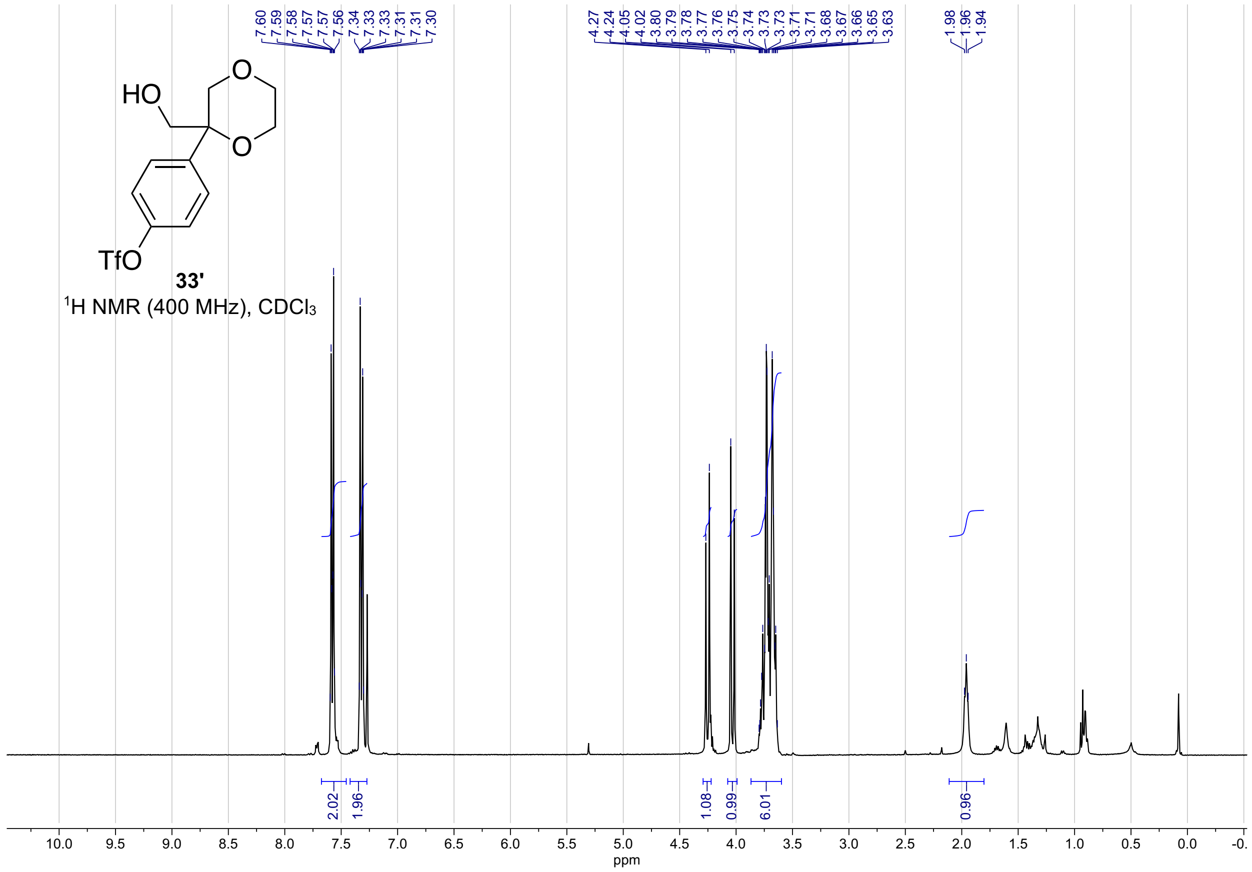
HO
O
O
TfO
33'
^{1}H NMR (400 MHz), $CDCl_3$
ppm

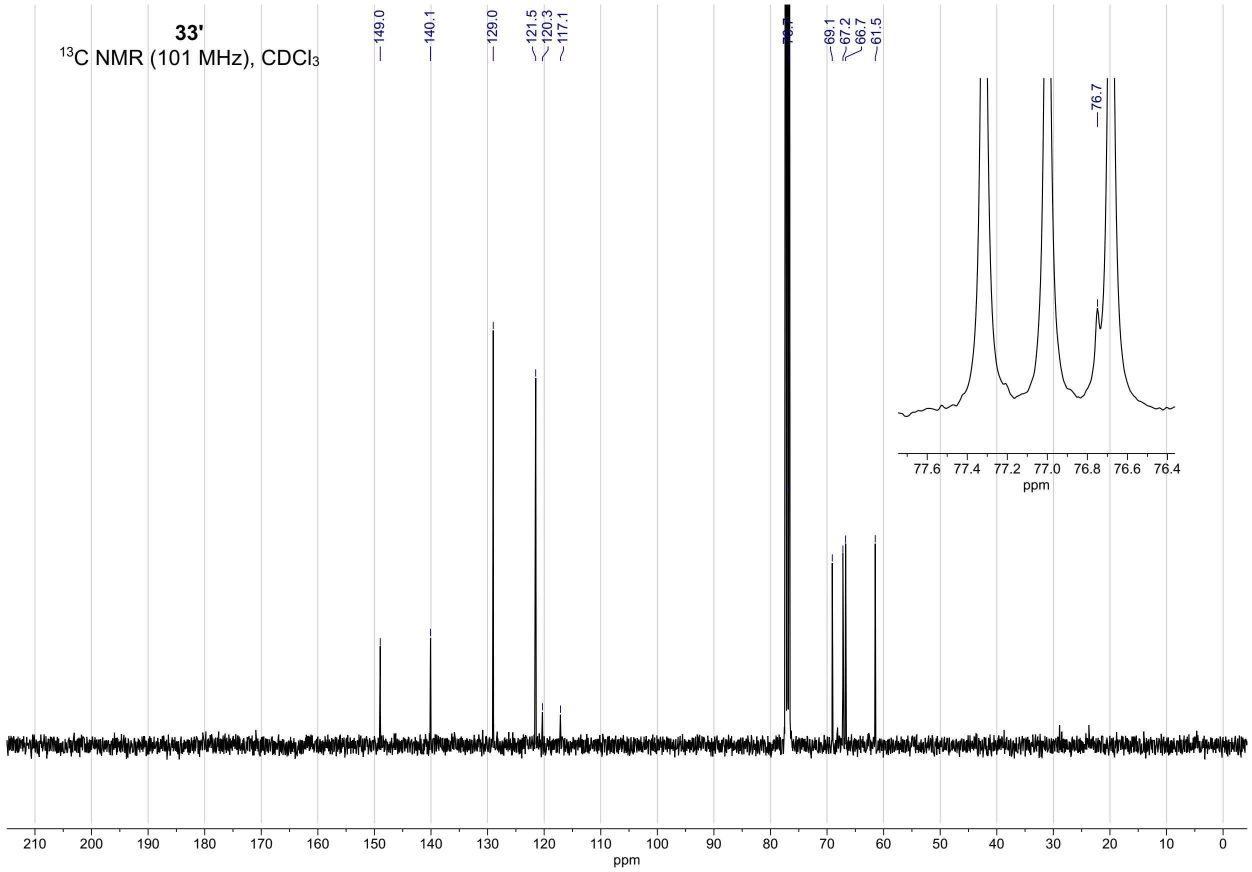
33'
^{13}C NMR (101 MHz), $CDCl_3$
149.0
140.1
129.0
121.5
120.3
117.1
69.1
67.2
66.7
61.5
76.7
ppm

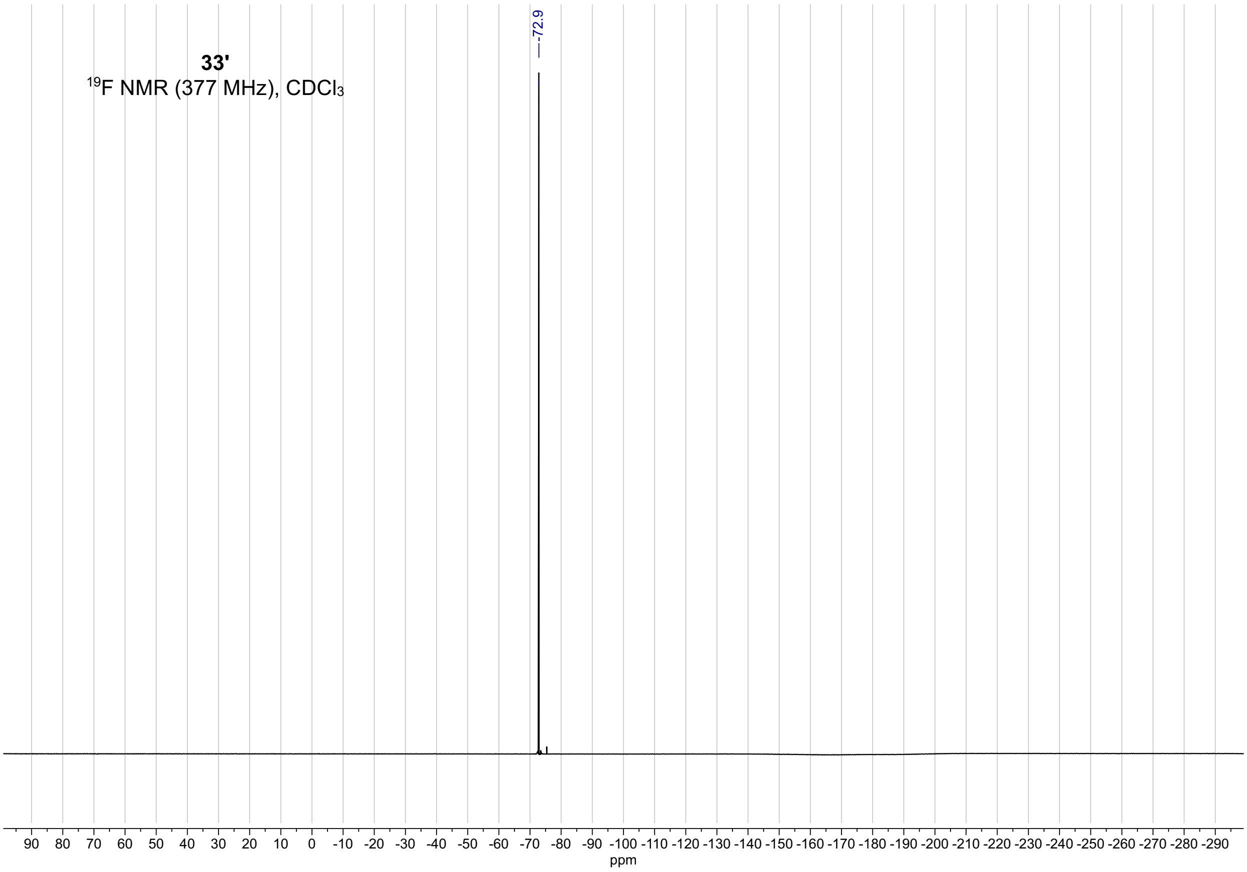
-72.9
33'
19F NMR (377 MHz), CDCl3
90 80 70 60 50 40 30 20 10 0 -10 -20 -30 -40 -50 -60 -70 -80 -90 -100 -110 -120 -130 -140 -150 -160 -170 -180 -190 -200 -210 -220 -230 -240 -250 -260 -270 -280 -290
ppm

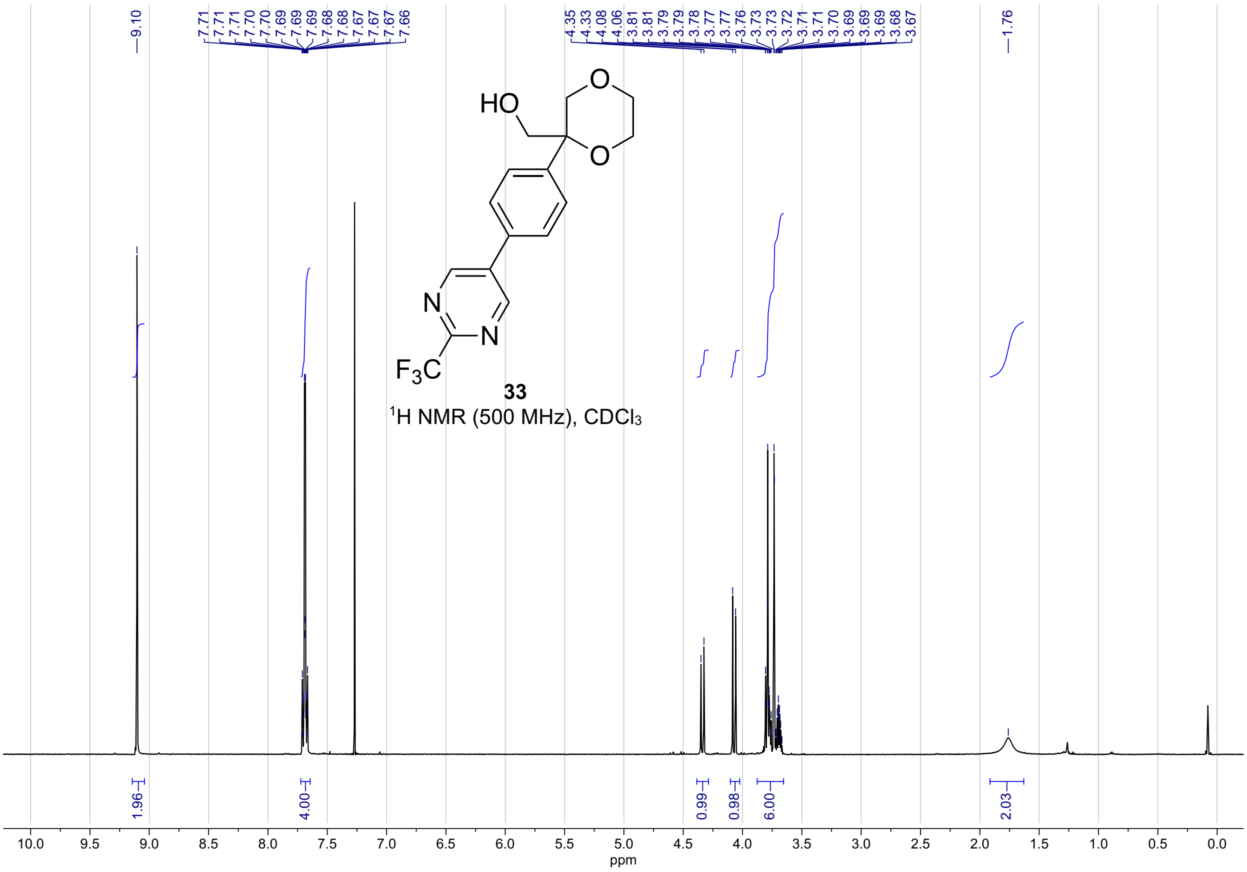
33
^{1}H NMR (500 MHz), $CDCl_3$

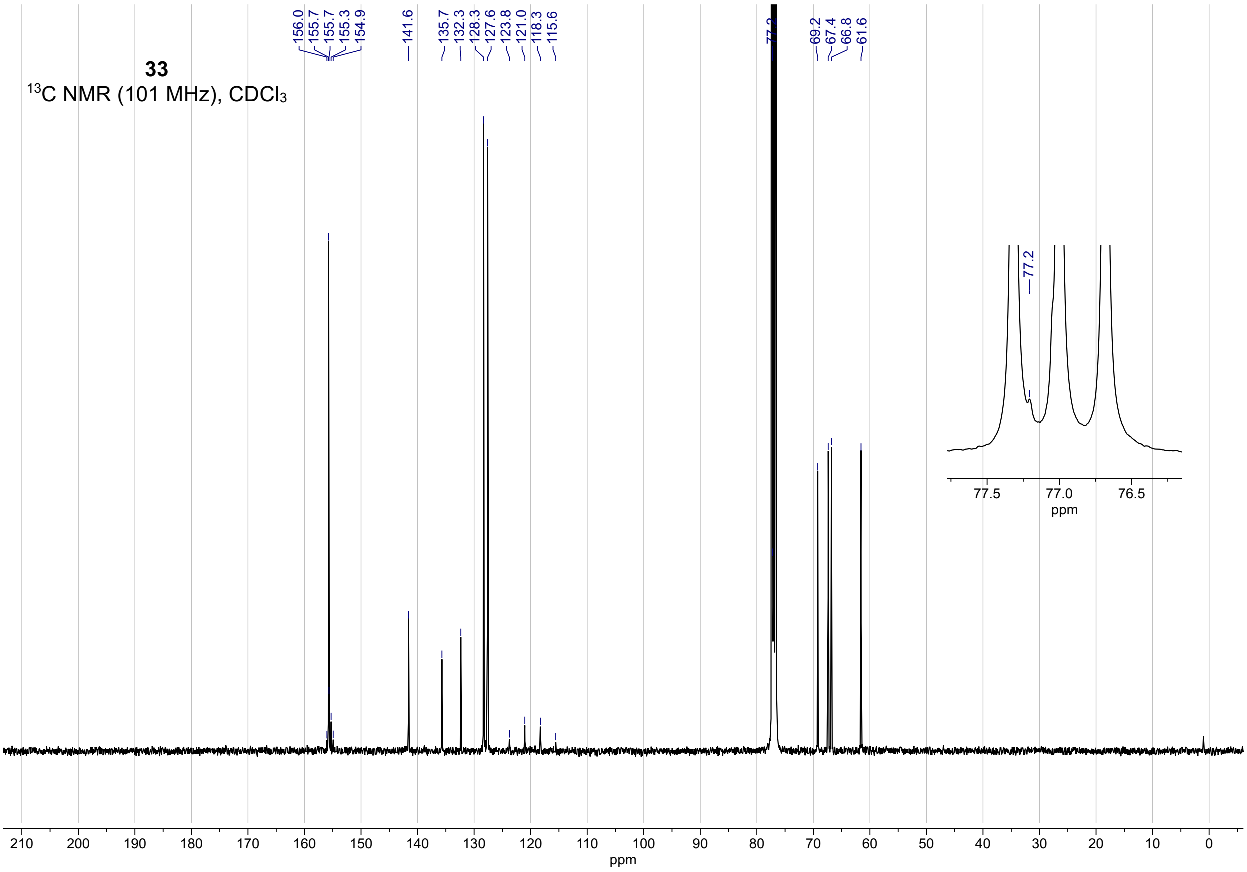
33
^{13}C NMR (101 MHz), $CDCl_3$

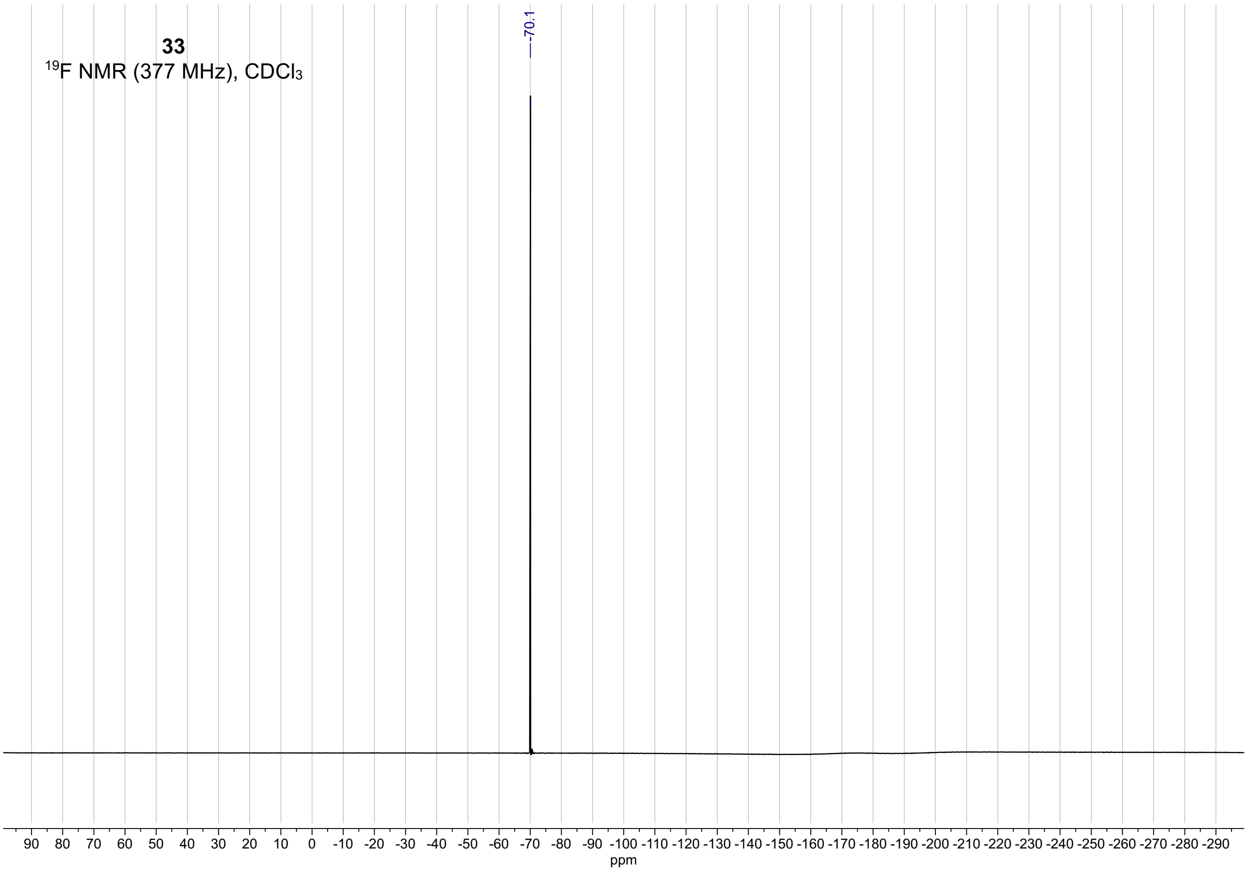
33
19F NMR (377 MHz), CDCl3
-70.1
90 80 70 60 50 40 30 20 10 0 -10 -20 -30 -40 -50 -60 -70 -80 -90 -100 -110 -120 -130 -140 -150 -160 -170 -180 -190 -200 -210 -220 -230 -240 -250 -260 -270 -280 -290
ppm

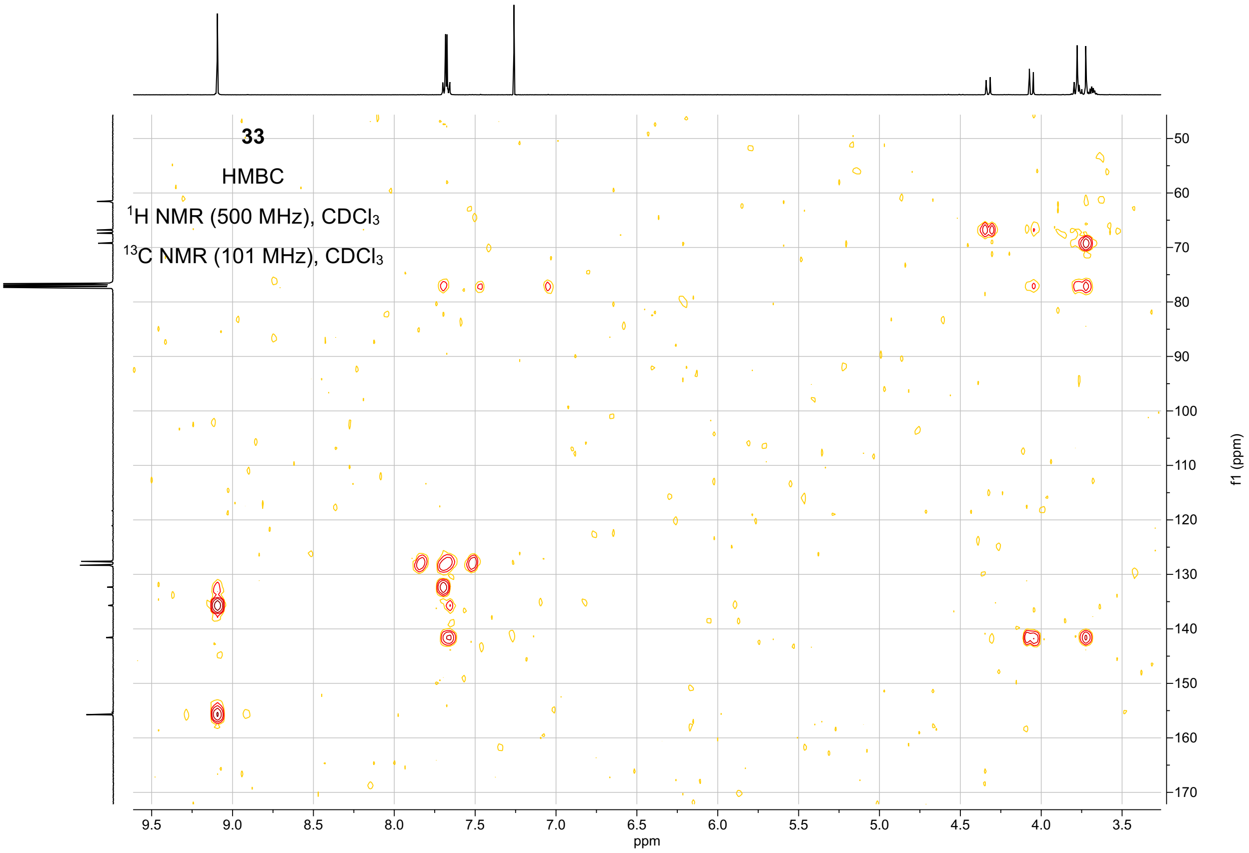
33
HMBC
1H NMR (500 MHz), CDCl3
13C NMR (101 MHz), CDCl3
9.5 9.0 8.5 8.0 7.5 7.0 6.5 6.0 5.5 5.0 4.5 4.0 3.5
ppm
50 60 70 80 90 100 110 120 130 140 150 160 170
f1 (ppm)

References

(1) Love, B. E.; Jones, E. G. *J. Org. Chem.* **1999**, *64*, 3755–3756.
(2) Croft, R. A.; Mousseau, J. J.; Choi, C.; Bull, J. A. *Chem. Eur. J.* **2016**, *22*, 16271–16276.
(3) Croft, R. A.; Mousseau, J. J.; Choi, C.; Bull, J. A. *Chem. Eur. J.* **2018**, *24*, 818–821.
(4) Croft, R. A.; Mousseau, J. J.; Choi, C.; Bull, J. A. *Tetrahedron* **2018**, *74*, 5427–5435.
(5) Croft, R. A.; Dubois, M. A. J.; Boddy, A. J.; Denis, C.; Lazaridou, A.; Voisin-Chiret, A. S.; Bureau, R.; Choi, C.; Mousseau, J. J.; Bull, J. A. *Eur. J. Org. Chem.* **2019**, *2019*, 5385–5395.
(6) Maji, R.; Champagne, P. A.; Houk, K. N.; Wheeler, S. E. *ACS Catal.* **2017**, *7*, 7332–7339.
(7) Rojas, J. J.; Croft, R. A.; Sterling, A. J.; Briggs, E. L.; Antermite, D.; Schmitt, D. C.; Blagojevic, L.; Haycock, P.; White, A. J. P.; Duarte, F.; Choi, C.; Mousseau, J. J.; Bull, J. A. *Nat. Chem.* **2022**. *Nat. Chem.* **2022**, *14,* 160–169.
(8) Dubois, M. A. J.; Smith, M. A.; White, A. J. P.; Lee Wei Jie, A.; Mousseau, J. J.; Choi, C.; Bull, J. A. *Org. Lett.* **2020**, *22*, 5279–5283.
(9) Liu, S.; Zang, Y.; Huang, H.; Sun, J. *Org. Lett.* **2020**, *22*, 8219–8223.
(10) Wuitschik, G. *Oxetanes in Drug Discovery*; Ph.D. Thesis, ETH Zürich, **2008**.
(11) Wang, Z.; Cui, Y.-T.; Xu, Z.-B.; Qu, J. *J. Org. Chem.* **2008**, *73*, 2270–2274.
(12) Chen, J.; Zhu, H.; Chen, J.; Le, Z.-G.; Tu, T. *Chem. Asian J.* **2017**, *12*, 2809–2812.
(13) Ismail, H. M.; Barton, V. E.; Panchana, M.; Charoensutthivarakul, S.; Biagini, G. A.; Ward, S. A.; O'Neill, P. M. *Angew. Chem. Int. Ed.* **2016**, *55*, 6401–6405.
(14) Qiu, G.; Wei, Y.; Lu, Y., Huang, Q.; Liao, P. *N substituted imidazole carboxylic acid ester compounds and preparation method thereof and purposes in medicine*, CN107382870A, 2017.
(15) Chapman, T. M.; Courtney, S.; Hay, P.; Davis, B. G. *Chem. Eur. J.* **2003**, *9*, 3397–3414.
(16) Stillings, M. R.; Chapleo, C. B.; Butler, R. C. M.; Davis, J. A.; England, C. D.; Myers, M.; Myers, P. L.; Tweddle, N.; Welbourn, A. P. *J. Med. Chem.* **1985**, *28*, 1054–1062.
(17) Pretsch, E.; Bühlmann, P.; Badertscher, M. in *Spektroskopische Daten zur Strukturaufklärung organischer Verbindungen*, 5th ed.; Springer, Berlin Heidelberg, **2010**.
(18) Préville, C.; Bonaventure, P.; Koudriakova, T.; Lord, B.; Nepomuceno, D.; Rizzolio, M.; Mani, N.; Coe, K. J.; Ndifor, A.; Dugovic, C.; Dvorak, C. A.; Coate, H.; Pippel, D. J.; Fitzgerald, A.; Allison, B.; Lovenberg, T. W.; Carruthers, N. I.; Shireman, B. T. *ACS Med. Chem. Lett.* **2020**, *11*, 2002–2009.
